# Atrial fibrillation and flutter in the Eastern Mediterranean: burden, disparities, and risk factor contributions from 1990 to 2021

**DOI:** 10.1186/s43044-025-00693-5

**Published:** 2025-10-22

**Authors:** Mohammad-Mahdi Bastan, Iman Elahi Vahed, Kamiar Izadpanah, Abedin Iranpour, Seyed Aria Nejadghaderi

**Affiliations:** 1 HIV/STI Surveillance Research Center, and WHO Collaborating Center for HIV Surveillance, Institute for Futures Studies in Health, Kerman University of Medical Sciences, Kerman, Iran; 2 Gastroenterology and Liver Diseases Research Center, Research Institute for Gastroenterology and Liver Diseases, Shahid Beheshti University of Medical Sciences, Tehran, Iran; 3 Knowledge Hub for Migrant and Refugee Health, Institute for Futures Studies in Health, Kerman University of Medical Sciences, Kerman, Iran

**Keywords:** Atrial fibrillation and flutter, Global burden of disease, Eastern Mediterranean Region, Disability-adjusted life years, Risk factors

## Abstract

**Background:**

Atrial fibrillation and flutter (AFF) are two of the most common cardiac tachyarrhythmias. The burden of AFF is increasing globally, with a particularly rapid increase in the Eastern Mediterranean region (EMR) due to economic and lifestyle changes. Despite extensive research on AFF, regional variations remain understudied.

**Aims:**

To analyze the burden and risk factors for AFF in the EMR using the Global Burden of Disease (GBD).

**Study design:**

Systematic analysis.

**Methods:**

Data from the GBD 2021 were used to evaluate the incidence, prevalence, disability-adjusted life years (DALYs), and deaths associated with AFF stratified by age, sex, and sociodemographic index (SDI) from 1990 to 2021. In addition, deaths and DALYs of AFF attributable to risk factors were estimated. Accompanying 95% uncertainty intervals (UIs) were provided to reflect the combination of data and estimates, and findings were presented as absolute counts and age-standardized rates.

**Results:**

From 1990 to 2021, the absolute number of incidence and prevalence of AFF in the EMR increased by 161.9% (from 64362.4 [48684.7–85763.7] in 1990 to 168555.0 [129706.9–220844.2] in 2021) and 162.4% (from 617721.6 [482076.0–807810.5] in 1990 to 1620763.1 [1270893.6–2108347.5] in 2021), respectively. From 1990 to 2021, the EMR revealed significant increases in the age-standardized incidence (3.0%, 95% UI 1.1–4.8) and prevalence (4.6%, 2.9–6.3) of AFF, with women experiencing greater increases than men did. The burden of AFF increased across all ages, with the incidence increasing by 161.9% and the prevalence increasing by 162.4%. From 2019 to 2021, the age-standardized rates of incidence, prevalence, DALYs, and deaths remained stable, with no significant changes. High systolic blood pressure was the leading risk factor, contributing to 25.5 age-standardized DALYs per 100,000 in 2021, whereas a high body mass index showed the largest increase in the attributable burden. Compared with low-SDI nations, high-SDI countries presented higher prevalence and incidence rates but lower death rates. Age-specific analysis revealed a sharp increase in the AFF burden with age, particularly among women aged 95+ years.

**Conclusion:**

The burden of AFF in the EMR increased from 1990 to 2021, with increasing incidence, DALY, and death rates. High systolic blood pressure and high body mass index are key contributors to these conditions. These findings underscore the need for public health interventions, including improved hypertension and obesity management, lifestyle modifications, and early detection strategies.

**Supplementary Information:**

The online version contains supplementary material available at 10.1186/s43044-025-00693-5.

## Introduction

Atrial fibrillation and flutter (AFF) are two of the most prevalent cardiac tachyarrhythmias affecting millions of people worldwide, significantly increasing the risk of stroke, heart failure, and death [1]. The prevalence of AFF has been increasing globally, driven by aging populations and an increasing burden of risk factors, including high systolic blood pressure (HSBP), high body mass index (HBMI), alcohol use, high dietary sodium intake, smoking, lead exposure, and aging [2–4].

The growing burden of AFF poses a challenge to global health systems, leading to a significant increase in healthcare costs and the consumption of social resources. Furthermore, the development of AFF is related to socioeconomic and cultural factors, with economic globalization influencing disease patterns. In some regions, the disparity between rapid economic growth and lower levels of education and health awareness has contributed to rising incidence and death rates [5, 6].

The Eastern Mediterranean Region (EMR) presents a unique and important context for studying AFF due to distinct demographic and clinical characteristics compared to Western populations [7, 8]. Patients with AFF in the countries of the region tend to be a decade younger on average but carry a higher burden of cardiovascular risk factors such as diabetes, obesity, smoking, and rheumatic valvular heart disease. These comorbidities, together with an observed high prevalence of undertreatment or lack of anticoagulation in moderate to high stroke risk patients, place this population at an increased risk of complications like stroke.

Despite the global recognition AFF as a major public health concern, there is a relative paucity of robust epidemiological data and clinical outcome studies from the EMR. This gap limits evidence-based clinical management and stroke prevention strategies tailored to the unique characteristics of this region’s population. We focus on the EMR due to its distinct demographic and clinical profile, including an earlier onset of AFF, higher prevalence of comorbidities such as diabetes and hypertension, and undertreatment, particularly concerning thrombo-prophylaxis. By evaluating these factors, this study seeks to provide region-specific insights that can inform the development of relevant clinical practice guidelines. We report the burden of AFF and the related risk factors in the EMR from 1990 to 2021 by age, sex, and sociodemographic index (SDI) using data from the Global Burden of Disease (GBD) 2021.

## Methods

### Overview

The GBD 2021 is a comprehensive research initiative that assesses the burden of diseases, injuries, and risk factors across the globe [9]. It encompasses data from 204 countries and territories, covering a wide range of health metrics from 1990 to 2021. This extensive dataset allows for the estimation of incidence, prevalence, death, and disability-adjusted life years (DALYs) for various health conditions. The scope of GBD studies is vast, addressing 371 diseases and injuries, along with 88 risk factors [10, 11]. It provides a detailed view of health disparities across different demographics, including age, sex, and socioeconomic status.

### Case definitions

Atrial fibrillation is a supraventricular arrhythmia caused by disorganization of the atrium [12]. Atrial flutter is a macroreentrant supraventricular arrhythmia that usually involves the cavo-tricuspid isthmus. Diagnosis requires an electrocardiogram demonstrating (1) irregularly irregular RR intervals (in the absence of complete atrioventricular block); (2) no distinct P waves on the surface electrocardiogram; and (3) an atrial cycle length (when visible) that is usually variable and less than 200 ms. The GBD study relies on International Classification of Diseases (ICD) codes for identifying health conditions, but changes in these codes over time can lead to inconsistencies in disease identification. The ICD has undergone several revisions, notably from ICD-9 to ICD-10, altering how diseases are classified and recorded, which can affect epidemiological estimates. These changes can create variability in case definitions, leading to different prevalence rates that reflect coding changes rather than actual changes in disease incidence. Additionally, for longitudinal studies, shifts in case definitions complicate the assessment of trends over time, requiring adjustments to reconcile differing datasets effectively. To address inconsistencies from changes in ICD codes, the GBD study employs several methodological strategies. The GBD study utilizes reclassification approaches by conducting systematic reviews and leveraging empirical evidence to update disease definitions according to the latest ICD versions, which helps harmonize definitions across different time periods and reduces the impact of variable diagnostics. Moreover, to manage variability in disease classification, GBD study employs the Disease Modeling Meta-Regression version 2.1 (DisMod-MR 2.1) framework, which harmonizes disparate data sources by borrowing strength across comparable healthcare systems and regions, imputing missing information using covariate-based predictive modeling, and enforcing epidemiological consistency among incidence, prevalence, and mortality estimates. In addition, heterogeneity classification was reduced through a hierarchical data-quality scheme that prioritizes population-based registries with active case ascertainment, followed by audited hospital registries, validated administrative databases, and survey/model-based estimates. Temporal pattern harmonization was incorporated to reflect the dynamic nature of AFF classification, with clinical progression modeling to account for transitions (e.g., paroxysmal to persistent), cross-sectional adjustments to reflect point-in-time distributions across disease stages, and the inclusion of treatment response parameters (cardioversion and ablation success) that influence reclassification. All estimates were age-standardized to align differences in population structure and ensure comparability across settings. Region- and system-specific adaptations were also implemented to address sources of misclassification and under-ascertainment pertinent to the EMR. These included adjustments for conflict-related service disruptions that affect case capture, accommodations for resource constraints such as limited electrocardiogram access and specialist availability, and recognition of sex-specific access barriers that influence detection. Geographic stratification incorporated distance-to-care effects, technology availability in rural settings, and referral patterns that introduce diagnostic delay. Robustness was evaluated through multi-source external validation alongside demographic consistency checks and temporal trend validation against known epidemiological transitions. Lastly, the GBD study robustly communicates uncertainty by quantifying the variability associated with case definitions and the specific data sources of different ICD versions, incorporating credible intervals to reflect uncertainties in health metric interpretations due to evolving definitions. The ICD codes used for inclusion of hospitals and claims are I48-I48.9 for the ICD-10 and 427.3 for the ICD-9. The GBD study employs a comprehensive array of data sources to analyze health metrics, disease burdens, and risk factors across diverse populations [12]. It primarily relies on primary data collection, including surveys, interviews, and observational studies, which are critical for estimating disease incidence and prevalence, particularly in regions with weak vital registration systems [10]. The GBD study relies on the integration and reconciliation of diverse data sources to produce reliable estimates of disease burden, death, and morbidity. To enhance the accuracy of these estimates, the GBD study employs systematic standardization of data to ensure consistency in age groups, geographical classifications, and disease definitions, using the ICD codes. These integrated efforts significantly enhance the robustness of health metrics used to understand global health challenges.

### Modeling strategy

The burden of AFF was estimated via the GBD’s standardized modeling approach, which incorporates a systematic review of available data. The GBD employs a variety of modeling frameworks, including Cause of Death Ensemble Modeling (CODEm), which estimates death by combining multiple data sources and statistical techniques to produce robust estimates of deaths attributable to AFF. The CODEm is a component of the GBD study, designed to provide reliable estimates of mortality and underlying causes of death across various populations. By integrating diverse data sources, CODEm enhances the accuracy of cause-specific mortality estimates, particularly for conditions that are often underreported or misclassified. Its methodology employs Bayesian hierarchical modeling to handle complex relationships among various covariates and combine multiple data inputs, allowing for a more nuanced understanding of mortality trends. This ensemble approach not only improves predictive accuracy by weighting the best-performing models but also facilitates both temporal and spatial analyses, capturing variations in cause-specific mortality over time and across different locations. The application of CODEm has proven essential in identifying priority health issues, thereby informing health policy and resource allocation globally. The continuous refinement of CODEm remains crucial as global health landscapes evolve, ensuring that the estimates remain relevant and can effectively guide public health strategies and interventions. CODEm serves as a cornerstone of the GBD study, enabling a clearer understanding of global health burdens and contributing valuable insights for improving health outcomes worldwide [12]. In addition, spatiotemporal Gaussian process regression (SGPR) models are used for the outcome variable, either the death rate or cause fraction, via various combinations of predictive covariates [12]. SGPR models are essential in analyzing health outcomes within GBD studies, combining the strengths of Gaussian processes with spatial and temporal modeling to address the complexities of health data variations. These models allow for precise predictions of disease incidence and prevalence by utilizing carefully selected covariance functions that capture spatial correlations and temporal trends. Their effectiveness hinges on robust data integration, allowing for the amalgamation of heterogeneous datasets from various sources, which is crucial for regions with sparse data. SGPR models enhance predictive capabilities by incorporating relevant covariates, estimating uncertainty around predictions, and rigorously accounting for spatial autocorrelation, thereby improving the accuracy of health outcome projections. This methodology facilitates detailed regional health assessments, estimates cause-specific mortality, and enables the forecasting of future health trends, making SGPR a powerful tool for informed public health interventions and resource allocation decisions [10].

### Sociodemographic Index (SDI)

The SDI is a composite measure that reflects the socioeconomic development of countries and regions [11]. It is calculated on the basis of three key indicators: income per capita, educational attainment, and total fertility rate. Countries are categorized into five SDI quintiles (i.e., low SDI, low-middle SDI, middle SDI, high-middle SDI, and high SDI), which helps to illustrate the disparities in health burdens and access to healthcare services.

### Risk factors

The analysis considered risk factors associated with AFF, including alcohol use, a diet high in sodium, HBMI, HSBP, lead exposure, and smoking. The GBD employs a comprehensive risk assessment framework that includes data collection on risk factors from national health surveys and epidemiological studies. Risk–outcome pairs that have a probable causal relationship between the risk factor and the outcome were identified and analyzed. An attributable risk calculation was performed to estimate the proportion of deaths and DALYs attributable to each risk factor via population-attributable fractions derived from the data sources. Moreover, relative risk estimates were calculated to quantify the strength of the association between risk factors and the incidence of AFF. Further details about the risk factors are provided elsewhere [11].

### Statistical analysis

Due to the nature of this study, which involves secondary data analysis utilizing the comprehensive datasets provided by the GBD study, a formal sample size calculation is not applicable. The GBD database aggregates data from multiple sources, including vital registration systems, health surveys, and epidemiological studies, enabling robust population-level estimates without the need for primary sample considerations. Furthermore, the analysis included information on different age and sex groups. The methodology for calculating age-standardized rates per 100,000 population involved direct standardization. The GBD world population age standard in the GBD study 2021 was used as the reference for standardization [12]. Uncertainty intervals (UIs) were calculated using a Monte Carlo simulation approach, which involve running multiple iterations with varied input parameters and distributions to account for variability and generate credible intervals around the burden estimates. This method allows for an assessment of uncertainty, helping to capture the full range of potential outcomes and ensuring that the findings are statistically robust and reflective of the inherent variability within the data. The reported values were represented in point estimation accompanied by the 95% UIs extracted using the 25th and 975th ranked draws of the uncertainty distribution by taking 500 samples from the posterior distribution. Data analysis was completed with Python (version 3.10.4), Stata (version 13.1), and R (version 4.5.1).

## Results

### Regional burden of AFF

**Fig. 1:**
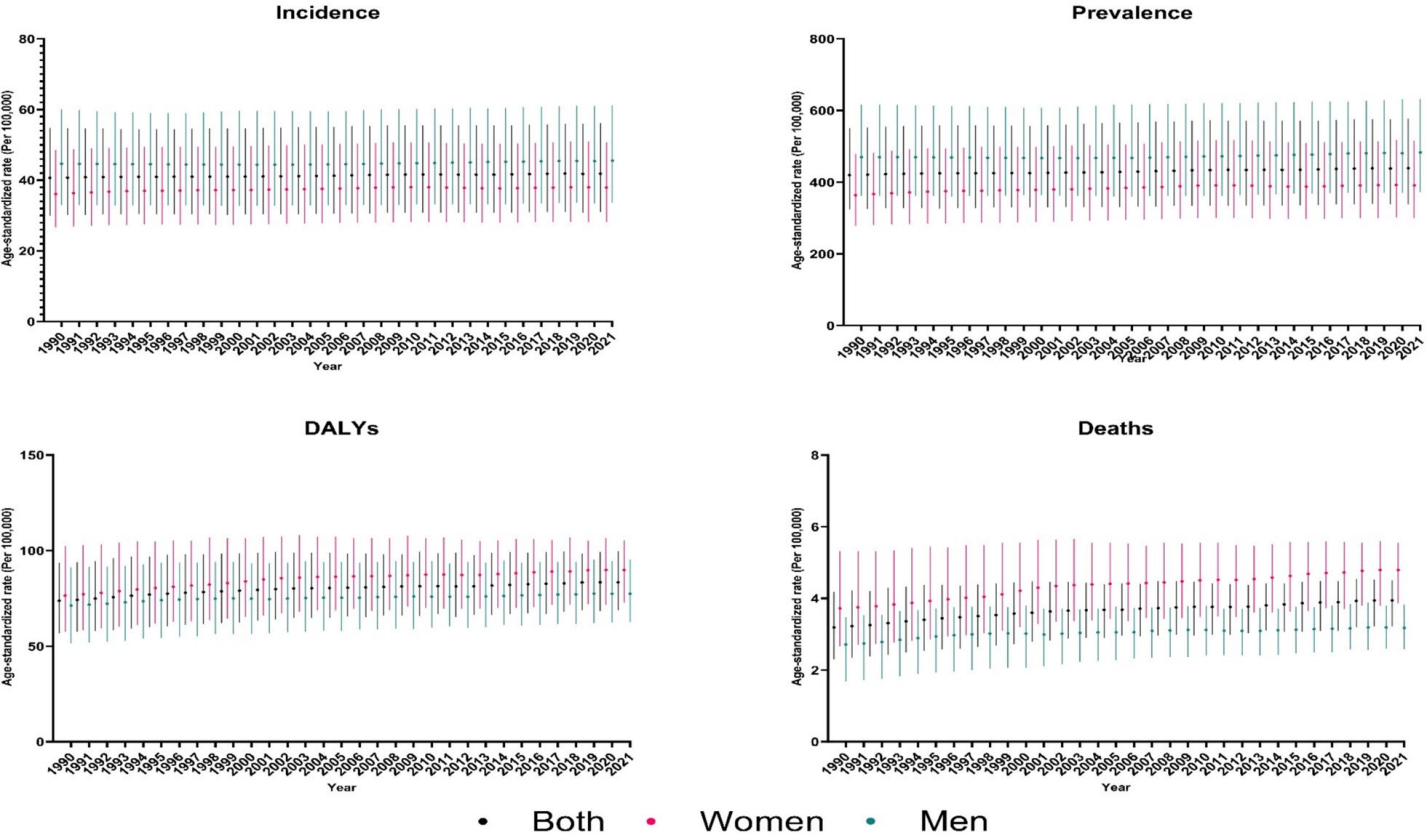
Time trend of age-standardized rate of incidence, prevalence, disability-adjusted life years (DALYs), and deaths of atrial fibrillation and flutter in the Eastern Mediterranean Region from 1990 to 2021, by sex

From 1990 to 2021, the EMR experienced increases in the age-standardized incidence and prevalence of AFF. The age-standardized incidence rate rose by 3.0% (95% UI 1.1–4.8), from 40.6 (30.0– 54.8) in 1990 to 41.8 (31.0–56.1) in 2021. Similarly, the age-standardized prevalence rate grew by 4.6% (2.9–6.3), from 419.7 (324.2–551.0) in 1990 to 439.0 (338.8–577.0) in 2021. However, changes in age-standardized DALYs and deaths were non-significant, with age-standardized DALYs elevated by 13.1% (− 2.8 to 30.6) and age-standardized deaths by 23.7% (− 4.0 to 60.1). All-ages numbers showed substantial growth, with all-ages incidence surged by 161.9% (154.7–170.3), all-ages prevalence increased by 162.4% (156.3–168.5), the all-ages DALYs rose by 178.2% (141.4–218.6), and the all-ages deaths grew by 207.1% (138.6–297.6) (Table 1 and Fig. 1).

**Table 1:** All‑ages number and age‑standardized rate of incidence, prevalence, disability-adjusted life years (DALYs), and deaths of atrial fibrillation and flutter by sex in 1990 and 2021 and overall percent change over 1990–2021 in the Eastern Mediterranean Region

Measure	Age, Metric	Year						% Change (1990–2021)		
		1990			2021					
		Both	Women	Men	Both	Women	Men	Both	Women	Men
Incidence	Age-standardized	40.6 (30–54.8)	36.1 (26.6–48.5)	44.7 (33–60.1)	41.8 (31–56.1)	37.9 (28.2–50.7)	45.6 (33.7–61.2)	3 (1.1–4.8)	5 (3.1–6.8)	2 (− 0.7 to 4.4)
	All ages	64,362.4 (48,684.7–85,763.7)	26,832.3 (20,314.9–35,807)	37,530 (28,263–49,849.2)	168,555 (129,706.9–220,844.2)	73,645.7 (56,223.9–97,185.5)	94,909.3 (73,081–124,276)	161.9 (154.7–170.3)	174.5 (168.6–181.2)	152.9 (143.6–162.9)
Prevalence	Age-standardized	419.7 (324.2–551)	363.7 (278.2–478.8)	470 (361.7–616.3)	439 (338.8–577)	391.3 (299.9–515.5)	483.3 (372.4–632.3)	4.6 (2.9–6.3)	7.6 (5.7–9.5)	2.8 (0.1–5.3)
	All ages	617,721.6 (482,076–807,810.5)	252,173.5 (193,436.4–332,030)	365,548.1 (285,244.4–475,780.5)	1,620,763.1 (1,270,893.6–2,108,347.5)	698,891.7 (539,905.9–917,111.1)	921,871.4 (724,649.8–1,194,163.1)	162.4 (156.3–168.5)	177.1 (171.7–183.1)	152.2 (143.9–160.5)
DALYs	Age-standardized	73.7 (56.6–93.6)	76.5 (57.6–102.3)	71.3 (51.5–91.3)	83.4 (68.7–99.8)	89.9 (72.9–105.4)	77.5 (62.6–95.2)	13.1 (− 2.8 to 30.6)	17.5 (− 4.5 to 38.3)	8.7 (− 3.8 to 31)
	All ages	100,324.1 (76,902.1–127,934)	48,467.6 (36,633.7–64,013.4)	51,856.5 (37,467.3–66,898.7)	279,136.9 (229,153.6–335,913.4)	141,800.5 (115,263.1–171,031.8)	137,336.4 (109,551.2–170,691.1)	178.2 (141.4–218.6)	192.6 (139.9–241.2)	164.8 (135.2–214.5)
Deaths	Age-standardized	3.2 (2.3–4.2)	3.7 (2.7–5.3)	2.7 (1.7–3.5)	3.9 (3.2–4.5)	4.8 (3.9–5.6)	3.2 (2.6–3.8)	23.7 (− 4 to 60.1)	−	17.2 (− 7.1 to 71.3)
	All ages	3361.1 (2447.1–4411.3)	1872.2 (1341.1–2679.7)	1488.9 (926.6–1896.6)	10,320.6 (8504.4–11,780.6)	5967.1 (4845.1–6938.7)	4353.4 (3593.4–5204.6)	207.1 (138.6–297.6)	218.7 (133.9–309.3)	192.4 (131.2–329.4)

### Sex-specific burden of AFF

Compared with men, women in the EMR experienced a greater elevation in the age-standardized burden of AFF. The age-standardized incidence rate for women surged by 5.0% (95% UI 3.1–6.8), compared with a nonsignificant increase of 2.0% (− 0.7 to 4.4) for men. Similarly, the age-standardized prevalence rate rose by 7.6% (5.7–9.5) for women, whereas men experienced a smaller grow of 2.8% (0.1–5.3). The changes in age-standardized DALYs and deaths were not significant for both sexes. The age-standardized DALY rates elevated by 17.5% (− 4.5 to 38.3) for women and 8.7% (− 3.8 to 31.0) for men, whereas age-standardized deaths surged by 28.9% (− 5.8 to 65.9) for women and 17.2% (− 7.1 to 71.3) for men. All-age numbers significantly increased for both sexes, with the all-age incidence rose by 174.5% (168.6–181.2) for women and 152.9% (143.6–162.9) for men (Table 1).

### Time trend of age-standardized rates of AFF in the EMR

The burden of AFF in the EMR has grown over time across age-standardized rates of incidence, prevalence, DALYs, and deaths. In both sexes, the age-standardized incidence rate elevated from 40.6 (95% UI 30.0–54.8) in 1990 to 41.8 (31.0–56.1) in 2021. Men consistently presented higher age-standardized incidence rates than women did, with rates surged from 44.7 (33.0–60.1) in 1990 to 45.6 (33.7–61.2) in 2021, whereas in women, age-standardized rates fluctuated slightly, ending at 37.9 (28.2–50.7) in 2021. The age-standardized prevalence rate increased from 419.7 (324.2–551.0) in 1990 to 439.0 (338.8–577.0) in 2021. Men had a higher age-standardized prevalence than women did, with values rising from 470.0 (361.7–616.3) in 1990 to 483.3 (372.4–632.3) in 2021, and women's age-standardized prevalence increased from 363.7 (278.2–478.8) in 1990 to 391.3 (299.9–515.5) in 2021 (Fig. 1 and Table S1).

The age-standardized rate of DALYs also showed a growing trend, from 73.7 (56.6–93.6) in 1990 to 83.4 (68.7–99.8) in 2021. Women consistently had a higher age-standardized DALY rate than men did, and a rising trend was also evident in the death rate, which elevated over the years. The age-standardized death rate for both sexes surged from 3.2 (2.3–4.2) in 1990 to 3.9 (3.2–4.5) in 2021, with a consistently higher age-standardized death rate among women. Age-standardized death rates among women rose from 3.7 (2.7–5.3) in 1990 to 4.8 (3.9 to 5.6) in 2021, whereas men experienced a more modest increase from 2.7 (1.7–3.5) in 1990 to 3.2 (2.6–3.8) in 2021 (Fig. 1 and Table S1).

### Regional and country-level variations in burden of AFF during the COVID-19 pandemic

During the COVID-19 pandemic (2019–2021), the age-standardized rates of incidence, prevalence, DALYs, and deaths due to AFF in the EMR remained largely stable, with no significant changes. The age-standardized incidence rate showed minimal variation, from 41.9 (95% UI 31.1–55.9) in 2019 to 41.8 (31.0–56.1) in 2021, with no statistically significant percent change. Similarly, the age-standardized prevalence rate remained steady, at 438.8 (339.2–575.1) in 2019 and 439.0 (338.8–577.0) in 2021. The age-standardized DALYs rate also did not significantly shift, maintaining rates of approximately 83.3 (68.9–98.4) in 2019 and 83.4 (68.7–99.8) in 2021. The age-standardized death rate remained unchanged, at 3.9 (3.2–4.6) in 2019 and 3.9 (3.2–4.5) in 2021 (Table S2).

At the country level, most EMR nations had regional trends, with nonsignificant changes in AFF metrics. For example, Iran and Oman reported slight reductions in age-standardized DALYs, with Iran showing a 3.0% decrease (95% UI − 5.9 to − 0.1) and Oman a 2.6% decline (− 8.2 to 3.9). Conversely, Iraq and Palestine experienced small grow in age-standardized DALYs, with Iraq reporting a 7.1% elevation (− 1.7 to 14.8) and Palestine reporting a 2.3% rise (− 2.4 to 7.5). However, these changes were not statistically significant. The United Arab Emirates was an exception, showing a significant reduction in the age-standardized rates of DALYs (− 24.5%, − 33.8 to − 16.6) and deaths (− 35.1%, − 45.9 to − 24.8) (Table S2).

### Country-specific burden of AFF

**Fig. 2:**
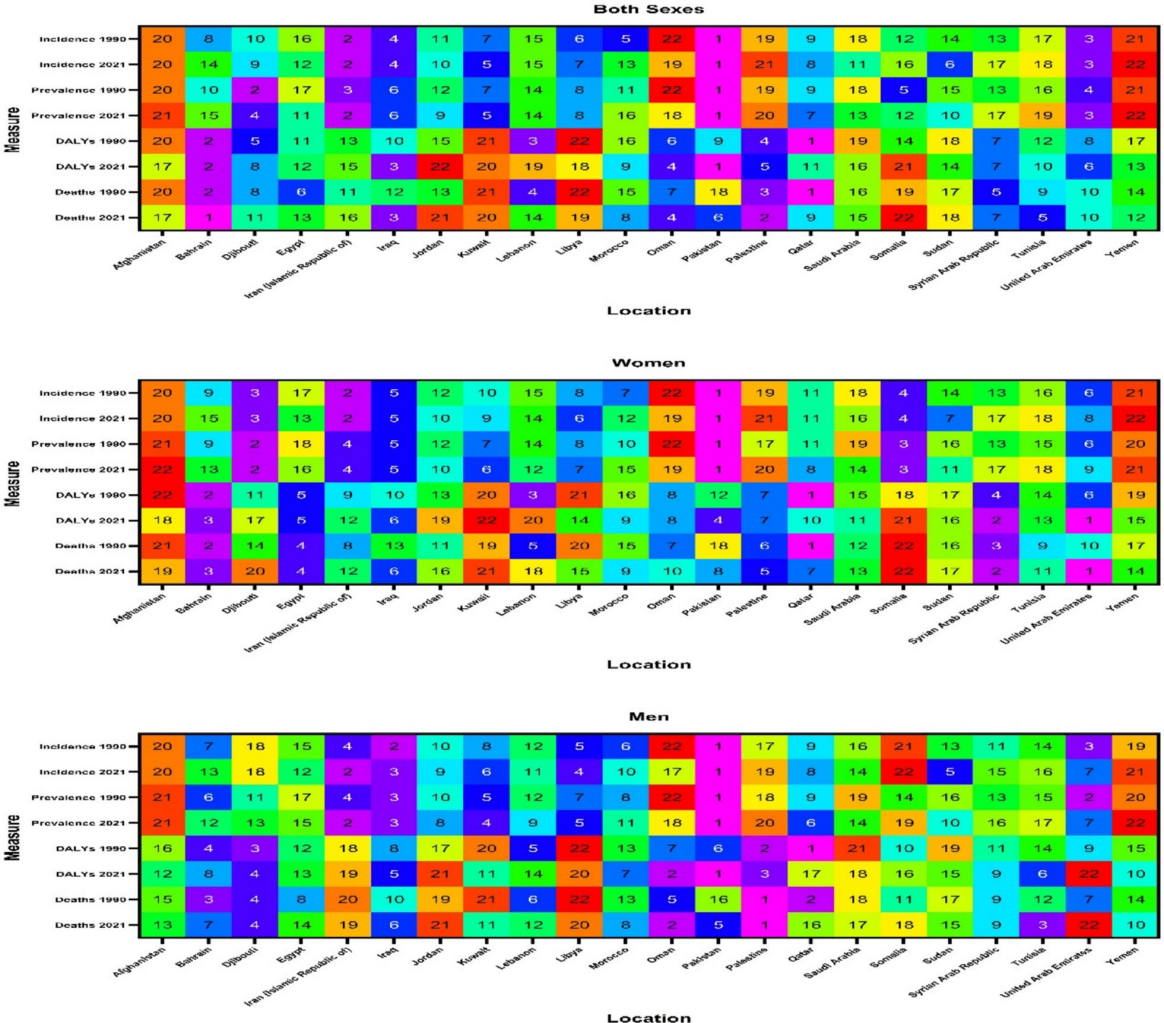
Ranking of the age-standardized rate of incidence, prevalence, disability-adjusted life years (DALYs), and deaths of atrial fibrillation and flutter in 1990 and 2021 in the Eastern Mediterranean Region

Across the EMR, the burden of AFF varied significantly by country. Oman reported the highest surge in age-standardized incidence rates 18.8% (95% UI 12.6–25.3), with nearly similar grow in both women 17.3% (10.2–25.3) and men 18.5% (10.1–27.2). In contrast, Tunisia showed minimal growth (4.7% increase). In terms of age-standardized prevalence rate, Oman had a 24.7% elevation, followed by Saudi Arabia (18.5%) and Egypt (17.7%), while Somalia had a minimal change (4.7% increase). With respect to the age-standardized DALY rates, Pakistan experienced the greatest grow (27.6%), whereas Qatar showed a decrease (− 32.5%). The age-standardized deaths rates varied widely, with Pakistan showing the highest surge in age-standardized death rates (63.6%) and Iraq following a 49.1% increase. Qatar demonstrated the most substantial improvement, with age-standardized death rates decreasing by 44.9% overall (Fig. 2 and Table S3).

In the EMR in 2021, Pakistan consistently presented the highest age-standardized rates of incidence (53.8 [95% UI 40.3–71.6]) and prevalence (558.6 [429.5–735.7]), while Yemen recorded the lowest the age-standardized rates of incidence (32.9 [24.2–44.1]) and prevalence (337.2 [255.8–443.6]). Regarding the age-standardized DALY rate, Pakistan had the highest rate (101.5 [79.7–131.7]), whereas Jordan had the lowest age-standardized DALY rate (63.1 [49.4–78.3]). In terms of age-standardized death rates, Bahrain reported the highest age-standardized death rate (5.7 [2.6–7.9]), while Somalia had the lowest age-standardized death rate (2.2 [1.0 to 4.0]) (Fig. 2 and Table S3).

### Burden of AFF by age and sex

**Fig. 3:**
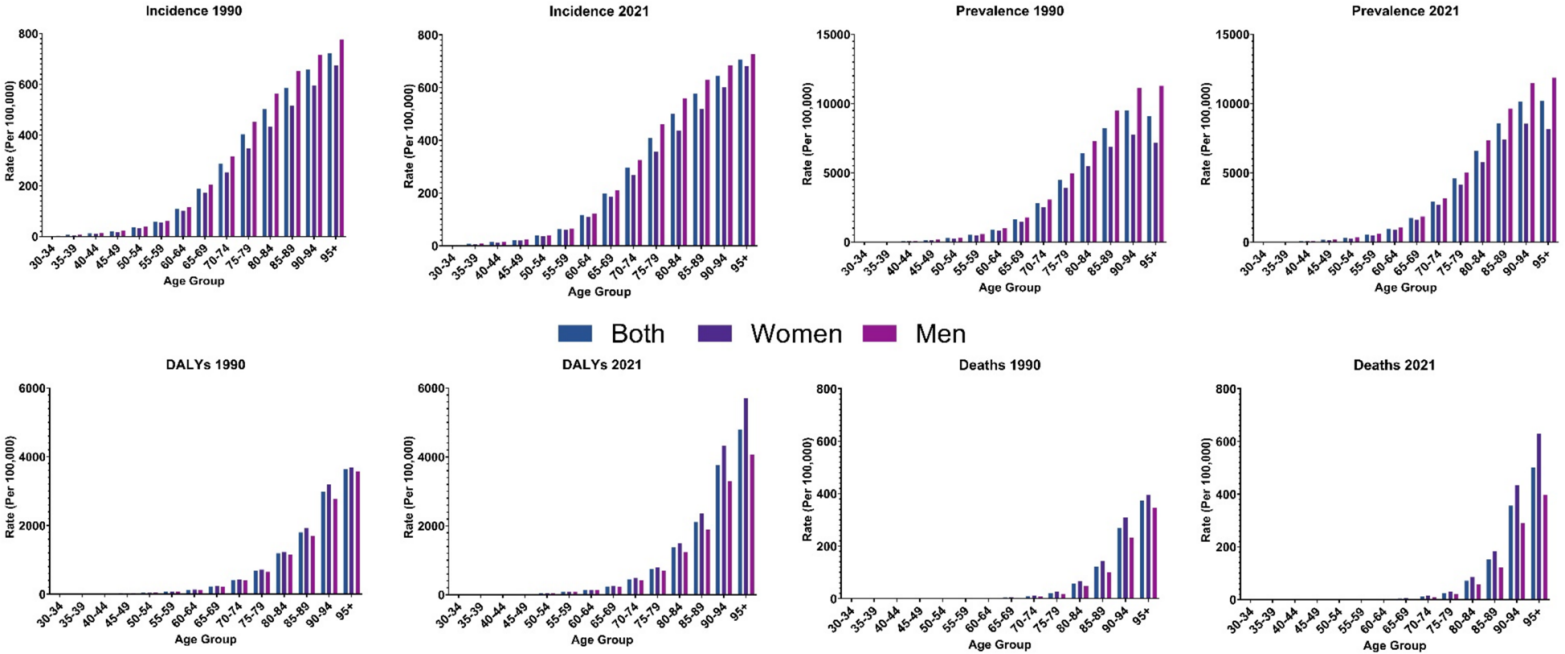
Rate of incidence, prevalence, disability-adjusted life years (DALYs), and deaths of atrial fibrillation and flutter in the Eastern Mediterranean Region in 1990 and 2021, by sex and age

Age-specific analysis revealed that the AFF burden elevated substantially with age in 2021, with incidence rates rising from 2.3 per 100,000 in the 30–34 age group to 706.7 in those aged 95 + years. Men had higher incidence rates across most age groups, particularly middle-aged individuals. The prevalence followed similar patterns in 2021, growing from 4.0 (30–34 age group) to 10,204.5 (95 + age group), with men aged 95 + showing notably higher rates than women (11,855.7 vs 8,153.3). DALYs and deaths demonstrated marked age-related surges and sex-specific variations. DALY rates increased from 1.4 (30–34 age group) to 4,801.2 (95 + age group) in 2021. Despite a lower overall disease frequency, women had higher DALYs (5,704.1 vs 4,074.3) and death (629.0 vs 398.2) rates in the 95 + age group (Fig. 3 and Table S4).

### Sociodemographic index (SDI) and its association with AFF burden

**Fig. 4:**
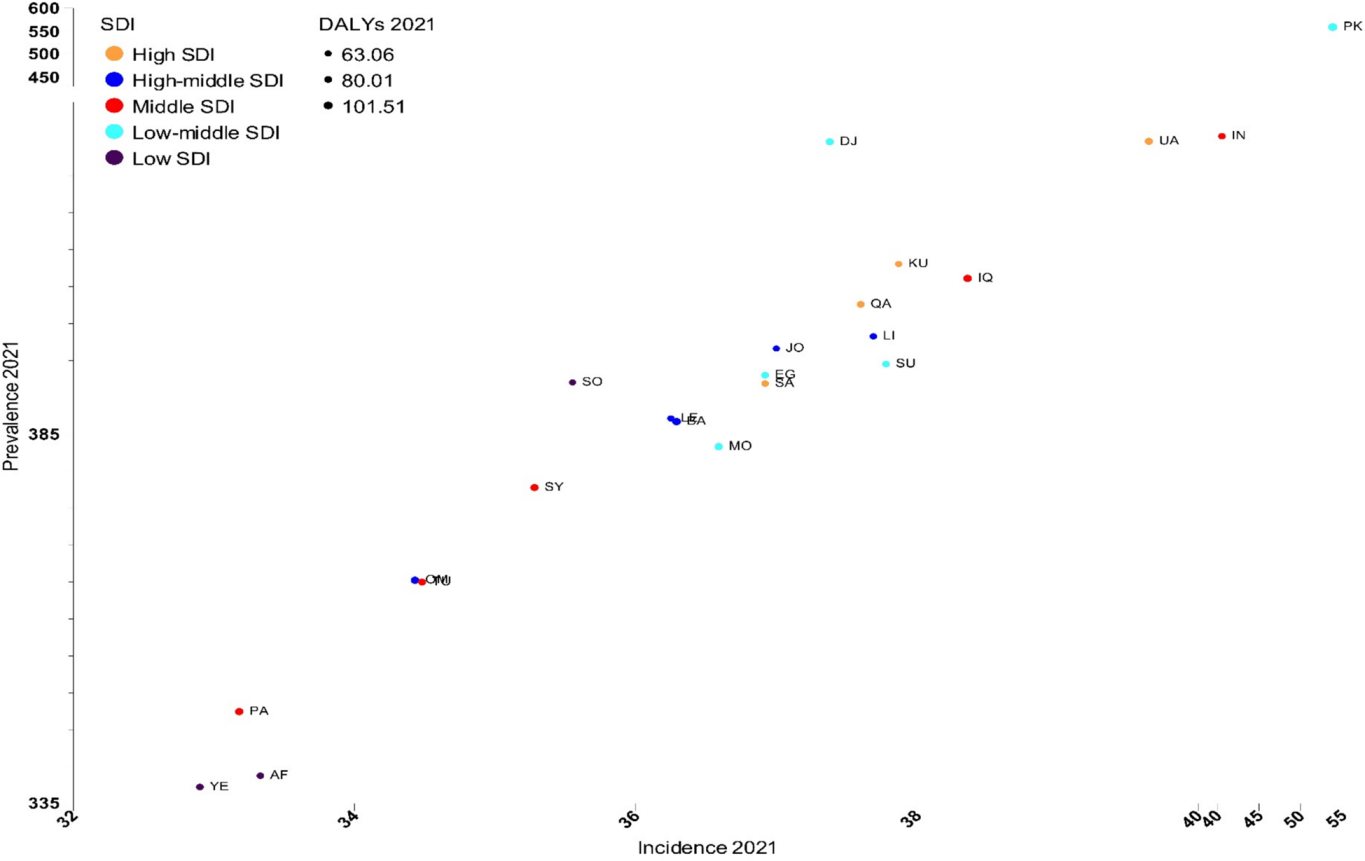
Age-standardized rate of incidence, prevalence, and disability-adjusted life years (DALYs) of atrial fibrillation and flutter in the countries of the Eastern Mediterranean Region in 2021, by sociodemographic index (SDI) quintiles among both sexes

**Fig. 5:**
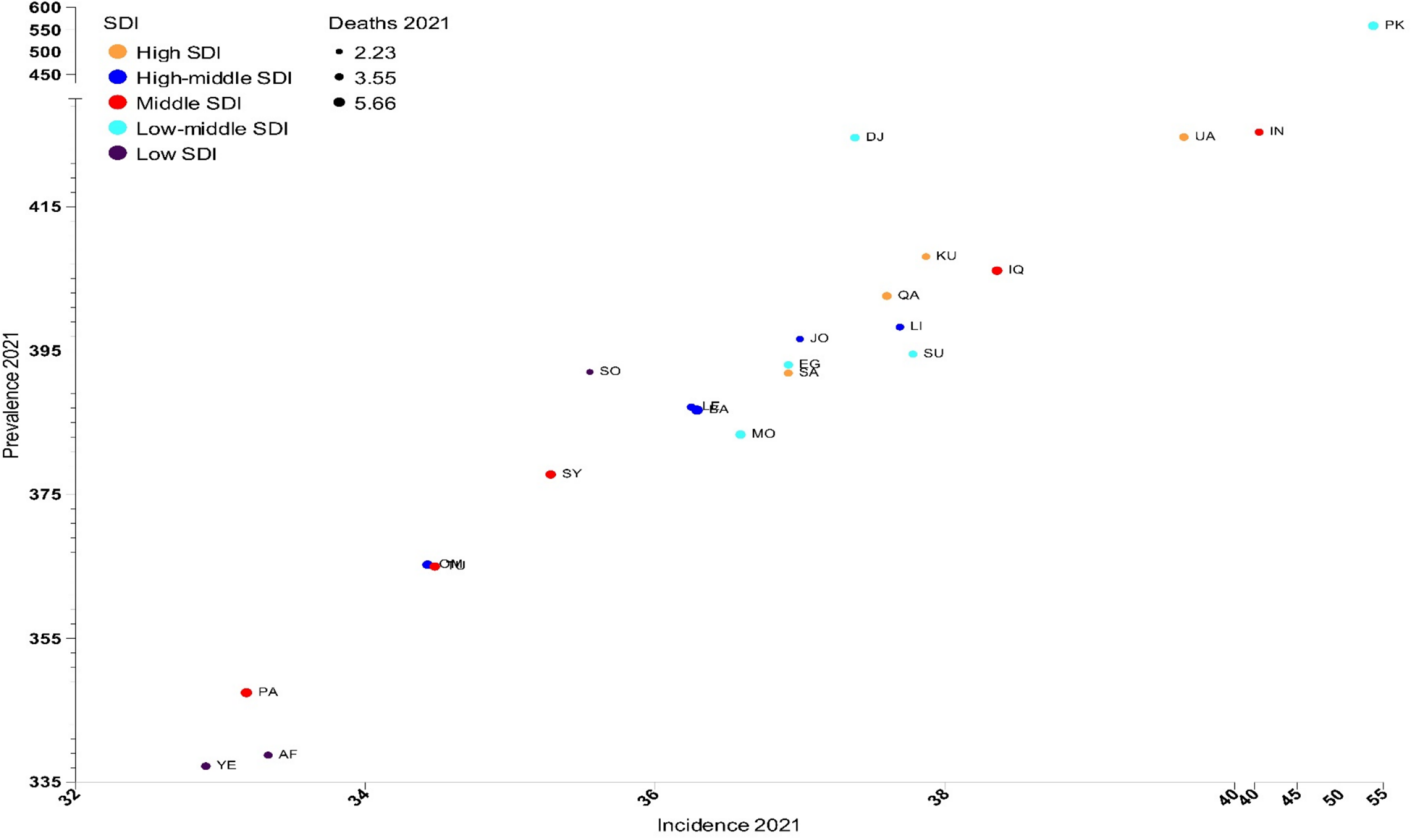
Age-standardized rate of incidence, prevalence, and deaths of atrial fibrillation and flutter in the countries of the Eastern Mediterranean Region in 2021, by sociodemographic index (SDI) quintiles among both sexes

High-SDI countries (United Arab Emirates, Kuwait, Qatar, and Saudi Arabia) demonstrated elevated age-standardized prevalence and incidence rates, with the age-standardized prevalence rate ranging from 391.9 (Saudi Arabia) to 424.7 (United Arab Emirates) and the age-standardized incidence rate ranging from 36.9 (Saudi Arabia) to 39.7 (United Arab Emirates), while maintaining relatively lower age-standardized death rates (3.0–4.1). Notably, Pakistan, despite its low-middle SDI classification, presented the highest burden across multiple metrics, including age-standardized rates for incidence (53.8), prevalence (558.6), and DALYs (101.5). Low-SDI countries (Yemen, Afghanistan, and Somalia) consistently demonstrated lower age-standardized prevalence rates (Yemen: 337.2, Afghanistan: 338.8, Somalia: 392.0), while middle-SDI countries such as Iran showed intermediate burden levels, with relatively high age-standardized incidence rates (40.6) but moderate age-standardized DALYs (72.4) (Figs. 4, 5, and Table S5).

### Attributable risk factors for AFF burden in the EMR

**Fig. 6:**
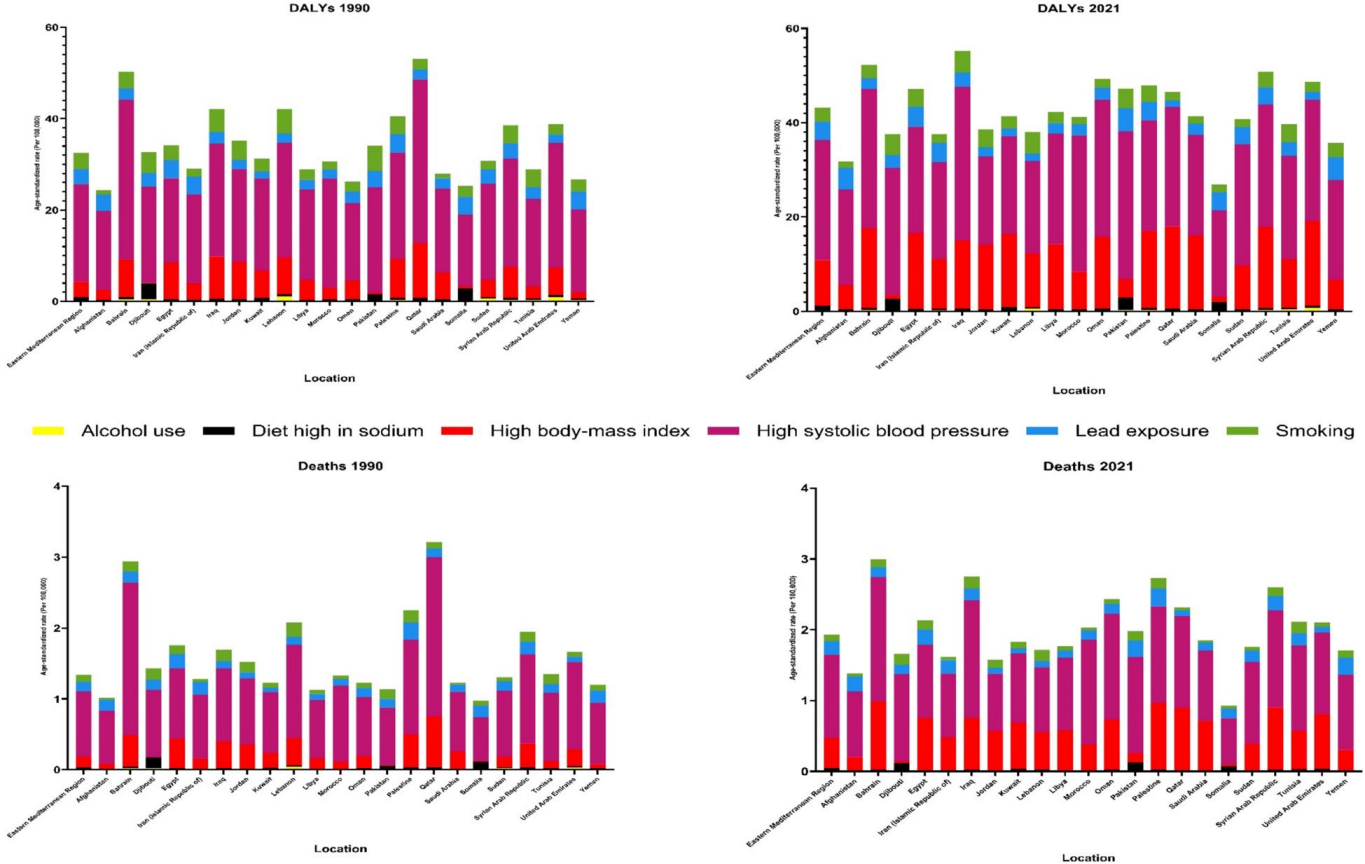
Age-standardized rate of disability-adjusted life years (DALYs) and deaths of atrial fibrillation and flutter attributable to risk factors among both sexes in 1990 and 2021 in the Eastern Mediterranean Region

The burden attributable to all risk factors showed substantial growth, with all-ages DALYs rising by 212.6% (95% UI 168.3–281.2) and age-standardized DALY rates growing by 26.2% (95% UI 7.4–55.7) from 1990 to 2021. HSBP was the predominant contributor, with the highest age-standardized DALY rate of 25.5 (8.8–42.0) in 2021, followed by HBMI at 9.7 (4.2–16.2). HBMI demonstrated the most significant elevation among risk factors, with a 182.6% (130.8–242.3) increase in age-standardized DALY rates. While smoking-attributable age-standardized DALY rates decreased by 14.2%, other modifiable risk factors showed rising trends: HSBP (19.4%), HBMI (182.6%), and lead exposure (17.1%) (Table 2, Fig. 6, and Fig. S3).

**Table 2:** All‑ages number and age‑standardized rate of disability-adjusted life years (DALYs) and deaths of atrial fibrillation and flutter attributable to risk factors by sex in 1990 and 2021 and overall percent change over 1990–2021 in the Eastern Mediterranean Region

Risk factor	Measure	Age, Metric	Year						% Change (1990– 2021)		
			1990			2021					
			Both	Women	Men	Both	Women	Men	Both	Women	Men
All risk factors	DALYs (Disability-Adjusted Life Years)	Age-standardized	25.7 (11.9–40.5)	27.5 (12.1–45.1)	24.2 (11.8–38)	32.5 (17.1–48.5)	34.9 (18.3–51.5)	30.2 (16–44.9)	26.2 (7.4–55.7)	27.2 (− 0.8 to 58.8)	24.9 (7.8–56.2)
		All ages	35,775.3 (17,404.7–56,049.2)	17,506.3 (7910.5–28,332.5)	18,269 (9071.5–28,313.9)	111,844.2 (59,019.1–165,701.7)	56,239.6 (29,097.8–83,137.3)	55,604.6 (29,808.8–82,448.1)	212.6 (168.3–281.2)	221.3 (153.9–301.5)	204.4 (164.2–277.7)
	Deaths	Age-standardized	1.1 (0.5–1.7)	1.3 (0.6–2.3)	0.9 (0.3–1.4)	1.5 (0.8–2.2)	1.8 (0.9–2.6)	1.2 (0.6–1.8)	36.1 (4.1–85.4)	37.3 (− 3.5 to 85.1)	34.3 (4.5–107.4)
		All ages	1162.3 (503.4–1880.8)	667.8 (290.4–1168.4)	494.5 (196–801.6)	3930.1 (2064–5790.7)	2289.1 (1143.4–3411.5)	1641 (888.3–2463.1)	238.1 (158.3–363)	242.8 (140.8–361.2)	231.9 (157.2–414)
Alcohol use	DALYs (Disability-Adjusted Life Years)	Age-standardized	0.1 (0.1–0.2)	0 (0–0)	0.2 (0.1–0.3)	0.2 (0.1–0.3)	0 (0–0)	0.3 (0.2–0.5)	28.6 (− 3.7 to 70.9)	28.4 (− 10.8 to 82.3)	30.6 (− 2.8 to 77.5)
		All ages	220 (124.4–324.8)	18.1 (10.3–26.7)	201.9 (112.3–303.1)	717.6 (444.6–1050.2)	60.2 (36.5–90.5)	657.4 (405.6–969)	226.2 (148–328.7)	232 (139.7–361.7)	225.6 (147.1–334.5)
	Deaths	Age-standardized	0 (0–0)	0 (0–0)	0 (0–0)	0 (0–0)	0 (0–0)	0 (0–0)	33.1 (− 12.6 to 101)	29.2 (− 20.6 to 105.5)	33.5 (− 15.6 to 108)
		All ages	5.1 (2.7–7.8)	0.6 (0.3–0.9)	4.5 (2.3–7.2)	16.2 (9.7–23.7)	1.9 (1.1–2.9)	14.4 (8.5–21.5)	220.4 (114.3–384.1)	222 (103.7–396.2)	220.2 (106.7–400.1)
Diet high in sodium	DALYs (Disability-Adjusted Life Years)	Age-standardized	0.8 (0–3.6)	0.6 (0–2.9)	0.9 (0–4.1)	1 (0–4.3)	0.9 (0–3.9)	1.1 (0–4.9)	30 (6.4–456.5)	43.6 (5.8–527.2)	22.8 (− 6.7 to 526.9)
		All ages	1085.4 (4.8–5058.1)	391.7 (1.5–1911.3)	693.7 (3–3088.7)	3486.3 (35.8–14,769.1)	1408.8 (10.4–6354.2)	2077.5 (20.1–8896.1)	221.2 (165.3–1241.2)	259.7 (166–1411.8)	199.5 (128.7–1453.3)
	Deaths	Age-standardized	0 (0–0.1)	0 (0–0.1)	0 (0–0.1)	0 (0–0.2)	0 (0–0.2)	0 (0–0.2)	48.4 (7.1–579.1)	63.1 (9.2–821.8)	37.4 (− 11 to 730.7)
		All ages	31.8 (0.1–136)	13.2 (0–69.1)	18.6 (0.1–81.6)	116.1 (1.1–513.2)	53.2 (0.3–255.3)	62.9 (0.5–261.5)	265 (171.8–1557.4)	301.2 (167.6–1910.2)	239.1 (125.3–1712)
High body mass index	DALYs (Disability-Adjusted Life Years)	Age-standardized	3.4 (1.3–5.9)	5 (2–8.8)	2 (0.7–3.5)	9.7 (4.2–16.2)	11.9 (5.3–20)	7.6 (3–12.7)	182.6 (130.8–242.3)	136.2 (82.6–194.5)	284.4 (204.7–400.6)
		All ages	5000.7 (1925.8–8606.6)	3454.4 (1364.1–6011.3)	1546.4 (589.8–2755.9)	34,355.5 (15,119.6–57,930.1)	20,151.1 (8987.3–33,817.7)	14,204.4 (5855.7–24,318.8)	587 (468.9–715.4)	483.4 (360.7–615.9)	818.6 (642.5–1066.7)
	Deaths	Age-standardized	0.2 (0.1–0.3)	0.2 (0.1–0.4)	0.1 (0–0.1)	0.4 (0.2–0.7)	0.6 (0.3–1)	0.3 (0.1–0.5)	184.4 (107.8–281.8)	148.3 (72.1–247.4)	288.7 (172.6–502)
		All ages	167.9 (66.6–295.1)	125.3 (51.5–220.1)	42.6 (14.9–78.7)	1164 (522.3–1911.9)	761.3 (339–1263.1)	402.7 (176–665.3)	593.4 (410.5–815.9)	507.8 (330–733.7)	845.2 (574.9–1327.9)
High systolic blood pressure	DALYs (Disability-Adjusted Life Years)	Age-standardized	21.4 (7.4–35.8)	24.4 (8.9–41.6)	18.7 (6–32.8)	25.5 (8.8–42)	28.4 (10.1–46.2)	22.8 (7.3–38.3)	19.4 (1.2–39.8)	16.7 (− 7.1 to 39.9)	22.1 (5.4–50.4)
		All ages	28,975.8 (9929.6–49,161.8)	15,317.5 (5571.1–26,097)	13,658.2 (4382.1–24,035.7)	85,903.3 (29,483.1–141,398.8)	45,133 (16,049.2–72,676.6)	40,770.3 (13,227.5–68,497.8)	196.5 (154.3–242.4)	194.6 (138.4–250)	198.5 (157.2–263.1)
	Deaths	Age-standardized	0.9 (0.3–1.6)	1.2 (0.4–2.1)	0.7 (0.2–1.3)	1.2 (0.4–1.9)	1.5 (0.5–2.4)	0.9 (0.3–1.5)	27.6 (− 2 to 67.1)	26.5 (− 9.3 to 65.2)	29.5 (1.2 to 96)
		All ages	983.7 (325–1722.5)	593.3 (217.1–1094.7)	390.4 (108.7–702.1)	3137.9 (1133.8–5082.2)	1877.5 (681.6–3037.2)	1260.4 (425.9–2127.1)	219 (143.1–316.3)	216.4 (127.2–313.5)	222.8 (151.5–392)
Lead exposure	DALYs (Disability-Adjusted Life Years)	Age-standardized	3.3 (− 0.5 to 8.2)	2.9 (− 0.4 to 7.7)	3.6 (− 0.5 to 9.1)	3.8 (− 0.5 to 9.6)	3.7 (− 0.5 to 9.1)	4 (− 0.5 to 10.1)	17.1 (1.8 to 38.8)	25.5 (− 0.2 to 50.7)	11.3 (− 2.6 to 35.8)
		All ages	4522.4 (− 627.1 to 11,350.6)	1874.5 (− 275.2 to 4899.1)	2647.9 (− 373.2 to 6636.7)	12,338.2 (− 1641.6 to 30,821.6)	5555.7 (− 735.5 to 13,766.1)	6782.5 (− 906.1 to 17,008.9)	172.8 (139.8–219.7)	196.4 (138.6–251.7)	156.1 (125.3–207.9)
	Deaths	Age-standardized	0.1 (0–0.4)	0.1 (0–0.4)	0.1 (0–0.4)	0.2 (0–0.5)	0.2 (0–0.5)	0.2 (0–0.4)	36.7 (7.8–82.9)	45.9 (5.6–88.2)	27.9 (0.1–95.3)
		All ages	145.6 (− 22.5 to 382.4)	70.8 (− 12.1 to 188.5)	74.8 (− 11.3 to 208.3)	477.6 (− 70.7 to 1170.5)	247.5 (− 35.7 to 590.3)	230.1 (− 35 to 581.9)	227.9 (157.4–340.4)	249.3 (153.3–351.9)	207.6 (140.7–369.3)
Smoking	DALYs (Disability-Adjusted Life Years)	Age-standardized	3.6 (2–5.5)	0.8 (0.4–1.3)	6 (3.3–9.3)	3.1 (1.7–4.6)	0.8 (0.4–1.2)	5.2 (2.9–7.7)	− 14.2 (− 25.8 to 2.1)	− 7.8 (− 30.2 to 17.4)	− 13.6 (− 26.2 to 4.2)
		All ages	5710 (3200–8733.9)	650 (349.1–1038.3)	5060 (2821.3–7865.4)	12,536 (7088.7–18,612.9)	1465 (814.7–2316.9)	11,071 (6321.6–16,510.9)	119.5 (91.3–157.9)	125.4 (74.1–185)	118.8 (89.3–160.4)
	Deaths	Age-standardized	0.1 (0.1–0.2)	0 (0–0)	0.2 (0.1–0.3)	0.1 (0.1–0.1)	0 (0–0)	0.2 (0.1–0.2)	− 5.7 (− 28.4 to 37.6)	10.1 (− 25.6 to 57.8)	− 7.9 (− 31.3 to 39.4)
		All ages	126.6 (65.2–194)	17.4 (9–28.1)	109.3 (53.1–172)	295.6 (173.1–430.7)	44.9 (23.9–68.5)	250.7 (148.8–365.5)	133.4 (80.5–239)	158.5 (72.9–263.6)	129.5 (74.8–248.2)

For all combined risk factors, women had higher age-standardized DALY rates than men did, growing from 27.5 (95% UI 12.1–45.1) in 1990 to 34.9 (18.3–51.5) among women and increasing from 24.2 (11.8–38.0) to 30.2 (16.0–44.9) among men. The age-standardized death rates were also higher in women, with rates ranging from 1.3 (0.6–2.3) to 1.8 (0.9–2.6), and those in men elevated from 0.9 (0.3–1.4) to 1.2 (0.6–1.8). All risk factors except HBMI (women: 5.0 [2.0–8.8] in 1990 to 11.9 [5.3–20.0] in 2021, men: 2.0 [0.7–3.5] in 1990 to 7.6 [3.0 to 12.7] in 2021) and HSBP (women: 24.4 [8.9 to 41.6] in 1990 to 28.4 [10.1–46.2] in 2021, men: 18.7 [6.0–32.8] in 1990 to 22.8 [7.3–38.3] in 2021), men had higher age-standardized DALY rates than women in both 1990 and 2021. Also, HBMI showed a 284.4% (204.7–400.6) increase in the age-standardized DALY rates by 2021 (1990: 2.0 [0.7–3.5]; 2021: 7.6 [3.0–12.7]) among men, far exceeding the 136.2% (82.6–194.5) rise in women (Figs. S1, S2, and Table S6).

HSBP was the predominant risk factor for age-standardized rates of DALYs and deaths across all the countries of the EMR in 1990 and 2021 among both sexes (Figs. S3, S4, and S5). Among the countries in the EMR, Somalia had the lowest rankings for age-standardized rates of DALYs and deaths from HSBP among both sexes (in 1990, with the age-standardized DALY rate of 15.7 [95% UI 4.5–30.3], and in 2021, with the age-standardized DALY rate of 18.3 [5.6–34.6]). Conversely, Qatar had the highest ranking, with the age-standardized DALY rate of 35.8 (11.3–63.1) in 1990, and Iraq had the highest ranking, with the age-standardized DALY rate of 32.6 (11.0–54.9) in 2021 (Figs. S3, S4, S5, S6, and Table S6).

Qatar had the highest burden of AFF attributed to HSBP in 1990 in terms of age-standardized DALY rates (women: 43.9 [95% UI 12.7–79.7] and men: 27.9 [8.4–53.9]) and age-standardized death rates (women: 2.9 [95% UI 0.8–5.4] and men: 1.5 [0.4–3.3]). In 2021, the highest age-standardized rates of DALYs and deaths among women were observed in the United Arab Emirates at 72.8 (23.8–124.5) and 4.9 (1.6–8.5), respectively. The highest age-standardized rates of DALYs and deaths among men were observed in Iraq (30.9 [10.1–53.0]) and Palestine (1.5 [0.5–3.0]), respectively (Figs. S7 and S8).

## Discussion

This study provided a comprehensive assessment of the burden of AFF in the EMR from 1990 to 2021. Using secondary analysis from the GBD study 2021, we highlighted trends in incidence, prevalence, DALYs, and death. The findings indicated a slight increase in the age-standardized incidence rate, with a greater increase observed in women than in men. Similarly, the age-standardized prevalence rate increased, with women experiencing a more significant increase than men. The disease burden, measured by DALYs and death rates, also rose significantly. Sex-specific differences were evident, as women consistently had greater increases in incidence, prevalence, DALYs, and death rates and higher values of age-standardized DALYs and death rates, despite men maintaining higher age-standardized incidence and prevalence rates throughout the study period. Analysis by age also revealed a significant increase in the AFF burden as age increased.

The burden of AFF also varied significantly across countries within the EMR, with Oman, Saudi Arabia, and Egypt showing the highest increases in prevalence, whereas Pakistan demonstrated minimal changes. Pakistan experienced the greatest increase in DALYs, whereas Qatar experienced an overall decline. The death patterns also varied, with Pakistan having the highest increase and Qatar exhibiting the most significant decrease. Risk factor analysis identified HSBP and HBMI as the leading contributors to AFF burden despite alcohol use having the lowest contribution. HBMI also had the greatest increase among the risk factors within the study period. Women had higher age-standardized rates for HBMI and HSBP, highlighting the need for targeted prevention efforts. The findings suggest substantial disparities in AFF burden across different SDI levels, with high-SDI countries exhibiting elevated prevalence rates but lower death rates. The increasing burden of AFF concerning age-standardized incidence and prevalence in the EMR from 1990 to 2021 contrasts with the global trends noted in earlier studies, which indicated a decline in age-standardized incidence and prevalence [13]. However, age-standardized DALYs and deaths corresponded with global trends, showing an increasing trend throughout the study period. Consistent with our study, the overall incidence, prevalence, DALY, and death rates have significantly increased in global burden studies due to population growth, aging, and enhanced diagnostic methods. In previous studies, the increasing burden of AFF was explained by the increase in chronic cardiovascular risk factors, which contribute to atrial remodeling and disease progression [14–16].

In the EMR, women experienced a more substantial increase in burdens of AFF than men did; however, in global studies, women experienced a decrease in incidence, prevalence, and DALYs [13]. Consistent with the global burden analysis of AFF, in the EMR, men had a higher absolute rate of incidence and prevalence, whereas women had higher rates of DALYs and death [13, 17, 18]. These trends were consistent across the region for the previous three decades [19]. In another study on the Middle East and North Africa populations, women's obesity rates exceeded men's rates by more than 10 percentage points on average. Globally, these rates were approximately four percent [10, 20]. Recent studies also revealed a worse prognosis of AFF in women, including more severe symptoms and a greater risk of stroke, than in men [21, 22].

We found a greater burden in the elderly population. Aging contributes to atrial structural remodeling, fibrosis, oxidative stress, and inflammation, all of which promote arrhythmogenesis. Recent studies also suggest that mechanisms such as mitochondrial dysfunction, telomere attrition, cellular senescence, impaired autophagy, and gut dysbiosis contribute to the increased susceptibility of older adults to AFF. However, there is an increased risk of thromboembolic events in elderly patients with AFF. These findings underscore the need for age-specific prevention and management strategies, particularly in regions experiencing rapid demographic aging [23–25].

The relationship between SDI levels and the burden of AFF reveals distinct patterns across the EMR. High-SDI countries demonstrate elevated age-standardized incidence and prevalence rates while maintaining relatively lower death rates. However, global studies have indicated that high-SDI countries experienced higher incidence, prevalence, DALY, and death rates in 2021 [19]. The findings suggest that while high-SDI countries in the EMR tend to report higher prevalence rates of AFF, their lower mortality rates can be attributed to several factors related to healthcare access and quality. These countries typically benefit from more robust healthcare systems with better infrastructure, allowing for earlier detection and timely management of AFF through routine screening, advanced diagnostic tools, and specialized cardiovascular care. Availability and widespread use of effective treatments including anticoagulation therapy, rate and rhythm control medications, and catheter ablation contribute to reducing stroke and other fatal complications associated with AFF. Moreover, risk factor management addressing hypertension, obesity, and diabetes, supported by targeted public health initiatives and improved control of comorbidities, further mitigates disease progression and mortality. The improved continuity of care enabled by multidisciplinary teams and health insurance coverage enhances adherence to guideline-based therapies. In contrast, lower-SDI countries face resource limitations leading to underdiagnosis, delayed treatment, and less effective management, which likely contribute to higher AFF-related mortality despite a lower reported prevalence. Understanding these disparities underscores the need to strengthen healthcare infrastructure and access in lower-SDI contexts to improve AFF outcomes across the region. Socioeconomic status impacts AFF incidence, management, and outcomes. Lower-income individuals consistently show higher AFF incidence rates and worse clinical outcomes, including increased stroke risk, hospitalizations, and mortality [26, 27]. These disparities are particularly pronounced in the EMR due to the region's unique socio-political and economic challenges. The EMR faces substantial healthcare access inequities that impact AFF management. Healthcare infrastructure is predominantly concentrated in urban areas, creating significant barriers for rural populations [28, 29]. This geographic maldistribution is especially problematic given that rural communities often have higher cardiovascular risk factor burdens. Political instability and conflict zones create unique barriers to cardiovascular care disruptions occur in Syria, Iraq, and Yemen, where healthcare system fragmentation impacts chronic disease management [30, 31]. Disruptions in health systems lead to higher rates of readmission and complications. Reinforcing AFF care in conflict-affected areas through better medication access and coordinated follow-up is key to reducing adverse outcomes [32]. There are stark disparities between resource-rich Gulf states and other EMR countries. Gulf states show higher AFF prevalence among older, more affluent populations with better access to specialized cardiac care, while lower-income EMR countries face challenges with younger-onset AFF complicated by higher rates of rheumatic valvular disease [8, 33, 34]. Women in the EMR face additional barriers to AFF care, particularly in conservative societies where healthcare access may be restricted. The Gulf SAFE registry revealed sex differences in treatment approaches, with women receiving different anticoagulation strategies despite similar risk profiles [34]. Healthcare fragmentation due to multiple providers, conflicting donor priorities, and political divisions creates coordination challenges. This fragmentation particularly impacts chronic disease management, where continuous care is essential for optimal AFF outcomes [30].

Identifying attributable risk factors for AFF is essential for planning the best prevention strategies in the EMR. HSBP and HBMI were the leading risk factors associated with AFF burden. HSBP contributes to both disease onset and progression. The hemodynamic and neurohormonal effects of hypertension lead to diffuse electrostructural remodeling of the left atrium, promoting arrhythmogenesis [35]. In addition to its role in AFF development, uncontrolled hypertension significantly increases the risk of stroke in patients with AFF, leading to a greater incidence of thromboembolic events [36]. Given the strong association between hypertension and AFF-related mortality, effective blood pressure control is essential in both prevention and clinical management strategies. Management of hypertension through appropriate interventions may help mitigate atrial remodeling, lower stroke risk, and decrease the burden of AFF [35–37].

HBMI also plays a significant role in the burden and progression of AFF through both direct and indirect mechanisms. Obesity-driven ventricular adaptation, diastolic dysfunction, and epicardial fat accumulation promote arrhythmogenesis by atrial structural and electrical remodeling [38]. Given that the prevalence of overweight and obesity in the EMR is significantly higher than the global prevalence (46% and 34.5%, respectively), the impact of obesity on the AFF burden in this region is particularly concerning [39]. In addition to the increased risk of AFF development, a higher BMI significantly increases AFF-related complications, emphasizing the need for targeted weight management strategies to mitigate the burden of AFF and associated comorbidities, especially in countries with a high prevalence of obesity [40].

The interplay between HSBP, HBMI, diabetes mellitus, and physical inactivity significantly contributes to the risk of developing AFF [41, 42]. Observational studies have established that diabetes mellitus is associated with a significantly increased risk of AFF, which is further exacerbated when coupled with hypertension and obesity [43]. Furthermore, physical inactivity compounds these issues by worsening metabolic profiles and contributing to HSBP and HBMI [44]. Approximately 30% of patients with AFF also had diabetes mellitus, highlighting the substantial overlap between these conditions [45]. This clustering of risk factors necessitates a comprehensive approach to patient management, emphasizing the need for tailored interventions to address the unique challenges posed by these interconnected conditions within the EMR, ultimately aiming to improve clinical outcomes for patients with AFF.

COVID-19 is associated with systemic inflammation, hypoxia, autonomic activation, and myocardial injury, all of which can precipitate new-onset AFF during hospitalization [46–48]. Observational data showed in-hospital AFF incidence around 10% among COVID-19 admissions, with 4% new-onset in those without prior arrhythmia; AFF was associated with substantially higher mortality in COVID-19 cohorts [48–50]. Compared with matched non-COVID or pre-pandemic patients, COVID-19 positivity was linked to higher odds of AFF, suggesting infection-related risk beyond baseline comorbidity burden [51, 52]. Emerging pooled and meta-analytic evidence indicates an increased risk of incident AFF after COVID-19 recovery at the population level, underscoring possible longer-term sequelae affecting post-2020 prevalence and DALYs [53, 54]. The pandemic disrupted cardiovascular care with deferred presentations, reduced hospitalizations, and treatment delays, which likely worsened outcomes and may have altered AFF detection patterns and coding during 2020–2021 [55, 56]. These disruptions could bias GBD inputs via missed diagnoses alongside over-representation of severe, hospitalized cases, complicating trend interpretation for 2020–2021 [48, 55]. Pre-existing antithrombotic use has been associated with lower odds of COVID-19 death in observational analyses, reinforcing the importance of anticoagulation optimization for patients with AFF during pandemic surges, while recognizing these findings are not causal [57]. COVID-19-related hypercoagulability increases thromboembolic risk, amplifying the stakes of anticoagulation continuity and access for patients with AFF in disrupted health systems [47, 51]. During the COVID-19 pandemic, the burden of AFF in the EMR remained relatively stable. This stability may largely result from incomplete data sources and inadequate reporting in the region. However, the pandemic caused significant healthcare disruptions, including lockdowns and the redirection of medical resources, which delayed the diagnosis and treatment of AFF [58]. Patients faced significant barriers to accessing essential cardiac care during the pandemic, leading to increased morbidity and mortality from untreated arrhythmias. Many avoided hospital visits due to fears of virus exposure, resulting in postponed follow-ups and worsening health conditions. Additionally, lifestyle changes such as reduced physical activity and increased psychological stress intensified risk factors like hypertension and obesity, raising the incidence of AFF. Emerging evidence also links COVID-19 infection to greater risks of cardiac arrhythmias, potentially increasing new AFF cases among vulnerable populations [59]. The long-term consequences are expected to surface in the post-pandemic period, highlighting the need for healthcare policymakers in the EMR to integrate AFF management into public health strategies to address these ongoing challenges.

To translate the study findings into clinical practice, we recommend structured task-shifting to expand AFF detection and management capacity, empowering primary care nurses to manage stable AFF under physician oversight, and introducing dedicated AFF coordinators that enhanced detection and care. Embedding digital health infrastructure into routine care can bridge specialty gaps and standardize follow-up, including telemedicine links between peripheral clinics and cardiology centers, electronic AFF care registries for surveillance and quality monitoring, and mobile applications that support symptom tracking and medication adherence. To address documented knowledge gaps, guideline implementation should include mandatory continuing education with emphasis on pathophysiological classification, multidisciplinary team training to improve cross-specialty communication, and mentorship pathways pairing experienced cardiologists with primary care providers. Collectively, these measures operationalize risk stratification, timely anticoagulation, and structured follow-up within primary care models while maintaining specialist oversight. From a public health policy perspective, priorities include equitable access and system resilience. Targeted coverage expansions for AFF services should comprise subsidized access to essential cardiovascular drugs particularly anticoagulants, free screening in underserved and rural communities, and transportation vouchers to reduce geographic barriers to specialist care. In conflict-affected settings, resilient delivery models are warranted: mobile cardiac units capable of navigating checkpoint delays, telemedicine consultations when movement is restricted, and decentralized community-based treatment programs that reduce reliance on tertiary facilities. Quality improvement should be anchored by national AFF registries, routine reporting of quality indicators (anticoagulation rates, stroke outcomes, guideline adherence), and regular benchmarking to inform accountability and resource allocation.

While this study provides a comprehensive assessment of the burden of AFF in the EMR, several limitations must be acknowledged. Our analysis was based on secondary, aggregated GBD inputs and was therefore inherently ecological. As such, it is susceptible to the ecological fallacy as population-level patterns may not reflect individual-level relationships and country-level aggregates can mask substantial within-country heterogeneity in AFF distribution, risk factor profiles, and access to care across EMR settings. Data quality and completeness also vary across the region, reflecting differences in health system maturity and surveillance capacity; for example, conflict-affected settings face severe constraints that limit case ascertainment and reporting. Furthermore, our reliance on ICD-coded administrative data introduces additional uncertainty due to documented variability in coding practices and upstream physician documentation barriers, including incomplete clinical records, inconsistent diagnostic terminology between clinicians and coders, and time pressures that lead to abbreviated documentation. These limitations can bias burden estimates and differentially affect countries, complicating direct cross-country comparisons. Model-based uncertainties further influence our estimates. Bayesian prior specifications may not align with EMR-specific epidemiology, convergence can be challenged by sparse data in conflict-affected settings, and structural assumptions about disease progression may inadequately capture AFF patterns in younger EMR populations. Although GBD provides 95% UIs, these may underestimate true uncertainty because primary data quality issues are only partially represented, modeling assumption uncertainty is not fully propagated through all analytical stages, and between-model uncertainty (where multiple plausible epidemiological models could fit the available data) is not incorporated. Consequently, our findings should be interpreted as approximations subject to these ecological and modeling constraints; in particular, individual-level inferences should be avoided and small differences especially those within overlapping uncertainty intervals should be interpreted with caution. These limitations regarding data completeness and potential biases must be recognized as they can lead to misinterpretation of findings and may impact public health strategies and resource allocation. Moreover, while this study highlights the role of HSBP and HBMI as main risk factors, the GBD framework does not yet account for certain other important contributors to AFF, such as diabetes, physical inactivity, and hyperthyroidism. The exclusion of diabetes is particularly concerning, given its well-documented association with increased risks for various cardiovascular events, including AFF. Similarly, physical inactivity is linked to obesity and hypertension, both significant precursors to AFF. Additionally, obstructive sleep apnea has been recognized as a substantial risk factor due to its contribution to increased sympathetic activity and arrhythmias. The absence of these risk factors may lead to an underestimation of the true burden of AFF and could bias public health strategies aimed at addressing this condition. However, the mentioned risk factors, as well as hyperthyroidism, were not included because GBD does not currently attribute AFF burden to these factors. Healthcare disparities, underdiagnosis, and limited access to specialized care could lead to an underrepresentation of the actual disease burden in some regions. Future research should focus on improving data collection, integrating a broader range of risk factors, and refining region-specific estimates to increase the accuracy and applicability of findings. We acknowledge that the study relies on GBD-modeled estimates due to sparse, uneven, or inaccessible primary data across several EMR settings, which may lead to under- or overestimation of true AFF burdens. To mitigate this, we triangulated estimates where possible with national reports, hospital discharge statistics, and published registries, and we present 95% UIs to reflect imprecision. We also emphasize that countries with limited surveillance capacity or conflict-related disruptions likely have greater uncertainty, and direct cross-country comparisons should be interpreted cautiously. Our findings should therefore be viewed as the best available region-wide approximation pending expanded investment in standardized surveillance systems, clinical registries, and routine reporting to strengthen primary data in the EMR. Our population-level analyses cannot fully account for unmeasured or incompletely measured factors such as war exposure and displacement, differential healthcare access and health literacy, and region-specific genetic structure that may influence AFF burden and its risk factor relationships. Where data were available, we incorporated covariates and stratified results to reduce confounding; however, residual confounding is likely, particularly in conflict-affected or data-sparse settings. Accordingly, we refrain from individual-level inference and interpret between-country differences with caution, recognizing that observed gradients may partially reflect unmeasured contextual factors and data artifacts in addition to true epidemiologic variation. By design, the study focuses on population metrics and does not resolve clinical subtypes of AFF (e.g., paroxysmal versus persistent), underlying etiologies, treatment patterns (including oral anticoagulation), or downstream outcomes such as stroke and systemic embolism. As a result, translation to bedside management is indirect, oriented toward public health planning, health system prioritization, and regional benchmarking rather than clinical decision-making. We highlight the need for complementary EMR initiatives like prospective clinical registries, EHR-linked cohorts, audits of anticoagulation use and quality, and integrated AFF and stroke surveillance to delineate subtype-specific patterns, treatment uptake, and outcome trajectories that our ecological approach cannot capture. Risk factor attributions follow the GBD comparative risk assessment framework and quantify population attributable fractions under counterfactual exposure scenarios. These estimates should not be interpreted as proof of causality and may not fully adjust for local confounders or effect modifiers. While the framework synthesizes global evidence on exposure–response relationships, its assumptions and data inputs may not perfectly align with EMR contexts, and residual confounding is possible. We therefore describe risk factor contributions as associative and present uncertainty intervals to reflect imprecision, avoiding causal language. Future EMR-focused causal designs, well-phenotyped cohorts, natural experiments, and genetic approaches are needed to establish causal pathways and intervention targets more definitively. Atrial fibrillation can be classified into two main categories: non-valvular atrial fibrillation (NVAF) and valvular atrial fibrillation (VAF). NVAF typically occurs in the absence of significant heart valve disease, while VAF is associated with structural heart changes, particularly from rheumatic heart disease, which is prevalent in several countries within the EMR, notably Pakistan and Egypt. The distinction between NVAF and VAF is vital, as it influences the underlying risk factors, epidemiology, and clinical management of atrial fibrillation. In younger populations, VAF, often due to rheumatic heart disease, can contribute to the overall burden of atrial fibrillation, leading to different treatment approaches and public health strategies compared to NVAF. Understanding these differences is essential for accurately assessing the risks and outcomes associated with atrial fibrillation and tailoring interventions that address the specific needs of affected populations. However, a limitation of our study arises from the reliance on data from the GBD 2021, which does not differentiate between NVAF and VAF. This absence of distinction limits our ability to evaluate the specific burden and impact of VAF, particularly in populations where rheumatic heart disease remains a significant issue. As a result, our findings may not fully capture the nuances of AFF epidemiology within the region, emphasizing the need for more refined data that can distinguish these two forms of atrial fibrillation to guide effective public health policies and clinical practices. Addressing this limitation in future research will be crucial for better understanding the complexity of AFF and improving health outcomes for affected individuals. Moreover, the GBD 2021 dataset does not explore the specific association between rheumatic heart disease and AFF. This absence of data limits our ability to assess how rheumatic heart disease contributes to the prevalence and outcomes of AFF, particularly in populations where rheumatic conditions are prevalent, such as in the EMR. Consequently, insights regarding the burden of VAF and its associated mortality, especially among younger patients, remain underexamined. The lack of this specific analysis detracts from a holistic understanding of the impact of rheumatic heart disease on AFF epidemiology and highlights the need for further research that incorporates this important intersection to inform public health strategies and clinical practices effectively.

In conclusions, the findings highlight sex differences, with women experiencing higher DALYs and death rates despite men having higher incidence and prevalence rates. Among the major modifiable risk factors, HSBP and HBMI emerged as the leading contributors to AFF burden. The data emphasize the need for targeted public health interventions, particularly in hypertension and obesity management, to reduce the burden of AFF. Given the considerable heterogeneity in sociodemographic profiles and healthcare infrastructure across the EMR, public health interventions for AFF must be carefully tailored to the unique needs of each sub-region. Strengthening primary healthcare systems through culturally sensitive, community-based screening and awareness programs can improve early detection and management of AFF-related risk factors, particularly in under-resourced areas. Equitable access to affordable anticoagulation therapies and enhanced provider training on guideline-based care are essential to reduce stroke risk and adverse outcomes. Furthermore, telemedicine and mobile health innovations offer promising solutions to overcome geographic and specialist shortages, expanding care access to rural and underserved populations. Public health messaging should be designed to address literacy, language, and gender disparities by engaging local communities and leaders. Also, integrating AFF management into broader noncommunicable disease strategies, alongside strengthening surveillance systems and healthcare workforce capacity, will be critical for achieving sustainable improvements in cardiovascular health throughout the EMR.

## Supplementary Information

**Additional file 1**.

**Additional file 2**.

**Additional file 3**.

**Additional file 4**.

**Additional file 5**.

**Additional file 6**.

**Additional file 7**.

**Additional file 8**.

**Additional file 9**.

## Data Availability

The data used for these analyses are all publicly available at https://vizhub.healthdata.org/gbd-results/

## Abbreviation

AFF: Atrial fibrillation and flutter
HSBP: High systolic blood pressure
HBMI: High body mass index
EMR: Eastern Mediterranean Region
SDI: Sociodemographic index
GBD: Global Burden of Disease
DALYs: Disability-adjusted life years
ICD: International Classification of Disease
DisMod-MR 2.1: Disease Modeling Meta-Regression version 2.1
CODEm: Cause of Death Ensemble Modeling
SGPR: Spatiotemporal Gaussian process regression
NVAF: Non-valvular atrial fibrillation
VAF: Valvular atrial fibrillation
UIs: Uncertainty intervals
