# Supplementary figures and images for: Atrial fibrillation and flutter in the Eastern Mediterranean: burden, disparities, and risk factor contributions from 1990 to 2021
Source: Egypt Heart J. 2025 Oct 22;77:98. doi: 10.1186/s43044-025-00693-5 (PMC12546237; doi:10.1186/s43044-025-00693-5)

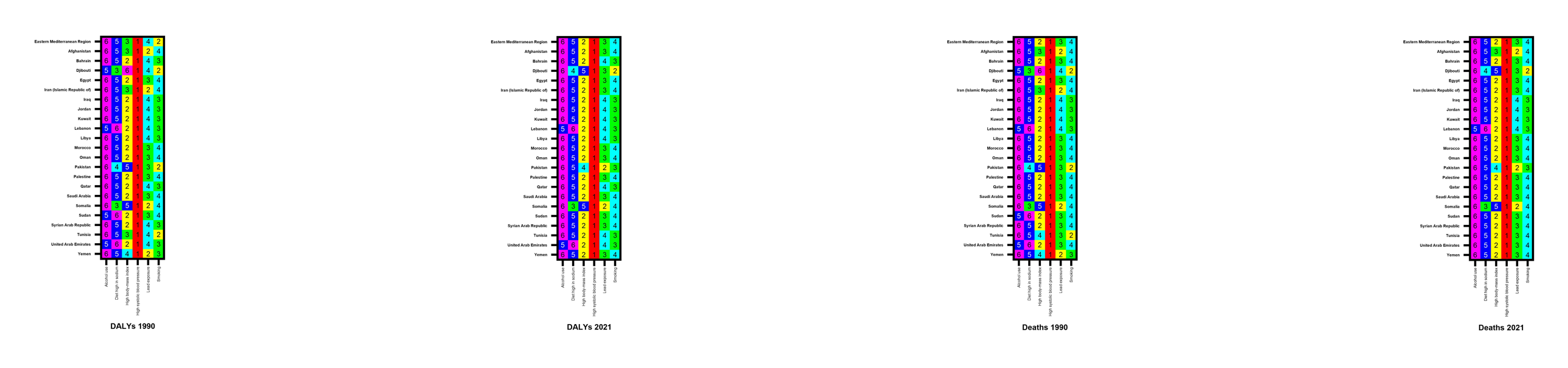

Supplement: Supplementary file 3 — Additional file 3. [file 43044_2025_693_MOESM3_ESM.jpg]

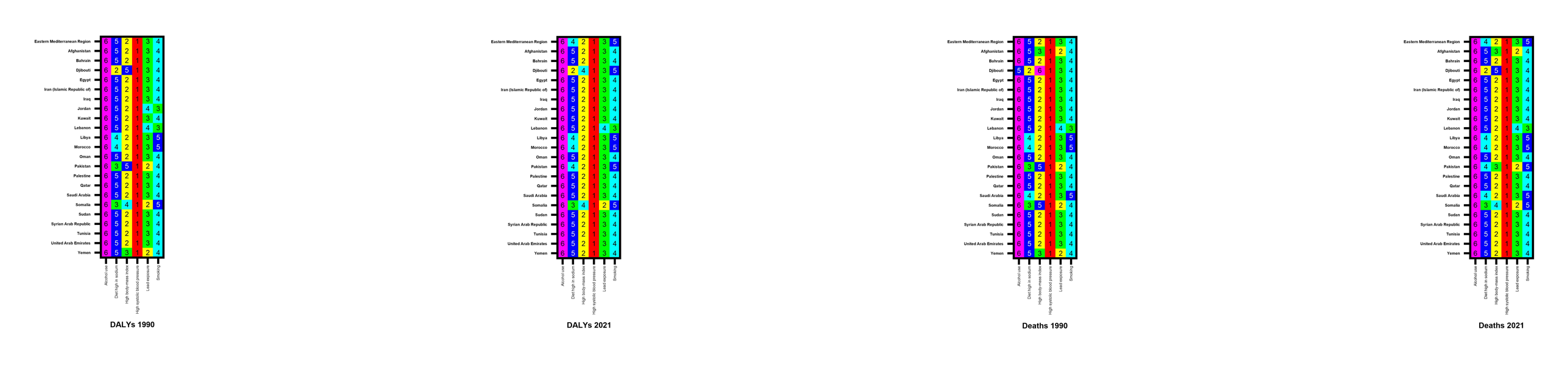

Supplement: Supplementary file 4 — Additional file 4. [file 43044_2025_693_MOESM4_ESM.jpg]

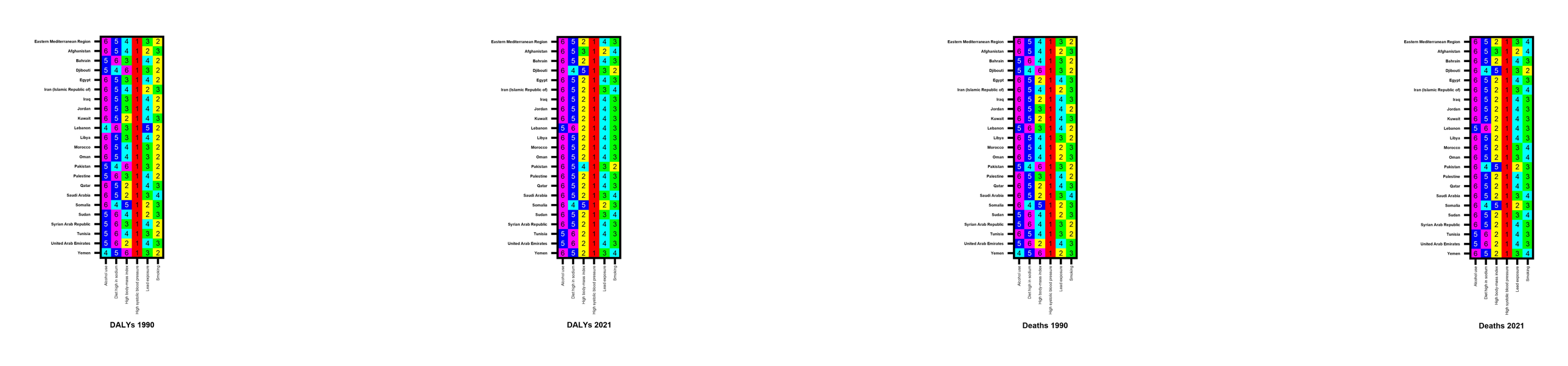

Supplement: Supplementary file 5 — Additional file 5. [file 43044_2025_693_MOESM5_ESM.jpg]

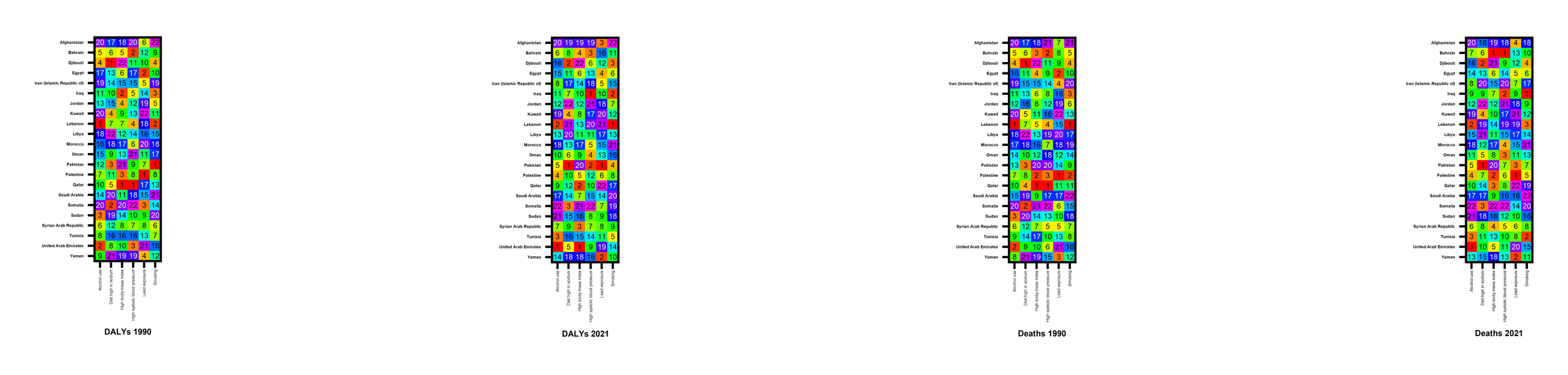

Supplement: Supplementary file 6 — Additional file 6. [file 43044_2025_693_MOESM6_ESM.jpg]

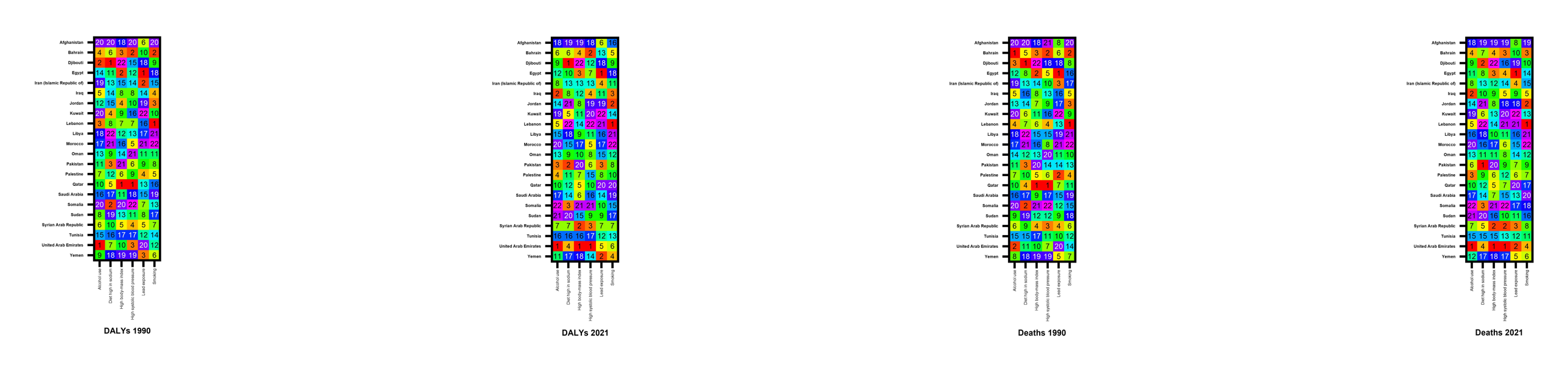

Supplement: Supplementary file 7 — Additional file 7. [file 43044_2025_693_MOESM7_ESM.jpg]

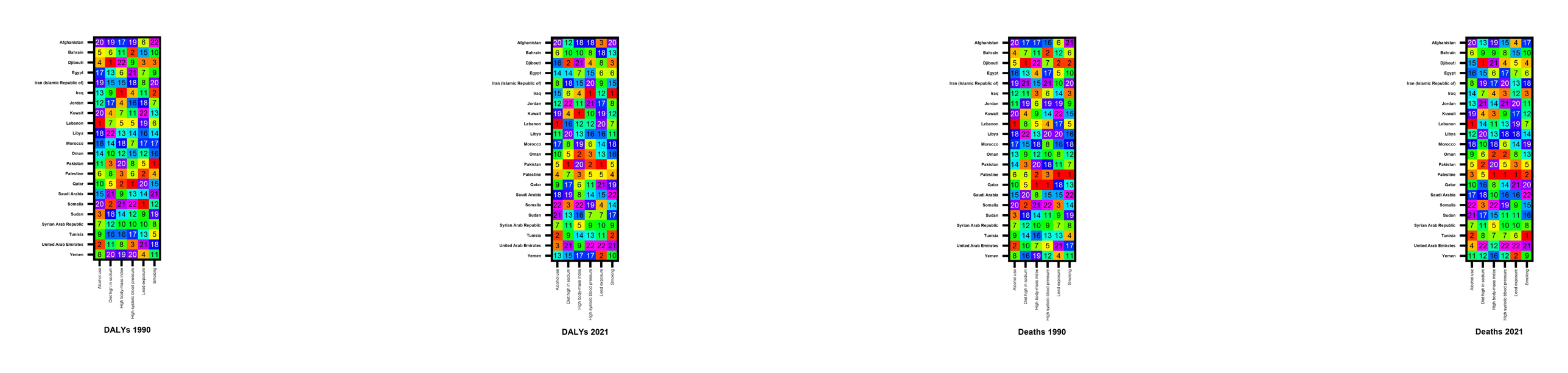

Supplement: Supplementary file 8 — Additional file 8. [file 43044_2025_693_MOESM8_ESM.jpg]
