# Supplementary material for: Atrial fibrillation and flutter in the Eastern Mediterranean: burden, disparities, and risk factor contributions from 1990 to 2021
Source: Egypt Heart J. 2025 Oct 22;77:98. doi: 10.1186/s43044-025-00693-5 (PMC12546237; doi:10.1186/s43044-025-00693-5)
Supplement: Supplementary file 9 — Additional file 9. [file 43044_2025_693_MOESM9_ESM.docx]

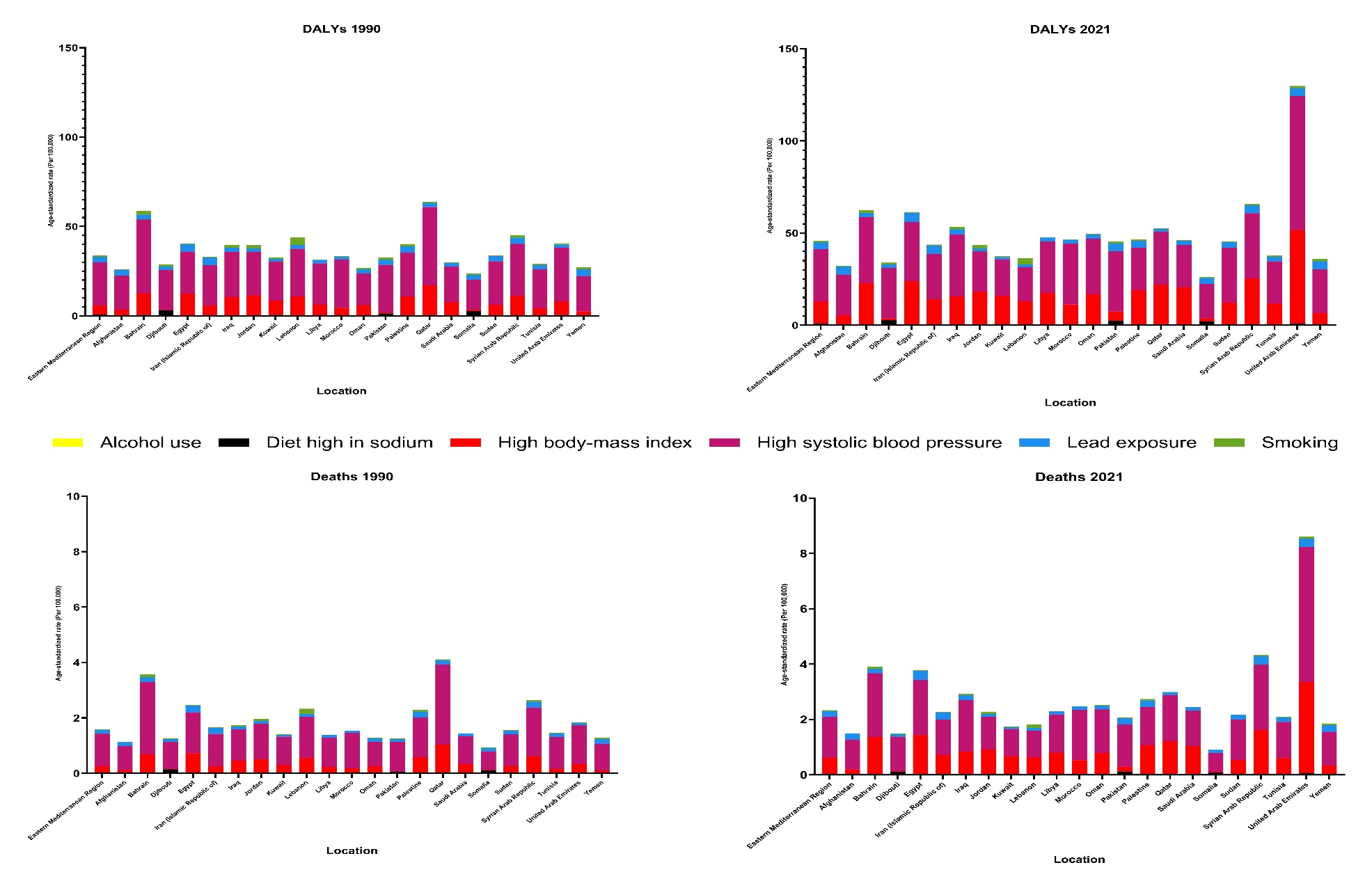


**Figure S1.** Age-standardized rate of disability-adjusted life years (DALYs) and deaths of atrial fibrillation and flutter attributable to risk factors among women in 1990 and 2021 in the Eastern Mediterranean Region


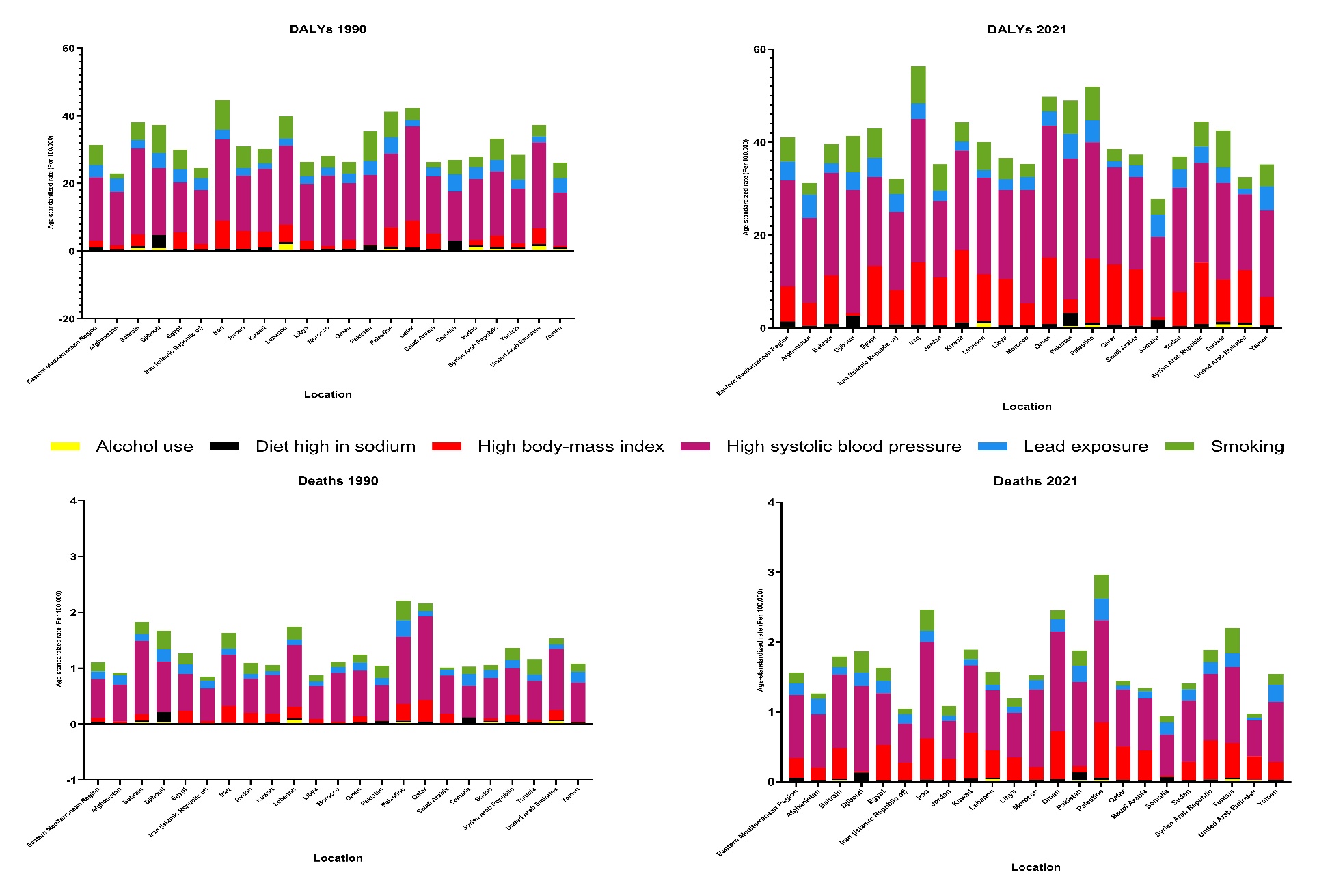


**Figure S2.** Age-standardized rate of disability-adjusted life years (DALYs) and deaths of atrial fibrillation and flutter attributable to risk factors among men in 1990 and 2021 in the Eastern Mediterranean Region


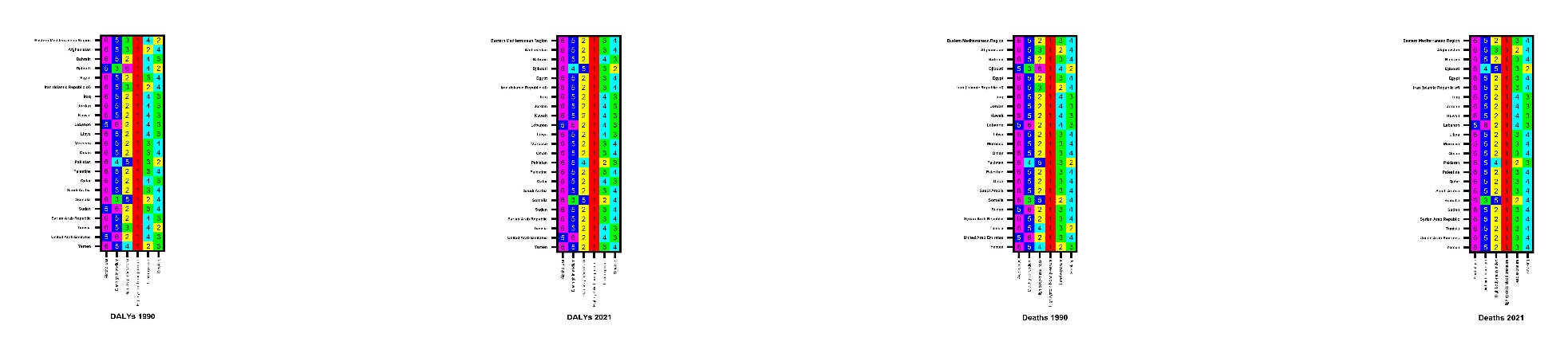


**Figure S3.** Regional and country-specific ranking of age-standardized rate of disability-adjusted life years (DALYs) and deaths of atrial fibrillation and flutter attributable to risk factors among both sexes in 1990 and 2021 in the Eastern Mediterranean Region


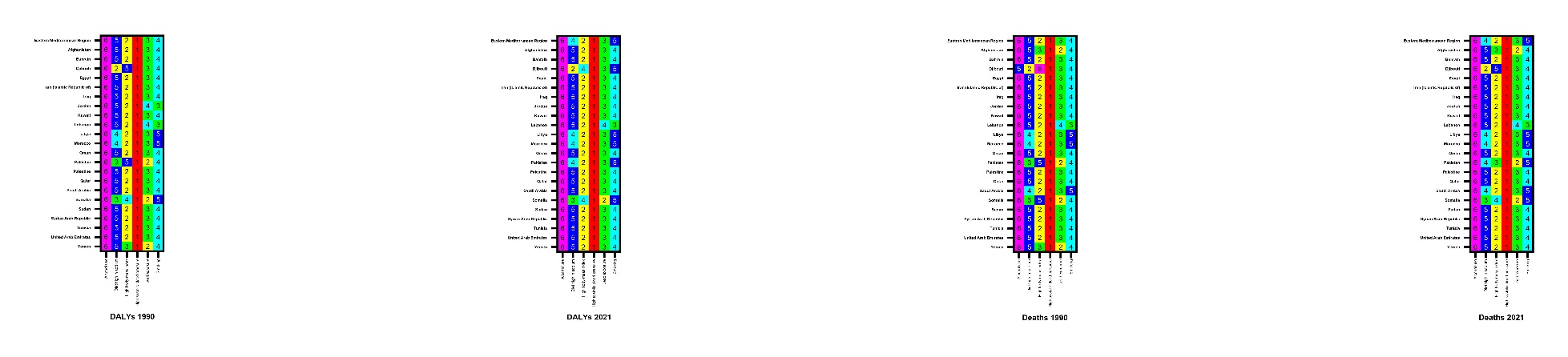


**Figure S4.** Regional and country-specific ranking of age-standardized rate of disability-adjusted life years (DALYs) and deaths of atrial fibrillation and flutter attributable to risk factors among women in 1990 and 2021 in the Eastern Mediterranean Region


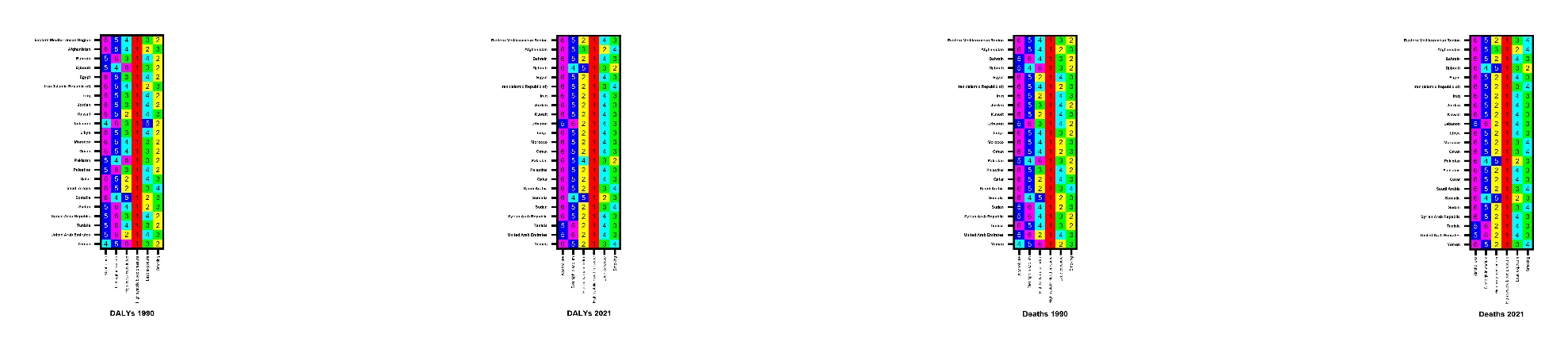


**Figure S5.** Regional and country-specific ranking of age-standardized rate of disability-adjusted life years (DALYs) and deaths of atrial fibrillation and flutter attributable to risk factors among men in 1990 and 2021 in the Eastern Mediterranean Region


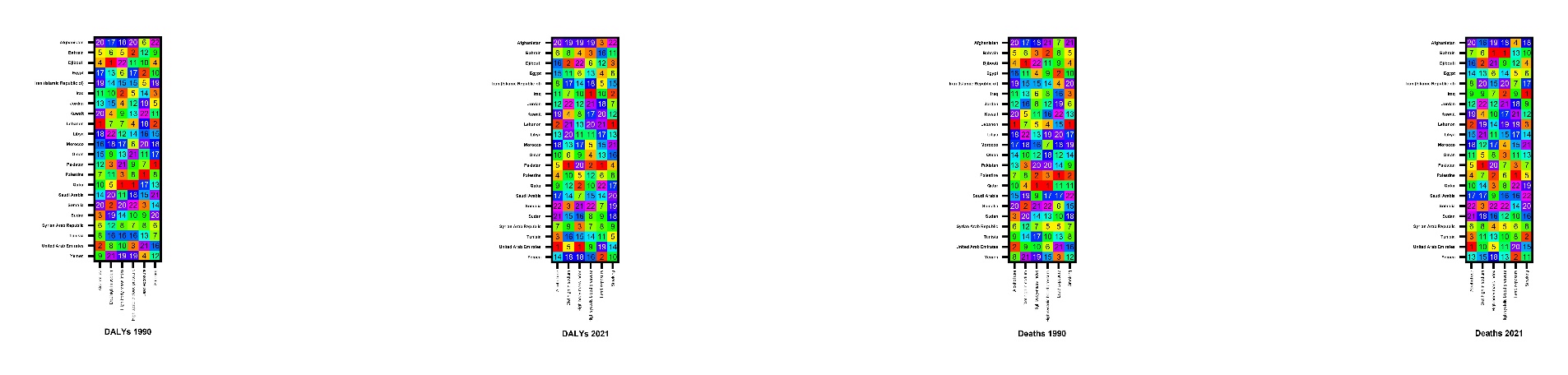


**Figure S6.** Country-level ranking of the age-standardized rate of disability-adjusted life years (DALYs) and deaths of atrial fibrillation and flutter attributable to risk factors among both sexes in 1990 and 2021 in the Eastern Mediterranean Region


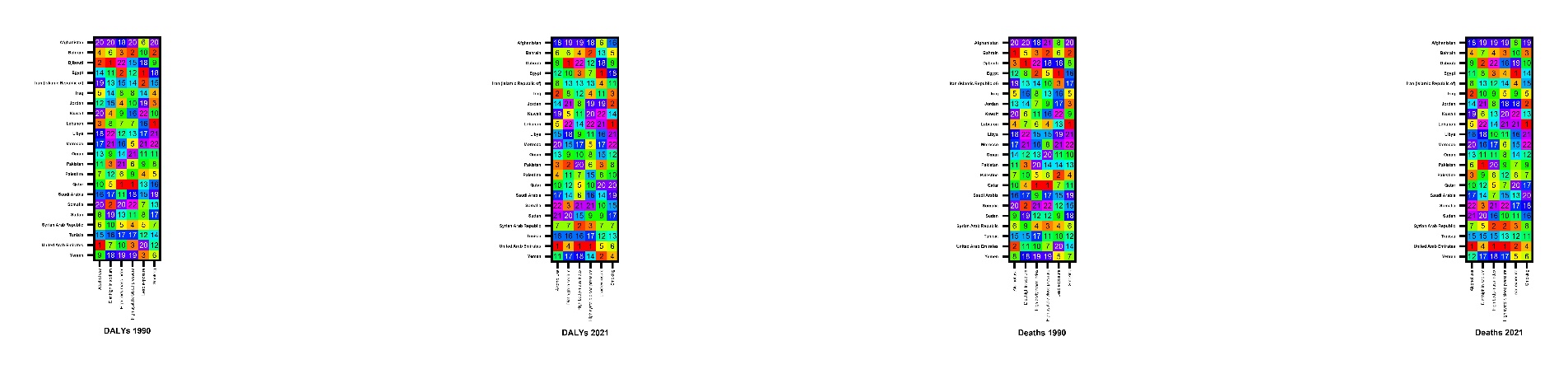


**Figure S7.** Country-level ranking of the age-standardized rate of disability-adjusted life years (DALYs) and deaths of atrial fibrillation and flutter attributable to risk factors among women in 1990 and 2021 in the Eastern Mediterranean Region


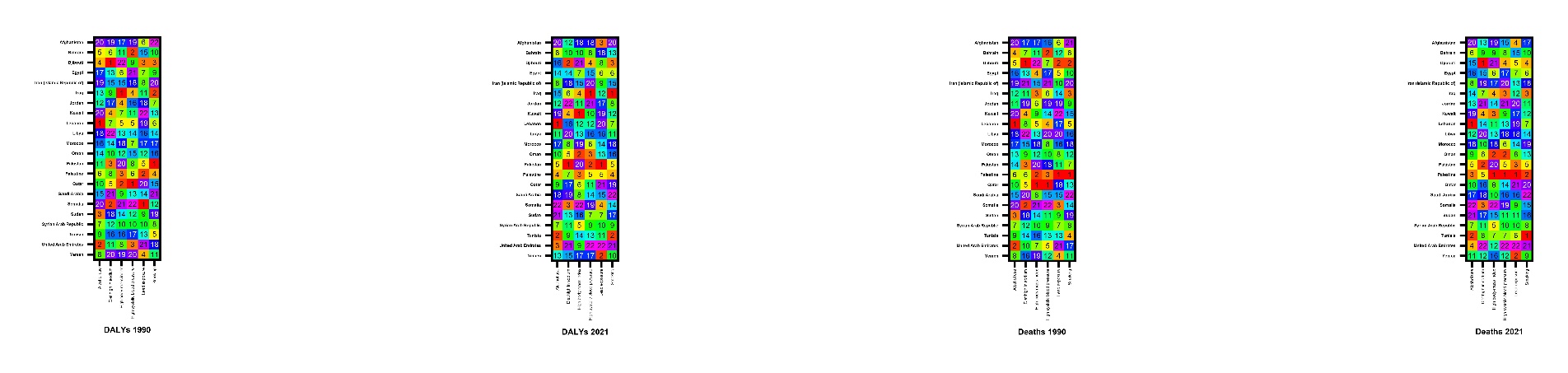


**Figure S8.** Country-level ranking of the age-standardized rate of disability-adjusted life years (DALYs) and deaths of atrial fibrillation and flutter attributable to risk factors among men in 1990 and 2021 in the Eastern Mediterranean Region

**Table S1.** Time trend of age-standardized rate of incidence, prevalence, disability-adjusted life years (DALYs), and deaths of atrial fibrillation and flutter in the Eastern Mediterranean Region from 1990 to 2021, by sex

| Measure | Sex | Year | Age-standardized Rate |
| --- | --- | --- | --- |
| Incidence | Both | 1990 | 40.6 (30 to 54.8) |
| Incidence | Both | 1991 | 40.7 (30.1 to 54.7) |
| Incidence | Both | 1992 | 40.8 (30.2 to 54.7) |
| Incidence | Both | 1993 | 40.9 (30.3 to 54.6) |
| Incidence | Both | 1994 | 40.9 (30.4 to 54.5) |
| Incidence | Both | 1995 | 41 (30.5 to 54.5) |
| Incidence | Both | 1996 | 41 (30.3 to 54.5) |
| Incidence | Both | 1997 | 41 (30.3 to 54.5) |
| Incidence | Both | 1998 | 41 (30.3 to 54.6) |
| Incidence | Both | 1999 | 41 (30.3 to 54.6) |
| Incidence | Both | 2000 | 41 (30.2 to 54.7) |
| Incidence | Both | 2001 | 41 (30.2 to 54.7) |
| Incidence | Both | 2002 | 41.1 (30.3 to 54.8) |
| Incidence | Both | 2003 | 41.1 (30.4 to 54.9) |
| Incidence | Both | 2004 | 41.2 (30.4 to 55) |
| Incidence | Both | 2005 | 41.2 (30.5 to 55.1) |
| Incidence | Both | 2006 | 41.3 (30.5 to 55.1) |
| Incidence | Both | 2007 | 41.4 (30.6 to 55.3) |
| Incidence | Both | 2008 | 41.5 (30.6 to 55.4) |
| Incidence | Both | 2009 | 41.5 (30.7 to 55.5) |
| Incidence | Both | 2010 | 41.6 (30.8 to 55.6) |
| Incidence | Both | 2011 | 41.6 (30.8 to 55.6) |
| Incidence | Both | 2012 | 41.6 (30.8 to 55.6) |
| Incidence | Both | 2013 | 41.6 (30.8 to 55.5) |
| Incidence | Both | 2014 | 41.6 (30.8 to 55.5) |
| Incidence | Both | 2015 | 41.6 (30.8 to 55.6) |
| Incidence | Both | 2016 | 41.7 (30.8 to 55.5) |
| Incidence | Both | 2017 | 41.7 (30.9 to 55.7) |
| Incidence | Both | 2018 | 41.8 (31 to 55.8) |
| Incidence | Both | 2019 | 41.9 (31.1 to 55.9) |
| Incidence | Both | 2020 | 41.8 (30.8 to 55.9) |
| Incidence | Both | 2021 | 41.8 (31 to 56.1) |
| Incidence | Women | 1990 | 36.1 (26.6 to 48.5) |
| Incidence | Women | 1991 | 36.3 (26.9 to 48.7) |
| Incidence | Women | 1992 | 36.5 (27.1 to 49) |
| Incidence | Women | 1993 | 36.7 (27.2 to 49.1) |
| Incidence | Women | 1994 | 36.9 (27.4 to 49.3) |
| Incidence | Women | 1995 | 37 (27.5 to 49.4) |
| Incidence | Women | 1996 | 37 (27.4 to 49.4) |
| Incidence | Women | 1997 | 37.1 (27.4 to 49.4) |
| Incidence | Women | 1998 | 37.1 (27.4 to 49.4) |
| Incidence | Women | 1999 | 37.2 (27.4 to 49.5) |
| Incidence | Women | 2000 | 37.2 (27.4 to 49.6) |
| Incidence | Women | 2001 | 37.3 (27.4 to 49.7) |
| Incidence | Women | 2002 | 37.3 (27.5 to 49.8) |
| Incidence | Women | 2003 | 37.4 (27.7 to 49.9) |
| Incidence | Women | 2004 | 37.5 (27.8 to 50) |
| Incidence | Women | 2005 | 37.5 (27.8 to 50.1) |
| Incidence | Women | 2006 | 37.6 (27.9 to 50.2) |
| Incidence | Women | 2007 | 37.8 (28 to 50.4) |
| Incidence | Women | 2008 | 37.9 (28 to 50.5) |
| Incidence | Women | 2009 | 38 (28.1 to 50.7) |
| Incidence | Women | 2010 | 38 (28.1 to 50.7) |
| Incidence | Women | 2011 | 38 (28.1 to 50.7) |
| Incidence | Women | 2012 | 37.9 (28.1 to 50.5) |
| Incidence | Women | 2013 | 37.8 (28.1 to 50.4) |
| Incidence | Women | 2014 | 37.7 (28 to 50.3) |
| Incidence | Women | 2015 | 37.7 (28 to 50.3) |
| Incidence | Women | 2016 | 37.7 (28.1 to 50.4) |
| Incidence | Women | 2017 | 37.8 (28.1 to 50.6) |
| Incidence | Women | 2018 | 37.9 (28.2 to 50.7) |
| Incidence | Women | 2019 | 38 (28.2 to 50.9) |
| Incidence | Women | 2020 | 38 (28.1 to 50.9) |
| Incidence | Women | 2021 | 37.9 (28.2 to 50.7) |
| Incidence | Men | 1990 | 44.7 (33 to 60.1) |
| Incidence | Men | 1991 | 44.6 (32.9 to 59.8) |
| Incidence | Men | 1992 | 44.6 (32.9 to 59.5) |
| Incidence | Men | 1993 | 44.6 (32.8 to 59.3) |
| Incidence | Men | 1994 | 44.5 (32.9 to 59.1) |
| Incidence | Men | 1995 | 44.5 (32.9 to 59) |
| Incidence | Men | 1996 | 44.5 (32.8 to 58.9) |
| Incidence | Men | 1997 | 44.4 (32.8 to 59) |
| Incidence | Men | 1998 | 44.4 (32.8 to 59.2) |
| Incidence | Men | 1999 | 44.4 (32.8 to 59.5) |
| Incidence | Men | 2000 | 44.4 (32.7 to 59.6) |
| Incidence | Men | 2001 | 44.4 (32.8 to 59.6) |
| Incidence | Men | 2002 | 44.4 (32.8 to 59.6) |
| Incidence | Men | 2003 | 44.4 (32.8 to 59.6) |
| Incidence | Men | 2004 | 44.4 (32.9 to 59.5) |
| Incidence | Men | 2005 | 44.5 (32.9 to 59.5) |
| Incidence | Men | 2006 | 44.5 (32.9 to 59.5) |
| Incidence | Men | 2007 | 44.6 (32.9 to 59.8) |
| Incidence | Men | 2008 | 44.7 (33 to 60.1) |
| Incidence | Men | 2009 | 44.8 (33.1 to 60.1) |
| Incidence | Men | 2010 | 44.8 (33.2 to 60.2) |
| Incidence | Men | 2011 | 44.9 (33.2 to 60.3) |
| Incidence | Men | 2012 | 45 (33.2 to 60.4) |
| Incidence | Men | 2013 | 45.1 (33.2 to 60.4) |
| Incidence | Men | 2014 | 45.1 (33.2 to 60.4) |
| Incidence | Men | 2015 | 45.2 (33.3 to 60.4) |
| Incidence | Men | 2016 | 45.3 (33.3 to 60.6) |
| Incidence | Men | 2017 | 45.4 (33.4 to 60.8) |
| Incidence | Men | 2018 | 45.4 (33.5 to 60.9) |
| Incidence | Men | 2019 | 45.5 (33.6 to 61.1) |
| Incidence | Men | 2020 | 45.4 (33.4 to 61) |
| Incidence | Men | 2021 | 45.6 (33.7 to 61.2) |
| Prevalence | Both | 1990 | 419.7 (324.2 to 551) |
| Prevalence | Both | 1991 | 421.1 (326 to 553.2) |
| Prevalence | Both | 1992 | 422.4 (327.1 to 555) |
| Prevalence | Both | 1993 | 423.4 (327.1 to 556.7) |
| Prevalence | Both | 1994 | 424.2 (327 to 557.5) |
| Prevalence | Both | 1995 | 424.6 (326.5 to 557.7) |
| Prevalence | Both | 1996 | 424.8 (327.4 to 558.1) |
| Prevalence | Both | 1997 | 425 (328.4 to 557.7) |
| Prevalence | Both | 1998 | 425.2 (329.3 to 557.4) |
| Prevalence | Both | 1999 | 425.4 (330.1 to 557.5) |
| Prevalence | Both | 2000 | 425.6 (330.3 to 557.4) |
| Prevalence | Both | 2001 | 425.9 (330.4 to 558.8) |
| Prevalence | Both | 2002 | 426.4 (330.7 to 560.7) |
| Prevalence | Both | 2003 | 427.1 (330.7 to 562.7) |
| Prevalence | Both | 2004 | 427.8 (330.8 to 564.7) |
| Prevalence | Both | 2005 | 428.6 (330.6 to 566.1) |
| Prevalence | Both | 2006 | 429.5 (331.3 to 566.9) |
| Prevalence | Both | 2007 | 430.7 (332.2 to 568.4) |
| Prevalence | Both | 2008 | 432 (333.1 to 569.9) |
| Prevalence | Both | 2009 | 433.2 (333.8 to 571.4) |
| Prevalence | Both | 2010 | 434 (334 to 572.4) |
| Prevalence | Both | 2011 | 434.3 (334.7 to 572.2) |
| Prevalence | Both | 2012 | 434.3 (334.8 to 571.7) |
| Prevalence | Both | 2013 | 434.2 (334.8 to 571.1) |
| Prevalence | Both | 2014 | 434.3 (334.9 to 570.9) |
| Prevalence | Both | 2015 | 434.7 (335.2 to 571.1) |
| Prevalence | Both | 2016 | 435.6 (336.3 to 571.8) |
| Prevalence | Both | 2017 | 436.9 (337.6 to 573.5) |
| Prevalence | Both | 2018 | 438.1 (338.6 to 575) |
| Prevalence | Both | 2019 | 438.8 (339.2 to 575.1) |
| Prevalence | Both | 2020 | 438.4 (338.3 to 576.4) |
| Prevalence | Both | 2021 | 439 (338.8 to 577) |
| Prevalence | Women | 1990 | 363.7 (278.2 to 478.8) |
| Prevalence | Women | 1991 | 366.6 (279.9 to 482.5) |
| Prevalence | Women | 1992 | 369.3 (282 to 487.3) |
| Prevalence | Women | 1993 | 371.6 (283.6 to 491.8) |
| Prevalence | Women | 1994 | 373.5 (284.9 to 494.1) |
| Prevalence | Women | 1995 | 374.7 (285.6 to 494.9) |
| Prevalence | Women | 1996 | 375.5 (285.9 to 496.4) |
| Prevalence | Women | 1997 | 376.3 (286.2 to 497.9) |
| Prevalence | Women | 1998 | 377.1 (286.9 to 498.4) |
| Prevalence | Women | 1999 | 377.8 (288 to 498.7) |
| Prevalence | Women | 2000 | 378.5 (289 to 498.1) |
| Prevalence | Women | 2001 | 379.3 (289.9 to 500.1) |
| Prevalence | Women | 2002 | 380.4 (291.1 to 502.1) |
| Prevalence | Women | 2003 | 381.5 (292.3 to 503.8) |
| Prevalence | Women | 2004 | 382.7 (293.2 to 504.6) |
| Prevalence | Women | 2005 | 383.9 (294.8 to 505.1) |
| Prevalence | Women | 2006 | 385.2 (295.5 to 507.6) |
| Prevalence | Women | 2007 | 386.9 (296.7 to 510.7) |
| Prevalence | Women | 2008 | 388.7 (298.4 to 513.8) |
| Prevalence | Women | 2009 | 390.2 (299.9 to 516.1) |
| Prevalence | Women | 2010 | 391.2 (300.6 to 517) |
| Prevalence | Women | 2011 | 391.1 (300.4 to 517.2) |
| Prevalence | Women | 2012 | 390.1 (299.3 to 516.2) |
| Prevalence | Women | 2013 | 388.8 (298.2 to 514) |
| Prevalence | Women | 2014 | 387.8 (297.9 to 510.8) |
| Prevalence | Women | 2015 | 387.7 (298 to 509.8) |
| Prevalence | Women | 2016 | 388.5 (298.1 to 511) |
| Prevalence | Women | 2017 | 389.7 (299.1 to 511.9) |
| Prevalence | Women | 2018 | 390.9 (300 to 512.9) |
| Prevalence | Women | 2019 | 391.8 (299.7 to 513.5) |
| Prevalence | Women | 2020 | 392 (302.6 to 517.8) |
| Prevalence | Women | 2021 | 391.3 (299.9 to 515.5) |
| Prevalence | Men | 1990 | 470 (361.7 to 616.3) |
| Prevalence | Men | 1991 | 469.9 (362 to 615.9) |
| Prevalence | Men | 1992 | 469.8 (362.3 to 615.2) |
| Prevalence | Men | 1993 | 469.6 (362.5 to 614.4) |
| Prevalence | Men | 1994 | 469.3 (361.9 to 613.3) |
| Prevalence | Men | 1995 | 469 (360.1 to 612.1) |
| Prevalence | Men | 1996 | 468.6 (360.9 to 611.9) |
| Prevalence | Men | 1997 | 468.2 (361.8 to 610.6) |
| Prevalence | Men | 1998 | 467.9 (362.6 to 609.3) |
| Prevalence | Men | 1999 | 467.6 (363.3 to 608.1) |
| Prevalence | Men | 2000 | 467.3 (363.1 to 607.3) |
| Prevalence | Men | 2001 | 467.1 (362.4 to 608.5) |
| Prevalence | Men | 2002 | 467.1 (361.6 to 610.8) |
| Prevalence | Men | 2003 | 467.3 (360.8 to 613.2) |
| Prevalence | Men | 2004 | 467.7 (360.3 to 615.7) |
| Prevalence | Men | 2005 | 468.1 (360.5 to 616.9) |
| Prevalence | Men | 2006 | 468.7 (361 to 617.4) |
| Prevalence | Men | 2007 | 469.6 (361.3 to 618.3) |
| Prevalence | Men | 2008 | 470.6 (361.6 to 619.1) |
| Prevalence | Men | 2009 | 471.5 (361.8 to 619.7) |
| Prevalence | Men | 2010 | 472.2 (362.1 to 620) |
| Prevalence | Men | 2011 | 473 (363.1 to 620.7) |
| Prevalence | Men | 2012 | 474.1 (364.7 to 621.8) |
| Prevalence | Men | 2013 | 475.2 (366.2 to 622.8) |
| Prevalence | Men | 2014 | 476.3 (367.7 to 623.7) |
| Prevalence | Men | 2015 | 477.3 (368.7 to 624.3) |
| Prevalence | Men | 2016 | 478.4 (369.5 to 624.5) |
| Prevalence | Men | 2017 | 479.8 (370.1 to 625.3) |
| Prevalence | Men | 2018 | 481 (370.5 to 626.7) |
| Prevalence | Men | 2019 | 481.6 (371 to 628.2) |
| Prevalence | Men | 2020 | 480.9 (369.9 to 631.7) |
| Prevalence | Men | 2021 | 483.3 (372.4 to 632.3) |
| DALYs | Both | 1990 | 73.7 (56.6 to 93.6) |
| DALYs | Both | 1991 | 74.3 (57.5 to 93.9) |
| DALYs | Both | 1992 | 74.9 (58.1 to 94.5) |
| DALYs | Both | 1993 | 75.7 (58.4 to 96.1) |
| DALYs | Both | 1994 | 76.4 (59.3 to 96.8) |
| DALYs | Both | 1995 | 77 (60 to 96.9) |
| DALYs | Both | 1996 | 77.5 (60.5 to 97.9) |
| DALYs | Both | 1997 | 78 (61.4 to 98) |
| DALYs | Both | 1998 | 78.3 (61.7 to 98.1) |
| DALYs | Both | 1999 | 78.7 (62.2 to 98.6) |
| DALYs | Both | 2000 | 79.1 (62.3 to 98.7) |
| DALYs | Both | 2001 | 79.4 (63.4 to 98.8) |
| DALYs | Both | 2002 | 79.9 (64.1 to 99.2) |
| DALYs | Both | 2003 | 80.2 (64.7 to 99) |
| DALYs | Both | 2004 | 80.4 (64.7 to 99) |
| DALYs | Both | 2005 | 80.5 (64.8 to 99) |
| DALYs | Both | 2006 | 80.5 (65.6 to 98.5) |
| DALYs | Both | 2007 | 80.8 (65.2 to 98.6) |
| DALYs | Both | 2008 | 81 (65.9 to 98.2) |
| DALYs | Both | 2009 | 81.2 (65.8 to 98.2) |
| DALYs | Both | 2010 | 81.4 (65.8 to 99.5) |
| DALYs | Both | 2011 | 81.4 (66.7 to 98.7) |
| DALYs | Both | 2012 | 81.3 (65.4 to 98.7) |
| DALYs | Both | 2013 | 81.3 (66.4 to 97.7) |
| DALYs | Both | 2014 | 81.7 (66.5 to 98.8) |
| DALYs | Both | 2015 | 82 (66.7 to 99.1) |
| DALYs | Both | 2016 | 82.4 (67.1 to 99) |
| DALYs | Both | 2017 | 82.7 (66.9 to 99.2) |
| DALYs | Both | 2018 | 82.9 (67.9 to 99.5) |
| DALYs | Both | 2019 | 83.3 (68.9 to 98.4) |
| DALYs | Both | 2020 | 83.4 (68.4 to 99.3) |
| DALYs | Both | 2021 | 83.4 (68.7 to 99.8) |
| DALYs | Women | 1990 | 76.5 (57.6 to 102.3) |
| DALYs | Women | 1991 | 77.2 (58.5 to 103) |
| DALYs | Women | 1992 | 77.9 (59.4 to 103.2) |
| DALYs | Women | 1993 | 78.8 (60.2 to 104.3) |
| DALYs | Women | 1994 | 79.7 (61 to 104.8) |
| DALYs | Women | 1995 | 80.4 (61.8 to 104.7) |
| DALYs | Women | 1996 | 81.1 (62.7 to 105.3) |
| DALYs | Women | 1997 | 81.7 (63 to 105.2) |
| DALYs | Women | 1998 | 82.2 (63.7 to 107) |
| DALYs | Women | 1999 | 83 (64.4 to 106.4) |
| DALYs | Women | 2000 | 83.9 (65.4 to 106.6) |
| DALYs | Women | 2001 | 84.9 (65.9 to 107.1) |
| DALYs | Women | 2002 | 85.6 (67.1 to 107.5) |
| DALYs | Women | 2003 | 86 (68 to 108.2) |
| DALYs | Women | 2004 | 86.2 (68.7 to 107.3) |
| DALYs | Women | 2005 | 86.4 (68.7 to 107.4) |
| DALYs | Women | 2006 | 86.5 (69.5 to 106.5) |
| DALYs | Women | 2007 | 86.6 (68.6 to 106.6) |
| DALYs | Women | 2008 | 86.8 (68.9 to 106.3) |
| DALYs | Women | 2009 | 87.2 (70.7 to 107.7) |
| DALYs | Women | 2010 | 87.5 (70.2 to 106.5) |
| DALYs | Women | 2011 | 87.5 (69.8 to 107) |
| DALYs | Women | 2012 | 87.3 (69.6 to 105.7) |
| DALYs | Women | 2013 | 87.3 (69.7 to 105) |
| DALYs | Women | 2014 | 87.7 (69.9 to 105.5) |
| DALYs | Women | 2015 | 88.2 (70.1 to 106.2) |
| DALYs | Women | 2016 | 88.7 (70.5 to 106) |
| DALYs | Women | 2017 | 89 (71.7 to 105.5) |
| DALYs | Women | 2018 | 89.2 (71.8 to 106.9) |
| DALYs | Women | 2019 | 89.7 (71.9 to 105.2) |
| DALYs | Women | 2020 | 90 (71.6 to 106.6) |
| DALYs | Women | 2021 | 89.9 (72.9 to 105.4) |
| DALYs | Men | 1990 | 71.3 (51.5 to 91.3) |
| DALYs | Men | 1991 | 71.7 (52.1 to 91.5) |
| DALYs | Men | 1992 | 72.2 (52.2 to 91.8) |
| DALYs | Men | 1993 | 72.9 (52.8 to 92) |
| DALYs | Men | 1994 | 73.5 (54 to 92.8) |
| DALYs | Men | 1995 | 74.1 (54.5 to 93.7) |
| DALYs | Men | 1996 | 74.4 (55 to 93.9) |
| DALYs | Men | 1997 | 74.7 (55.3 to 93.7) |
| DALYs | Men | 1998 | 74.9 (56.3 to 94) |
| DALYs | Men | 1999 | 75 (56.4 to 94.3) |
| DALYs | Men | 2000 | 74.9 (56.4 to 93.2) |
| DALYs | Men | 2001 | 74.7 (56.8 to 93.5) |
| DALYs | Men | 2002 | 74.9 (57.4 to 93.6) |
| DALYs | Men | 2003 | 75.2 (57.7 to 94.3) |
| DALYs | Men | 2004 | 75.3 (58.1 to 94) |
| DALYs | Men | 2005 | 75.3 (57.9 to 94.1) |
| DALYs | Men | 2006 | 75.4 (58.6 to 93.5) |
| DALYs | Men | 2007 | 75.7 (58.9 to 94.1) |
| DALYs | Men | 2008 | 75.9 (59.1 to 94.6) |
| DALYs | Men | 2009 | 76 (59 to 94.2) |
| DALYs | Men | 2010 | 76 (59.8 to 94.4) |
| DALYs | Men | 2011 | 75.9 (60.4 to 93.1) |
| DALYs | Men | 2012 | 75.9 (59.6 to 94.2) |
| DALYs | Men | 2013 | 76 (59.9 to 93.5) |
| DALYs | Men | 2014 | 76.3 (61.3 to 94.8) |
| DALYs | Men | 2015 | 76.6 (60.9 to 93.7) |
| DALYs | Men | 2016 | 76.8 (61.4 to 94.3) |
| DALYs | Men | 2017 | 77 (61.6 to 94.2) |
| DALYs | Men | 2018 | 77.2 (61.7 to 94.1) |
| DALYs | Men | 2019 | 77.5 (62.2 to 95.3) |
| DALYs | Men | 2020 | 77.5 (62.4 to 94.5) |
| DALYs | Men | 2021 | 77.5 (62.6 to 95.2) |
| Deaths | Both | 1990 | 3.2 (2.3 to 4.2) |
| Deaths | Both | 1991 | 3.2 (2.3 to 4.2) |
| Deaths | Both | 1992 | 3.3 (2.4 to 4.2) |
| Deaths | Both | 1993 | 3.3 (2.4 to 4.3) |
| Deaths | Both | 1994 | 3.4 (2.5 to 4.3) |
| Deaths | Both | 1995 | 3.4 (2.5 to 4.4) |
| Deaths | Both | 1996 | 3.4 (2.6 to 4.4) |
| Deaths | Both | 1997 | 3.5 (2.6 to 4.4) |
| Deaths | Both | 1998 | 3.5 (2.7 to 4.4) |
| Deaths | Both | 1999 | 3.5 (2.7 to 4.4) |
| Deaths | Both | 2000 | 3.6 (2.7 to 4.4) |
| Deaths | Both | 2001 | 3.6 (2.8 to 4.5) |
| Deaths | Both | 2002 | 3.6 (2.8 to 4.5) |
| Deaths | Both | 2003 | 3.7 (2.9 to 4.5) |
| Deaths | Both | 2004 | 3.7 (2.9 to 4.4) |
| Deaths | Both | 2005 | 3.7 (2.9 to 4.4) |
| Deaths | Both | 2006 | 3.7 (3 to 4.4) |
| Deaths | Both | 2007 | 3.7 (2.9 to 4.4) |
| Deaths | Both | 2008 | 3.7 (3 to 4.5) |
| Deaths | Both | 2009 | 3.8 (3 to 4.5) |
| Deaths | Both | 2010 | 3.8 (3 to 4.5) |
| Deaths | Both | 2011 | 3.8 (3 to 4.4) |
| Deaths | Both | 2012 | 3.8 (3 to 4.4) |
| Deaths | Both | 2013 | 3.8 (3 to 4.4) |
| Deaths | Both | 2014 | 3.8 (3.1 to 4.4) |
| Deaths | Both | 2015 | 3.8 (3.1 to 4.4) |
| Deaths | Both | 2016 | 3.9 (3.1 to 4.5) |
| Deaths | Both | 2017 | 3.9 (3.1 to 4.5) |
| Deaths | Both | 2018 | 3.9 (3.1 to 4.5) |
| Deaths | Both | 2019 | 3.9 (3.2 to 4.6) |
| Deaths | Both | 2020 | 3.9 (3.2 to 4.5) |
| Deaths | Both | 2021 | 3.9 (3.2 to 4.5) |
| Deaths | Women | 1990 | 3.7 (2.7 to 5.3) |
| Deaths | Women | 1991 | 3.8 (2.7 to 5.3) |
| Deaths | Women | 1992 | 3.8 (2.7 to 5.3) |
| Deaths | Women | 1993 | 3.8 (2.8 to 5.3) |
| Deaths | Women | 1994 | 3.9 (2.8 to 5.4) |
| Deaths | Women | 1995 | 3.9 (2.9 to 5.5) |
| Deaths | Women | 1996 | 4 (2.9 to 5.4) |
| Deaths | Women | 1997 | 4 (3 to 5.5) |
| Deaths | Women | 1998 | 4 (3 to 5.5) |
| Deaths | Women | 1999 | 4.1 (3.1 to 5.6) |
| Deaths | Women | 2000 | 4.2 (3.2 to 5.6) |
| Deaths | Women | 2001 | 4.3 (3.3 to 5.6) |
| Deaths | Women | 2002 | 4.3 (3.3 to 5.6) |
| Deaths | Women | 2003 | 4.4 (3.4 to 5.7) |
| Deaths | Women | 2004 | 4.4 (3.4 to 5.6) |
| Deaths | Women | 2005 | 4.4 (3.4 to 5.5) |
| Deaths | Women | 2006 | 4.4 (3.4 to 5.5) |
| Deaths | Women | 2007 | 4.4 (3.5 to 5.5) |
| Deaths | Women | 2008 | 4.4 (3.4 to 5.6) |
| Deaths | Women | 2009 | 4.5 (3.4 to 5.5) |
| Deaths | Women | 2010 | 4.5 (3.5 to 5.5) |
| Deaths | Women | 2011 | 4.5 (3.5 to 5.5) |
| Deaths | Women | 2012 | 4.5 (3.5 to 5.5) |
| Deaths | Women | 2013 | 4.5 (3.6 to 5.5) |
| Deaths | Women | 2014 | 4.6 (3.6 to 5.5) |
| Deaths | Women | 2015 | 4.6 (3.6 to 5.6) |
| Deaths | Women | 2016 | 4.7 (3.6 to 5.6) |
| Deaths | Women | 2017 | 4.7 (3.7 to 5.6) |
| Deaths | Women | 2018 | 4.7 (3.7 to 5.6) |
| Deaths | Women | 2019 | 4.8 (3.8 to 5.6) |
| Deaths | Women | 2020 | 4.8 (3.8 to 5.6) |
| Deaths | Women | 2021 | 4.8 (3.9 to 5.6) |
| Deaths | Men | 1990 | 2.7 (1.7 to 3.5) |
| Deaths | Men | 1991 | 2.7 (1.7 to 3.5) |
| Deaths | Men | 1992 | 2.8 (1.8 to 3.5) |
| Deaths | Men | 1993 | 2.8 (1.8 to 3.6) |
| Deaths | Men | 1994 | 2.9 (1.9 to 3.7) |
| Deaths | Men | 1995 | 2.9 (1.9 to 3.7) |
| Deaths | Men | 1996 | 3 (2 to 3.8) |
| Deaths | Men | 1997 | 3 (2 to 3.8) |
| Deaths | Men | 1998 | 3 (2 to 3.8) |
| Deaths | Men | 1999 | 3 (2.1 to 3.8) |
| Deaths | Men | 2000 | 3 (2.1 to 3.8) |
| Deaths | Men | 2001 | 3 (2.1 to 3.7) |
| Deaths | Men | 2002 | 3 (2.2 to 3.7) |
| Deaths | Men | 2003 | 3 (2.2 to 3.7) |
| Deaths | Men | 2004 | 3 (2.2 to 3.7) |
| Deaths | Men | 2005 | 3.1 (2.3 to 3.8) |
| Deaths | Men | 2006 | 3.1 (2.3 to 3.7) |
| Deaths | Men | 2007 | 3.1 (2.3 to 3.7) |
| Deaths | Men | 2008 | 3.1 (2.4 to 3.7) |
| Deaths | Men | 2009 | 3.1 (2.4 to 3.8) |
| Deaths | Men | 2010 | 3.1 (2.4 to 3.8) |
| Deaths | Men | 2011 | 3.1 (2.4 to 3.7) |
| Deaths | Men | 2012 | 3.1 (2.4 to 3.7) |
| Deaths | Men | 2013 | 3.1 (2.4 to 3.7) |
| Deaths | Men | 2014 | 3.1 (2.4 to 3.7) |
| Deaths | Men | 2015 | 3.1 (2.5 to 3.8) |
| Deaths | Men | 2016 | 3.1 (2.5 to 3.8) |
| Deaths | Men | 2017 | 3.2 (2.5 to 3.8) |
| Deaths | Men | 2018 | 3.2 (2.6 to 3.8) |
| Deaths | Men | 2019 | 3.2 (2.6 to 3.9) |
| Deaths | Men | 2020 | 3.2 (2.6 to 3.8) |
| Deaths | Men | 2021 | 3.2 (2.6 to 3.8) |

**Table S2.** All ages number and age‑standardized rate of incidence, prevalence, disability-adjusted life years (DALYs), and deaths of atrial fibrillation and flutter by sex in 2019 and 2021 and overall percent change over 2019–2021 in the Eastern Mediterranean Region countries

| Location | Measure | Age, Metric | Year | | | | | | % Change (2019 to 2021) | | |
| --- | --- | --- | --- | --- | --- | --- | --- | --- | --- | --- | --- |
|  |  |  | 2019 | | | 2021 | | |  |  |  |
|  |  |  | Both | Women | Men | Both | Women | Men | Both | Women | Men |
| Eastern Mediterranean Region | Incidence | Age-standardized | 41.9 (31.1 to 55.9) | 38 (28.2 to 50.9) | 45.5 (33.6 to 61.1) | 41.8 (31 to 56.1) | 37.9 (28.2 to 50.7) | 45.6 (33.7 to 61.2) | -0.1 (-1.3 to 1) | -0.2 (-1.8 to 1.3) | 0.2 (-1.8 to 1.9) |
|  |  | All ages | 158825.5 (122383.9 to 207957.5) | 69112.8 (52970.4 to 91309.6) | 89712.7 (69181 to 117376.9) | 168555 (129706.9 to 220844.2) | 73645.7 (56223.9 to 97185.5) | 94909.3 (73081 to 124276) | 6.1 (4.8 to 7.5) | 6.6 (4.7 to 8.4) | 5.8 (3.6 to 7.8) |
|  | Prevalence | Age-standardized | 438.8 (339.2 to 575.1) | 391.8 (299.7 to 513.5) | 481.6 (371 to 628.2) | 439 (338.8 to 577) | 391.3 (299.9 to 515.5) | 483.3 (372.4 to 632.3) | 0 (-1.2 to 1.1) | -0.1 (-1.8 to 1.6) | 0.4 (-1.6 to 2.1) |
|  |  | All ages | 1533380.6 (1206073.7 to 1995364.7) | 657724.8 (506901.9 to 859369.5) | 875655.8 (688188.2 to 1132802.6) | 1620763.1 (1270893.6 to 2108347.5) | 698891.7 (539905.9 to 917111.1) | 921871.4 (724649.8 to 1194163.1) | 5.7 (4.3 to 6.9) | 6.3 (4.5 to 8) | 5.3 (3.3 to 7) |
|  | DALYs (Disability-Adjusted Life Years) | Age-standardized | 83.3 (68.9 to 98.4) | 89.7 (71.9 to 105.2) | 77.5 (62.2 to 95.3) | 83.4 (68.7 to 99.8) | 89.9 (72.9 to 105.4) | 77.5 (62.6 to 95.2) | 0.1 (-4.1 to 4.6) | 0.1 (-5 to 5.6) | -0.1 (-5.8 to 5.2) |
|  |  | All ages | 266515.8 (217183.7 to 318150.2) | 134489.9 (107486.4 to 160693.6) | 132026 (104366.2 to 162376.5) | 279136.9 (229153.6 to 335913.4) | 141800.5 (115263.1 to 171031.8) | 137336.4 (109551.2 to 170691.1) | 4.7 (0.1 to 9.5) | 5.4 (-0.2 to 11.7) | 4 (-2.1 to 9.5) |
|  | Deaths | Age-standardized | 3.9 (3.2 to 4.6) | 4.8 (3.8 to 5.6) | 3.2 (2.6 to 3.9) | 3.9 (3.2 to 4.5) | 4.8 (3.9 to 5.6) | 3.2 (2.6 to 3.8) | 0.4 (-5.6 to 6.4) | 0.5 (-6.6 to 7.8) | -0.4 (-9.4 to 8.5) |
|  |  | All ages | 10043.4 (8199.3 to 11627.9) | 5731.9 (4604.3 to 6718.3) | 4311.5 (3509.2 to 5196.9) | 10320.6 (8504.4 to 11780.6) | 5967.1 (4845.1 to 6938.7) | 4353.4 (3593.4 to 5204.6) | 2.8 (-3.9 to 10) | 4.1 (-3.7 to 12.4) | 1 (-8.7 to 10.8) |
| Afghanistan | Incidence | Age-standardized | 33.3 (24.6 to 44) | 29.9 (22.1 to 39.7) | 36.7 (26.8 to 48.4) | 33.3 (24.5 to 44.5) | 29.9 (22 to 39.9) | 37 (27.1 to 49.6) | 0.1 (-4.8 to 5.6) | -0.2 (-6.3 to 6.2) | 0.8 (-6.6 to 8.3) |
|  |  | All ages | 2721.8 (2009.3 to 3578.5) | 1280.3 (939.8 to 1680.2) | 1441.5 (1067.1 to 1886.4) | 2735.2 (2055.6 to 3602.5) | 1300.7 (957.6 to 1719.9) | 1434.5 (1078.6 to 1901.7) | 0.5 (-4.8 to 6.9) | 1.6 (-4.7 to 9) | -0.5 (-7.7 to 7) |
|  | Prevalence | Age-standardized | 337.3 (256.1 to 439.3) | 297.7 (224.7 to 391.1) | 376.6 (286 to 486.9) | 338.8 (257.5 to 442.8) | 298.1 (221.2 to 394.7) | 380.9 (289 to 505.4) | 0.4 (-4.6 to 5.7) | 0.1 (-5.8 to 7.5) | 1.1 (-5.3 to 8.3) |
|  |  | All ages | 25586.1 (19659.2 to 32654.9) | 11804.1 (8962.4 to 15208.9) | 13782 (10578.1 to 17500) | 25638.9 (19700.9 to 33184.4) | 11934 (8982.2 to 15588.1) | 13704.9 (10544 to 17473.7) | 0.2 (-4.7 to 5.9) | 1.1 (-4.9 to 8.2) | -0.6 (-6.8 to 7.2) |
|  | DALYs (Disability-Adjusted Life Years) | Age-standardized | 72.3 (51.3 to 97.1) | 73.5 (52.6 to 105.8) | 70.6 (45.8 to 95.5) | 72.1 (51.5 to 95.1) | 73.9 (53.1 to 105.5) | 69.9 (45.7 to 93.9) | -0.3 (-4.6 to 4.8) | 0.5 (-4.7 to 6) | -1 (-7 to 6) |
|  |  | All ages | 5066.3 (3586.1 to 6769.4) | 2686.4 (1919.2 to 3815.9) | 2380 (1562.1 to 3195) | 5074.3 (3663.3 to 6760.9) | 2744 (1928.8 to 3920.2) | 2330.2 (1519.7 to 3157.8) | 0.2 (-4.6 to 5.7) | 2.1 (-4.5 to 9) | -2.1 (-8.6 to 5.9) |
|  | Deaths | Age-standardized | 3.4 (2.1 to 5) | 3.6 (2.3 to 5.8) | 3.1 (1.6 to 4.9) | 3.3 (2 to 4.9) | 3.6 (2.3 to 5.8) | 3 (1.6 to 4.6) | -2.6 (-8.2 to 4.2) | 0 (-5.9 to 6.1) | -5.2 (-12.2 to 3.8) |
|  |  | All ages | 185 (112.5 to 275) | 100.8 (64 to 160.7) | 84.2 (43.6 to 129.7) | 179.3 (110.9 to 263.9) | 101.4 (64.7 to 159.3) | 77.9 (40.7 to 118.1) | -3.1 (-8.4 to 2.8) | 0.6 (-5.5 to 6.8) | -7.5 (-13.2 to 0.1) |
| Bahrain | Incidence | Age-standardized | 36.1 (26.7 to 48.9) | 32.1 (23.4 to 43) | 40 (29.4 to 54.2) | 36.3 (26.6 to 48.2) | 32.2 (23.8 to 42.8) | 40.3 (29.4 to 53.8) | 0.5 (-3.7 to 5.6) | 0.2 (-5.5 to 6.5) | 0.6 (-5.5 to 8.1) |
|  |  | All ages | 262.9 (204.3 to 342) | 94 (70.5 to 124.5) | 168.9 (130.8 to 222.7) | 297 (229 to 382.3) | 104.9 (78.6 to 139.4) | 192.1 (148 to 243.9) | 13 (7.1 to 19.2) | 11.5 (4.4 to 19.8) | 13.8 (5.9 to 22.8) |
|  | Prevalence | Age-standardized | 385.1 (293 to 505.4) | 340.6 (254.8 to 449.8) | 430.2 (330 to 570.1) | 386.7 (293.8 to 505.6) | 341.1 (256.6 to 449.1) | 432.3 (326.7 to 562.5) | 0.4 (-4 to 5) | 0.2 (-6 to 6.4) | 0.5 (-4.8 to 7.3) |
|  |  | All ages | 2342.5 (1797 to 3088.4) | 856.4 (654.3 to 1134.1) | 1486.2 (1122.3 to 1968) | 2667.3 (2055.1 to 3468.5) | 954.7 (735.4 to 1248.1) | 1712.6 (1303.4 to 2213.8) | 13.9 (8.6 to 19.3) | 11.5 (4.4 to 19.1) | 15.2 (8.3 to 23.3) |
|  | DALYs (Disability-Adjusted Life Years) | Age-standardized | 93.6 (58.3 to 120.5) | 109.3 (62.5 to 147.8) | 73.3 (49.4 to 95.9) | 94.9 (57.6 to 122.8) | 111.4 (61.4 to 152.7) | 74.3 (49.4 to 99.3) | 1.4 (-4 to 6.2) | 1.9 (-3.9 to 7.1) | 1.5 (-5.9 to 8.6) |
|  |  | All ages | 390.4 (285.7 to 493.9) | 201.7 (130.8 to 265.7) | 188.7 (137.5 to 241.5) | 442 (315.6 to 569.3) | 226.6 (142.2 to 298.5) | 215.4 (159.5 to 281.1) | 13.2 (5.9 to 21.1) | 12.3 (5.1 to 19.2) | 14.1 (2.7 to 27.8) |
|  | Deaths | Age-standardized | 5.5 (2.6 to 7.6) | 7 (3 to 10.1) | 3.5 (1.9 to 5.3) | 5.7 (2.6 to 7.9) | 7.2 (3 to 10.6) | 3.6 (1.9 to 5.6) | 2.9 (-3.9 to 8.6) | 3.2 (-3.3 to 8.8) | 4.2 (-6.2 to 12) |
|  |  | All ages | 13 (7.7 to 17.4) | 8.9 (4.4 to 12.6) | 4.1 (2.8 to 5.6) | 14.7 (8.1 to 19.9) | 10.1 (4.8 to 14.6) | 4.6 (3.1 to 6.6) | 12.8 (3 to 20.3) | 13.5 (3.8 to 20.8) | 11.2 (-3.5 to 23.6) |
| Djibouti | Incidence | Age-standardized | 37.3 (28.5 to 49.4) | 37 (28.6 to 49.1) | 37.6 (28.7 to 50.2) | 37.4 (28.6 to 49.6) | 37.2 (28.4 to 49.6) | 37.6 (28.7 to 49.3) | 0.2 (-3.5 to 3.9) | 0.5 (-5.8 to 7.2) | -0.1 (-6.5 to 5.7) |
|  |  | All ages | 204.2 (160.6 to 268.6) | 93.6 (73.9 to 121.7) | 110.6 (86.3 to 147.5) | 223.6 (175.4 to 292.7) | 103.5 (81.8 to 136.5) | 120.1 (93.1 to 158.2) | 9.5 (5.6 to 13.5) | 10.6 (4.6 to 17.4) | 8.5 (2 to 14.3) |
|  | Prevalence | Age-standardized | 422.3 (333.4 to 554.9) | 416.9 (327.6 to 545.3) | 430.3 (338.2 to 563.2) | 424.6 (335.8 to 555.3) | 421.1 (331.7 to 557.9) | 431 (335.1 to 562.5) | 0.5 (-3.1 to 4.2) | 1 (-4.6 to 7.9) | 0.2 (-5.8 to 5.5) |
|  |  | All ages | 2017.3 (1590.4 to 2603.7) | 927.8 (721.4 to 1197.3) | 1089.6 (849.9 to 1411.2) | 2212.7 (1710.4 to 2890.5) | 1028.4 (791.2 to 1357.3) | 1184.3 (914.8 to 1539.8) | 9.7 (5.5 to 14.1) | 10.8 (4.4 to 18.1) | 8.7 (2.8 to 14.7) |
|  | DALYs (Disability-Adjusted Life Years) | Age-standardized | 83 (60.8 to 111.4) | 77.5 (52.2 to 111.4) | 89.2 (64.2 to 118.8) | 83.7 (61.7 to 110.9) | 78.6 (54.7 to 113.8) | 89.5 (63.7 to 119.6) | 0.9 (-4.5 to 6.2) | 1.4 (-4.6 to 8.4) | 0.3 (-5.5 to 6.8) |
|  |  | All ages | 336.9 (243.6 to 450.4) | 147.6 (101.1 to 209.9) | 189.3 (135.7 to 255.7) | 368.1 (267.3 to 489.4) | 163 (112.9 to 230.1) | 205.1 (146.9 to 270.6) | 9.2 (3 to 16.1) | 10.4 (2.4 to 19.4) | 8.3 (0.4 to 17) |
|  | Deaths | Age-standardized | 3.7 (2.4 to 5.4) | 3.4 (2 to 5.5) | 4.2 (2.7 to 6) | 3.8 (2.5 to 5.5) | 3.5 (2 to 5.7) | 4.2 (2.7 to 6.1) | 1.2 (-6 to 8.8) | 2.1 (-6.3 to 10.7) | 0.2 (-8.3 to 9.6) |
|  |  | All ages | 9.7 (6.2 to 13.9) | 4.4 (2.5 to 7.2) | 5.2 (3.3 to 7.7) | 10.5 (6.8 to 15.1) | 4.9 (2.8 to 8) | 5.6 (3.6 to 8.3) | 8.2 (0.1 to 16.7) | 9.4 (-0.2 to 19) | 7.1 (-1.6 to 16.9) |
| Egypt | Incidence | Age-standardized | 36.7 (26.6 to 49.3) | 32.2 (23.7 to 42.8) | 40 (28.9 to 53.6) | 36.9 (27.1 to 49.4) | 32.4 (24 to 43.5) | 40.3 (29.4 to 53.9) | 0.7 (-3.7 to 5.4) | 0.8 (-5.9 to 7.6) | 0.7 (-4.9 to 6.9) |
|  |  | All ages | 18478.3 (13880 to 24195.2) | 7516.7 (5551 to 9974.4) | 10961.6 (8168.2 to 14315.4) | 19786.2 (14849 to 25927.2) | 8095.2 (5906 to 10812.7) | 11691 (8726.6 to 15465.4) | 7.1 (1.9 to 12.2) | 7.7 (-0.8 to 15.9) | 6.7 (0 to 13.1) |
|  | Prevalence | Age-standardized | 389.8 (295.6 to 507.8) | 334.1 (250.5 to 432.8) | 423.7 (321.4 to 555.2) | 393 (297.7 to 514.2) | 336.8 (251.4 to 438.2) | 427.3 (322.5 to 564.1) | 0.8 (-3.5 to 6) | 0.8 (-6.3 to 8.3) | 0.9 (-5.1 to 7) |
|  |  | All ages | 172722.7 (133992.3 to 222543.9) | 67590.7 (51260.8 to 89771.6) | 105132.1 (81685.7 to 135707.9) | 184696.9 (143378.1 to 239672.1) | 72748.6 (54793.2 to 95018.5) | 111948.3 (87183.8 to 146087) | 6.9 (2.1 to 13) | 7.6 (0.7 to 16.7) | 6.5 (-0.1 to 13.6) |
|  | DALYs (Disability-Adjusted Life Years) | Age-standardized | 75.6 (62.7 to 91.1) | 104.1 (82.2 to 121.3) | 67.9 (54.8 to 84.1) | 75.9 (62.4 to 91.2) | 100 (80 to 116) | 68.6 (55.1 to 86) | 0.3 (-8.3 to 8.8) | -3.9 (-11.8 to 3.8) | 1.1 (-8.2 to 10.3) |
|  |  | All ages | 28495.4 (23348.8 to 34983.5) | 13058.4 (10664 to 15574.7) | 15437 (12347.8 to 19417.2) | 30353.3 (24425.2 to 37293) | 13938.9 (11213.4 to 17009.4) | 16414.4 (12876.7 to 20540.4) | 6.5 (-3.2 to 16.6) | 6.7 (-4.3 to 18.2) | 6.3 (-4.6 to 16.9) |
|  | Deaths | Age-standardized | 3.6 (3 to 4.1) | 6.9 (5.3 to 8) | 2.7 (2.2 to 3.2) | 3.6 (2.9 to 4.3) | 6.4 (4.8 to 7.5) | 2.8 (2.2 to 3.4) | -0.6 (-11.5 to 11.4) | -7.6 (-15.1 to 1.1) | 1.8 (-10.9 to 16.6) |
|  |  | All ages | 924.9 (802 to 1017.5) | 465.1 (382.7 to 525.6) | 459.8 (386.7 to 527.7) | 971.5 (799.2 to 1144.7) | 486.3 (389.3 to 575.3) | 485.2 (383 to 596.2) | 5 (-8.1 to 19.9) | 4.6 (-8.6 to 18.6) | 5.5 (-8.9 to 22) |
| Iran (Islamic Republic of) | Incidence | Age-standardized | 40.5 (30.1 to 54.1) | 37.4 (27.8 to 49.6) | 43.5 (32.4 to 58.2) | 40.6 (30 to 54.4) | 37.4 (27.7 to 50.2) | 43.7 (32.3 to 58.7) | 0.2 (-1.1 to 1.6) | 0.1 (-1.6 to 1.8) | 0.5 (-1.3 to 2.2) |
|  |  | All ages | 27685.4 (21235 to 36313.8) | 12746.5 (9751.9 to 16787.2) | 14938.9 (11441.1 to 19762.5) | 29418.3 (22665.1 to 38723.1) | 13622.3 (10363.4 to 18155.8) | 15796 (12121.5 to 20711) | 6.3 (4.5 to 8) | 6.9 (5 to 8.7) | 5.7 (3.4 to 8) |
|  | Prevalence | Age-standardized | 424.5 (325.8 to 555.4) | 387.4 (297.1 to 506.4) | 459.1 (350.5 to 599.9) | 425.4 (327.2 to 559.2) | 387.8 (298 to 510.7) | 461.1 (354.7 to 601.6) | 0.2 (-1.2 to 1.6) | 0.1 (-1.5 to 1.7) | 0.5 (-1.3 to 2.2) |
|  |  | All ages | 278770.9 (216848.8 to 360907.9) | 125967.5 (97128.5 to 164514.5) | 152803.4 (117891.9 to 197505.6) | 294248.7 (230024.2 to 382165.9) | 134056.9 (103750.2 to 176465.5) | 160191.8 (124346.3 to 206830.5) | 5.6 (4 to 7) | 6.4 (4.8 to 8.1) | 4.8 (2.7 to 6.9) |
|  | DALYs (Disability-Adjusted Life Years) | Age-standardized | 74.7 (59.1 to 91) | 86.2 (67.7 to 101.4) | 64.5 (49.7 to 81.9) | 72.4 (57.4 to 88.3) | 83.5 (65.3 to 99.2) | 62.5 (47.4 to 80.3) | -3 (-5.9 to -0.1) | -3.1 (-7 to 0.7) | -3.1 (-6.6 to 0) |
|  |  | All ages | 46952.6 (37082.5 to 57309.6) | 25858.2 (20240.6 to 30539.4) | 21094.5 (16143.2 to 26593.5) | 47524.7 (37527.7 to 58270.9) | 26310.9 (20508.1 to 31274.3) | 21213.7 (16083.8 to 27193.4) | 1.2 (-1.8 to 4.2) | 1.8 (-2.1 to 6) | 0.6 (-3 to 3.8) |
|  | Deaths | Age-standardized | 3.5 (2.6 to 3.9) | 4.8 (3.5 to 5.4) | 2.4 (1.6 to 2.8) | 3.3 (2.5 to 3.8) | 4.6 (3.3 to 5.3) | 2.2 (1.5 to 2.7) | -5.2 (-9.8 to -0.5) | -4.1 (-9.6 to 1.1) | -7.4 (-14.1 to -1.2) |
|  |  | All ages | 2004.4 (1498.6 to 2250.6) | 1270.1 (940.8 to 1436.8) | 734.2 (505.4 to 847.2) | 1948.9 (1462.6 to 2233.1) | 1258.4 (920 to 1445.6) | 690.6 (478.6 to 823.5) | -2.8 (-7.6 to 2) | -0.9 (-6.7 to 4.7) | -5.9 (-12.6 to 0.4) |
| Iraq | Incidence | Age-standardized | 38.1 (28.5 to 50.6) | 34.2 (25.3 to 45) | 42.6 (31.4 to 56.4) | 38.4 (28.1 to 51.2) | 34.4 (25.2 to 45.8) | 42.9 (31.2 to 58.3) | 0.6 (-4.1 to 5.2) | 0.7 (-5.5 to 7.4) | 0.7 (-5.9 to 8.6) |
|  |  | All ages | 7654.4 (5821.9 to 10005.9) | 3507.1 (2672.2 to 4568.9) | 4147.3 (3142.7 to 5414) | 8150.8 (6210.3 to 10702.5) | 3761.8 (2831.3 to 4947.6) | 4389 (3338.6 to 5758.3) | 6.5 (2 to 11) | 7.3 (0.7 to 14.6) | 5.8 (-0.6 to 12.8) |
|  | Prevalence | Age-standardized | 403.3 (304.8 to 526.5) | 359.1 (271.3 to 470.6) | 455.1 (344.6 to 593.9) | 406.1 (308.5 to 532) | 361.6 (273.1 to 471.7) | 459.7 (349.9 to 600.7) | 0.7 (-3.3 to 5) | 0.7 (-4.9 to 7.5) | 1 (-5.3 to 7.8) |
|  |  | All ages | 74370.4 (57634.1 to 96238) | 34267.8 (26214.1 to 44913.1) | 40102.6 (30791.7 to 52260.1) | 78700.6 (61203.2 to 102059.9) | 36513.9 (28054.3 to 47836) | 42186.8 (32671 to 54683.5) | 5.8 (2 to 10.8) | 6.6 (0.7 to 13.7) | 5.2 (-1.4 to 12.2) |
|  | DALYs (Disability-Adjusted Life Years) | Age-standardized | 87.4 (70.5 to 106) | 90.5 (72.6 to 112.5) | 83 (64.4 to 106.1) | 93.6 (74.2 to 114) | 97.1 (78 to 120.1) | 88.2 (67.2 to 113.4) | 7.1 (-1.7 to 14.8) | 7.3 (-1.6 to 15.4) | 6.3 (-3.5 to 17.1) |
|  |  | All ages | 15145.7 (12164.8 to 18371) | 8187.6 (6548 to 10156.6) | 6958.1 (5409.1 to 8595.1) | 16445.3 (13020.4 to 19937.7) | 8974.6 (7110.4 to 11078.1) | 7470.7 (5779 to 9283.1) | 8.6 (1.3 to 16.2) | 9.6 (1.6 to 18.1) | 7.4 (-1.1 to 18.1) |
|  | Deaths | Age-standardized | 4.3 (3.3 to 5.4) | 4.8 (3.6 to 6.2) | 3.6 (2.7 to 5.1) | 4.9 (3.5 to 6.2) | 5.4 (3.9 to 7) | 4 (2.8 to 5.7) | 13.7 (-0.5 to 24.8) | 13.3 (-0.6 to 23.4) | 12.5 (-5.2 to 28.7) |
|  |  | All ages | 587.2 (453.1 to 747.9) | 365.9 (276.9 to 471.2) | 221.3 (166.5 to 301.2) | 647.1 (481.2 to 825.7) | 407.8 (299.6 to 522.8) | 239.2 (172.9 to 325.5) | 10.2 (-1.6 to 20.4) | 11.5 (-0.8 to 21.1) | 8.1 (-5.9 to 22.9) |
| Jordan | Incidence | Age-standardized | 36.9 (27.5 to 49.3) | 32.7 (24.4 to 43.5) | 40.7 (30.2 to 54.7) | 37 (27.5 to 49.7) | 32.8 (24.3 to 44) | 41 (30.2 to 54.7) | 0.4 (-4.2 to 5.3) | 0.2 (-6.3 to 7.6) | 0.7 (-6.1 to 7.4) |
|  |  | All ages | 2157.9 (1638.8 to 2821.3) | 910.1 (683.7 to 1188.6) | 1247.8 (943.3 to 1653.1) | 2471.6 (1878 to 3282.5) | 1042.8 (793.1 to 1394) | 1428.8 (1086.8 to 1852.4) | 14.5 (9.4 to 19.8) | 14.6 (7.4 to 23) | 14.5 (7.2 to 21.7) |
|  | Prevalence | Age-standardized | 395 (301.1 to 514.5) | 346 (262.8 to 448.4) | 436.5 (329.9 to 574.5) | 396.6 (300.6 to 520.3) | 346.8 (263.6 to 458.2) | 440 (331.9 to 574.6) | 0.4 (-4 to 5.1) | 0.2 (-6 to 7.4) | 0.8 (-5.4 to 7.2) |
|  |  | All ages | 20915.2 (16198.7 to 27058) | 8726 (6637.3 to 11360.8) | 12189.2 (9411.9 to 15794.9) | 23983 (18450.5 to 31112) | 10018.2 (7633.2 to 13133.4) | 13964.8 (10761 to 18087.1) | 14.7 (9.6 to 20) | 14.8 (6.4 to 23.3) | 14.6 (7.2 to 22.2) |
|  | DALYs (Disability-Adjusted Life Years) | Age-standardized | 61.5 (48.4 to 76.5) | 69.5 (55.3 to 85.8) | 56.7 (43.4 to 72.2) | 63.1 (49.4 to 78.3) | 71.2 (56.4 to 87.4) | 57.6 (43.6 to 73.9) | 2.6 (-2.5 to 7.8) | 2.4 (-4.6 to 9.2) | 1.6 (-5 to 8.6) |
|  |  | All ages | 2905.8 (2223.6 to 3696.4) | 1414.7 (1110.4 to 1765.7) | 1491.1 (1129.4 to 1954.4) | 3377 (2602.3 to 4222.8) | 1663.7 (1312 to 2050.5) | 1713.4 (1286.7 to 2240.3) | 16.2 (9.3 to 23) | 17.6 (8.9 to 27.1) | 14.9 (5.7 to 24.8) |
|  | Deaths | Age-standardized | 2.6 (2 to 3.1) | 3.8 (2.9 to 4.7) | 1.9 (1.4 to 2.3) | 2.7 (2.1 to 3.3) | 3.9 (2.9 to 4.8) | 1.9 (1.5 to 2.5) | 4.9 (-2.2 to 12.6) | 3.7 (-5.1 to 12.4) | 2.6 (-5.6 to 12.6) |
|  |  | All ages | 86.5 (69 to 102.2) | 50.5 (39.6 to 62.4) | 35.9 (27.1 to 44.4) | 102.5 (80.5 to 125.4) | 61.5 (47.1 to 76.7) | 41 (31.6 to 52.5) | 18.6 (10.2 to 27.7) | 21.8 (11 to 33.1) | 14 (4 to 25.8) |
| Kuwait | Incidence | Age-standardized | 37.8 (27.7 to 50) | 32.9 (24.3 to 43.7) | 41 (29.9 to 54.3) | 37.9 (27.9 to 50.9) | 33.1 (24.4 to 44) | 41.3 (30.4 to 55.5) | 0.3 (-5.2 to 5.4) | 0.6 (-5 to 7) | 0.8 (-6.5 to 8) |
|  |  | All ages | 898.9 (709.4 to 1148.1) | 321.6 (253.5 to 408.5) | 577.3 (449.7 to 739.3) | 1013.2 (804.2 to 1301.5) | 376.2 (294.2 to 484.6) | 637.1 (500.9 to 825.4) | 12.7 (7.4 to 18.4) | 17 (10.7 to 23.8) | 10.3 (3 to 17.7) |
|  | Prevalence | Age-standardized | 406.2 (308.4 to 529.9) | 351.7 (267.5 to 459.9) | 443.6 (337.9 to 577.5) | 408.1 (310.6 to 530.2) | 354.1 (268 to 463.7) | 447.3 (341.6 to 586.7) | 0.5 (-4.5 to 5.2) | 0.7 (-5.5 to 6.6) | 0.8 (-5.9 to 7.4) |
|  |  | All ages | 8752.2 (6857.8 to 11160.9) | 3032.9 (2354.8 to 3898.9) | 5719.3 (4502.6 to 7350.2) | 9835.5 (7696.8 to 12638) | 3527.2 (2755.1 to 4533.9) | 6308.4 (4952.4 to 8095.6) | 12.4 (7.5 to 16.9) | 16.3 (9.2 to 22.9) | 10.3 (3.9 to 16) |
|  | DALYs (Disability-Adjusted Life Years) | Age-standardized | 64.7 (52.2 to 80.1) | 59.2 (47.7 to 73) | 68.7 (55.3 to 84.6) | 66.6 (52.8 to 82.2) | 60.5 (48.2 to 74) | 70.9 (55.9 to 89.2) | 2.9 (-5.3 to 11.3) | 2.3 (-5.5 to 9.2) | 3.3 (-6.8 to 14.1) |
|  |  | All ages | 1326.6 (1077.6 to 1625.4) | 487.8 (394.1 to 603.5) | 838.8 (680.1 to 1044) | 1524.7 (1212.5 to 1889) | 580.6 (460.2 to 700.2) | 944.1 (744.7 to 1187.3) | 14.9 (5.7 to 25.5) | 19 (8.5 to 28.5) | 12.6 (0.8 to 26.8) |
|  | Deaths | Age-standardized | 2.9 (2.3 to 3.2) | 2.7 (2.1 to 3.2) | 3 (2.5 to 3.3) | 3 (2.4 to 3.6) | 2.9 (2.1 to 3.3) | 3.1 (2.4 to 3.8) | 4.6 (-7.5 to 19.2) | 4.6 (-6.1 to 16.5) | 4.3 (-10.5 to 21.4) |
|  |  | All ages | 51.3 (40.9 to 56.8) | 20.4 (15.4 to 24) | 30.9 (25.8 to 34) | 60.3 (48.1 to 71.8) | 25.2 (18.8 to 29.8) | 35.1 (27.7 to 42.6) | 17.6 (4.1 to 34.2) | 23.3 (10.3 to 37.4) | 13.9 (-2.4 to 32.6) |
| Lebanon | Incidence | Age-standardized | 36.2 (26.9 to 48.2) | 32.3 (24 to 43) | 40.5 (30 to 53.8) | 36.2 (27 to 48.3) | 32.4 (24.2 to 42.9) | 40.7 (30.1 to 54.2) | 0.2 (-3.7 to 4.5) | 0.3 (-6.1 to 7.2) | 0.5 (-4.9 to 7.3) |
|  |  | All ages | 2159.4 (1581.6 to 2918) | 1021.8 (751.8 to 1372.6) | 1137.6 (827.3 to 1525.2) | 2243.6 (1665.5 to 3000.9) | 1080.2 (803.4 to 1433.9) | 1163.4 (853.4 to 1553.7) | 3.9 (-0.3 to 8.6) | 5.7 (-1 to 13) | 2.3 (-3.5 to 9.5) |
|  | Prevalence | Age-standardized | 386.2 (294.3 to 508.8) | 343.2 (258.6 to 450.2) | 435 (334 to 567.8) | 387.1 (298.5 to 511.1) | 343.9 (262.1 to 454.8) | 438 (336 to 576.7) | 0.2 (-3.7 to 4.2) | 0.2 (-6.1 to 7.1) | 0.7 (-4.8 to 7) |
|  |  | All ages | 23659.8 (17961 to 31005.9) | 11135.8 (8355.7 to 14700.4) | 12524 (9500.4 to 16362.7) | 24591.1 (18725.1 to 32515.7) | 11779.5 (8922.9 to 15562.5) | 12811.5 (9737.4 to 16956.4) | 3.9 (-0.1 to 8.1) | 5.8 (-0.8 to 13.2) | 2.3 (-3.5 to 8.9) |
|  | DALYs (Disability-Adjusted Life Years) | Age-standardized | 68.8 (55.3 to 85.4) | 69.3 (56.2 to 86.2) | 68.1 (53.3 to 86.2) | 68.9 (56.3 to 84.2) | 69.1 (55.6 to 85.5) | 68.5 (53.5 to 86.9) | 0.2 (-4.4 to 5.5) | -0.2 (-6.3 to 5.7) | 0.5 (-5.2 to 7.4) |
|  |  | All ages | 4347.8 (3513.1 to 5374.3) | 2347.4 (1899.6 to 2937.1) | 2000.4 (1570 to 2563.4) | 4546.2 (3720.4 to 5509.3) | 2496.6 (2020.5 to 3098.9) | 2049.6 (1599.9 to 2597.7) | 4.6 (-0.2 to 10.1) | 6.4 (0 to 12.1) | 2.5 (-3.3 to 9.3) |
|  | Deaths | Age-standardized | 3.4 (2.8 to 4.3) | 3.7 (2.9 to 4.6) | 3 (2.4 to 4.6) | 3.4 (2.8 to 4.4) | 3.7 (2.9 to 4.6) | 3 (2.3 to 4.5) | 0.3 (-5.5 to 7.1) | -0.4 (-6.7 to 6.4) | 0.7 (-6.7 to 8.4) |
|  |  | All ages | 226 (184 to 289) | 134.3 (105.6 to 167) | 91.6 (71.8 to 139.1) | 238.9 (192.7 to 307.8) | 144.3 (112.2 to 181) | 94.6 (74.2 to 141.3) | 5.7 (-0.5 to 12.9) | 7.4 (0.7 to 14.6) | 3.3 (-4.4 to 11.6) |
| Libya | Incidence | Age-standardized | 37.6 (27.4 to 50.9) | 33.3 (24.6 to 44.8) | 41.6 (30.4 to 56.1) | 37.7 (27.8 to 50.7) | 33.5 (24.9 to 45.1) | 41.8 (30.4 to 55.7) | 0.4 (-3.4 to 4.8) | 0.5 (-6.2 to 7.3) | 0.5 (-4.9 to 7.3) |
|  |  | All ages | 1665.4 (1275 to 2196.4) | 726.7 (554.2 to 956.6) | 938.8 (720.5 to 1263.1) | 1776.9 (1361.2 to 2315.1) | 785.8 (604.1 to 1029) | 991 (755.1 to 1298) | 6.7 (2.7 to 11.3) | 8.1 (1.3 to 14.3) | 5.6 (0.1 to 11.8) |
|  | Prevalence | Age-standardized | 397.4 (304.7 to 519.7) | 350.2 (264.9 to 460) | 444.2 (337.6 to 582.7) | 398.3 (304.8 to 518.5) | 350.4 (264.3 to 459.2) | 446.6 (341.9 to 577.1) | 0.2 (-3.7 to 4.9) | 0.1 (-6.5 to 6.4) | 0.5 (-5 to 6.2) |
|  |  | All ages | 16544.8 (12779.1 to 21345) | 7152 (5503.8 to 9371.6) | 9392.8 (7269.6 to 12022.8) | 17554.5 (13696.2 to 22623.3) | 7662.3 (5873.7 to 9986.9) | 9892.2 (7702.9 to 12632) | 6.1 (2.1 to 10.8) | 7.1 (0.4 to 13.6) | 5.3 (-0.5 to 11.4) |
|  | DALYs (Disability-Adjusted Life Years) | Age-standardized | 70.4 (50.5 to 92.7) | 80.2 (53.4 to 110.6) | 60.4 (44.6 to 78.8) | 70.1 (50.4 to 92.3) | 79.5 (53 to 110.3) | 60.2 (44.2 to 77.9) | -0.5 (-5.7 to 5) | -0.9 (-6.7 to 5.6) | -0.4 (-6.5 to 6.4) |
|  |  | All ages | 2812.1 (2028.3 to 3687.9) | 1590.7 (1055.5 to 2196.6) | 1221.4 (898.3 to 1604.3) | 2949.8 (2127.2 to 3911.1) | 1678.3 (1115.1 to 2327.7) | 1271.4 (923.6 to 1642.9) | 4.9 (-1 to 10.9) | 5.5 (-0.9 to 12.7) | 4.1 (-3.5 to 11.7) |
|  | Deaths | Age-standardized | 3.1 (2 to 4.5) | 4 (2.3 to 6.3) | 2.1 (1.5 to 3.2) | 3.1 (1.9 to 4.5) | 4 (2.2 to 6.2) | 2.1 (1.4 to 3.2) | -1.2 (-8.3 to 5.6) | -1.4 (-8.7 to 5.9) | -1.6 (-10.6 to 6.6) |
|  |  | All ages | 108.6 (69.4 to 157.1) | 72.6 (41 to 113.8) | 36 (25 to 53.8) | 111.3 (68.6 to 163.2) | 74.9 (42 to 116.3) | 36.4 (24 to 55.6) | 2.5 (-4.8 to 9.5) | 3.2 (-4.6 to 10.9) | 1 (-8 to 9.5) |
| Morocco | Incidence | Age-standardized | 36.5 (26.9 to 49.3) | 32.8 (24 to 43.8) | 40.4 (29.7 to 54.3) | 36.6 (26.9 to 49.6) | 32.6 (24.1 to 43.5) | 40.9 (29.9 to 56.6) | 0.3 (-3.9 to 4.8) | -0.4 (-6.8 to 5.2) | 1 (-5.1 to 7.6) |
|  |  | All ages | 11152.8 (8375 to 14998.9) | 5092.9 (3793.7 to 6811.6) | 6059.9 (4563.2 to 8115.7) | 11737.8 (8719.8 to 15855.7) | 5343.7 (4017.5 to 7082.9) | 6394 (4707.8 to 8692.9) | 5.2 (0.6 to 10.3) | 4.9 (-1.5 to 10.8) | 5.5 (-1 to 13.2) |
|  | Prevalence | Age-standardized | 381.5 (292.1 to 502.2) | 339 (253.8 to 447.5) | 427 (327.2 to 560.5) | 383.3 (292.5 to 506) | 338.4 (257.6 to 443.5) | 432.4 (329.3 to 571.9) | 0.5 (-3.7 to 4.6) | -0.2 (-6.2 to 5.4) | 1.3 (-4.9 to 8) |
|  |  | All ages | 111547.2 (86053.6 to 145408.1) | 50751.3 (38221.4 to 67121.6) | 60795.9 (47090 to 79222.7) | 117079.3 (89915.8 to 151799.8) | 53213.3 (40503.1 to 69685) | 63866 (49170.4 to 83435.9) | 5 (0.6 to 9.5) | 4.9 (-1.6 to 10.5) | 5 (-1.1 to 12.2) |
|  | DALYs (Disability-Adjusted Life Years) | Age-standardized | 83.2 (64.6 to 104) | 89.4 (65.3 to 117.8) | 76.6 (59.2 to 96.2) | 83.6 (65.6 to 104.3) | 89 (66.1 to 118.4) | 77.7 (59.3 to 97.7) | 0.4 (-4.9 to 7.2) | -0.4 (-6 to 6.9) | 1.4 (-4.5 to 9) |
|  |  | All ages | 22442.5 (17440.6 to 28454) | 12369.7 (9098.3 to 16307.4) | 10072.8 (7758.7 to 12660) | 23354.5 (18286.7 to 29438.3) | 12872 (9639.2 to 17118.3) | 10482.5 (8060.8 to 13252.3) | 4.1 (-1.2 to 11.1) | 4.1 (-2 to 11.8) | 4.1 (-1.9 to 11.9) |
|  | Deaths | Age-standardized | 4.2 (3 to 5.5) | 4.9 (3.3 to 6.9) | 3.5 (2.5 to 4.6) | 4.3 (3 to 5.5) | 4.9 (3.3 to 6.9) | 3.6 (2.6 to 4.6) | 0.9 (-5.8 to 10.8) | -0.2 (-6.8 to 8.9) | 2.2 (-6.3 to 16.7) |
|  |  | All ages | 961.6 (698.8 to 1250.7) | 583.6 (393.2 to 821.5) | 378 (268.7 to 491.5) | 991.4 (711.3 to 1279.9) | 603.1 (410.5 to 866) | 388.3 (289.3 to 495.4) | 3.1 (-3.7 to 13) | 3.3 (-3.8 to 13) | 2.7 (-6 to 16.8) |
| Oman | Incidence | Age-standardized | 34.4 (25.2 to 45.9) | 30.6 (22.4 to 41.4) | 38.1 (27.9 to 50.3) | 34.4 (25.3 to 46.3) | 30.7 (22.6 to 41.5) | 38.2 (28 to 51.1) | 0.1 (-4.3 to 4.5) | 0.3 (-5.7 to 7.6) | 0.2 (-6.1 to 7.7) |
|  |  | All ages | 563.9 (438 to 719.3) | 216 (165.4 to 283.6) | 347.9 (275.1 to 444.4) | 643.4 (503.6 to 828.9) | 243.4 (182.9 to 319.8) | 400.1 (310.3 to 521.8) | 14.1 (8.7 to 19.2) | 12.7 (5.8 to 20.3) | 15 (7.7 to 22.1) |
|  | Prevalence | Age-standardized | 364.2 (275.6 to 474.2) | 323.3 (244.3 to 424.1) | 408.4 (311.3 to 529.1) | 365.3 (276.4 to 482.9) | 324.6 (244.4 to 430.1) | 409.8 (311.2 to 536.7) | 0.3 (-4.4 to 4.7) | 0.4 (-5.7 to 7.3) | 0.4 (-5.6 to 7.5) |
|  |  | All ages | 5133.2 (4013.6 to 6574.4) | 2054.7 (1572.5 to 2647.9) | 3078.5 (2370.3 to 3978.4) | 5827.2 (4557.2 to 7636.5) | 2310.9 (1756.4 to 3014.4) | 3516.3 (2743.5 to 4648.9) | 13.5 (8.6 to 19) | 12.5 (5.5 to 19.9) | 14.2 (7.1 to 21.8) |
|  | DALYs (Disability-Adjusted Life Years) | Age-standardized | 93.4 (76.9 to 109.5) | 91.8 (72.6 to 110) | 100.1 (82.5 to 119.9) | 91 (74.6 to 109.2) | 90.3 (71.1 to 110.3) | 94.3 (76.7 to 114) | -2.6 (-8.2 to 3.9) | -1.6 (-8.4 to 6.3) | -5.8 (-11.1 to -0.1) |
|  |  | All ages | 1148.9 (952.6 to 1356.4) | 546.4 (440.3 to 647) | 602.5 (483 to 744.5) | 1236.6 (991.4 to 1517.6) | 590.8 (467.1 to 711.4) | 645.8 (491.2 to 854.2) | 7.6 (-1.3 to 17.4) | 8.1 (-0.2 to 18.1) | 7.2 (-5 to 21.6) |
|  | Deaths | Age-standardized | 4.9 (3.8 to 5.9) | 4.8 (3.5 to 6) | 5.5 (4.1 to 6.9) | 4.7 (3.6 to 5.9) | 4.8 (3.5 to 6.1) | 5 (3.5 to 6.6) | -3.1 (-10.9 to 3.7) | -0.7 (-8.7 to 7.8) | -9.8 (-22.1 to -0.7) |
|  |  | All ages | 43.9 (35.5 to 51.8) | 24.2 (18 to 29.9) | 19.7 (15.8 to 23.9) | 45.3 (35.8 to 55.2) | 25.8 (19.6 to 32.4) | 19.6 (15.4 to 24.5) | 3.2 (-5.5 to 12.7) | 6.3 (-2.4 to 16.1) | -0.6 (-10.2 to 12) |
| Pakistan | Incidence | Age-standardized | 54.1 (40.6 to 72.7) | 50.2 (37.7 to 67.6) | 57.6 (43.1 to 76.9) | 53.8 (40.3 to 71.6) | 49.8 (37.3 to 66.2) | 57.5 (43 to 76.3) | -0.5 (-3.1 to 2.3) | -0.8 (-4.4 to 3.4) | -0.1 (-4.1 to 3.5) |
|  |  | All ages | 55214.9 (42403 to 72252.4) | 24083.1 (18385.6 to 31755) | 31131.9 (23888.5 to 40910.3) | 57873.9 (44288.7 to 75860.6) | 25397.2 (19516.2 to 33388.4) | 32476.8 (24823.1 to 42728) | 4.8 (2.3 to 7.5) | 5.5 (2.2 to 9.5) | 4.3 (0.4 to 8.3) |
|  | Prevalence | Age-standardized | 561 (428.1 to 733.3) | 507.1 (385.7 to 668) | 608.6 (465.3 to 794.2) | 558.6 (429.5 to 735.7) | 503.7 (386.1 to 664.7) | 608.5 (467.5 to 801.8) | -0.4 (-2.9 to 2.1) | -0.7 (-3.9 to 3.5) | 0 (-3.7 to 4.3) |
|  |  | All ages | 525365.9 (405730.6 to 690821.4) | 224389.8 (172829.1 to 294990.8) | 300976.1 (232981.2 to 397208.8) | 548173.6 (427140.9 to 726636.8) | 236096.7 (183830.9 to 313314.4) | 312076.9 (242287.9 to 413910) | 4.3 (1.8 to 7.2) | 5.2 (1.7 to 9.6) | 3.7 (-0.3 to 7.9) |
|  | DALYs (Disability-Adjusted Life Years) | Age-standardized | 100.8 (79.1 to 128.6) | 102.4 (77.1 to 139) | 99.5 (72.5 to 126.8) | 101.5 (79.7 to 131.7) | 103.4 (77.3 to 140.8) | 99.8 (73.5 to 129.1) | 0.7 (-11 to 14.1) | 1 (-14.8 to 20.7) | 0.3 (-14.1 to 16.5) |
|  |  | All ages | 86646.7 (68413.7 to 111666.7) | 40888.6 (30625.4 to 55535.4) | 45758 (33757.3 to 58341) | 90387.2 (69208.3 to 116432.2) | 43286.7 (32096.3 to 59689.4) | 47100.6 (34616.1 to 61263.2) | 4.3 (-7.9 to 17.5) | 5.9 (-11 to 26) | 2.9 (-12.4 to 20) |
|  | Deaths | Age-standardized | 4.5 (3.3 to 6.2) | 5 (3.4 to 7.3) | 4.1 (2.6 to 5.9) | 4.6 (3.3 to 6.4) | 5 (3.5 to 7.6) | 4.2 (2.7 to 5.9) | 1.5 (-15.8 to 22.3) | 2 (-19.6 to 30.8) | 0.7 (-21.7 to 29.5) |
|  |  | All ages | 3037.5 (2220.5 to 4198.9) | 1570 (1079.1 to 2326.2) | 1467.5 (919.7 to 2079.4) | 3114.8 (2310.5 to 4332.2) | 1649.7 (1146.7 to 2527.5) | 1465.1 (941.5 to 2053.4) | 2.5 (-16.5 to 25.5) | 5.1 (-18.4 to 36.9) | -0.2 (-24.8 to 31.7) |
| Palestine | Incidence | Age-standardized | 32.9 (24 to 44) | 29.8 (21.9 to 39.8) | 37 (27 to 49.8) | 33.2 (24.4 to 44.7) | 29.9 (22 to 39.7) | 37.4 (27.5 to 50.3) | 0.8 (-4.3 to 5.8) | 0.4 (-7.4 to 6.6) | 1 (-6.6 to 8.4) |
|  |  | All ages | 686.2 (524 to 891.5) | 328 (250.4 to 428.9) | 358.2 (269.4 to 463.5) | 748.6 (570.4 to 981.6) | 353.7 (270.6 to 469.6) | 394.9 (299.4 to 522.5) | 9.1 (3.6 to 14.6) | 7.9 (-0.1 to 14.3) | 10.2 (2.8 to 18.5) |
|  | Prevalence | Age-standardized | 343.8 (260.8 to 444) | 310.1 (234.2 to 402.8) | 392.1 (295.7 to 506.5) | 347.4 (265.2 to 454) | 311.9 (237.7 to 411.2) | 397.3 (303.5 to 526.2) | 1.1 (-3.8 to 6.1) | 0.6 (-6.1 to 6.4) | 1.3 (-5.5 to 8.7) |
|  |  | All ages | 6602.3 (5128.5 to 8477.6) | 3198.3 (2464.2 to 4168.3) | 3404 (2630.3 to 4308.8) | 7196.7 (5524.6 to 9404.9) | 3443.1 (2642.1 to 4502.7) | 3753.6 (2873.5 to 4911.3) | 9 (4 to 14.4) | 7.7 (0.4 to 14.1) | 10.3 (2.6 to 18.9) |
|  | DALYs (Disability-Adjusted Life Years) | Age-standardized | 87.7 (73.5 to 102.6) | 88.9 (73.4 to 105) | 90 (71.6 to 114.3) | 89.8 (74.3 to 107.6) | 90.3 (73.8 to 109.9) | 93.2 (69.8 to 125.2) | 2.3 (-2.4 to 7.5) | 1.6 (-4.2 to 8) | 3.5 (-5.3 to 14.6) |
|  |  | All ages | 1395.8 (1154.3 to 1664.1) | 812.2 (668.2 to 959.4) | 583.7 (463.7 to 730.4) | 1522.4 (1248.7 to 1837.6) | 877 (717.7 to 1063.3) | 645.4 (503.3 to 817.5) | 9.1 (3.4 to 15) | 8 (1.4 to 15.1) | 10.6 (2.4 to 19.2) |
|  | Deaths | Age-standardized | 5.2 (4.2 to 6.3) | 5.3 (4.3 to 6.4) | 5.3 (3.9 to 7.3) | 5.4 (4.3 to 6.7) | 5.5 (4.4 to 6.7) | 5.6 (3.8 to 8.6) | 3.9 (-2.6 to 10.5) | 2.7 (-4 to 10.7) | 6 (-7.7 to 23) |
|  |  | All ages | 61.8 (51.5 to 72.8) | 41.2 (33.7 to 49.1) | 20.6 (15.7 to 26.8) | 68.1 (55.2 to 82) | 44.9 (35.8 to 54.5) | 23.3 (17 to 31.1) | 10.2 (3.2 to 16.9) | 8.8 (1.5 to 16.9) | 13 (3 to 22.7) |
| Qatar | Incidence | Age-standardized | 37.6 (28 to 50.1) | 32.6 (23.8 to 43.4) | 40.8 (30 to 54.1) | 37.6 (27.6 to 50.6) | 32.7 (24.6 to 43.8) | 41.1 (29.8 to 55.6) | 0 (-4.4 to 5.1) | 0.1 (-6 to 7.1) | 0.8 (-5.9 to 7.1) |
|  |  | All ages | 329.4 (256.5 to 424.6) | 76.7 (59.9 to 99.5) | 252.7 (195 to 327.1) | 365.7 (282.6 to 474.3) | 91 (71.1 to 117.8) | 274.7 (209.7 to 354.6) | 11 (5.1 to 16.8) | 18.7 (11.4 to 28.2) | 8.7 (1.3 to 16) |
|  | Prevalence | Age-standardized | 401.7 (303.2 to 523.8) | 349.1 (265.1 to 461.7) | 441.2 (333.2 to 574.1) | 402.6 (305.8 to 529.3) | 349.7 (262.6 to 464.3) | 444.3 (334.1 to 586.4) | 0.2 (-4 to 4.6) | 0.2 (-6.7 to 8.3) | 0.7 (-5.4 to 6.5) |
|  |  | All ages | 2720.4 (2076.2 to 3661.8) | 656.9 (504.3 to 852.2) | 2063.5 (1552.6 to 2824.5) | 3005 (2251.2 to 3953.1) | 777.1 (597.5 to 1013.1) | 2227.9 (1652.1 to 2974.8) | 10.5 (4.1 to 16.6) | 18.3 (10.9 to 26.5) | 8 (-0.5 to 16.7) |
|  | DALYs (Disability-Adjusted Life Years) | Age-standardized | 70.8 (43 to 92.8) | 82.7 (40.4 to 115.1) | 58.3 (42.4 to 77.4) | 78.5 (45.5 to 103.7) | 88.9 (42 to 126.2) | 65.8 (46.4 to 91) | 10.8 (2.1 to 18.3) | 7.6 (-0.7 to 14.2) | 12.8 (0.4 to 25.8) |
|  |  | All ages | 362.2 (265 to 496.3) | 125.1 (79 to 169.8) | 237.1 (151 to 347.9) | 407.6 (298 to 555.1) | 148.5 (90.1 to 200.3) | 259.1 (168.9 to 380.5) | 12.5 (1 to 25.5) | 18.7 (9.1 to 27.8) | 9.3 (-6.3 to 29.9) |
|  | Deaths | Age-standardized | 3.3 (1.2 to 4.8) | 4.4 (1.2 to 6.9) | 1.9 (1 to 3.4) | 4.1 (1.5 to 6) | 5.1 (1.4 to 8) | 2.6 (1.3 to 5) | 24.6 (13.6 to 34.3) | 17.8 (9.5 to 27.3) | 40 (14 to 60.5) |
|  |  | All ages | 8 (4.6 to 12.2) | 4.6 (1.7 to 7.2) | 3.4 (2 to 5.4) | 10 (5.4 to 15) | 5.9 (2 to 9) | 4.1 (2.5 to 6.5) | 24.5 (11.6 to 34.5) | 27.6 (16.6 to 36.7) | 20.3 (3.5 to 37.4) |
| Saudi Arabia | Incidence | Age-standardized | 36.6 (27 to 50.4) | 31.9 (23.7 to 43.3) | 39.9 (29.1 to 54.3) | 36.9 (27.2 to 49.8) | 32.1 (23.8 to 43) | 40.1 (29.5 to 54.1) | 0.8 (-3.9 to 5.5) | 0.7 (-6.8 to 9.8) | 0.5 (-6.9 to 6.3) |
|  |  | All ages | 5447 (4219.2 to 7004.2) | 1856.2 (1438.6 to 2432.5) | 3590.8 (2773.2 to 4577.8) | 6095.8 (4757.1 to 7921.4) | 2069.2 (1593.7 to 2712) | 4026.6 (3126.7 to 5193.3) | 11.9 (7.7 to 16.7) | 11.5 (3.2 to 20) | 12.1 (5.3 to 18) |
|  | Prevalence | Age-standardized | 388.5 (297.2 to 514.6) | 336.5 (253.9 to 445) | 426.7 (325.6 to 561.4) | 391.9 (301.9 to 511.7) | 338.8 (256.8 to 445.4) | 429.6 (326.8 to 558) | 0.9 (-3.3 to 5.2) | 0.7 (-6.4 to 9.5) | 0.7 (-6.6 to 6.2) |
|  |  | All ages | 48451.8 (37670.9 to 63728.6) | 16336.9 (12560 to 21301.4) | 32114.8 (24969.1 to 42035.5) | 54167 (42124.9 to 71479.1) | 18072.8 (13872.5 to 23966.4) | 36094.2 (27819.9 to 47627.7) | 11.8 (7.2 to 16.6) | 10.6 (3.2 to 19.6) | 12.4 (4.9 to 18.9) |
|  | DALYs (Disability-Adjusted Life Years) | Age-standardized | 72.8 (59.4 to 88.5) | 84.9 (66.4 to 102.4) | 63.3 (49.2 to 80.6) | 72.2 (58.2 to 88.6) | 83.9 (65.2 to 101.6) | 63.2 (48.9 to 80.2) | -0.8 (-4.9 to 3.2) | -1.2 (-6.5 to 4.4) | -0.1 (-5.7 to 5.2) |
|  |  | All ages | 8275.6 (6644.2 to 10287.2) | 3835.1 (2988 to 4730.2) | 4440.5 (3408.8 to 5695.4) | 9049.2 (7157.3 to 11409) | 4112.3 (3182.8 to 5123.5) | 4936.8 (3746.4 to 6552.8) | 9.3 (2.7 to 16.2) | 7.2 (-0.8 to 15.3) | 11.2 (0.9 to 22.2) |
|  | Deaths | Age-standardized | 3.4 (2.7 to 4.2) | 4.6 (3.4 to 5.7) | 2.4 (1.9 to 3.2) | 3.4 (2.7 to 4.1) | 4.5 (3.3 to 5.7) | 2.4 (1.8 to 3.2) | -1.9 (-6.4 to 2.4) | -1.9 (-8.3 to 4.1) | -0.9 (-6.2 to 6.1) |
|  |  | All ages | 238.3 (192 to 286.3) | 141.5 (106.5 to 175.5) | 96.8 (73.6 to 125.5) | 248.9 (197.4 to 305.4) | 145 (106.4 to 183.5) | 103.9 (77 to 135.7) | 4.4 (-1 to 9.7) | 2.5 (-4.5 to 9.4) | 7.3 (0.8 to 14.8) |
| Somalia | Incidence | Age-standardized | 35.6 (27.3 to 47.2) | 35.3 (27.2 to 47) | 36.1 (27.4 to 48.8) | 35.6 (27.4 to 47.3) | 35.5 (27.4 to 46.7) | 35.8 (27.4 to 48.1) | -0.1 (-4.5 to 3.9) | 0.5 (-5.8 to 6.5) | -1 (-6.8 to 5.9) |
|  |  | All ages | 1851.1 (1450.2 to 2391.2) | 1046.9 (820.9 to 1349.2) | 804.1 (629.4 to 1056.2) | 1950.9 (1521.5 to 2560.4) | 1112.8 (857.9 to 1455.1) | 838.1 (649.5 to 1096.5) | 5.4 (0.6 to 10.1) | 6.3 (0.1 to 13.4) | 4.2 (-2.9 to 11.2) |
|  | Prevalence | Age-standardized | 391.9 (309.2 to 513.1) | 386.5 (304 to 499.2) | 403.2 (316.3 to 529.1) | 392 (308.4 to 516.9) | 388.5 (302.7 to 514.1) | 400.5 (313.2 to 526.7) | 0 (-4.3 to 4.3) | 0.5 (-5.5 to 7.2) | -0.7 (-7.1 to 5.5) |
|  |  | All ages | 17811.5 (13792.2 to 23331.5) | 10306.2 (7933.2 to 13504.2) | 7505.3 (5792.8 to 9845.8) | 18687.3 (14346.6 to 24314.4) | 10906.2 (8318.8 to 14271.9) | 7781.1 (5936 to 10138.6) | 4.9 (-0.1 to 9.9) | 5.8 (-1 to 13.6) | 3.7 (-3.6 to 11.2) |
|  | DALYs (Disability-Adjusted Life Years) | Age-standardized | 65.5 (40 to 99.1) | 62.9 (40.2 to 102.9) | 69.9 (37.7 to 106.2) | 63.6 (39.5 to 97.4) | 61.8 (39.4 to 101) | 66.3 (36 to 100.9) | -2.9 (-8.1 to 2.2) | -1.7 (-8 to 4.8) | -5.1 (-11.1 to 1.6) |
|  |  | All ages | 2668.3 (1649.7 to 4029) | 1491.6 (947 to 2378.4) | 1176.7 (631.9 to 1778.3) | 2767.5 (1735.5 to 4169) | 1562.4 (996 to 2489.9) | 1205.1 (659.7 to 1841.7) | 3.7 (-2.6 to 10) | 4.7 (-3.1 to 14.1) | 2.4 (-5.9 to 12.2) |
|  | Deaths | Age-standardized | 2.4 (1.1 to 4.2) | 2.3 (1.2 to 4.7) | 2.7 (0.8 to 4.9) | 2.2 (1 to 4) | 2.2 (1.1 to 4.4) | 2.3 (0.7 to 4.2) | -8.4 (-16.3 to -1.5) | -5.5 (-13.8 to 2.6) | -14 (-25.7 to -5.2) |
|  |  | All ages | 60.1 (25.6 to 109.3) | 34.6 (16.9 to 71.3) | 25.5 (6.9 to 47.1) | 60.9 (26 to 110.8) | 35.6 (17.4 to 71.8) | 25.3 (6.8 to 47) | 1.3 (-5.7 to 7.3) | 2.9 (-4.1 to 9.7) | -1 (-8.6 to 5.6) |
| Sudan | Incidence | Age-standardized | 37.8 (28 to 51.4) | 33.5 (25 to 45) | 41.4 (30.1 to 55.8) | 37.8 (27.5 to 51) | 33.4 (24.5 to 44.7) | 41.4 (30.2 to 55.7) | -0.1 (-4.5 to 6) | -0.2 (-5.6 to 7.7) | 0.1 (-6.1 to 8.4) |
|  |  | All ages | 6330.3 (4819.6 to 8433.4) | 2534.5 (1947.4 to 3347.9) | 3795.8 (2870.2 to 5109.6) | 6613.9 (4970.4 to 8738.6) | 2675.5 (2017.2 to 3528.6) | 3938.4 (2936.4 to 5238.6) | 4.5 (0.2 to 10.5) | 5.6 (-0.3 to 13.8) | 3.8 (-2.7 to 12.3) |
|  | Prevalence | Age-standardized | 393.7 (298.6 to 522.5) | 343.7 (259.5 to 458.7) | 435 (330.4 to 574.2) | 394.5 (298.4 to 515.7) | 344.1 (258.2 to 452.7) | 437.1 (331.1 to 573) | 0.2 (-3.7 to 5.7) | 0.1 (-6.2 to 7.9) | 0.5 (-6 to 7.7) |
|  |  | All ages | 61479.1 (47155 to 80271.6) | 24020.4 (18363.1 to 31553.1) | 37458.8 (28538.5 to 48850) | 63916.4 (49241.7 to 83109.6) | 25242.2 (19254.3 to 33337.7) | 38674.2 (29612.8 to 49851) | 4 (-0.4 to 9.4) | 5.1 (-1.7 to 13.2) | 3.2 (-3.6 to 10.1) |
|  | DALYs (Disability-Adjusted Life Years) | Age-standardized | 71.3 (55 to 89.1) | 77.5 (55.2 to 99.5) | 66.5 (50.6 to 85) | 72.5 (56.1 to 90.4) | 78.7 (56.3 to 101.5) | 67.5 (51.7 to 85.8) | 1.7 (-3.3 to 7.2) | 1.6 (-4.5 to 8.4) | 1.6 (-4.5 to 8.6) |
|  |  | All ages | 10525.2 (8046.8 to 13056.8) | 5132.4 (3678.5 to 6538.1) | 5392.8 (4073.2 to 6913.9) | 10967.1 (8323.7 to 13688.8) | 5406.5 (3854.9 to 6957.8) | 5560.7 (4156.9 to 6986.6) | 4.2 (-1.1 to 10) | 5.3 (-1.4 to 12.4) | 3.1 (-3.7 to 10.5) |
|  | Deaths | Age-standardized | 3.1 (2.2 to 4.1) | 3.8 (2.4 to 5) | 2.6 (1.8 to 3.5) | 3.2 (2.3 to 4.2) | 3.9 (2.6 to 5.3) | 2.7 (1.8 to 3.7) | 3.1 (-3.9 to 11.7) | 2.8 (-4.2 to 11.5) | 2.7 (-5.1 to 12.3) |
|  |  | All ages | 385.6 (274.8 to 500.4) | 212.4 (135.5 to 283.8) | 173.2 (119.6 to 234.2) | 394.5 (287.3 to 518.9) | 220.4 (142.4 to 300.9) | 174.1 (119.4 to 234.4) | 2.3 (-4.6 to 10.6) | 3.7 (-3.2 to 12.2) | 0.5 (-7.4 to 9.4) |
| Syrian Arab Republic | Incidence | Age-standardized | 35.3 (26.2 to 47.3) | 31.2 (22.9 to 42.1) | 38.8 (28.6 to 52.2) | 35.3 (25.7 to 47.1) | 31.1 (22.8 to 41.5) | 39.1 (28.5 to 52.3) | 0.1 (-4.4 to 4.6) | -0.2 (-7.6 to 6.1) | 0.6 (-4.8 to 8.3) |
|  |  | All ages | 3973.9 (2992.8 to 5317.8) | 1713.7 (1264.3 to 2297.4) | 2260.1 (1699.9 to 3012.4) | 4228.1 (3162.9 to 5673.7) | 1836.7 (1379.7 to 2463.1) | 2391.4 (1787.2 to 3187.8) | 6.4 (0.7 to 11.9) | 7.2 (-1.2 to 14) | 5.8 (-0.1 to 13.6) |
|  | Prevalence | Age-standardized | 376.3 (285.1 to 496.9) | 327 (245.2 to 433.2) | 412.9 (315.1 to 541.2) | 377.8 (288.1 to 497.3) | 327.4 (250.9 to 430.3) | 416.7 (317.9 to 547.8) | 0.4 (-4 to 5.3) | 0.1 (-7.4 to 6.3) | 0.9 (-4.8 to 7.5) |
|  |  | All ages | 38997.4 (30070.8 to 50903.7) | 16349.9 (12361.3 to 21464.9) | 22647.5 (17576.8 to 29802.3) | 41606 (32219.9 to 54570.1) | 17599.7 (13550.2 to 23401.2) | 24006.4 (18328.6 to 31424.7) | 6.7 (1.8 to 11.4) | 7.6 (-0.6 to 15.1) | 6 (-0.6 to 13) |
|  | DALYs (Disability-Adjusted Life Years) | Age-standardized | 84.4 (60.5 to 104.2) | 112.1 (69.2 to 150.6) | 72.4 (53.1 to 91.1) | 85.7 (63.8 to 105.2) | 113.4 (75 to 149.4) | 73.4 (55.3 to 90.9) | 1.5 (-5.8 to 10) | 1.1 (-7.5 to 16.7) | 1.5 (-5.7 to 10.1) |
|  |  | All ages | 7602.7 (5616 to 9481) | 3991.5 (2739.1 to 5213) | 3611.1 (2726.7 to 4540.2) | 8108.2 (6087.1 to 10069) | 4281.7 (2950 to 5489.8) | 3826.5 (2908.8 to 4787.1) | 6.6 (-0.9 to 15.3) | 7.3 (-1.9 to 17.5) | 6 (-2.3 to 15.3) |
|  | Deaths | Age-standardized | 4.4 (2.6 to 5.9) | 7.5 (3.8 to 10.7) | 3.2 (2 to 4.4) | 4.6 (2.9 to 5.9) | 7.7 (4.6 to 10.5) | 3.3 (2.2 to 4.4) | 2.8 (-6.1 to 18.2) | 2 (-8.1 to 29.5) | 2.6 (-6.8 to 16.7) |
|  |  | All ages | 298.1 (187.9 to 392.1) | 174.4 (98.6 to 250.6) | 123.7 (82.4 to 162.9) | 318.3 (209.6 to 419.4) | 187 (110.6 to 266.7) | 131.3 (91.4 to 171.4) | 6.8 (-3.2 to 21.2) | 7.2 (-3.5 to 22.6) | 6.2 (-4.3 to 18.7) |
| Tunisia | Incidence | Age-standardized | 34.4 (25.4 to 45.8) | 30.9 (22.7 to 40.4) | 38.4 (28.2 to 51.7) | 34.5 (25 to 45.6) | 30.9 (22.5 to 40.7) | 38.6 (28.1 to 51.5) | 0.2 (-4.1 to 4.1) | 0 (-6.6 to 6.5) | 0.5 (-5.4 to 6.4) |
|  |  | All ages | 4151.6 (3091.2 to 5511.7) | 1946.3 (1453.4 to 2545.4) | 2205.3 (1619.1 to 2953.6) | 4413.6 (3251.2 to 5837.6) | 2076 (1527.7 to 2745.4) | 2337.6 (1718.5 to 3104.6) | 6.3 (1.8 to 10.7) | 6.7 (-0.6 to 13.3) | 6 (-0.1 to 12.9) |
|  | Prevalence | Age-standardized | 363.9 (276 to 479.2) | 325 (247.9 to 427) | 410.3 (308.2 to 542.4) | 365 (276.8 to 471.6) | 325.2 (243.8 to 421.9) | 413.1 (313.6 to 534.1) | 0.3 (-3.4 to 4.3) | 0.1 (-6.1 to 6.2) | 0.7 (-5 to 6) |
|  |  | All ages | 42476.7 (32333.7 to 55607.1) | 20029.6 (15362.2 to 26177.6) | 22447.1 (16948.1 to 29626.3) | 45068.6 (34412.2 to 58372.3) | 21340.3 (15960.1 to 27603.6) | 23728.4 (18292.8 to 30632.1) | 6.1 (2.3 to 10.6) | 6.5 (0 to 13) | 5.7 (-0.2 to 11.8) |
|  | DALYs (Disability-Adjusted Life Years) | Age-standardized | 81.7 (60.6 to 107.2) | 82.7 (57.2 to 112.7) | 81.1 (60.2 to 107) | 81.8 (60.5 to 107.5) | 82.4 (56.5 to 113.9) | 82 (59.9 to 107.2) | 0.2 (-5.2 to 6.6) | -0.4 (-6.7 to 7.1) | 1.1 (-5.4 to 9.2) |
|  |  | All ages | 8822.4 (6551.4 to 11472.7) | 4897.9 (3383.3 to 6693.6) | 3924.5 (2933.1 to 5099.4) | 9283.2 (6895.4 to 12246.5) | 5169.3 (3554 to 7089) | 4114 (3042 to 5309.9) | 5.2 (-0.5 to 11.9) | 5.5 (-1.2 to 13.2) | 4.8 (-2 to 13) |
|  | Deaths | Age-standardized | 4.6 (3 to 6.4) | 4.8 (2.9 to 7.2) | 4.4 (2.8 to 6.5) | 4.6 (3 to 6.7) | 4.8 (2.8 to 7.4) | 4.5 (2.9 to 6.7) | 0.5 (-6.6 to 8) | -0.3 (-8.7 to 9) | 2 (-7.8 to 11.5) |
|  |  | All ages | 441.6 (291.4 to 608.1) | 268.8 (164.2 to 399.8) | 172.8 (115.4 to 244.7) | 462.5 (305.1 to 649.1) | 282.8 (168.8 to 433.3) | 179.7 (121.9 to 256.7) | 4.7 (-2.7 to 12.8) | 5.2 (-3.5 to 15.2) | 4 (-6 to 13.3) |
| United Arab Emirates | Incidence | Age-standardized | 39.3 (28.6 to 52.7) | 33 (24.3 to 44.9) | 41 (29.5 to 54.8) | 39.7 (29.2 to 52.8) | 33.1 (24.5 to 44.6) | 41.2 (30.1 to 54.6) | 0.8 (-4 to 7) | 0.3 (-5.4 to 8) | 0.5 (-5.2 to 7.8) |
|  |  | All ages | 1369.2 (1036.4 to 1811.6) | 252.8 (194.2 to 331.5) | 1116.4 (836.4 to 1466.9) | 1662 (1236.1 to 2172.9) | 305.1 (229.8 to 412.2) | 1357 (1001.6 to 1756.7) | 21.4 (14 to 29.7) | 20.7 (12.4 to 30.5) | 21.6 (13.5 to 31.6) |
|  | Prevalence | Age-standardized | 420.1 (318.9 to 550.1) | 346.1 (262.4 to 455.2) | 437.4 (332 to 577.6) | 424.7 (326.5 to 554.8) | 347.4 (266 to 458.8) | 440.6 (337.3 to 572.5) | 1.1 (-3.4 to 6.4) | 0.4 (-5.6 to 7.4) | 0.7 (-4.7 to 6.8) |
|  |  | All ages | 10772.6 (7843.7 to 14882.2) | 1884.5 (1371.1 to 2534.5) | 8888.1 (6403.4 to 12414) | 13474.7 (9821 to 18266.3) | 2310.2 (1710.7 to 3127) | 11164.5 (8037.4 to 15121.3) | 25.1 (16.1 to 34.3) | 22.6 (14.6 to 31.9) | 25.6 (15.6 to 36.8) |
|  | DALYs (Disability-Adjusted Life Years) | Age-standardized | 113.7 (88.3 to 138.9) | 302.7 (220.3 to 376.4) | 69.9 (53.7 to 88.4) | 85.8 (66.1 to 105.2) | 232 (162.2 to 285.6) | 57.4 (40.7 to 74.2) | -24.5 (-33.8 to -16.6) | -23.4 (-33.8 to -13.6) | -18 (-27.5 to -8.6) |
|  |  | All ages | 1625.1 (1239.5 to 2148.8) | 550.5 (424 to 679) | 1074.6 (756.1 to 1514) | 1763.2 (1272.6 to 2342) | 551.8 (429.8 to 673.5) | 1211.4 (794.3 to 1692.2) | 8.5 (-6.8 to 25.3) | 0.2 (-12.8 to 14) | 12.7 (-7.1 to 34.8) |
|  | Deaths | Age-standardized | 5.9 (4.3 to 7.4) | 21.1 (14.9 to 26.5) | 2.8 (1.8 to 3.8) | 3.8 (2.5 to 4.9) | 15.7 (10.2 to 19.8) | 1.8 (1.1 to 2.6) | -35.1 (-45.9 to -24.8) | -25.6 (-37.3 to -14.8) | -34.8 (-47.9 to -21.3) |
|  |  | All ages | 33.9 (26.7 to 42.8) | 20 (14.7 to 25.3) | 14 (10.6 to 19.7) | 30.1 (23.5 to 37.4) | 17.9 (13.3 to 22.4) | 12.3 (9.3 to 16.7) | -11.2 (-26.7 to 5.2) | -10.4 (-24.6 to 3.6) | -12.4 (-30.7 to 9.4) |
| Yemen | Incidence | Age-standardized | 32.8 (24.1 to 44.2) | 29.5 (21.8 to 40.2) | 36.3 (26.7 to 48.3) | 32.9 (24.2 to 44.1) | 29.4 (21.7 to 39.5) | 36.5 (26.5 to 49.8) | 0.2 (-4.7 to 5.2) | 0 (-6.7 to 6.8) | 0.6 (-5.4 to 8.9) |
|  |  | All ages | 3866.6 (2924 to 5053.1) | 1747.3 (1335.1 to 2340.7) | 2119.3 (1580.7 to 2758.9) | 4104.8 (3060.8 to 5426.7) | 1867.9 (1400.4 to 2466.6) | 2236.9 (1682.6 to 2949.6) | 6.2 (1.5 to 11.2) | 6.9 (-0.1 to 15.2) | 5.5 (-1 to 13.7) |
|  | Prevalence | Age-standardized | 336.4 (256.6 to 440.1) | 298 (227.8 to 394.3) | 377.6 (286.8 to 490.4) | 337.2 (255.8 to 443.6) | 298.1 (225.2 to 389.4) | 379.9 (287.3 to 501.9) | 0.2 (-4.3 to 5) | 0.1 (-5.9 to 7.6) | 0.6 (-5.5 to 8.3) |
|  |  | All ages | 36340.4 (28246.4 to 47119.2) | 16285.3 (12619 to 21289.8) | 20055.1 (15487.4 to 25678.6) | 38432.1 (29592.5 to 49670.6) | 17355.6 (13127.6 to 22822.7) | 21076.4 (16219.7 to 27197.2) | 5.8 (1 to 10.6) | 6.6 (0.5 to 14.2) | 5.1 (-1.2 to 12.4) |
|  | DALYs (Disability-Adjusted Life Years) | Age-standardized | 73.9 (55.2 to 97.6) | 77.2 (55.1 to 105.4) | 70.4 (50 to 94.4) | 75.2 (55.6 to 97.4) | 78.8 (55.6 to 105.5) | 71.2 (49.6 to 96.3) | 1.7 (-2.7 to 7.1) | 2.1 (-3.3 to 8.1) | 1.1 (-5.4 to 7.7) |
|  |  | All ages | 7220.6 (5353.8 to 9416.6) | 3868.4 (2721.1 to 5315.8) | 3352.2 (2343.8 to 4477.6) | 7684.9 (5624.9 to 9928.4) | 4164.3 (2900.6 to 5519.6) | 3520.5 (2425.4 to 4748.5) | 6.4 (1.2 to 12.2) | 7.6 (1.2 to 15) | 5 (-2 to 12.4) |
|  | Deaths | Age-standardized | 3.7 (2.5 to 5) | 4 (2.6 to 6) | 3.2 (1.9 to 5) | 3.7 (2.6 to 5.1) | 4.2 (2.6 to 6) | 3.3 (1.9 to 5) | 2.4 (-3.7 to 9.6) | 3 (-3 to 10.2) | 0.9 (-8 to 9.3) |
|  |  | All ages | 276.4 (189.4 to 381.2) | 163.3 (102 to 240) | 113 (67.9 to 178.7) | 290.8 (198.2 to 403.6) | 174.3 (107.9 to 253.2) | 116.5 (68.9 to 181) | 5.2 (-0.5 to 12.3) | 6.7 (0.1 to 14) | 3 (-5 to 10.9) |

**Table S3.** All ages number and age‑standardized rate of incidence, prevalence, disability-adjusted life years (DALYs), and deaths of atrial fibrillation and flutter by sex in 1990 and 2021 and overall percent change over 1990–2021 in the Eastern Mediterranean Region countries

| Location | Measure | Age, Metric | Year | | | | | | % Change (1990 to 2021) | | |
| --- | --- | --- | --- | --- | --- | --- | --- | --- | --- | --- | --- |
|  |  |  | 1990 | | | 2021 | | |  |  |  |
|  |  |  | Both | Women | Men | Both | Women | Men | Both | Women | Men |
| Eastern Mediterranean Region | Incidence | Age-standardized | 40.6 (30 to 54.8) | 36.1 (26.6 to 48.5) | 44.7 (33 to 60.1) | 41.8 (31 to 56.1) | 37.9 (28.2 to 50.7) | 45.6 (33.7 to 61.2) | 3 (1.1 to 4.8) | 5 (3.1 to 6.8) | 2 (-0.7 to 4.4) |
|  |  | All ages | 64362.4 (48684.7 to 85763.7) | 26832.3 (20314.9 to 35807) | 37530 (28263 to 49849.2) | 168555 (129706.9 to 220844.2) | 73645.7 (56223.9 to 97185.5) | 94909.3 (73081 to 124276) | 161.9 (154.7 to 170.3) | 174.5 (168.6 to 181.2) | 152.9 (143.6 to 162.9) |
|  | Prevalence | Age-standardized | 419.7 (324.2 to 551) | 363.7 (278.2 to 478.8) | 470 (361.7 to 616.3) | 439 (338.8 to 577) | 391.3 (299.9 to 515.5) | 483.3 (372.4 to 632.3) | 4.6 (2.9 to 6.3) | 7.6 (5.7 to 9.5) | 2.8 (0.1 to 5.3) |
|  |  | All ages | 617721.6 (482076 to 807810.5) | 252173.5 (193436.4 to 332030) | 365548.1 (285244.4 to 475780.5) | 1620763.1 (1270893.6 to 2108347.5) | 698891.7 (539905.9 to 917111.1) | 921871.4 (724649.8 to 1194163.1) | 162.4 (156.3 to 168.5) | 177.1 (171.7 to 183.1) | 152.2 (143.9 to 160.5) |
|  | DALYs (Disability-Adjusted Life Years) | Age-standardized | 73.7 (56.6 to 93.6) | 76.5 (57.6 to 102.3) | 71.3 (51.5 to 91.3) | 83.4 (68.7 to 99.8) | 89.9 (72.9 to 105.4) | 77.5 (62.6 to 95.2) | 13.1 (-2.8 to 30.6) | 17.5 (-4.5 to 38.3) | 8.7 (-3.8 to 31) |
|  |  | All ages | 100324.1 (76902.1 to 127934) | 48467.6 (36633.7 to 64013.4) | 51856.5 (37467.3 to 66898.7) | 279136.9 (229153.6 to 335913.4) | 141800.5 (115263.1 to 171031.8) | 137336.4 (109551.2 to 170691.1) | 178.2 (141.4 to 218.6) | 192.6 (139.9 to 241.2) | 164.8 (135.2 to 214.5) |
|  | Deaths | Age-standardized | 3.2 (2.3 to 4.2) | 3.7 (2.7 to 5.3) | 2.7 (1.7 to 3.5) | 3.9 (3.2 to 4.5) | 4.8 (3.9 to 5.6) | 3.2 (2.6 to 3.8) | 23.7 (-4 to 60.1) | 28.9 (-5.8 to 65.9) | 17.2 (-7.1 to 71.3) |
|  |  | All ages | 3361.1 (2447.1 to 4411.3) | 1872.2 (1341.1 to 2679.7) | 1488.9 (926.6 to 1896.6) | 10320.6 (8504.4 to 11780.6) | 5967.1 (4845.1 to 6938.7) | 4353.4 (3593.4 to 5204.6) | 207.1 (138.6 to 297.6) | 218.7 (133.9 to 309.3) | 192.4 (131.2 to 329.4) |
| Afghanistan | Incidence | Age-standardized | 31.9 (23.7 to 43) | 28.6 (21.2 to 38.6) | 34.8 (25.6 to 46.5) | 33.3 (24.5 to 44.5) | 29.9 (22 to 39.9) | 37 (27.1 to 49.6) | 4.3 (-0.1 to 8.7) | 4.5 (-2.2 to 11.5) | 6.3 (-1.2 to 13.1) |
|  |  | All ages | 1905.7 (1414.6 to 2571) | 805 (592.1 to 1084.1) | 1100.7 (806.6 to 1482.4) | 2735.2 (2055.6 to 3602.5) | 1300.7 (957.6 to 1719.9) | 1434.5 (1078.6 to 1901.7) | 43.5 (30.4 to 59.5) | 61.6 (49.7 to 75.3) | 30.3 (12.3 to 49.1) |
|  | Prevalence | Age-standardized | 312.8 (240.1 to 412.3) | 274.6 (204.6 to 361.3) | 345.6 (265.2 to 452.7) | 338.8 (257.5 to 442.8) | 298.1 (221.2 to 394.7) | 380.9 (289 to 505.4) | 8.3 (3.9 to 13.1) | 8.6 (1.9 to 15.2) | 10.2 (3.3 to 17) |
|  |  | All ages | 17747.2 (13622.4 to 23160.1) | 7293 (5469.6 to 9635.2) | 10454.1 (7874.8 to 13670.2) | 25638.9 (19700.9 to 33184.4) | 11934 (8982.2 to 15588.1) | 13704.9 (10544 to 17473.7) | 44.5 (35.2 to 53.4) | 63.6 (53 to 74.8) | 31.1 (18.8 to 42.2) |
|  | DALYs (Disability-Adjusted Life Years) | Age-standardized | 63 (40.5 to 92.6) | 63.7 (41.6 to 103.9) | 62.6 (36.8 to 93.5) | 72.1 (51.5 to 95.1) | 73.9 (53.1 to 105.5) | 69.9 (45.7 to 93.9) | 14.3 (-6.2 to 37.4) | 16 (-8.5 to 45.6) | 11.6 (-11.8 to 40.8) |
|  |  | All ages | 3394.1 (2188.1 to 4976.7) | 1611.1 (1044.4 to 2603) | 1783 (1051 to 2674.1) | 5074.3 (3663.3 to 6760.9) | 2744 (1928.8 to 3920.2) | 2330.2 (1519.7 to 3157.8) | 49.5 (20.9 to 82.9) | 70.3 (32 to 117.8) | 30.7 (2.1 to 64.4) |
|  | Deaths | Age-standardized | 2.7 (1.5 to 4.5) | 2.9 (1.6 to 5.3) | 2.6 (1.1 to 4.5) | 3.3 (2 to 4.9) | 3.6 (2.3 to 5.8) | 3 (1.6 to 4.6) | 19.4 (-7 to 61) | 23.6 (-9 to 67.9) | 12.9 (-17.1 to 66.8) |
|  |  | All ages | 112.6 (59.4 to 189.7) | 56.3 (30.3 to 105.6) | 56.3 (23.3 to 98.5) | 179.3 (110.9 to 263.9) | 101.4 (64.7 to 159.3) | 77.9 (40.7 to 118.1) | 59.2 (22.3 to 118.5) | 80.2 (29.4 to 152.1) | 38.2 (-2.3 to 105) |
| Bahrain | Incidence | Age-standardized | 35.3 (26.2 to 47.7) | 31.7 (23.8 to 42.7) | 39.1 (28.4 to 53.6) | 36.3 (26.6 to 48.2) | 32.2 (23.8 to 42.8) | 40.3 (29.4 to 53.8) | 2.7 (-2.5 to 7.1) | 1.3 (-5.9 to 8.2) | 2.8 (-4.5 to 9.9) |
|  |  | All ages | 53.4 (40.8 to 70.2) | 20.8 (15.5 to 27.5) | 32.6 (24.7 to 43) | 297 (229 to 382.3) | 104.9 (78.6 to 139.4) | 192.1 (148 to 243.9) | 456.3 (410.8 to 499) | 404 (363.1 to 452.2) | 489.7 (428.8 to 546.1) |
|  | Prevalence | Age-standardized | 361.4 (274.6 to 473.5) | 323.9 (244.2 to 424.2) | 407.4 (310.2 to 541) | 386.7 (293.8 to 505.6) | 341.1 (256.6 to 449.1) | 432.3 (326.7 to 562.5) | 7 (1.8 to 11.5) | 5.3 (-1.8 to 12.2) | 6.1 (-1.7 to 13.1) |
|  |  | All ages | 462.3 (355.5 to 611.2) | 186.5 (142.2 to 242.9) | 275.8 (213 to 367.8) | 2667.3 (2055.1 to 3468.5) | 954.7 (735.4 to 1248.1) | 1712.6 (1303.4 to 2213.8) | 476.9 (441.8 to 510.2) | 412 (376.1 to 450.6) | 520.9 (472.9 to 567.9) |
|  | DALYs (Disability-Adjusted Life Years) | Age-standardized | 113.1 (67 to 143.1) | 130.1 (73.8 to 173.2) | 87.7 (52.7 to 121.6) | 94.9 (57.6 to 122.8) | 111.4 (61.4 to 152.7) | 74.3 (49.4 to 99.3) | -16.1 (-32.7 to -2) | -14.4 (-35 to 4.6) | -15.2 (-33.4 to 8.5) |
|  |  | All ages | 101.3 (65.3 to 127.5) | 57.7 (33.4 to 76.3) | 43.6 (28.6 to 58.7) | 442 (315.6 to 569.3) | 226.6 (142.2 to 298.5) | 215.4 (159.5 to 281.1) | 336.2 (246.4 to 433.4) | 292.8 (194.3 to 389.8) | 393.7 (270.1 to 545.6) |
|  | Deaths | Age-standardized | 7 (3.4 to 9.3) | 8.3 (4 to 11.5) | 4.5 (2 to 6.8) | 5.7 (2.6 to 7.9) | 7.2 (3 to 10.6) | 3.6 (1.9 to 5.6) | -18.7 (-39 to -0.7) | -13.2 (-38.7 to 11.2) | -20.8 (-42.4 to 19.4) |
|  |  | All ages | 3.7 (1.9 to 4.8) | 2.5 (1.2 to 3.5) | 1.2 (0.6 to 1.7) | 14.7 (8.1 to 19.9) | 10.1 (4.8 to 14.6) | 4.6 (3.1 to 6.6) | 299.1 (190.1 to 408.1) | 298.6 (176.2 to 418.4) | 300.2 (170.4 to 548.9) |
| Djibouti | Incidence | Age-standardized | 34.8 (26.7 to 46.4) | 34.9 (26.9 to 46.5) | 34.9 (26.6 to 45.9) | 37.4 (28.6 to 49.6) | 37.2 (28.4 to 49.6) | 37.6 (28.7 to 49.3) | 7.3 (3.1 to 13.1) | 6.7 (-0.6 to 14.6) | 7.8 (0.2 to 14) |
|  |  | All ages | 42.9 (33.9 to 55.8) | 21.2 (16.6 to 27.7) | 21.7 (17.1 to 28.4) | 223.6 (175.4 to 292.7) | 103.5 (81.8 to 136.5) | 120.1 (93.1 to 158.2) | 421.1 (399.3 to 448) | 388.5 (355.7 to 426.7) | 452.8 (416 to 488.6) |
|  | Prevalence | Age-standardized | 388.3 (306 to 508.9) | 386.5 (300.9 to 505.4) | 393.4 (308 to 516.6) | 424.6 (335.8 to 555.3) | 421.1 (331.7 to 557.9) | 431 (335.1 to 562.5) | 9.4 (5.2 to 14.3) | 8.9 (1.9 to 17.1) | 9.6 (2 to 15.8) |
|  |  | All ages | 415.9 (323.7 to 544.6) | 207.5 (160.9 to 267.9) | 208.4 (160.5 to 274.2) | 2212.7 (1710.4 to 2890.5) | 1028.4 (791.2 to 1357.3) | 1184.3 (914.8 to 1539.8) | 432 (408.3 to 459) | 395.5 (363.5 to 432.5) | 468.3 (431.6 to 506.7) |
|  | DALYs (Disability-Adjusted Life Years) | Age-standardized | 82 (62.7 to 107.2) | 77.5 (54.4 to 112.5) | 88.2 (63 to 116.7) | 83.7 (61.7 to 110.9) | 78.6 (54.7 to 113.8) | 89.5 (63.7 to 119.6) | 2.1 (-17.8 to 24.7) | 1.5 (-18.5 to 24.7) | 1.5 (-19.9 to 31.5) |
|  |  | All ages | 75.6 (56.9 to 99.9) | 36 (25.5 to 52) | 39.5 (28.8 to 52.2) | 368.1 (267.3 to 489.4) | 163 (112.9 to 230.1) | 205.1 (146.9 to 270.6) | 387 (295.3 to 498.6) | 352.2 (263.1 to 453.7) | 418.8 (310.5 to 567.8) |
|  | Deaths | Age-standardized | 3.7 (2.6 to 5.2) | 3.5 (2.2 to 5.7) | 4.2 (2.5 to 6.1) | 3.8 (2.5 to 5.5) | 3.5 (2 to 5.7) | 4.2 (2.7 to 6.1) | 1.5 (-25 to 36.3) | 1 (-26.8 to 38.5) | 0.2 (-27.8 to 47.4) |
|  |  | All ages | 2.2 (1.5 to 3) | 1.1 (0.7 to 1.8) | 1.1 (0.7 to 1.6) | 10.5 (6.8 to 15.1) | 4.9 (2.8 to 8) | 5.6 (3.6 to 8.3) | 376.7 (246.8 to 555.6) | 341.6 (210.9 to 514.2) | 412 (257.2 to 681.5) |
| Egypt | Incidence | Age-standardized | 33 (24.1 to 44.2) | 29.4 (21.5 to 39.4) | 36.1 (26.3 to 48.5) | 36.9 (27.1 to 49.4) | 32.4 (24 to 43.5) | 40.3 (29.4 to 53.9) | 11.9 (5.8 to 17.7) | 10.3 (2.7 to 18.5) | 11.7 (3.4 to 19.4) |
|  |  | All ages | 7316.4 (5543.3 to 9606.5) | 3187.8 (2399.9 to 4229.6) | 4128.6 (3110.9 to 5400.8) | 19786.2 (14849 to 25927.2) | 8095.2 (5906 to 10812.7) | 11691 (8726.6 to 15465.4) | 170.4 (156 to 185.3) | 153.9 (135.1 to 176.7) | 183.2 (161.5 to 202.3) |
|  | Prevalence | Age-standardized | 333.8 (255.5 to 434.8) | 292 (221.8 to 383.1) | 367.1 (277.7 to 476.9) | 393 (297.7 to 514.2) | 336.8 (251.4 to 438.2) | 427.3 (322.5 to 564.1) | 17.7 (12.2 to 24) | 15.4 (8.1 to 24.1) | 16.4 (9 to 23.8) |
|  |  | All ages | 67055 (51334.8 to 87638.1) | 28513.4 (21619.7 to 37486.4) | 38541.6 (29378.5 to 50251.8) | 184696.9 (143378.1 to 239672.1) | 72748.6 (54793.2 to 95018.5) | 111948.3 (87183.8 to 146087) | 175.4 (162.5 to 190.4) | 155.1 (138.3 to 176.4) | 190.5 (171.7 to 210) |
|  | DALYs (Disability-Adjusted Life Years) | Age-standardized | 76.4 (61 to 96.3) | 89.5 (68.1 to 123.4) | 67.3 (50.4 to 86.2) | 75.9 (62.4 to 91.2) | 100 (80 to 116) | 68.6 (55.1 to 86) | -0.7 (-18.7 to 19.2) | 11.7 (-17.4 to 42) | 2 (-16.8 to 28.8) |
|  |  | All ages | 12708.7 (10080.8 to 15899.4) | 6449.5 (4958.7 to 8500.1) | 6259.2 (4681.9 to 8003.1) | 30353.3 (24425.2 to 37293) | 13938.9 (11213.4 to 17009.4) | 16414.4 (12876.7 to 20540.4) | 138.8 (98.4 to 182.3) | 116.1 (66 to 165.2) | 162.2 (116.6 to 224.7) |
|  | Deaths | Age-standardized | 4.1 (3.2 to 5.3) | 5.6 (4.1 to 8.3) | 3.1 (2.1 to 4) | 3.6 (2.9 to 4.3) | 6.4 (4.8 to 7.5) | 2.8 (2.2 to 3.4) | -12.1 (-34.9 to 18.2) | 13.3 (-25.1 to 58.9) | -8.5 (-34.7 to 35.4) |
|  |  | All ages | 459.1 (359.7 to 583.2) | 266.1 (200.2 to 374.9) | 192.9 (130.2 to 252.1) | 971.5 (799.2 to 1144.7) | 486.3 (389.3 to 575.3) | 485.2 (383 to 596.2) | 111.6 (58.1 to 181.5) | 82.7 (21.8 to 151.9) | 151.5 (78 to 282.5) |
| Iran (Islamic Republic of) | Incidence | Age-standardized | 37.3 (27.6 to 50.2) | 35 (25.9 to 46.8) | 40 (29.6 to 53.7) | 40.6 (30 to 54.4) | 37.4 (27.7 to 50.2) | 43.7 (32.3 to 58.7) | 8.6 (6.9 to 10.2) | 7 (5.3 to 8.8) | 9.2 (6.4 to 11.6) |
|  |  | All ages | 8136.4 (6131.5 to 10786.8) | 3690.9 (2775 to 4890.5) | 4445.5 (3327.9 to 5896.3) | 29418.3 (22665.1 to 38723.1) | 13622.3 (10363.4 to 18155.8) | 15796 (12121.5 to 20711) | 261.6 (236.1 to 289.9) | 269.1 (249.1 to 290.5) | 255.3 (223.9 to 291.2) |
|  | Prevalence | Age-standardized | 386 (296.2 to 507.6) | 358.1 (273.8 to 471.7) | 416.7 (318.6 to 546.4) | 425.4 (327.2 to 559.2) | 387.8 (298 to 510.7) | 461.1 (354.7 to 601.6) | 10.2 (8.6 to 11.7) | 8.3 (6.4 to 10.2) | 10.7 (8.1 to 12.8) |
|  |  | All ages | 75832.5 (58813.8 to 99095.2) | 34230.7 (26107.8 to 44725) | 41601.8 (32447.4 to 54379.3) | 294248.7 (230024.2 to 382165.9) | 134056.9 (103750.2 to 176465.5) | 160191.8 (124346.3 to 206830.5) | 288 (268.9 to 313.2) | 291.6 (274.1 to 311) | 285.1 (260.5 to 316.7) |
|  | DALYs (Disability-Adjusted Life Years) | Age-standardized | 70.5 (55.4 to 88.3) | 78.7 (61.2 to 98.7) | 61.4 (46.2 to 76.9) | 72.4 (57.4 to 88.3) | 83.5 (65.3 to 99.2) | 62.5 (47.4 to 80.3) | 2.6 (-13.1 to 18.2) | 6.1 (-14.5 to 24.6) | 1.8 (-9.9 to 23) |
|  |  | All ages | 12115.7 (9430.1 to 15214.4) | 6569.4 (5120.9 to 8236.8) | 5546.3 (4130.2 to 7086.4) | 47524.7 (37527.7 to 58270.9) | 26310.9 (20508.1 to 31274.3) | 21213.7 (16083.8 to 27193.4) | 292.3 (239.7 to 352.3) | 300.5 (227.6 to 368.7) | 282.5 (236.4 to 359.6) |
|  | Deaths | Age-standardized | 3.3 (2.4 to 4.1) | 4.1 (2.9 to 5.3) | 2.4 (1.6 to 2.9) | 3.3 (2.5 to 3.8) | 4.6 (3.3 to 5.3) | 2.2 (1.5 to 2.7) | -0.4 (-22.3 to 28.3) | 10.2 (-18.8 to 40.3) | -6.6 (-24 to 41.4) |
|  |  | All ages | 410.6 (305.9 to 509.9) | 266.4 (193.5 to 341.2) | 144.2 (97.9 to 176.6) | 1948.9 (1462.6 to 2233.1) | 1258.4 (920 to 1445.6) | 690.6 (478.6 to 823.5) | 374.6 (268.7 to 511.3) | 372.3 (246.1 to 502.3) | 378.7 (281.6 to 618.5) |
| Iraq | Incidence | Age-standardized | 36.6 (26.5 to 48.7) | 33 (24 to 44) | 40.4 (29.2 to 53.6) | 38.4 (28.1 to 51.2) | 34.4 (25.2 to 45.8) | 42.9 (31.2 to 58.3) | 4.9 (-0.8 to 11.2) | 4.2 (-2.2 to 10.5) | 6.1 (-1.8 to 15.2) |
|  |  | All ages | 2716.9 (2025.3 to 3569.6) | 1270.6 (942.1 to 1659.9) | 1446.4 (1072.1 to 1895.6) | 8150.8 (6210.3 to 10702.5) | 3761.8 (2831.3 to 4947.6) | 4389 (3338.6 to 5758.3) | 200 (177.9 to 223.6) | 196.1 (172.8 to 225) | 203.5 (176 to 231.4) |
|  | Prevalence | Age-standardized | 373.4 (285 to 485.8) | 333.7 (251.2 to 437) | 418.2 (317.7 to 547.6) | 406.1 (308.5 to 532) | 361.6 (273.1 to 471.7) | 459.7 (349.9 to 600.7) | 8.8 (3.7 to 14.3) | 8.3 (1.9 to 15.5) | 9.9 (2.5 to 17.6) |
|  |  | All ages | 26905.9 (20773.2 to 34732.7) | 12554.6 (9492.3 to 16404.2) | 14351.3 (10992.9 to 18572.4) | 78700.6 (61203.2 to 102059.9) | 36513.9 (28054.3 to 47836) | 42186.8 (32671 to 54683.5) | 192.5 (172.1 to 210.9) | 190.8 (170.6 to 212.9) | 194 (169.2 to 218.5) |
|  | DALYs (Disability-Adjusted Life Years) | Age-standardized | 76.5 (59.5 to 96.6) | 77.5 (59.7 to 100.1) | 75.3 (56.7 to 98.2) | 93.6 (74.2 to 114) | 97.1 (78 to 120.1) | 88.2 (67.2 to 113.4) | 22.5 (-2.4 to 65.8) | 25.3 (-5.3 to 72) | 17.1 (-7.3 to 71.5) |
|  |  | All ages | 5572.2 (4347.6 to 6977.9) | 2975.8 (2302.9 to 3821.8) | 2596.4 (1953.8 to 3346.8) | 16445.3 (13020.4 to 19937.7) | 8974.6 (7110.4 to 11078.1) | 7470.7 (5779 to 9283.1) | 195.1 (133.8 to 299.9) | 201.6 (128.4 to 316.3) | 187.7 (127.5 to 308.8) |
|  | Deaths | Age-standardized | 3.3 (2.3 to 4.4) | 3.5 (2.5 to 4.9) | 2.9 (1.9 to 4.6) | 4.9 (3.5 to 6.2) | 5.4 (3.9 to 7) | 4 (2.8 to 5.7) | 49.1 (5.9 to 132.1) | 53.6 (3.5 to 135.7) | 36.8 (-6.8 to 159.6) |
|  |  | All ages | 226.1 (161.2 to 304.3) | 134.3 (94.2 to 185.8) | 91.8 (60.3 to 139.4) | 647.1 (481.2 to 825.7) | 407.8 (299.6 to 522.8) | 239.2 (172.9 to 325.5) | 186.1 (99.6 to 350.9) | 203.6 (105.5 to 371.1) | 160.6 (77.2 to 392.4) |
| Jordan | Incidence | Age-standardized | 34.4 (25 to 46.2) | 30.8 (22.3 to 41.4) | 38 (27.7 to 51.3) | 37 (27.5 to 49.7) | 32.8 (24.3 to 44) | 41 (30.2 to 54.7) | 7.7 (3.2 to 13.1) | 6.2 (0 to 14.8) | 7.8 (1 to 15) |
|  |  | All ages | 385.7 (295 to 505.1) | 171.7 (128.5 to 225.7) | 214 (163.2 to 278.5) | 2471.6 (1878 to 3282.5) | 1042.8 (793.1 to 1394) | 1428.8 (1086.8 to 1852.4) | 540.8 (510.5 to 575.8) | 507.4 (469.3 to 563.3) | 567.6 (525.7 to 611) |
|  | Prevalence | Age-standardized | 354.2 (268.4 to 466.1) | 313.3 (233 to 411.1) | 394 (297.4 to 515) | 396.6 (300.6 to 520.3) | 346.8 (263.6 to 458.2) | 440 (331.9 to 574.6) | 12 (7.5 to 18.1) | 10.7 (3.9 to 20.2) | 11.7 (4.8 to 18.8) |
|  |  | All ages | 3648.8 (2816.1 to 4742.9) | 1600.1 (1222.1 to 2097.8) | 2048.7 (1572.2 to 2687.9) | 23983 (18450.5 to 31112) | 10018.2 (7633.2 to 13133.4) | 13964.8 (10761 to 18087.1) | 557.3 (532.6 to 594.2) | 526.1 (486.7 to 581.9) | 581.6 (540.2 to 631.7) |
|  | DALYs (Disability-Adjusted Life Years) | Age-standardized | 68.7 (55.4 to 84.6) | 76 (62.1 to 94) | 61.7 (47.2 to 78.5) | 63.1 (49.4 to 78.3) | 71.2 (56.4 to 87.4) | 57.6 (43.6 to 73.9) | -8.2 (-23.8 to 6.1) | -6.2 (-27.1 to 12.3) | -6.6 (-23.7 to 10.5) |
|  |  | All ages | 658.3 (525.7 to 813.4) | 352.6 (286.7 to 433.5) | 305.7 (228 to 389.2) | 3377 (2602.3 to 4222.8) | 1663.7 (1312 to 2050.5) | 1713.4 (1286.7 to 2240.3) | 413 (326.6 to 493.8) | 371.9 (270.1 to 471) | 460.5 (352.4 to 558.8) |
|  | Deaths | Age-standardized | 3.1 (2.5 to 3.8) | 4 (3.2 to 5) | 2.4 (1.7 to 3) | 2.7 (2.1 to 3.3) | 3.9 (2.9 to 4.8) | 1.9 (1.5 to 2.5) | -13.2 (-36 to 9.6) | -1.6 (-31.3 to 29.7) | -18.9 (-41.5 to 11) |
|  |  | All ages | 23.3 (19.1 to 28.3) | 14.3 (11.5 to 18) | 9.1 (6.7 to 11.6) | 102.5 (80.5 to 125.4) | 61.5 (47.1 to 76.7) | 41 (31.6 to 52.5) | 339.3 (222.3 to 456.8) | 330.9 (196.4 to 472) | 352.5 (223.7 to 525.3) |
| Kuwait | Incidence | Age-standardized | 35.5 (25.7 to 47.4) | 31.4 (23 to 42) | 38.9 (28.1 to 52.1) | 37.9 (27.9 to 50.9) | 33.1 (24.4 to 44) | 41.3 (30.4 to 55.5) | 6.8 (1.3 to 11.6) | 5.4 (-2.6 to 12.6) | 6.2 (-0.8 to 13.7) |
|  |  | All ages | 188.1 (147.4 to 241.5) | 64.4 (49.7 to 83.7) | 123.7 (96.4 to 157.6) | 1013.2 (804.2 to 1301.5) | 376.2 (294.2 to 484.6) | 637.1 (500.9 to 825.4) | 438.7 (407.7 to 466.8) | 484.3 (439.4 to 536.9) | 415 (372.2 to 456.2) |
|  | Prevalence | Age-standardized | 369.8 (282.8 to 483.4) | 326.5 (245.8 to 429.4) | 410.4 (312.2 to 533.5) | 408.1 (310.6 to 530.2) | 354.1 (268 to 463.7) | 447.3 (341.6 to 586.7) | 10.3 (4.9 to 14.9) | 8.5 (0.4 to 16.1) | 9 (2.2 to 15.9) |
|  |  | All ages | 1720.8 (1336.6 to 2199.1) | 614.5 (476.8 to 806.1) | 1106.3 (853.9 to 1419.9) | 9835.5 (7696.8 to 12638) | 3527.2 (2755.1 to 4533.9) | 6308.4 (4952.4 to 8095.6) | 471.6 (441.3 to 497.2) | 474 (436.4 to 520.6) | 470.2 (424.5 to 514.4) |
|  | DALYs (Disability-Adjusted Life Years) | Age-standardized | 62.3 (49.5 to 76.9) | 65.6 (52.1 to 79.4) | 60.2 (47.5 to 77.2) | 66.6 (52.8 to 82.2) | 60.5 (48.2 to 74) | 70.9 (55.9 to 89.2) | 6.9 (-2.9 to 17.4) | -7.7 (-18.9 to 5.9) | 17.8 (4.6 to 32.4) |
|  |  | All ages | 272.1 (216.5 to 338.6) | 123 (99.1 to 146.8) | 149 (114.9 to 192.8) | 1524.7 (1212.5 to 1889) | 580.6 (460.2 to 700.2) | 944.1 (744.7 to 1187.3) | 460.4 (407.3 to 517.3) | 371.9 (317.4 to 440.8) | 533.5 (458.2 to 630.6) |
|  | Deaths | Age-standardized | 2.6 (2.1 to 3) | 3 (2.3 to 3.5) | 2.3 (1.9 to 2.5) | 3 (2.4 to 3.6) | 2.9 (2.1 to 3.3) | 3.1 (2.4 to 3.8) | 15.9 (-1.2 to 32.8) | -3.7 (-19.4 to 16.5) | 37.3 (11.8 to 64.2) |
|  |  | All ages | 8.6 (7.1 to 9.6) | 5.1 (4 to 6) | 3.4 (3 to 3.8) | 60.3 (48.1 to 71.8) | 25.2 (18.8 to 29.8) | 35.1 (27.7 to 42.6) | 605.6 (501.8 to 712) | 391.1 (312 to 493.8) | 927.1 (737 to 1125.1) |
| Lebanon | Incidence | Age-standardized | 33.3 (24.8 to 44.9) | 30.1 (22.3 to 40.1) | 37.2 (27.6 to 49.8) | 36.2 (27 to 48.3) | 32.4 (24.2 to 42.9) | 40.7 (30.1 to 54.2) | 8.7 (2.8 to 14.3) | 7.6 (-0.4 to 17.1) | 9.3 (0.6 to 17.1) |
|  |  | All ages | 649.5 (494 to 854.8) | 309.9 (234 to 404.4) | 339.6 (256.9 to 449.5) | 2243.6 (1665.5 to 3000.9) | 1080.2 (803.4 to 1433.9) | 1163.4 (853.4 to 1553.7) | 245.4 (216.5 to 280) | 248.6 (222 to 283.4) | 242.6 (202.1 to 284.5) |
|  | Prevalence | Age-standardized | 342.3 (261.2 to 444.6) | 306.8 (232.5 to 402.7) | 385.9 (294.3 to 504.9) | 387.1 (298.5 to 511.1) | 343.9 (262.1 to 454.8) | 438 (336 to 576.7) | 13.1 (7.2 to 18.4) | 12.1 (4.1 to 21.6) | 13.5 (5 to 21.5) |
|  |  | All ages | 6336.1 (4938.9 to 8255.3) | 3019.8 (2315.3 to 3973.6) | 3316.3 (2565.1 to 4262.8) | 24591.1 (18725.1 to 32515.7) | 11779.5 (8922.9 to 15562.5) | 12811.5 (9737.4 to 16956.4) | 288.1 (260.4 to 320.2) | 290.1 (260.2 to 331.7) | 286.3 (251.9 to 325.7) |
|  | DALYs (Disability-Adjusted Life Years) | Age-standardized | 92.9 (60.7 to 132.4) | 99.9 (62.8 to 154) | 84.4 (48.9 to 130.3) | 68.9 (56.3 to 84.2) | 69.1 (55.6 to 85.5) | 68.5 (53.5 to 86.9) | -25.9 (-49.2 to 11.9) | -30.8 (-57.8 to 7.4) | -18.9 (-47.1 to 31.4) |
|  |  | All ages | 1614.2 (1080 to 2271.1) | 932.7 (595.3 to 1421.5) | 681.5 (401 to 1031.3) | 4546.2 (3720.4 to 5509.3) | 2496.6 (2020.5 to 3098.9) | 2049.6 (1599.9 to 2597.7) | 181.6 (94.6 to 318.8) | 167.7 (65 to 314.7) | 200.7 (99.7 to 391.5) |
|  | Deaths | Age-standardized | 5 (2.7 to 7.8) | 5.6 (3 to 9.8) | 4.1 (1.8 to 7.2) | 3.4 (2.8 to 4.4) | 3.7 (2.9 to 4.6) | 3 (2.3 to 4.5) | -31.3 (-57.6 to 33.1) | -33.7 (-65 to 24.8) | -26.4 (-60.5 to 74.6) |
|  |  | All ages | 72.7 (40.9 to 113.3) | 46.6 (25.2 to 80.7) | 26.2 (12 to 45.4) | 238.9 (192.7 to 307.8) | 144.3 (112.2 to 181) | 94.6 (74.2 to 141.3) | 228.5 (101.3 to 532) | 209.7 (64.3 to 491.3) | 261.8 (94.5 to 753.2) |
| Libya | Incidence | Age-standardized | 35.7 (26.1 to 47.9) | 31.9 (23 to 43.1) | 39.2 (29 to 52.2) | 37.7 (27.8 to 50.7) | 33.5 (24.9 to 45.1) | 41.8 (30.4 to 55.7) | 5.6 (0.2 to 11.2) | 4.9 (-2.4 to 12.1) | 6.6 (-0.4 to 14.1) |
|  |  | All ages | 614.5 (465.2 to 806.3) | 262.5 (195.4 to 351.3) | 351.9 (267.7 to 459.6) | 1776.9 (1361.2 to 2315.1) | 785.8 (604.1 to 1029) | 991 (755.1 to 1298) | 189.2 (173.1 to 207.4) | 199.3 (177.4 to 221.7) | 181.6 (164 to 203.3) |
|  | Prevalence | Age-standardized | 367.3 (276.5 to 479.7) | 325.1 (242.6 to 431.9) | 407.3 (307.7 to 534.6) | 398.3 (304.8 to 518.5) | 350.4 (264.3 to 459.2) | 446.6 (341.9 to 577.1) | 8.4 (3.3 to 13.7) | 7.8 (0.1 to 15.6) | 9.6 (3.5 to 16.6) |
|  |  | All ages | 6060.6 (4644.6 to 7874.7) | 2579.3 (1927.3 to 3396.6) | 3481.2 (2702.8 to 4515.1) | 17554.5 (13696.2 to 22623.3) | 7662.3 (5873.7 to 9986.9) | 9892.2 (7702.9 to 12632) | 189.7 (174.7 to 205.4) | 197.1 (175.2 to 219.5) | 184.2 (167.5 to 202.8) |
|  | DALYs (Disability-Adjusted Life Years) | Age-standardized | 60.2 (45.1 to 77.2) | 64.7 (46.9 to 83.9) | 56 (42.6 to 73.5) | 70.1 (50.4 to 92.3) | 79.5 (53 to 110.3) | 60.2 (44.2 to 77.9) | 16.4 (-6.6 to 48.4) | 22.9 (-11.2 to 65.7) | 7.5 (-13.7 to 40) |
|  |  | All ages | 977.8 (740.8 to 1254.6) | 511.2 (369 to 662.4) | 466.6 (351.4 to 612.8) | 2949.8 (2127.2 to 3911.1) | 1678.3 (1115.1 to 2327.7) | 1271.4 (923.6 to 1642.9) | 201.7 (141.8 to 284) | 228.3 (134 to 344.9) | 172.5 (119.8 to 252.2) |
|  | Deaths | Age-standardized | 2.5 (1.8 to 3.4) | 3 (1.9 to 4.2) | 2 (1.4 to 3) | 3.1 (1.9 to 4.5) | 4 (2.2 to 6.2) | 2.1 (1.4 to 3.2) | 23.7 (-19.8 to 83.2) | 34.6 (-22.6 to 101.6) | 3.9 (-35.9 to 80.3) |
|  |  | All ages | 38 (27.3 to 53.1) | 22.7 (14.4 to 32.3) | 15.3 (10.6 to 23.2) | 111.3 (68.6 to 163.2) | 74.9 (42 to 116.3) | 36.4 (24 to 55.6) | 192.8 (83.6 to 340.6) | 230.6 (84.6 to 401) | 137 (42.9 to 312.8) |
| Morocco | Incidence | Age-standardized | 35.7 (26 to 48) | 32.1 (23.8 to 43.5) | 39.2 (28.7 to 52.8) | 36.6 (26.9 to 49.6) | 32.6 (24.1 to 43.5) | 40.9 (29.9 to 56.6) | 2.5 (-2.2 to 7.5) | 1.7 (-5.6 to 7.9) | 4.2 (-2.9 to 12) |
|  |  | All ages | 4690.4 (3492.1 to 6273.4) | 2091.4 (1560.9 to 2809.9) | 2598.9 (1918.4 to 3437) | 11737.8 (8719.8 to 15855.7) | 5343.7 (4017.5 to 7082.9) | 6394 (4707.8 to 8692.9) | 150.3 (138.9 to 162.5) | 155.5 (136.5 to 171.3) | 146 (128.3 to 167) |
|  | Prevalence | Age-standardized | 360.2 (273.2 to 470.7) | 320.1 (240.1 to 424.9) | 401 (307.4 to 523.3) | 383.3 (292.5 to 506) | 338.4 (257.6 to 443.5) | 432.4 (329.3 to 571.9) | 6.4 (1.7 to 11.3) | 5.7 (-2.3 to 11.9) | 7.8 (0.7 to 14.9) |
|  |  | All ages | 45335.3 (34423.4 to 58980.3) | 20009 (15086.3 to 26393.4) | 25326.3 (19233.2 to 32452.6) | 117079.3 (89915.8 to 151799.8) | 53213.3 (40503.1 to 69685) | 63866 (49170.4 to 83435.9) | 158.3 (146.7 to 170.8) | 165.9 (146.8 to 181.7) | 152.2 (134.4 to 170.6) |
|  | DALYs (Disability-Adjusted Life Years) | Age-standardized | 67.3 (48.4 to 86.6) | 69.5 (48.5 to 93.7) | 65.5 (43.6 to 88.2) | 83.6 (65.6 to 104.3) | 89 (66.1 to 118.4) | 77.7 (59.3 to 97.7) | 24.1 (3.5 to 49.3) | 28.2 (0.6 to 55.7) | 18.5 (-2.9 to 51.4) |
|  |  | All ages | 7963.6 (5787.8 to 10135) | 4141.2 (2914.5 to 5456.6) | 3822.4 (2611.3 to 4996.9) | 23354.5 (18286.7 to 29438.3) | 12872 (9639.2 to 17118.3) | 10482.5 (8060.8 to 13252.3) | 193.3 (146.3 to 245.9) | 210.8 (145.5 to 277) | 174.2 (129.7 to 238.9) |
|  | Deaths | Age-standardized | 3 (1.8 to 4.2) | 3.3 (1.9 to 4.9) | 2.8 (1.3 to 4.4) | 4.3 (3 to 5.5) | 4.9 (3.3 to 6.9) | 3.6 (2.6 to 4.6) | 40.7 (4.4 to 96.3) | 48.6 (2.4 to 97.7) | 27.6 (-14.6 to 128.3) |
|  |  | All ages | 303.9 (182.9 to 416.6) | 171.7 (103.4 to 251.2) | 132.3 (63.5 to 197.9) | 991.4 (711.3 to 1279.9) | 603.1 (410.5 to 866) | 388.3 (289.3 to 495.4) | 226.2 (144.9 to 344.1) | 251.3 (149.6 to 364.6) | 193.5 (108.4 to 399.5) |
| Oman | Incidence | Age-standardized | 29 (21.4 to 38.7) | 26.2 (19.5 to 35) | 32.2 (23.6 to 43) | 34.4 (25.3 to 46.3) | 30.7 (22.6 to 41.5) | 38.2 (28 to 51.1) | 18.8 (12.6 to 25.3) | 17.3 (10.2 to 25.3) | 18.5 (10.1 to 27.2) |
|  |  | All ages | 176.3 (134.3 to 226.5) | 73 (54.9 to 96.3) | 103.3 (78.8 to 133) | 643.4 (503.6 to 828.9) | 243.4 (182.9 to 319.8) | 400.1 (310.3 to 521.8) | 265 (239.6 to 291.2) | 233.5 (206.1 to 260.3) | 287.3 (255.7 to 319.2) |
|  | Prevalence | Age-standardized | 292.9 (225.1 to 380.8) | 264 (200.7 to 344) | 331 (250.1 to 429.5) | 365.3 (276.4 to 482.9) | 324.6 (244.4 to 430.1) | 409.8 (311.2 to 536.7) | 24.7 (18 to 30.3) | 23 (15.9 to 31.1) | 23.8 (15 to 32) |
|  |  | All ages | 1615.2 (1248 to 2101.2) | 706.3 (537.9 to 918.9) | 909 (696.4 to 1188.6) | 5827.2 (4557.2 to 7636.5) | 2310.9 (1756.4 to 3014.4) | 3516.3 (2743.5 to 4648.9) | 260.8 (238.3 to 281.7) | 227.2 (204.2 to 252.8) | 286.8 (255.8 to 313.9) |
|  | DALYs (Disability-Adjusted Life Years) | Age-standardized | 80 (60.9 to 102.8) | 84.1 (63.2 to 115.6) | 80.1 (55.7 to 108) | 91 (74.6 to 109.2) | 90.3 (71.1 to 110.3) | 94.3 (76.7 to 114) | 13.7 (-17 to 54.3) | 7.4 (-28.3 to 48.5) | 17.8 (-15.7 to 79) |
|  |  | All ages | 412.8 (309.7 to 525.7) | 222 (167 to 304.7) | 190.9 (132.8 to 260.5) | 1236.6 (991.4 to 1517.6) | 590.8 (467.1 to 711.4) | 645.8 (491.2 to 854.2) | 199.5 (110.3 to 297.2) | 166.2 (73.9 to 268.2) | 238.4 (119.7 to 404.6) |
|  | Deaths | Age-standardized | 4 (2.9 to 5.6) | 4.2 (2.9 to 6.3) | 4.1 (2.6 to 6) | 4.7 (3.6 to 5.9) | 4.8 (3.5 to 6.1) | 5 (3.5 to 6.6) | 17.4 (-22.7 to 82.3) | 12.9 (-34.4 to 78.6) | 19.8 (-27.7 to 137.3) |
|  |  | All ages | 16.6 (12 to 23) | 10.3 (7.1 to 15.3) | 6.3 (3.8 to 9.4) | 45.3 (35.8 to 55.2) | 25.8 (19.6 to 32.4) | 19.6 (15.4 to 24.5) | 173.1 (76.8 to 314.1) | 150.9 (46.6 to 289.6) | 209.2 (85.5 to 458.9) |
| Pakistan | Incidence | Age-standardized | 53.3 (39.6 to 71.1) | 47 (35.3 to 62.9) | 58 (43.5 to 77.4) | 53.8 (40.3 to 71.6) | 49.8 (37.3 to 66.2) | 57.5 (43 to 76.3) | 1.1 (-2 to 4.5) | 5.8 (1.9 to 10.4) | -0.9 (-5.6 to 4.4) |
|  |  | All ages | 27496.9 (20886.9 to 36777.3) | 10523.5 (7960.1 to 14088.1) | 16973.4 (12698.8 to 22554.4) | 57873.9 (44288.7 to 75860.6) | 25397.2 (19516.2 to 33388.4) | 32476.8 (24823.1 to 42728) | 110.5 (100.3 to 121.2) | 141.3 (129.5 to 153.6) | 91.3 (78.4 to 104.7) |
|  | Prevalence | Age-standardized | 554.7 (429.4 to 728.7) | 473.4 (363.1 to 625.5) | 615.2 (477.9 to 803.8) | 558.6 (429.5 to 735.7) | 503.7 (386.1 to 664.7) | 608.5 (467.5 to 801.8) | 0.7 (-2.6 to 4) | 6.4 (2.2 to 11) | -1.1 (-6 to 3.7) |
|  |  | All ages | 270729 (206527.9 to 355785.2) | 99828.4 (76761.7 to 132225.4) | 170900.6 (130445.9 to 223906) | 548173.6 (427140.9 to 726636.8) | 236096.7 (183830.9 to 313314.4) | 312076.9 (242287.9 to 413910) | 102.5 (93.5 to 111.9) | 136.5 (125.2 to 147.6) | 82.6 (71.9 to 93.7) |
|  | DALYs (Disability-Adjusted Life Years) | Age-standardized | 79.5 (57.9 to 106.3) | 76.9 (55.7 to 110.5) | 81.6 (56.2 to 106.4) | 101.5 (79.7 to 131.7) | 103.4 (77.3 to 140.8) | 99.8 (73.5 to 129.1) | 27.6 (10.2 to 50.8) | 34.4 (8.4 to 69.5) | 22.3 (2.2 to 48.3) |
|  |  | All ages | 36868.2 (26780.4 to 49761.6) | 15204.4 (11051.6 to 21727.2) | 21663.8 (14908.5 to 28526.5) | 90387.2 (69208.3 to 116432.2) | 43286.7 (32096.3 to 59689.4) | 47100.6 (34616.1 to 61263.2) | 145.2 (111 to 185.9) | 184.7 (133.3 to 253.8) | 117.4 (84.7 to 161.6) |
|  | Deaths | Age-standardized | 2.8 (1.8 to 4.1) | 3.1 (1.9 to 5.3) | 2.6 (1.4 to 3.7) | 4.6 (3.3 to 6.4) | 5 (3.5 to 7.6) | 4.2 (2.7 to 5.9) | 63.6 (22.5 to 126.3) | 64.7 (12.9 to 138.9) | 59.1 (9.6 to 142.2) |
|  |  | All ages | 1035.2 (659.7 to 1504.3) | 483.5 (302.8 to 823.9) | 551.7 (288 to 780.5) | 3114.8 (2310.5 to 4332.2) | 1649.7 (1146.7 to 2527.5) | 1465.1 (941.5 to 2053.4) | 200.9 (128.3 to 317.2) | 241.2 (138.2 to 393) | 165.6 (87 to 299.6) |
| Palestine | Incidence | Age-standardized | 32 (23.5 to 42.9) | 29 (21.4 to 39) | 35.6 (26.4 to 48.5) | 33.2 (24.4 to 44.7) | 29.9 (22 to 39.7) | 37.4 (27.5 to 50.3) | 3.8 (-0.3 to 8.7) | 3.1 (-3.7 to 10.4) | 5 (-1.2 to 12.4) |
|  |  | All ages | 252.3 (188.7 to 337.7) | 126.1 (94.1 to 168.8) | 126.2 (93.6 to 172.8) | 748.6 (570.4 to 981.6) | 353.7 (270.6 to 469.6) | 394.9 (299.4 to 522.5) | 196.7 (180.4 to 217.4) | 180.5 (160.1 to 200.8) | 212.9 (187.6 to 244.3) |
|  | Prevalence | Age-standardized | 323.2 (245 to 422.7) | 292.1 (220 to 382) | 366.1 (278 to 484.8) | 347.4 (265.2 to 454) | 311.9 (237.7 to 411.2) | 397.3 (303.5 to 526.2) | 7.5 (3 to 12.1) | 6.8 (-0.5 to 13.5) | 8.5 (2.9 to 15.2) |
|  |  | All ages | 2436.6 (1858.8 to 3179.3) | 1220 (913.6 to 1593) | 1216.6 (936.6 to 1584.1) | 7196.7 (5524.6 to 9404.9) | 3443.1 (2642.1 to 4502.7) | 3753.6 (2873.5 to 4911.3) | 195.4 (179.5 to 213.7) | 182.2 (162.2 to 202.3) | 208.5 (185.5 to 236.6) |
|  | DALYs (Disability-Adjusted Life Years) | Age-standardized | 87.1 (67.4 to 108.7) | 85.4 (63.9 to 110.8) | 91.1 (64.9 to 117.5) | 89.8 (74.3 to 107.6) | 90.3 (73.8 to 109.9) | 93.2 (69.8 to 125.2) | 3.1 (-16.6 to 29.1) | 5.8 (-19.4 to 40.3) | 2.3 (-19.3 to 32.1) |
|  |  | All ages | 582.9 (451.5 to 730.7) | 331.3 (248.9 to 427.2) | 251.6 (183.1 to 317.9) | 1522.4 (1248.7 to 1837.6) | 877 (717.7 to 1063.3) | 645.4 (503.3 to 817.5) | 161.2 (110.4 to 223.8) | 164.7 (101.7 to 248.4) | 156.5 (107 to 227.8) |
|  | Deaths | Age-standardized | 5.1 (3.7 to 6.7) | 5 (3.6 to 6.9) | 5.4 (3.4 to 7.8) | 5.4 (4.3 to 6.7) | 5.5 (4.4 to 6.7) | 5.6 (3.8 to 8.6) | 5.1 (-20 to 43.1) | 9 (-21.7 to 58.6) | 4.9 (-25.5 to 48.3) |
|  |  | All ages | 28.5 (21 to 37.1) | 17.4 (12.6 to 23.9) | 11.1 (7.3 to 15) | 68.1 (55.2 to 82) | 44.9 (35.8 to 54.5) | 23.3 (17 to 31.1) | 139.1 (80.9 to 228.4) | 157.7 (84.7 to 274.7) | 110 (51.2 to 213.1) |
| Qatar | Incidence | Age-standardized | 35.2 (25.6 to 47) | 31 (22.8 to 41.6) | 38.2 (27.8 to 51.4) | 37.6 (27.6 to 50.6) | 32.7 (24.6 to 43.8) | 41.1 (29.8 to 55.6) | 6.8 (0.8 to 11.9) | 5.4 (-2.2 to 13.2) | 7.7 (1 to 16.3) |
|  |  | All ages | 36.3 (28.6 to 46.1) | 9.3 (7.3 to 12.1) | 27 (20.9 to 34.7) | 365.7 (282.6 to 474.3) | 91 (71.1 to 117.8) | 274.7 (209.7 to 354.6) | 906.6 (854.6 to 968.2) | 875.6 (809.8 to 947) | 917.3 (854.1 to 992.1) |
|  | Prevalence | Age-standardized | 363.5 (278.2 to 475.5) | 318 (242.7 to 414.3) | 398.3 (303.1 to 525.3) | 402.6 (305.8 to 529.3) | 349.7 (262.6 to 464.3) | 444.3 (334.1 to 586.4) | 10.8 (5.6 to 15.8) | 10 (2.5 to 18.8) | 11.5 (4.8 to 19.7) |
|  |  | All ages | 298.1 (228.1 to 389) | 80.1 (61.7 to 103.9) | 218 (165.2 to 288.2) | 3005 (2251.2 to 3953.1) | 777.1 (597.5 to 1013.1) | 2227.9 (1652.1 to 2974.8) | 908.2 (856.1 to 964.4) | 870.4 (802.9 to 946.3) | 922 (853.7 to 1001.3) |
|  | DALYs (Disability-Adjusted Life Years) | Age-standardized | 116.2 (65.7 to 150.1) | 138.3 (69.8 to 189.1) | 94.1 (55.4 to 131.3) | 78.5 (45.5 to 103.7) | 88.9 (42 to 126.2) | 65.8 (46.4 to 91) | -32.5 (-47.4 to -15) | -35.7 (-52.1 to -19.4) | -30.1 (-50.6 to -0.4) |
|  |  | All ages | 62.1 (41.1 to 78.2) | 27 (14.8 to 37) | 35.1 (23.8 to 46.6) | 407.6 (298 to 555.1) | 148.5 (90.1 to 200.3) | 259.1 (168.9 to 380.5) | 556.7 (391 to 812) | 449.8 (311.6 to 653.8) | 639 (393.4 to 987.9) |
|  | Deaths | Age-standardized | 7.4 (3.2 to 10) | 9.2 (3.6 to 13.3) | 5.1 (2.3 to 8.1) | 4.1 (1.5 to 6) | 5.1 (1.4 to 8) | 2.6 (1.3 to 5) | -44.9 (-61.9 to -27.3) | -44.4 (-68.1 to -25.6) | -48.8 (-66.3 to -22.7) |
|  |  | All ages | 2.3 (1.1 to 3.1) | 1.4 (0.6 to 2) | 0.9 (0.5 to 1.4) | 10 (5.4 to 15) | 5.9 (2 to 9) | 4.1 (2.5 to 6.5) | 331.2 (185.3 to 544.3) | 329.8 (175 to 470) | 333.4 (122.9 to 828.9) |
| Saudi Arabia | Incidence | Age-standardized | 32.7 (24.2 to 44.1) | 29.1 (21.5 to 38.9) | 35.7 (26.4 to 48.4) | 36.9 (27.2 to 49.8) | 32.1 (23.8 to 43) | 40.1 (29.5 to 54.1) | 13 (8.1 to 19.3) | 10.5 (2.8 to 19.4) | 12.2 (5.1 to 20.4) |
|  |  | All ages | 1685.5 (1283.8 to 2222.9) | 633.4 (475.6 to 839.3) | 1052.1 (804.7 to 1379.3) | 6095.8 (4757.1 to 7921.4) | 2069.2 (1593.7 to 2712) | 4026.6 (3126.7 to 5193.3) | 261.7 (227 to 300.8) | 226.7 (186.6 to 270.8) | 282.7 (247.1 to 327.7) |
|  | Prevalence | Age-standardized | 330.7 (250.3 to 435.2) | 290.9 (221.6 to 380.9) | 366.1 (278.4 to 481.2) | 391.9 (301.9 to 511.7) | 338.8 (256.8 to 445.4) | 429.6 (326.8 to 558) | 18.5 (13.7 to 25) | 16.5 (8.5 to 25.3) | 17.3 (9.8 to 25.4) |
|  |  | All ages | 15737.4 (12130.4 to 20449.7) | 6000.9 (4578.3 to 7828.1) | 9736.5 (7540.1 to 12663.5) | 54167 (42124.9 to 71479.1) | 18072.8 (13872.5 to 23966.4) | 36094.2 (27819.9 to 47627.7) | 244.2 (214.6 to 278.2) | 201.2 (166.7 to 237.4) | 270.7 (232.6 to 311.4) |
|  | DALYs (Disability-Adjusted Life Years) | Age-standardized | 64 (50.2 to 81.4) | 70 (53.2 to 91.4) | 58.8 (45.1 to 77.7) | 72.2 (58.2 to 88.6) | 83.9 (65.2 to 101.6) | 63.2 (48.9 to 80.2) | 12.8 (-11 to 39.4) | 19.8 (-17.4 to 56.9) | 7.6 (-15.1 to 35.3) |
|  |  | All ages | 2831.3 (2244.6 to 3620.4) | 1389.3 (1054.9 to 1798) | 1442 (1094.8 to 1926.2) | 9049.2 (7157.3 to 11409) | 4112.3 (3182.8 to 5123.5) | 4936.8 (3746.4 to 6552.8) | 219.6 (151.8 to 300.3) | 196 (100.8 to 287.8) | 242.4 (165.9 to 334.4) |
|  | Deaths | Age-standardized | 3 (2.3 to 4.1) | 3.5 (2.6 to 5.1) | 2.4 (1.7 to 3.5) | 3.4 (2.7 to 4.1) | 4.5 (3.3 to 5.7) | 2.4 (1.8 to 3.2) | 12.7 (-22 to 54.4) | 26.9 (-23.6 to 82.7) | -0.1 (-33.4 to 53.9) |
|  |  | All ages | 102.4 (78.2 to 142) | 61.1 (44.5 to 88.1) | 41.2 (29.1 to 59.9) | 248.9 (197.4 to 305.4) | 145 (106.4 to 183.5) | 103.9 (77 to 135.7) | 143.1 (65.3 to 236.4) | 137.2 (39 to 242.9) | 151.8 (60.6 to 288.5) |
| Somalia | Incidence | Age-standardized | 34.1 (26 to 45.4) | 34.1 (26 to 45.4) | 34.3 (26 to 45.5) | 35.6 (27.4 to 47.3) | 35.5 (27.4 to 46.7) | 35.8 (27.4 to 48.1) | 4.1 (-0.1 to 8.6) | 4.2 (-2.5 to 10.9) | 4.4 (-2.2 to 10.2) |
|  |  | All ages | 725.7 (577.8 to 944.7) | 382.4 (303 to 498.2) | 343.3 (273.6 to 452.6) | 1950.9 (1521.5 to 2560.4) | 1112.8 (857.9 to 1455.1) | 838.1 (649.5 to 1096.5) | 168.8 (151.1 to 184.8) | 191 (169.3 to 216.7) | 144.1 (126.5 to 163.1) |
|  | Prevalence | Age-standardized | 374.4 (294.5 to 490) | 369.9 (289.5 to 484.9) | 380.6 (301.4 to 495.4) | 392 (308.4 to 516.9) | 388.5 (302.7 to 514.1) | 400.5 (313.2 to 526.7) | 4.7 (0.3 to 9.8) | 5 (-1 to 11.7) | 5.2 (-1.9 to 11.9) |
|  |  | All ages | 6910.3 (5439.3 to 8954.2) | 3650.3 (2860 to 4695.4) | 3260 (2556.4 to 4209.8) | 18687.3 (14346.6 to 24314.4) | 10906.2 (8318.8 to 14271.9) | 7781.1 (5936 to 10138.6) | 170.4 (156.8 to 185.6) | 198.8 (180.1 to 220.4) | 138.7 (120.5 to 156.3) |
|  | DALYs (Disability-Adjusted Life Years) | Age-standardized | 70.1 (43.6 to 99.6) | 68.2 (45.1 to 105.7) | 72.4 (38.4 to 101.8) | 63.6 (39.5 to 97.4) | 61.8 (39.4 to 101) | 66.3 (36 to 100.9) | -9.2 (-22.8 to 8) | -9.4 (-25.8 to 10.4) | -8.4 (-25.4 to 9.6) |
|  |  | All ages | 1182.1 (742.1 to 1681.7) | 606.8 (403.4 to 933.9) | 575.3 (316.2 to 811.8) | 2767.5 (1735.5 to 4169) | 1562.4 (996 to 2489.9) | 1205.1 (659.7 to 1841.7) | 134.1 (98.3 to 183.8) | 157.5 (106.1 to 212.1) | 109.5 (64.9 to 160) |
|  | Deaths | Age-standardized | 2.8 (1.3 to 4.5) | 2.7 (1.4 to 5.2) | 2.9 (1 to 4.9) | 2.2 (1 to 4) | 2.2 (1.1 to 4.4) | 2.3 (0.7 to 4.2) | -20 (-40.6 to 7.9) | -19.5 (-44.6 to 11.5) | -20.9 (-44.2 to 3.7) |
|  |  | All ages | 31.7 (14.8 to 51.4) | 16.6 (8.7 to 30.9) | 15.1 (5.1 to 24.3) | 60.9 (26 to 110.8) | 35.6 (17.4 to 71.8) | 25.3 (6.8 to 47) | 91.8 (37.5 to 162.9) | 114.1 (43.1 to 203.8) | 67.3 (10 to 134.5) |
| Sudan | Incidence | Age-standardized | 33.7 (24.6 to 45) | 30.3 (22.3 to 40.8) | 37.1 (26.9 to 49.2) | 37.8 (27.5 to 51) | 33.4 (24.5 to 44.7) | 41.4 (30.2 to 55.7) | 12.1 (6.9 to 18.1) | 10.4 (3 to 17.8) | 11.8 (4.1 to 20.2) |
|  |  | All ages | 2799.6 (2050.3 to 3759.2) | 1202.1 (882.9 to 1623.7) | 1597.5 (1163.4 to 2141.1) | 6613.9 (4970.4 to 8738.6) | 2675.5 (2017.2 to 3528.6) | 3938.4 (2936.4 to 5238.6) | 136.2 (121.7 to 151.6) | 122.6 (103.8 to 143.4) | 146.5 (127.6 to 167.3) |
|  | Prevalence | Age-standardized | 336.4 (256.3 to 438.2) | 298.1 (225.2 to 393.9) | 374.8 (284.3 to 486.8) | 394.5 (298.4 to 515.7) | 344.1 (258.2 to 452.7) | 437.1 (331.1 to 573) | 17.3 (11.9 to 23.2) | 15.4 (7.6 to 23.4) | 16.6 (9.3 to 25.3) |
|  |  | All ages | 26218.6 (19909.2 to 34244.9) | 11232.3 (8450.8 to 14794.2) | 14986.3 (11356.6 to 19496.3) | 63916.4 (49241.7 to 83109.6) | 25242.2 (19254.3 to 33337.7) | 38674.2 (29612.8 to 49851) | 143.8 (130 to 156.4) | 124.7 (107 to 143.2) | 158.1 (139.2 to 176.4) |
|  | DALYs (Disability-Adjusted Life Years) | Age-standardized | 64.8 (46.1 to 88.6) | 69.3 (47.6 to 105.9) | 60.7 (39.1 to 82.3) | 72.5 (56.1 to 90.4) | 78.7 (56.3 to 101.5) | 67.5 (51.7 to 85.8) | 12 (-10.7 to 43) | 13.7 (-16.2 to 49.6) | 11.2 (-12.8 to 47.7) |
|  |  | All ages | 4645.6 (3339.6 to 6249.6) | 2436.3 (1690.5 to 3662.3) | 2209.4 (1453.1 to 3002) | 10967.1 (8323.7 to 13688.8) | 5406.5 (3854.9 to 6957.8) | 5560.7 (4156.9 to 6986.6) | 136.1 (89.9 to 199.9) | 121.9 (64.2 to 190.9) | 151.7 (98 to 229.8) |
|  | Deaths | Age-standardized | 2.9 (1.8 to 4.2) | 3.3 (1.9 to 5.7) | 2.5 (1.2 to 3.8) | 3.2 (2.3 to 4.2) | 3.9 (2.6 to 5.3) | 2.7 (1.8 to 3.7) | 12.6 (-18.7 to 69) | 18.2 (-22.9 to 72.6) | 9.2 (-26.9 to 99.1) |
|  |  | All ages | 155.1 (97.8 to 231.6) | 91.9 (54.5 to 161.2) | 63.2 (31.1 to 98.9) | 394.5 (287.3 to 518.9) | 220.4 (142.4 to 300.9) | 174.1 (119.4 to 234.4) | 154.3 (82.2 to 286.7) | 139.9 (58.6 to 259.1) | 175.2 (81.7 to 398.6) |
| Syrian Arab Republic | Incidence | Age-standardized | 34.1 (25 to 45.5) | 30.6 (22.2 to 41.2) | 37.3 (27.6 to 49.9) | 35.3 (25.7 to 47.1) | 31.1 (22.8 to 41.5) | 39.1 (28.5 to 52.3) | 3.4 (-1 to 7.6) | 1.9 (-4.4 to 9.2) | 4.6 (-2 to 12.1) |
|  |  | All ages | 1567.3 (1191.3 to 2036.6) | 667.2 (498.2 to 883.8) | 900.1 (685.7 to 1171.4) | 4228.1 (3162.9 to 5673.7) | 1836.7 (1379.7 to 2463.1) | 2391.4 (1787.2 to 3187.8) | 169.8 (154.5 to 184.8) | 175.3 (153.5 to 194.4) | 165.7 (148.2 to 186) |
|  | Prevalence | Age-standardized | 348.4 (264 to 453.9) | 307.6 (230.4 to 405.5) | 384.4 (292.4 to 501.1) | 377.8 (288.1 to 497.3) | 327.4 (250.9 to 430.3) | 416.7 (317.9 to 547.8) | 8.5 (3.9 to 12.8) | 6.4 (0.4 to 13.7) | 8.4 (1.8 to 15.4) |
|  |  | All ages | 15094.7 (11604.8 to 19661.4) | 6287.2 (4796.5 to 8298.5) | 8807.5 (6767.7 to 11416.1) | 41606 (32219.9 to 54570.1) | 17599.7 (13550.2 to 23401.2) | 24006.4 (18328.6 to 31424.7) | 175.6 (161.6 to 188.1) | 179.9 (161.8 to 200.5) | 172.6 (155.8 to 190.8) |
|  | DALYs (Disability-Adjusted Life Years) | Age-standardized | 79.9 (62.6 to 101.4) | 94.1 (70.9 to 123.9) | 67.9 (49.9 to 87.2) | 85.7 (63.8 to 105.2) | 113.4 (75 to 149.4) | 73.4 (55.3 to 90.9) | 7.3 (-15 to 39) | 20.5 (-13.3 to 57.6) | 8.1 (-12.6 to 46.6) |
|  |  | All ages | 3246.6 (2558.5 to 4087.5) | 1752.4 (1330.8 to 2278.7) | 1494.2 (1093 to 1914.8) | 8108.2 (6087.1 to 10069) | 4281.7 (2950 to 5489.8) | 3826.5 (2908.8 to 4787.1) | 149.7 (99.8 to 218) | 144.3 (80.5 to 220.9) | 156.1 (105.7 to 233.5) |
|  | Deaths | Age-standardized | 4.3 (3 to 5.6) | 5.7 (3.8 to 7.8) | 3.1 (2 to 4) | 4.6 (2.9 to 5.9) | 7.7 (4.6 to 10.5) | 3.3 (2.2 to 4.4) | 7.4 (-28.3 to 59.2) | 34.1 (-13.1 to 91.9) | 9 (-27.7 to 92.3) |
|  |  | All ages | 152.5 (109 to 200.6) | 92.9 (61.7 to 127.4) | 59.6 (39.3 to 79.7) | 318.3 (209.6 to 419.4) | 187 (110.6 to 266.7) | 131.3 (91.4 to 171.4) | 108.7 (44.8 to 207.7) | 101.2 (26.4 to 197.5) | 120.3 (52.4 to 279.9) |
| Tunisia | Incidence | Age-standardized | 32.9 (24.1 to 44.5) | 29.5 (21.5 to 39.3) | 36.3 (26.2 to 49.4) | 34.5 (25 to 45.6) | 30.9 (22.5 to 40.7) | 38.6 (28.1 to 51.5) | 4.7 (0.2 to 10.3) | 4.6 (-1.6 to 11.2) | 6.4 (-0.9 to 15.5) |
|  |  | All ages | 1485.1 (1093.9 to 2018.1) | 641.8 (475.5 to 857.9) | 843.3 (609.8 to 1154.1) | 4413.6 (3251.2 to 5837.6) | 2076 (1527.7 to 2745.4) | 2337.6 (1718.5 to 3104.6) | 197.2 (181.7 to 219) | 223.4 (201.8 to 250.4) | 177.2 (156 to 205.7) |
|  | Prevalence | Age-standardized | 335.7 (257.8 to 444.2) | 299.9 (226.4 to 394.4) | 375.4 (287.9 to 491.7) | 365 (276.8 to 471.6) | 325.2 (243.8 to 421.9) | 413.1 (313.6 to 534.1) | 8.7 (4.5 to 13.7) | 8.4 (2.7 to 15) | 10 (3.4 to 18.5) |
|  |  | All ages | 14174.5 (10853.5 to 18659.1) | 6118.2 (4644.1 to 8056.8) | 8056.3 (6122.5 to 10639.2) | 45068.6 (34412.2 to 58372.3) | 21340.3 (15960.1 to 27603.6) | 23728.4 (18292.8 to 30632.1) | 218 (202.5 to 237.7) | 248.8 (226.1 to 274.1) | 194.5 (175.2 to 217.4) |
|  | DALYs (Disability-Adjusted Life Years) | Age-standardized | 70.9 (55.6 to 87.8) | 74.7 (52.2 to 96.6) | 65 (46.8 to 86.7) | 81.8 (60.5 to 107.5) | 82.4 (56.5 to 113.9) | 82 (59.9 to 107.2) | 15.5 (-10.6 to 45.7) | 10.2 (-18.9 to 42.6) | 26.1 (-6.8 to 75.1) |
|  |  | All ages | 2561 (1966.7 to 3177.3) | 1324.5 (951.7 to 1696.6) | 1236.5 (905.3 to 1578.1) | 9283.2 (6895.4 to 12246.5) | 5169.3 (3554 to 7089) | 4114 (3042 to 5309.9) | 262.5 (186.7 to 354.3) | 290.3 (190.1 to 399.8) | 232.7 (165.1 to 331.9) |
|  | Deaths | Age-standardized | 3.6 (2.6 to 4.6) | 4.1 (2.6 to 5.6) | 2.9 (1.8 to 4.3) | 4.6 (3 to 6.7) | 4.8 (2.8 to 7.4) | 4.5 (2.9 to 6.7) | 27.1 (-16.9 to 79.9) | 16.9 (-26.7 to 67) | 56.4 (-10.5 to 194.2) |
|  |  | All ages | 93.6 (69 to 117.1) | 55.2 (36.4 to 74.7) | 38.4 (25.4 to 55.3) | 462.5 (305.1 to 649.1) | 282.8 (168.8 to 433.3) | 179.7 (121.9 to 256.7) | 394.4 (223.8 to 593.8) | 412.4 (220.4 to 634.1) | 368.5 (195.3 to 666.6) |
| United Arab Emirates | Incidence | Age-standardized | 37 (27.3 to 49.4) | 32.8 (24.6 to 44) | 40.3 (29.2 to 53.9) | 39.7 (29.2 to 52.8) | 33.1 (24.5 to 44.6) | 41.2 (30.1 to 54.6) | 7.1 (2.4 to 13) | 0.9 (-6.3 to 8.9) | 2.2 (-3.6 to 8.6) |
|  |  | All ages | 155.1 (120.1 to 198.6) | 44.2 (33.6 to 57.3) | 111 (84.6 to 144.6) | 1662 (1236.1 to 2172.9) | 305.1 (229.8 to 412.2) | 1357 (1001.6 to 1756.7) | 971.3 (846.2 to 1098.9) | 590.4 (475 to 726.7) | 1122.9 (984.4 to 1271.6) |
|  | Prevalence | Age-standardized | 381.7 (289.4 to 495) | 333 (249.2 to 433.9) | 418.3 (315.8 to 548.3) | 424.7 (326.5 to 554.8) | 347.4 (266 to 458.8) | 440.6 (337.3 to 572.5) | 11.3 (6.2 to 16.8) | 4.3 (-3.1 to 12.6) | 5.3 (0 to 11.1) |
|  |  | All ages | 1293.7 (989.6 to 1673.8) | 394.5 (300.5 to 510.9) | 899.1 (672.1 to 1192.5) | 13474.7 (9821 to 18266.3) | 2310.2 (1710.7 to 3127) | 11164.5 (8037.4 to 15121.3) | 941.6 (814.5 to 1068.1) | 485.6 (375.2 to 597.4) | 1141.7 (1010 to 1286.9) |
|  | DALYs (Disability-Adjusted Life Years) | Age-standardized | 79.5 (60.3 to 104.9) | 86.6 (58.7 to 124.7) | 74.1 (54.4 to 97.4) | 85.8 (66.1 to 105.2) | 232 (162.2 to 285.6) | 57.4 (40.7 to 74.2) | 7.9 (-15.2 to 30.2) | 168 (85.6 to 251) | -22.6 (-37.3 to 0.1) |
|  |  | All ages | 231.9 (170.6 to 301.4) | 98.1 (69.2 to 137.8) | 133.8 (93.4 to 182.5) | 1763.2 (1272.6 to 2342) | 551.8 (429.8 to 673.5) | 1211.4 (794.3 to 1692.2) | 660.3 (477 to 846.5) | 462.3 (280.1 to 625.1) | 805.5 (555.1 to 1071) |
|  | Deaths | Age-standardized | 3.6 (2.6 to 5.2) | 4.1 (2.1 to 6.4) | 3.3 (2.1 to 4.5) | 3.8 (2.5 to 4.9) | 15.7 (10.2 to 19.8) | 1.8 (1.1 to 2.6) | 5.6 (-30.4 to 47.1) | 287.9 (144.6 to 477.9) | -44.1 (-69 to 4.7) |
|  |  | All ages | 6.8 (4.8 to 9.7) | 3.7 (2.1 to 5.8) | 3.1 (2 to 4.3) | 30.1 (23.5 to 37.4) | 17.9 (13.3 to 22.4) | 12.3 (9.3 to 16.7) | 345 (200.9 to 493.6) | 382.7 (199.6 to 596.9) | 299.5 (179.3 to 538.5) |
| Yemen | Incidence | Age-standardized | 31 (22.9 to 41.5) | 28.6 (21.1 to 38.3) | 34.9 (25.4 to 46.2) | 32.9 (24.2 to 44.1) | 29.4 (21.7 to 39.5) | 36.5 (26.5 to 49.8) | 6 (0.6 to 11.6) | 3.1 (-4.1 to 9.6) | 4.8 (-2.5 to 11.3) |
|  |  | All ages | 1282.2 (964 to 1685.1) | 633.1 (465.7 to 843.5) | 649.1 (482.9 to 855.4) | 4104.8 (3060.8 to 5426.7) | 1867.9 (1400.4 to 2466.6) | 2236.9 (1682.6 to 2949.6) | 220.1 (203.5 to 239.6) | 195 (173.8 to 215.3) | 244.6 (216.7 to 272.3) |
|  | Prevalence | Age-standardized | 307.4 (234.1 to 401.1) | 280.4 (212.8 to 371.1) | 351.4 (266.6 to 456.6) | 337.2 (255.8 to 443.6) | 298.1 (225.2 to 389.4) | 379.9 (287.3 to 501.9) | 9.7 (4.9 to 16.2) | 6.3 (-1.3 to 13.7) | 8.1 (1 to 15.8) |
|  |  | All ages | 11693 (8973.8 to 15102) | 5846.8 (4476.7 to 7734) | 5846.2 (4482.7 to 7570.6) | 38432.1 (29592.5 to 49670.6) | 17355.6 (13127.6 to 22822.7) | 21076.4 (16219.7 to 27197.2) | 228.7 (213.1 to 248.3) | 196.8 (174 to 218.6) | 260.5 (233 to 291.1) |
|  | DALYs (Disability-Adjusted Life Years) | Age-standardized | 65.5 (44.7 to 94.7) | 67.6 (43.3 to 105.5) | 63.7 (37.8 to 88.1) | 75.2 (55.6 to 97.4) | 78.8 (55.6 to 105.5) | 71.2 (49.6 to 96.3) | 14.7 (-11 to 49.4) | 16.7 (-13.4 to 59) | 11.8 (-10.3 to 56.4) |
|  |  | All ages | 2246.1 (1543.7 to 3172.2) | 1315.2 (857.4 to 2077.2) | 930.9 (573.1 to 1313.4) | 7684.9 (5624.9 to 9928.4) | 4164.3 (2900.6 to 5519.6) | 3520.5 (2425.4 to 4748.5) | 242.1 (167.5 to 335.9) | 216.6 (136.2 to 327.4) | 278.2 (203.8 to 405.5) |
|  | Deaths | Age-standardized | 3.1 (1.8 to 4.9) | 3.3 (1.8 to 5.7) | 2.8 (1.2 to 4.3) | 3.7 (2.6 to 5.1) | 4.2 (2.6 to 6) | 3.3 (1.9 to 5) | 21.6 (-17.1 to 84.4) | 26.6 (-13.5 to 97.9) | 17 (-20.9 to 134.1) |
|  |  | All ages | 75.5 (44.4 to 121.5) | 51.1 (27.7 to 90.5) | 24.4 (10.6 to 39.2) | 290.8 (198.2 to 403.6) | 174.3 (107.9 to 253.2) | 116.5 (68.9 to 181) | 285.1 (164.5 to 483.1) | 241.1 (130.2 to 433) | 377.3 (232.1 to 799) |

**Table S4.** Rate of incidence, prevalence, disability-adjusted life years (DALYs), and deaths of atrial fibrillation and flutter in the Eastern Mediterranean Region in 1990 and 2021, by sex and age

| Measure | Year | Age Group | Sex | | |
| --- | --- | --- | --- | --- | --- |
| Incidence | 1990 |  | Both | Women | Men |
|  |  | <5 years | 0 (0 to 0) | 0 (0 to 0) | 0 (0 to 0) |
|  |  | 5-9 years | 0 (0 to 0) | 0 (0 to 0) | 0 (0 to 0) |
|  |  | 10-14 years | 0 (0 to 0) | 0 (0 to 0) | 0 (0 to 0) |
|  |  | 15-19 years | 0 (0 to 0) | 0 (0 to 0) | 0 (0 to 0) |
|  |  | 20-24 years | 0 (0 to 0) | 0 (0 to 0) | 0 (0 to 0) |
|  |  | 25-29 years | 0 (0 to 0) | 0 (0 to 0) | 0 (0 to 0) |
|  |  | 30-34 years | 2.2 (0.9 to 4) | 1.6 (0.6 to 3.1) | 2.6 (1.2 to 4.8) |
|  |  | 35-39 years | 6.5 (2.8 to 11.9) | 4.9 (1.9 to 9.3) | 8 (3.5 to 14.3) |
|  |  | 40-44 years | 12.4 (7.6 to 18.8) | 10.3 (6.3 to 15.9) | 14.4 (8.9 to 21.7) |
|  |  | 45-49 years | 20.2 (10.5 to 35.1) | 18 (9.1 to 32) | 22.3 (11.8 to 38.1) |
|  |  | 50-54 years | 35.8 (22.2 to 56.2) | 33 (20.4 to 51.6) | 38.4 (23.8 to 60.1) |
|  |  | 55-59 years | 58.7 (30 to 100.3) | 55.1 (28.3 to 94) | 61.9 (31.5 to 106.1) |
|  |  | 60-64 years | 109.4 (70 to 162.2) | 101.3 (64.8 to 149.4) | 116.4 (75.3 to 172.5) |
|  |  | 65-69 years | 188.9 (89.3 to 317.7) | 171.9 (81.8 to 285.6) | 203.6 (95.7 to 341.7) |
|  |  | 70-74 years | 287.3 (165.6 to 427.4) | 253.4 (143.2 to 380.1) | 316.1 (182.2 to 472.2) |
|  |  | 75-79 years | 402.9 (194.4 to 696.1) | 346.2 (167.3 to 600.1) | 451 (217.3 to 779.9) |
|  |  | 80-84 years | 501.9 (284.7 to 798.2) | 432.8 (246.9 to 687) | 563.3 (318.6 to 898.4) |
|  |  | 85-89 years | 586.2 (306.2 to 1030.1) | 515 (263.3 to 908.8) | 652.7 (344 to 1144.9) |
|  |  | 90-94 years | 657.3 (328.7 to 1076.2) | 594.2 (303.1 to 978.1) | 716.7 (362.5 to 1172.7) |
|  |  | 95+ years | 722.3 (307.7 to 1359.7) | 674.3 (285.4 to 1271.2) | 776.4 (328.1 to 1443) |
| Incidence | 2021 |  | Both | Women | Men |
|  |  | <5 years | 0 (0 to 0) | 0 (0 to 0) | 0 (0 to 0) |
|  |  | 5-9 years | 0 (0 to 0) | 0 (0 to 0) | 0 (0 to 0) |
|  |  | 10-14 years | 0 (0 to 0) | 0 (0 to 0) | 0 (0 to 0) |
|  |  | 15-19 years | 0 (0 to 0) | 0 (0 to 0) | 0 (0 to 0) |
|  |  | 20-24 years | 0 (0 to 0) | 0 (0 to 0) | 0 (0 to 0) |
|  |  | 25-29 years | 0 (0 to 0) | 0 (0 to 0) | 0 (0 to 0) |
|  |  | 30-34 years | 2.3 (1 to 4.3) | 1.8 (0.7 to 3.4) | 2.9 (1.3 to 5.1) |
|  |  | 35-39 years | 7 (3.1 to 13) | 5.3 (2.1 to 10.2) | 8.6 (3.8 to 15.5) |
|  |  | 40-44 years | 13.5 (8.3 to 20.1) | 11.1 (6.8 to 16.8) | 15.5 (9.8 to 23.2) |
|  |  | 45-49 years | 21.5 (11.2 to 37.1) | 19.3 (9.8 to 34) | 23.5 (12.4 to 39.8) |
|  |  | 50-54 years | 37.9 (23.5 to 59.3) | 35.5 (21.9 to 56) | 40 (24.5 to 62.4) |
|  |  | 55-59 years | 62.5 (32.1 to 107.9) | 59.8 (30.7 to 103) | 65 (33.5 to 110.8) |
|  |  | 60-64 years | 116.7 (75.2 to 170.8) | 110.4 (70.7 to 161.1) | 122.5 (79.6 to 178.6) |
|  |  | 65-69 years | 198.4 (95.9 to 332.7) | 185.5 (89.9 to 309.3) | 210.4 (99.1 to 352.1) |
|  |  | 70-74 years | 297.1 (171.3 to 440.9) | 268.1 (153 to 397.8) | 324.8 (189.9 to 484.4) |
|  |  | 75-79 years | 408.8 (197.7 to 699.3) | 356 (172.4 to 622.5) | 460.1 (223.4 to 770.4) |
|  |  | 80-84 years | 499.7 (282 to 793.6) | 437.6 (247.1 to 687.3) | 559.9 (317.6 to 894.5) |
|  |  | 85-89 years | 577.4 (304.4 to 1011.6) | 519.5 (266.6 to 929.6) | 630.3 (335.6 to 1091.1) |
|  |  | 90-94 years | 645.4 (317.8 to 1061.2) | 600.3 (298.6 to 980) | 683.7 (333.9 to 1130.2) |
|  |  | 95+ years | 706.7 (302.1 to 1329.3) | 682.1 (287.7 to 1278) | 726.5 (309.5 to 1371.1) |
| Prevalence | 1990 |  | Both | Women | Men |
|  |  | <5 years | 0 (0 to 0) | 0 (0 to 0) | 0 (0 to 0) |
|  |  | 5-9 years | 0 (0 to 0) | 0 (0 to 0) | 0 (0 to 0) |
|  |  | 10-14 years | 0 (0 to 0) | 0 (0 to 0) | 0 (0 to 0) |
|  |  | 15-19 years | 0 (0 to 0) | 0 (0 to 0) | 0 (0 to 0) |
|  |  | 20-24 years | 0 (0 to 0) | 0 (0 to 0) | 0 (0 to 0) |
|  |  | 25-29 years | 0 (0 to 0) | 0 (0 to 0) | 0 (0 to 0) |
|  |  | 30-34 years | 3.7 (1.6 to 6.8) | 2.7 (1.1 to 5.2) | 4.5 (2 to 8.1) |
|  |  | 35-39 years | 25.1 (10.8 to 46.5) | 18.9 (7.5 to 36.1) | 31 (13.8 to 55.6) |
|  |  | 40-44 years | 70.9 (36.5 to 120.6) | 55.1 (28.1 to 96) | 85.6 (44.4 to 143.7) |
|  |  | 45-49 years | 151.7 (92.8 to 231.9) | 125.2 (75.9 to 194.9) | 176.9 (109.3 to 268.7) |
|  |  | 50-54 years | 283.8 (183.2 to 418.9) | 244 (153.6 to 369.1) | 320.7 (208.8 to 467.9) |
|  |  | 55-59 years | 514.6 (340.5 to 744.9) | 457.2 (298.9 to 670.6) | 566.2 (383.7 to 807.9) |
|  |  | 60-64 years | 905.2 (621.6 to 1314) | 817.5 (554.6 to 1184.8) | 981.3 (669.3 to 1428.3) |
|  |  | 65-69 years | 1648.2 (1177.1 to 2297.2) | 1485.6 (1061.6 to 2078.1) | 1789.1 (1284.6 to 2492.8) |
|  |  | 70-74 years | 2807 (1909.1 to 3974.3) | 2498.5 (1694 to 3564.3) | 3069.2 (2098.9 to 4309.8) |
|  |  | 75-79 years | 4480.8 (3049 to 6250.4) | 3899.2 (2628.8 to 5463.4) | 4975.1 (3393.7 to 6924.7) |
|  |  | 80-84 years | 6437.5 (4292.3 to 9266.2) | 5483.1 (3639.8 to 7932.1) | 7285.9 (4864 to 10492.2) |
|  |  | 85-89 years | 8236.1 (5625.7 to 11605) | 6902 (4729.8 to 9753.3) | 9482.3 (6500.6 to 13289.6) |
|  |  | 90-94 years | 9503.9 (6491.3 to 13722.4) | 7770.7 (5280.6 to 11300.4) | 11135.5 (7609.2 to 15988.2) |
|  |  | 95+ years | 9094 (6207.1 to 12847.5) | 7156.4 (4773.3 to 10053.5) | 11277.9 (7789.7 to 15800.2) |
| Prevalence | 2021 |  | Both | Women | Men |
|  |  | <5 years | 0 (0 to 0) | 0 (0 to 0) | 0 (0 to 0) |
|  |  | 5-9 years | 0 (0 to 0) | 0 (0 to 0) | 0 (0 to 0) |
|  |  | 10-14 years | 0 (0 to 0) | 0 (0 to 0) | 0 (0 to 0) |
|  |  | 15-19 years | 0 (0 to 0) | 0 (0 to 0) | 0 (0 to 0) |
|  |  | 20-24 years | 0 (0 to 0) | 0 (0 to 0) | 0 (0 to 0) |
|  |  | 25-29 years | 0 (0 to 0) | 0 (0 to 0) | 0 (0 to 0) |
|  |  | 30-34 years | 4 (1.7 to 7.4) | 3 (1.2 to 5.8) | 4.8 (2.2 to 8.7) |
|  |  | 35-39 years | 27.4 (11.9 to 50.7) | 20.5 (8.3 to 39.7) | 33.6 (15 to 60.4) |
|  |  | 40-44 years | 77.2 (40.4 to 131.6) | 59.5 (30.1 to 104.2) | 93.1 (48.5 to 154.8) |
|  |  | 45-49 years | 164 (100.7 to 247.7) | 134.6 (81.6 to 204.5) | 190.1 (118.8 to 287.3) |
|  |  | 50-54 years | 303.8 (195.8 to 450.2) | 262 (163.8 to 393.9) | 341.2 (223.1 to 502.9) |
|  |  | 55-59 years | 551.6 (369.7 to 800.4) | 495.4 (326.5 to 737.1) | 602.4 (405.7 to 853.9) |
|  |  | 60-64 years | 969.5 (668.2 to 1404.3) | 894 (612.3 to 1296.2) | 1038.8 (712.2 to 1479.8) |
|  |  | 65-69 years | 1735.1 (1240 to 2420.1) | 1613.1 (1146 to 2250.9) | 1849.6 (1322.2 to 2572) |
|  |  | 70-74 years | 2924 (2006.6 to 4123.8) | 2694.7 (1839.6 to 3790.9) | 3143.8 (2144.9 to 4449.3) |
|  |  | 75-79 years | 4590.5 (3149 to 6391.2) | 4135.3 (2828.4 to 5751.3) | 5033.1 (3444.5 to 7027.1) |
|  |  | 80-84 years | 6580.6 (4392.2 to 9440.4) | 5799.4 (3883.1 to 8351.4) | 7337.6 (4883.1 to 10512.4) |
|  |  | 85-89 years | 8584.4 (5855 to 12056.9) | 7429.3 (5060.5 to 10455.7) | 9639.7 (6579.9 to 13513.9) |
|  |  | 90-94 years | 10143.2 (6959.5 to 14565) | 8544.6 (5832.6 to 12357.3) | 11497.7 (7881.8 to 16300.3) |
|  |  | 95+ years | 10204.5 (6995.3 to 14392.4) | 8153.3 (5476.6 to 11524.1) | 11855.7 (8203 to 16666.1) |
| DALYs (Disability-Adjusted Life Years) | 1990 |  | Both | Women | Men |
|  |  | <5 years | 0 (0 to 0) | 0 (0 to 0) | 0 (0 to 0) |
|  |  | 5-9 years | 0 (0 to 0) | 0 (0 to 0) | 0 (0 to 0) |
|  |  | 10-14 years | 0 (0 to 0) | 0 (0 to 0) | 0 (0 to 0) |
|  |  | 15-19 years | 0 (0 to 0) | 0 (0 to 0) | 0 (0 to 0) |
|  |  | 20-24 years | 0 (0 to 0) | 0 (0 to 0) | 0 (0 to 0) |
|  |  | 25-29 years | 0 (0 to 0) | 0 (0 to 0) | 0 (0 to 0) |
|  |  | 30-34 years | 1.3 (0.9 to 1.8) | 1.4 (1 to 2.1) | 1.3 (0.8 to 1.8) |
|  |  | 35-39 years | 3.7 (2.2 to 5.9) | 3.1 (1.9 to 4.9) | 4.2 (2.4 to 6.9) |
|  |  | 40-44 years | 10.3 (6.6 to 15.3) | 9.1 (6 to 13.8) | 11.3 (6.7 to 18.2) |
|  |  | 45-49 years | 21.9 (15 to 31.1) | 20.2 (13.6 to 29.3) | 23.4 (15.3 to 33.6) |
|  |  | 50-54 years | 42.5 (29.9 to 57.8) | 40.4 (28.2 to 56.7) | 44.3 (29.4 to 62.4) |
|  |  | 55-59 years | 77.1 (55.9 to 104.3) | 77.2 (56.4 to 107.6) | 76.9 (53.1 to 107.2) |
|  |  | 60-64 years | 132.2 (95.8 to 180) | 135.5 (99.2 to 187.1) | 129.5 (89.5 to 178.9) |
|  |  | 65-69 years | 227.6 (168.5 to 302.2) | 231.9 (171.2 to 306.8) | 223.9 (156.3 to 310.6) |
|  |  | 70-74 years | 414.5 (302.3 to 558.1) | 432.6 (320.1 to 585.8) | 399.1 (268.1 to 551.4) |
|  |  | 75-79 years | 682.7 (500.6 to 905.6) | 719.8 (525.8 to 997) | 651.2 (457 to 888.7) |
|  |  | 80-84 years | 1189.2 (892.3 to 1577.1) | 1234.7 (908.5 to 1728.2) | 1148.7 (798.6 to 1485) |
|  |  | 85-89 years | 1800.2 (1355.5 to 2323.3) | 1919.5 (1411.9 to 2573.9) | 1688.7 (1183.5 to 2167) |
|  |  | 90-94 years | 2980.5 (2253.7 to 3752.4) | 3196.9 (2322.9 to 4260.8) | 2776.7 (1930.1 to 3495.4) |
|  |  | 95+ years | 3635.4 (2639.1 to 4676) | 3684.3 (2554.3 to 4975.3) | 3580.2 (2396.5 to 4892.6) |
| DALYs (Disability-Adjusted Life Years) | 2021 |  | Both | Women | Men |
|  |  | <5 years | 0 (0 to 0) | 0 (0 to 0) | 0 (0 to 0) |
|  |  | 5-9 years | 0 (0 to 0) | 0 (0 to 0) | 0 (0 to 0) |
|  |  | 10-14 years | 0 (0 to 0) | 0 (0 to 0) | 0 (0 to 0) |
|  |  | 15-19 years | 0 (0 to 0) | 0 (0 to 0) | 0 (0 to 0) |
|  |  | 20-24 years | 0 (0 to 0) | 0 (0 to 0) | 0 (0 to 0) |
|  |  | 25-29 years | 0 (0 to 0) | 0 (0 to 0) | 0 (0 to 0) |
|  |  | 30-34 years | 1.4 (1.1 to 1.8) | 1.5 (1.1 to 1.9) | 1.4 (1 to 1.9) |
|  |  | 35-39 years | 3.8 (2.4 to 6) | 3.2 (1.9 to 5) | 4.5 (2.7 to 7.2) |
|  |  | 40-44 years | 11 (7.4 to 16.9) | 9.5 (6.4 to 13.8) | 12.2 (8 to 19.4) |
|  |  | 45-49 years | 22.9 (15.9 to 31.9) | 21 (14.7 to 28.1) | 24.6 (16.8 to 35.4) |
|  |  | 50-54 years | 43.9 (31.8 to 59.2) | 41.5 (30 to 55.9) | 46 (32.2 to 64) |
|  |  | 55-59 years | 81.4 (60.1 to 107.2) | 81.1 (60.3 to 105.6) | 81.7 (58.5 to 111.7) |
|  |  | 60-64 years | 139.7 (107.7 to 185.3) | 143.2 (107.2 to 186.8) | 136.6 (99.8 to 185.9) |
|  |  | 65-69 years | 241.2 (187.1 to 312.9) | 249.4 (189.9 to 317.5) | 233.4 (173.8 to 309.3) |
|  |  | 70-74 years | 451.4 (350.2 to 576.9) | 480.3 (379.1 to 598.6) | 423.7 (315.2 to 562.6) |
|  |  | 75-79 years | 747.1 (582.2 to 940) | 800.5 (626 to 989.1) | 695.3 (519.9 to 901.6) |
|  |  | 80-84 years | 1371.7 (1136.9 to 1683.8) | 1502.8 (1216.6 to 1785.3) | 1244.7 (988.5 to 1572.1) |
|  |  | 85-89 years | 2113.8 (1739.8 to 2508.8) | 2352.5 (1881.6 to 2770.6) | 1895.8 (1564.6 to 2347.3) |
|  |  | 90-94 years | 3764.5 (3049.8 to 4477.3) | 4325 (3382.3 to 5078) | 3289.6 (2575.7 to 4078.2) |
|  |  | 95+ years | 4801.2 (3703.7 to 6074.2) | 5704.1 (4252.1 to 7503.9) | 4074.3 (3027.7 to 5653.8) |
| Deaths | 1990 |  | Both | Women | Men |
|  |  | 30-34 years | 0 (0 to 0) | 0 (0 to 0) | 0 (0 to 0) |
|  |  | 35-39 years | 0 (0 to 0) | 0 (0 to 0) | 0 (0 to 0) |
|  |  | 40-44 years | 0.1 (0.1 to 0.1) | 0.1 (0.1 to 0.1) | 0.1 (0.1 to 0.1) |
|  |  | 45-49 years | 0.2 (0.2 to 0.3) | 0.2 (0.2 to 0.4) | 0.2 (0.1 to 0.3) |
|  |  | 50-54 years | 0.5 (0.4 to 0.7) | 0.5 (0.4 to 0.8) | 0.5 (0.3 to 0.6) |
|  |  | 55-59 years | 1.1 (0.8 to 1.4) | 1.2 (0.9 to 1.8) | 0.9 (0.6 to 1.2) |
|  |  | 60-64 years | 2.1 (1.5 to 2.8) | 2.5 (1.8 to 3.6) | 1.8 (1.1 to 2.3) |
|  |  | 65-69 years | 4.1 (3 to 5.5) | 4.8 (3.5 to 7.1) | 3.4 (2.2 to 4.5) |
|  |  | 70-74 years | 10 (7.3 to 13.5) | 12.1 (9 to 17.9) | 8.1 (5 to 10.7) |
|  |  | 75-79 years | 21.7 (15.6 to 29) | 26.8 (19.7 to 39.4) | 17.3 (10.4 to 22.7) |
|  |  | 80-84 years | 57.4 (41 to 77) | 66.8 (48.1 to 100) | 49.1 (29.6 to 66.4) |
|  |  | 85-89 years | 122.3 (86.6 to 158.9) | 144.4 (99.3 to 203) | 101.7 (61.2 to 133.5) |
|  |  | 90-94 years | 269.4 (188.5 to 354) | 308.9 (209.8 to 436.5) | 232.3 (146.3 to 303.7) |
|  |  | 95+ years | 373.1 (255.5 to 494.2) | 396.6 (255.2 to 563.7) | 346.6 (212.9 to 505.1) |
| Deaths | 2021 |  | Both | Women | Men |
|  |  | 30-34 years | 0 (0 to 0) | 0 (0 to 0) | 0 (0 to 0) |
|  |  | 35-39 years | 0 (0 to 0) | 0 (0 to 0) | 0 (0 to 0) |
|  |  | 40-44 years | 0.1 (0.1 to 0.1) | 0.1 (0.1 to 0.1) | 0.1 (0.1 to 0.1) |
|  |  | 45-49 years | 0.2 (0.2 to 0.3) | 0.2 (0.2 to 0.3) | 0.2 (0.2 to 0.3) |
|  |  | 50-54 years | 0.5 (0.4 to 0.6) | 0.5 (0.4 to 0.7) | 0.5 (0.4 to 0.6) |
|  |  | 55-59 years | 1.1 (0.9 to 1.4) | 1.3 (0.9 to 1.6) | 1 (0.8 to 1.2) |
|  |  | 60-64 years | 2.2 (1.8 to 2.6) | 2.5 (1.9 to 3.2) | 1.9 (1.5 to 2.3) |
|  |  | 65-69 years | 4.4 (3.6 to 5.2) | 5.2 (4.1 to 6.4) | 3.7 (2.9 to 4.4) |
|  |  | 70-74 years | 11.5 (9.6 to 13.3) | 13.9 (11.1 to 16.9) | 9.2 (7.5 to 11) |
|  |  | 75-79 years | 25.4 (20.3 to 29.6) | 31 (24.6 to 37.4) | 19.9 (15.8 to 24.5) |
|  |  | 80-84 years | 71.5 (59.2 to 81.9) | 86.8 (70.7 to 102.4) | 56.7 (46.3 to 67.3) |
|  |  | 85-89 years | 151.6 (120.2 to 174.3) | 184.3 (142.8 to 212.5) | 121.8 (95.7 to 143.8) |
|  |  | 90-94 years | 356 (279.3 to 416.9) | 434.5 (330.8 to 509.8) | 289.4 (211.7 to 370) |
|  |  | 95+ years | 501.1 (368.3 to 646.7) | 629 (454.4 to 847.8) | 398.2 (271.9 to 568.6) |

**Table S5.** Age-standardized rate of incidence, prevalence, disability-adjusted life years (DALYs), and deaths of atrial fibrillation and flutter in the countries of Eastern Mediterranean Region in 2021, by sociodemographic index (SDI) quintiles in both sexes.

| Country | SDI | Abbreviation | Incidence 2021 | Prevalence 2021 | DALYs 2021 | Deaths 2021 |
| --- | --- | --- | --- | --- | --- | --- |
| United Arab Emirates | High SDI | UA | 39.7 (29.2 to 52.8) | 424.7 (326.5 to 554.8) | 85.8 (66.1 to 105.2) | 3.8 (2.5 to 4.9) |
| Kuwait | High SDI | KU | 37.9 (27.9 to 50.9) | 408.1 (310.6 to 530.2) | 66.6 (52.8 to 82.2) | 3 (2.4 to 3.6) |
| Qatar | High SDI | QA | 37.6 (27.6 to 50.6) | 402.6 (305.8 to 529.3) | 78.5 (45.5 to 103.7) | 4.1 (1.5 to 6) |
| Saudi Arabia | High SDI | SA | 36.9 (27.2 to 49.8) | 391.9 (301.9 to 511.7) | 72.2 (58.2 to 88.6) | 3.4 (2.7 to 4.1) |
| Oman | High-middle SDI | OM | 34.4 (25.3 to 46.3) | 365.3 (276.4 to 482.9) | 91 (74.6 to 109.2) | 4.7 (3.6 to 5.9) |
| Bahrain | High-middle SDI | BA | 36.3 (26.6 to 48.2) | 386.7 (293.8 to 505.6) | 94.9 (57.6 to 122.8) | 5.7 (2.6 to 7.9) |
| Lebanon | High-middle SDI | LE | 36.2 (27 to 48.3) | 387.1 (298.5 to 511.1) | 68.9 (56.3 to 84.2) | 3.4 (2.8 to 4.4) |
| Libya | High-middle SDI | LI | 37.7 (27.8 to 50.7) | 398.3 (304.8 to 518.5) | 70.1 (50.4 to 92.3) | 3.1 (1.9 to 4.5) |
| Jordan | High-middle SDI | JO | 37 (27.5 to 49.7) | 396.6 (300.6 to 520.3) | 63.1 (49.4 to 78.3) | 2.7 (2.1 to 3.3) |
| Iran (Islamic Republic of) | Middle SDI | IN | 40.6 (30 to 54.4) | 425.4 (327.2 to 559.2) | 72.4 (57.4 to 88.3) | 3.3 (2.5 to 3.8) |
| Tunisia | Middle SDI | TU | 34.5 (25 to 45.6) | 365 (276.8 to 471.6) | 81.8 (60.5 to 107.5) | 4.6 (3 to 6.7) |
| Iraq | Middle SDI | IQ | 38.4 (28.1 to 51.2) | 406.1 (308.5 to 532) | 93.6 (74.2 to 114) | 4.9 (3.5 to 6.2) |
| Palestine | Middle SDI | PA | 33.2 (24.4 to 44.7) | 347.4 (265.2 to 454) | 89.8 (74.3 to 107.6) | 5.4 (4.3 to 6.7) |
| Syrian Arab Republic | Middle SDI | SY | 35.3 (25.7 to 47.1) | 377.8 (288.1 to 497.3) | 85.7 (63.8 to 105.2) | 4.6 (2.9 to 5.9) |
| Egypt | Low-middle SDI | EG | 36.9 (27.1 to 49.4) | 393 (297.7 to 514.2) | 75.9 (62.4 to 91.2) | 3.6 (2.9 to 4.3) |
| Morocco | Low-middle SDI | MO | 36.6 (26.9 to 49.6) | 383.3 (292.5 to 506) | 83.6 (65.6 to 104.3) | 4.3 (3 to 5.5) |
| Sudan | Low-middle SDI | SU | 37.8 (27.5 to 51) | 394.5 (298.4 to 515.7) | 72.5 (56.1 to 90.4) | 3.2 (2.3 to 4.2) |
| Pakistan | Low-middle SDI | PK | 53.8 (40.3 to 71.6) | 558.6 (429.5 to 735.7) | 101.5 (79.7 to 131.7) | 4.6 (3.3 to 6.4) |
| Djibouti | Low-middle SDI | DJ | 37.4 (28.6 to 49.6) | 424.6 (335.8 to 555.3) | 83.7 (61.7 to 110.9) | 3.8 (2.5 to 5.5) |
| Yemen | Low SDI | YE | 32.9 (24.2 to 44.1) | 337.2 (255.8 to 443.6) | 75.2 (55.6 to 97.4) | 3.7 (2.6 to 5.1) |
| Afghanistan | Low SDI | AF | 33.3 (24.5 to 44.5) | 338.8 (257.5 to 442.8) | 72.1 (51.5 to 95.1) | 3.3 (2 to 4.9) |
| Somalia | Low SDI | SO | 35.6 (27.4 to 47.3) | 392 (308.4 to 516.9) | 63.6 (39.5 to 97.4) | 2.2 (1 to 4) |

**Table S6.** All ages number and age‑standardized rate of disability-adjusted life years (DALYs) and deaths of atrial fibrillation and flutter attributable to risk factors by sex in 1990 and 2021 and overall percent change over 1990–2021 in the Eastern Mediterranean Region countries

| Location | Risk factor | Measure | Age, Metric | Year | | | | | | % Change (1990 to 2021) | | |
| --- | --- | --- | --- | --- | --- | --- | --- | --- | --- | --- | --- | --- |
|  |  |  |  | 1990 | | | 2021 | | |  |  |  |
|  |  |  |  | Both | Women | Men | Both | Women | Men | Both | Women | Men |
| Eastern Mediterranean Region | All risk factors | DALYs (Disability-Adjusted Life Years) | Age-standardized | 25.7 (11.9 to 40.5) | 27.5 (12.1 to 45.1) | 24.2 (11.8 to 38) | 32.5 (17.1 to 48.5) | 34.9 (18.3 to 51.5) | 30.2 (16 to 44.9) | 26.2 (7.4 to 55.7) | 27.2 (-0.8 to 58.8) | 24.9 (7.8 to 56.2) |
|  |  |  | All ages | 35775.3 (17404.7 to 56049.2) | 17506.3 (7910.5 to 28332.5) | 18269 (9071.5 to 28313.9) | 111844.2 (59019.1 to 165701.7) | 56239.6 (29097.8 to 83137.3) | 55604.6 (29808.8 to 82448.1) | 212.6 (168.3 to 281.2) | 221.3 (153.9 to 301.5) | 204.4 (164.2 to 277.7) |
|  |  | Deaths | Age-standardized | 1.1 (0.5 to 1.7) | 1.3 (0.6 to 2.3) | 0.9 (0.3 to 1.4) | 1.5 (0.8 to 2.2) | 1.8 (0.9 to 2.6) | 1.2 (0.6 to 1.8) | 36.1 (4.1 to 85.4) | 37.3 (-3.5 to 85.1) | 34.3 (4.5 to 107.4) |
|  |  |  | All ages | 1162.3 (503.4 to 1880.8) | 667.8 (290.4 to 1168.4) | 494.5 (196 to 801.6) | 3930.1 (2064 to 5790.7) | 2289.1 (1143.4 to 3411.5) | 1641 (888.3 to 2463.1) | 238.1 (158.3 to 363) | 242.8 (140.8 to 361.2) | 231.9 (157.2 to 414) |
|  | Alcohol use | DALYs (Disability-Adjusted Life Years) | Age-standardized | 0.1 (0.1 to 0.2) | 0 (0 to 0) | 0.2 (0.1 to 0.3) | 0.2 (0.1 to 0.3) | 0 (0 to 0) | 0.3 (0.2 to 0.5) | 28.6 (-3.7 to 70.9) | 28.4 (-10.8 to 82.3) | 30.6 (-2.8 to 77.5) |
|  |  |  | All ages | 220 (124.4 to 324.8) | 18.1 (10.3 to 26.7) | 201.9 (112.3 to 303.1) | 717.6 (444.6 to 1050.2) | 60.2 (36.5 to 90.5) | 657.4 (405.6 to 969) | 226.2 (148 to 328.7) | 232 (139.7 to 361.7) | 225.6 (147.1 to 334.5) |
|  |  | Deaths | Age-standardized | 0 (0 to 0) | 0 (0 to 0) | 0 (0 to 0) | 0 (0 to 0) | 0 (0 to 0) | 0 (0 to 0) | 33.1 (-12.6 to 101) | 29.2 (-20.6 to 105.5) | 33.5 (-15.6 to 108) |
|  |  |  | All ages | 5.1 (2.7 to 7.8) | 0.6 (0.3 to 0.9) | 4.5 (2.3 to 7.2) | 16.2 (9.7 to 23.7) | 1.9 (1.1 to 2.9) | 14.4 (8.5 to 21.5) | 220.4 (114.3 to 384.1) | 222 (103.7 to 396.2) | 220.2 (106.7 to 400.1) |
|  | Diet high in sodium | DALYs (Disability-Adjusted Life Years) | Age-standardized | 0.8 (0 to 3.6) | 0.6 (0 to 2.9) | 0.9 (0 to 4.1) | 1 (0 to 4.3) | 0.9 (0 to 3.9) | 1.1 (0 to 4.9) | 30 (6.4 to 456.5) | 43.6 (5.8 to 527.2) | 22.8 (-6.7 to 526.9) |
|  |  |  | All ages | 1085.4 (4.8 to 5058.1) | 391.7 (1.5 to 1911.3) | 693.7 (3 to 3088.7) | 3486.3 (35.8 to 14769.1) | 1408.8 (10.4 to 6354.2) | 2077.5 (20.1 to 8896.1) | 221.2 (165.3 to 1241.2) | 259.7 (166 to 1411.8) | 199.5 (128.7 to 1453.3) |
|  |  | Deaths | Age-standardized | 0 (0 to 0.1) | 0 (0 to 0.1) | 0 (0 to 0.1) | 0 (0 to 0.2) | 0 (0 to 0.2) | 0 (0 to 0.2) | 48.4 (7.1 to 579.1) | 63.1 (9.2 to 821.8) | 37.4 (-11 to 730.7) |
|  |  |  | All ages | 31.8 (0.1 to 136) | 13.2 (0 to 69.1) | 18.6 (0.1 to 81.6) | 116.1 (1.1 to 513.2) | 53.2 (0.3 to 255.3) | 62.9 (0.5 to 261.5) | 265 (171.8 to 1557.4) | 301.2 (167.6 to 1910.2) | 239.1 (125.3 to 1712) |
|  | High body-mass index | DALYs (Disability-Adjusted Life Years) | Age-standardized | 3.4 (1.3 to 5.9) | 5 (2 to 8.8) | 2 (0.7 to 3.5) | 9.7 (4.2 to 16.2) | 11.9 (5.3 to 20) | 7.6 (3 to 12.7) | 182.6 (130.8 to 242.3) | 136.2 (82.6 to 194.5) | 284.4 (204.7 to 400.6) |
|  |  |  | All ages | 5000.7 (1925.8 to 8606.6) | 3454.4 (1364.1 to 6011.3) | 1546.4 (589.8 to 2755.9) | 34355.5 (15119.6 to 57930.1) | 20151.1 (8987.3 to 33817.7) | 14204.4 (5855.7 to 24318.8) | 587 (468.9 to 715.4) | 483.4 (360.7 to 615.9) | 818.6 (642.5 to 1066.7) |
|  |  | Deaths | Age-standardized | 0.2 (0.1 to 0.3) | 0.2 (0.1 to 0.4) | 0.1 (0 to 0.1) | 0.4 (0.2 to 0.7) | 0.6 (0.3 to 1) | 0.3 (0.1 to 0.5) | 184.4 (107.8 to 281.8) | 148.3 (72.1 to 247.4) | 288.7 (172.6 to 502) |
|  |  |  | All ages | 167.9 (66.6 to 295.1) | 125.3 (51.5 to 220.1) | 42.6 (14.9 to 78.7) | 1164 (522.3 to 1911.9) | 761.3 (339 to 1263.1) | 402.7 (176 to 665.3) | 593.4 (410.5 to 815.9) | 507.8 (330 to 733.7) | 845.2 (574.9 to 1327.9) |
|  | High systolic blood pressure | DALYs (Disability-Adjusted Life Years) | Age-standardized | 21.4 (7.4 to 35.8) | 24.4 (8.9 to 41.6) | 18.7 (6 to 32.8) | 25.5 (8.8 to 42) | 28.4 (10.1 to 46.2) | 22.8 (7.3 to 38.3) | 19.4 (1.2 to 39.8) | 16.7 (-7.1 to 39.9) | 22.1 (5.4 to 50.4) |
|  |  |  | All ages | 28975.8 (9929.6 to 49161.8) | 15317.5 (5571.1 to 26097) | 13658.2 (4382.1 to 24035.7) | 85903.3 (29483.1 to 141398.8) | 45133 (16049.2 to 72676.6) | 40770.3 (13227.5 to 68497.8) | 196.5 (154.3 to 242.4) | 194.6 (138.4 to 250) | 198.5 (157.2 to 263.1) |
|  |  | Deaths | Age-standardized | 0.9 (0.3 to 1.6) | 1.2 (0.4 to 2.1) | 0.7 (0.2 to 1.3) | 1.2 (0.4 to 1.9) | 1.5 (0.5 to 2.4) | 0.9 (0.3 to 1.5) | 27.6 (-2 to 67.1) | 26.5 (-9.3 to 65.2) | 29.5 (1.2 to 96) |
|  |  |  | All ages | 983.7 (325 to 1722.5) | 593.3 (217.1 to 1094.7) | 390.4 (108.7 to 702.1) | 3137.9 (1133.8 to 5082.2) | 1877.5 (681.6 to 3037.2) | 1260.4 (425.9 to 2127.1) | 219 (143.1 to 316.3) | 216.4 (127.2 to 313.5) | 222.8 (151.5 to 392) |
|  | Lead exposure | DALYs (Disability-Adjusted Life Years) | Age-standardized | 3.3 (-0.5 to 8.2) | 2.9 (-0.4 to 7.7) | 3.6 (-0.5 to 9.1) | 3.8 (-0.5 to 9.6) | 3.7 (-0.5 to 9.1) | 4 (-0.5 to 10.1) | 17.1 (1.8 to 38.8) | 25.5 (-0.2 to 50.7) | 11.3 (-2.6 to 35.8) |
|  |  |  | All ages | 4522.4 (-627.1 to 11350.6) | 1874.5 (-275.2 to 4899.1) | 2647.9 (-373.2 to 6636.7) | 12338.2 (-1641.6 to 30821.6) | 5555.7 (-735.5 to 13766.1) | 6782.5 (-906.1 to 17008.9) | 172.8 (139.8 to 219.7) | 196.4 (138.6 to 251.7) | 156.1 (125.3 to 207.9) |
|  |  | Deaths | Age-standardized | 0.1 (0 to 0.4) | 0.1 (0 to 0.4) | 0.1 (0 to 0.4) | 0.2 (0 to 0.5) | 0.2 (0 to 0.5) | 0.2 (0 to 0.4) | 36.7 (7.8 to 82.9) | 45.9 (5.6 to 88.2) | 27.9 (0.1 to 95.3) |
|  |  |  | All ages | 145.6 (-22.5 to 382.4) | 70.8 (-12.1 to 188.5) | 74.8 (-11.3 to 208.3) | 477.6 (-70.7 to 1170.5) | 247.5 (-35.7 to 590.3) | 230.1 (-35 to 581.9) | 227.9 (157.4 to 340.4) | 249.3 (153.3 to 351.9) | 207.6 (140.7 to 369.3) |
|  | Smoking | DALYs (Disability-Adjusted Life Years) | Age-standardized | 3.6 (2 to 5.5) | 0.8 (0.4 to 1.3) | 6 (3.3 to 9.3) | 3.1 (1.7 to 4.6) | 0.8 (0.4 to 1.2) | 5.2 (2.9 to 7.7) | -14.2 (-25.8 to 2.1) | -7.8 (-30.2 to 17.4) | -13.6 (-26.2 to 4.2) |
|  |  |  | All ages | 5710 (3200 to 8733.9) | 650 (349.1 to 1038.3) | 5060 (2821.3 to 7865.4) | 12536 (7088.7 to 18612.9) | 1465 (814.7 to 2316.9) | 11071 (6321.6 to 16510.9) | 119.5 (91.3 to 157.9) | 125.4 (74.1 to 185) | 118.8 (89.3 to 160.4) |
|  |  | Deaths | Age-standardized | 0.1 (0.1 to 0.2) | 0 (0 to 0) | 0.2 (0.1 to 0.3) | 0.1 (0.1 to 0.1) | 0 (0 to 0) | 0.2 (0.1 to 0.2) | -5.7 (-28.4 to 37.6) | 10.1 (-25.6 to 57.8) | -7.9 (-31.3 to 39.4) |
|  |  |  | All ages | 126.6 (65.2 to 194) | 17.4 (9 to 28.1) | 109.3 (53.1 to 172) | 295.6 (173.1 to 430.7) | 44.9 (23.9 to 68.5) | 250.7 (148.8 to 365.5) | 133.4 (80.5 to 239) | 158.5 (72.9 to 263.6) | 129.5 (74.8 to 248.2) |
| Afghanistan | All risk factors | DALYs (Disability-Adjusted Life Years) | Age-standardized | 19 (6.9 to 34.3) | 20.9 (8 to 40.7) | 17.5 (5.2 to 33.7) | 23.9 (10.5 to 39.3) | 24.9 (10.8 to 43.1) | 22.6 (9.2 to 38.2) | 25.4 (-2.1 to 65.3) | 19.5 (-14.7 to 66.2) | 29.1 (-4.7 to 86.8) |
|  |  |  | All ages | 1050.6 (396.3 to 1874.2) | 537.4 (207.4 to 1019.8) | 513.2 (160.8 to 962.3) | 1706.7 (776.2 to 2817.4) | 934 (421 to 1593.2) | 772.7 (334 to 1292.7) | 62.4 (27.8 to 115.5) | 73.8 (27 to 144.1) | 50.6 (10.5 to 116.4) |
|  |  | Deaths | Age-standardized | 0.8 (0.2 to 1.7) | 0.9 (0.3 to 2) | 0.7 (0.1 to 1.6) | 1.1 (0.4 to 1.8) | 1.2 (0.4 to 2.4) | 0.9 (0.3 to 1.7) | 30.1 (-4.6 to 90.4) | 26.9 (-18.3 to 87) | 29.7 (-14.7 to 118.7) |
|  |  |  | All ages | 34.5 (10 to 70.7) | 18.6 (6.2 to 40.5) | 15.9 (3.5 to 35) | 59.1 (22.7 to 102.3) | 34.3 (13.4 to 65.1) | 24.9 (7.9 to 45.5) | 71.4 (24.9 to 150) | 84.4 (22.1 to 190.3) | 56.3 (3 to 170.8) |
|  | Alcohol use | DALYs (Disability-Adjusted Life Years) | Age-standardized | 0 (0 to 0) | 0 (0 to 0) | 0 (0 to 0) | 0 (0 to 0) | 0 (0 to 0) | 0 (0 to 0.1) | NA | NA | NA |
|  |  |  | All ages | 0 (0 to 0) | 0 (0 to 0) | 0 (0 to 0) | 1.3 (0.4 to 2.8) | 0.1 (0 to 0.3) | 1.2 (0.3 to 2.7) | NA | NA | NA |
|  |  | Deaths | Age-standardized | 0 (0 to 0) | 0 (0 to 0) | 0 (0 to 0) | 0 (0 to 0) | 0 (0 to 0) | 0 (0 to 0) | NA | NA | NA |
|  |  |  | All ages | 0 (0 to 0) | 0 (0 to 0) | 0 (0 to 0) | 0 (0 to 0.1) | 0 (0 to 0) | 0 (0 to 0.1) | NA | NA | NA |
|  | Diet high in sodium | DALYs (Disability-Adjusted Life Years) | Age-standardized | 0.4 (0 to 2.1) | 0.2 (0 to 1.6) | 0.5 (0 to 2.6) | 0.4 (0 to 2.3) | 0.2 (0 to 1.8) | 0.5 (0 to 3) | 4.8 (-94.5 to 770.5) | 10.9 (-228.9 to 13441.7) | 10.1 (-93.9 to 933.1) |
|  |  |  | All ages | 21.9 (0 to 124.4) | 6.2 (0 to 43.6) | 15.6 (0 to 82.4) | 28.6 (0 to 170.9) | 9.8 (0 to 71) | 18.8 (0 to 100.7) | 30.8 (-86.6 to 1270.3) | 57.7 (-251.7 to 88992.1) | 20.1 (-86.7 to 1324.9) |
|  |  | Deaths | Age-standardized | 0 (0 to 0.1) | 0 (0 to 0.1) | 0 (0 to 0.1) | 0 (0 to 0.1) | 0 (0 to 0.1) | 0 (0 to 0.1) | 11.8 (-97.3 to 1325.5) | 17.3 (-221.4 to 19992.5) | 13.9 (-96.8 to 1778.2) |
|  |  |  | All ages | 0.6 (0 to 3.7) | 0.2 (0 to 1.5) | 0.4 (0 to 2.4) | 0.9 (0 to 5.7) | 0.3 (0 to 2.6) | 0.6 (0 to 3.2) | 39.2 (-94.7 to 2018.9) | 66.4 (-573.4 to 68246.4) | 26.7 (-94.7 to 2264.3) |
|  | High body-mass index | DALYs (Disability-Adjusted Life Years) | Age-standardized | 2.2 (0.7 to 4.3) | 3.3 (1.2 to 6.9) | 1.2 (0.3 to 2.7) | 5.1 (2 to 9.3) | 5.2 (2 to 9.5) | 4.9 (1.7 to 9.1) | 135 (59.9 to 254.4) | 56 (4.4 to 151.7) | 311.6 (112.6 to 878) |
|  |  |  | All ages | 150.8 (51.7 to 299) | 109.3 (39.6 to 228.8) | 41.5 (11.2 to 93.3) | 413.4 (160.7 to 738.6) | 236.9 (92.2 to 422.7) | 176.5 (64.9 to 333.4) | 174.1 (96 to 306.1) | 116.7 (49 to 232) | 325.2 (140.5 to 887.7) |
|  |  | Deaths | Age-standardized | 0.1 (0 to 0.2) | 0.1 (0 to 0.3) | 0 (0 to 0.1) | 0.2 (0.1 to 0.4) | 0.2 (0.1 to 0.4) | 0.2 (0.1 to 0.4) | 184.4 (57.2 to 442.8) | 78.4 (-1.2 to 264.2) | 445 (112.9 to 1694.1) |
|  |  |  | All ages | 3.9 (1.2 to 8.7) | 2.9 (1 to 7) | 1 (0.2 to 2.5) | 11.9 (4.3 to 22.9) | 6.8 (2.5 to 14.4) | 5.1 (1.5 to 10.6) | 205.4 (89 to 405.6) | 133.6 (40.3 to 305.9) | 420.9 (143.6 to 1340.6) |
|  | High systolic blood pressure | DALYs (Disability-Adjusted Life Years) | Age-standardized | 17.3 (5 to 32.3) | 19 (6 to 38.6) | 15.9 (4 to 31.7) | 20.3 (6.9 to 35.9) | 22 (7.8 to 40.5) | 18.3 (5.5 to 33.9) | 17.3 (-8.1 to 51) | 15.9 (-20.2 to 64.2) | 15.4 (-17.7 to 61.8) |
|  |  |  | All ages | 932.5 (277.7 to 1736) | 476.4 (145 to 958.1) | 456.1 (118.2 to 885.5) | 1396.2 (474.8 to 2475.3) | 797.3 (271.5 to 1456.5) | 598.9 (179.5 to 1127.8) | 49.7 (15.8 to 93.9) | 67.4 (16.4 to 145.5) | 31.3 (-6.4 to 80.7) |
|  |  | Deaths | Age-standardized | 0.8 (0.2 to 1.6) | 0.9 (0.3 to 1.9) | 0.7 (0.1 to 1.5) | 0.9 (0.3 to 1.7) | 1.1 (0.4 to 2.2) | 0.8 (0.2 to 1.5) | 22.5 (-10.4 to 78.2) | 23.7 (-23.1 to 88.5) | 16.9 (-24 to 93.5) |
|  |  |  | All ages | 31.5 (8 to 66.1) | 17 (4.7 to 38.3) | 14.5 (2.7 to 32.9) | 50.9 (15.7 to 94.4) | 30.5 (10.5 to 61.1) | 20.5 (4.8 to 41.7) | 61.7 (16.9 to 131.3) | 79.5 (16.4 to 185.8) | 40.9 (-10.2 to 133.2) |
|  | Lead exposure | DALYs (Disability-Adjusted Life Years) | Age-standardized | 3.6 (-0.6 to 10) | 3.1 (-0.5 to 8.8) | 4 (-0.7 to 11.7) | 4.6 (-0.7 to 12.1) | 4.2 (-0.7 to 10.9) | 5 (-0.7 to 13.9) | 28 (4.8 to 58.5) | 35.8 (2.7 to 76.1) | 24.6 (-2.4 to 62.6) |
|  |  |  | All ages | 194.2 (-31.4 to 542.6) | 79.3 (-12.2 to 222.9) | 114.9 (-19 to 332.9) | 309.7 (-46.5 to 813.7) | 150.5 (-23.2 to 374.2) | 159.2 (-23.4 to 441.9) | 59.5 (29.9 to 99.3) | 89.8 (40.5 to 152) | 38.5 (6.6 to 78.8) |
|  |  | Deaths | Age-standardized | 0.2 (0 to 0.5) | 0.1 (0 to 0.4) | 0.2 (0 to 0.6) | 0.2 (0 to 0.6) | 0.2 (0 to 0.5) | 0.2 (0 to 0.7) | 38.5 (7.6 to 94.5) | 49.6 (6.9 to 113.8) | 30.2 (-6.5 to 96.7) |
|  |  |  | All ages | 6.4 (-1 to 18.6) | 2.7 (-0.4 to 8.2) | 3.7 (-0.6 to 12.5) | 11.4 (-1.9 to 31) | 5.8 (-0.9 to 14.8) | 5.7 (-0.9 to 17.2) | 78.9 (33.1 to 153.9) | 110.8 (45.8 to 210.5) | 54.8 (7.9 to 135.3) |
|  | Smoking | DALYs (Disability-Adjusted Life Years) | Age-standardized | 0.9 (0.4 to 1.5) | 0.3 (0.1 to 0.5) | 1.4 (0.7 to 2.5) | 1.4 (0.7 to 2.2) | 0.4 (0.2 to 0.7) | 2.5 (1.3 to 4) | 56.2 (20.5 to 100.9) | 55.6 (10.3 to 121) | 75.3 (30.8 to 137.4) |
|  |  |  | All ages | 56 (27.3 to 96.4) | 8 (3.7 to 15.4) | 48 (23.3 to 84.1) | 133.4 (72.8 to 215.6) | 21.7 (10.5 to 37.6) | 111.6 (59.8 to 184.7) | 138.1 (82.5 to 210.5) | 171.9 (89.4 to 292.1) | 132.4 (72.4 to 208.3) |
|  |  | Deaths | Age-standardized | 0 (0 to 0.1) | 0 (0 to 0) | 0 (0 to 0.1) | 0 (0 to 0.1) | 0 (0 to 0) | 0.1 (0 to 0.1) | 57.3 (9.3 to 133) | 55.1 (-1.8 to 154) | 72.8 (14.1 to 167) |
|  |  |  | All ages | 1.4 (0.5 to 2.8) | 0.2 (0.1 to 0.5) | 1.2 (0.4 to 2.5) | 3 (1.4 to 5.2) | 0.5 (0.2 to 1) | 2.5 (1.1 to 4.5) | 111.8 (46.5 to 220.5) | 146 (61.4 to 283.9) | 105.7 (34.8 to 219.6) |
| Bahrain | All risk factors | DALYs (Disability-Adjusted Life Years) | Age-standardized | 41.6 (16.7 to 68.7) | 48.6 (18 to 83.7) | 31.4 (12.2 to 55.3) | 40 (18.1 to 64.2) | 47.9 (19.9 to 81.4) | 30.2 (15.3 to 49) | -3.9 (-26.3 to 30.1) | -1.5 (-28.4 to 39.3) | -3.8 (-30.1 to 36.5) |
|  |  |  | All ages | 39.2 (18.3 to 61.8) | 22.3 (9.1 to 36.4) | 16.9 (8.1 to 27.5) | 195.1 (107.4 to 296.3) | 100.3 (48.2 to 166) | 94.8 (53.3 to 147.7) | 397.2 (285.9 to 575.7) | 349.8 (224.6 to 550.5) | 459.6 (306.9 to 689.6) |
|  |  | Deaths | Age-standardized | 2.5 (0.9 to 4.4) | 3 (1 to 5.6) | 1.5 (0.4 to 3) | 2.3 (0.9 to 4) | 3 (1 to 5.5) | 1.4 (0.5 to 2.7) | -6.6 (-34.2 to 34.3) | 0.3 (-33.2 to 49.3) | -10.1 (-40.1 to 51.9) |
|  |  |  | All ages | 1.4 (0.5 to 2.3) | 1 (0.3 to 1.7) | 0.4 (0.2 to 0.8) | 6.3 (2.7 to 10.5) | 4.4 (1.6 to 7.7) | 1.9 (1 to 3.1) | 350.8 (222.7 to 542.7) | 354.4 (204.7 to 574.8) | 342.9 (176.1 to 668.9) |
|  | Alcohol use | DALYs (Disability-Adjusted Life Years) | Age-standardized | 0.5 (0.3 to 0.7) | 0.1 (0.1 to 0.2) | 0.9 (0.5 to 1.3) | 0.2 (0.1 to 0.3) | 0 (0 to 0.1) | 0.4 (0.2 to 0.6) | -51.7 (-65.6 to -33.7) | -67.7 (-82 to -47.3) | -53.9 (-67.9 to -32.5) |
|  |  |  | All ages | 0.7 (0.4 to 1) | 0.1 (0 to 0.1) | 0.6 (0.4 to 0.9) | 2.1 (1.3 to 3.1) | 0.1 (0.1 to 0.2) | 2 (1.2 to 2.9) | 189.9 (103.9 to 304.5) | 52.6 (-9.8 to 141.8) | 204.2 (112.3 to 335.2) |
|  |  | Deaths | Age-standardized | 0 (0 to 0) | 0 (0 to 0) | 0 (0 to 0.1) | 0 (0 to 0) | 0 (0 to 0) | 0 (0 to 0) | -58.5 (-74.3 to -35.8) | -68 (-84 to -38) | -60.6 (-77.3 to -31.8) |
|  |  |  | All ages | 0 (0 to 0) | 0 (0 to 0) | 0 (0 to 0) | 0 (0 to 0) | 0 (0 to 0) | 0 (0 to 0) | 99.9 (20.1 to 235.9) | 44.6 (-20.3 to 149.5) | 110.8 (19.4 to 289.9) |
|  | Diet high in sodium | DALYs (Disability-Adjusted Life Years) | Age-standardized | 0.5 (0 to 3.3) | 0.4 (0 to 3.3) | 0.7 (0 to 3.7) | 0.5 (0 to 2.9) | 0.3 (0 to 2.8) | 0.6 (0 to 3.2) | -15.5 (-93.7 to 760) | -19.1 (-3177.3 to 2590.1) | -16 (-95.2 to 782) |
|  |  |  | All ages | 0.6 (0 to 3.7) | 0.2 (0 to 1.7) | 0.4 (0 to 2.1) | 2.8 (0 to 16.1) | 0.8 (0 to 6.2) | 2 (0 to 10.4) | 341.4 (-67.3 to 2579.2) | 269.6 (-4211.8 to 9630.5) | 379.5 (-67.4 to 2582.2) |
|  |  | Deaths | Age-standardized | 0 (0 to 0.2) | 0 (0 to 0.2) | 0 (0 to 0.2) | 0 (0 to 0.1) | 0 (0 to 0.2) | 0 (0 to 0.1) | -19.8 (-97.9 to 760.4) | -19 (-5441.9 to 2267.9) | -22.3 (-98.5 to 846.4) |
|  |  |  | All ages | 0 (0 to 0.1) | 0 (0 to 0.1) | 0 (0 to 0.1) | 0.1 (0 to 0.5) | 0 (0 to 0.2) | 0 (0 to 0.2) | 261.7 (-82.4 to 2843.6) | 256.1 (-3648.9 to 7777.9) | 266.5 (-83.6 to 2861.1) |
|  | High body-mass index | DALYs (Disability-Adjusted Life Years) | Age-standardized | 8.2 (2.9 to 17) | 12 (4.1 to 24.5) | 3.3 (1.2 to 7.2) | 16.9 (6.3 to 32.4) | 22.5 (8 to 42.3) | 10.4 (4.2 to 20.1) | 106.8 (40.7 to 199.9) | 87.1 (22.1 to 176.7) | 211.7 (85.7 to 414.4) |
|  |  |  | All ages | 9.4 (3.4 to 18.5) | 6.6 (2.3 to 13.4) | 2.8 (1 to 5.4) | 89.4 (37.1 to 162) | 51 (20.1 to 92.6) | 38.4 (15.9 to 70) | 855.6 (622.3 to 1205.5) | 671.6 (454.6 to 974.9) | 1297.4 (865.5 to 2038.3) |
|  |  | Deaths | Age-standardized | 0.4 (0.1 to 1) | 0.6 (0.2 to 1.5) | 0.1 (0 to 0.3) | 1 (0.3 to 1.9) | 1.4 (0.4 to 2.8) | 0.4 (0.1 to 1) | 115.4 (27.5 to 270.6) | 108.5 (18.1 to 264.4) | 272.1 (77.5 to 726) |
|  |  |  | All ages | 0.3 (0.1 to 0.6) | 0.2 (0.1 to 0.5) | 0.1 (0 to 0.1) | 2.7 (1 to 5.2) | 2 (0.7 to 4) | 0.7 (0.3 to 1.3) | 814.9 (523 to 1259.8) | 732 (428.6 to 1181.2) | 1210.1 (636.7 to 2333.9) |
|  | High systolic blood pressure | DALYs (Disability-Adjusted Life Years) | Age-standardized | 34.9 (10.3 to 61.2) | 41.3 (11.5 to 74.8) | 25.5 (7.4 to 49.1) | 29.6 (9.4 to 53.2) | 35.7 (10.3 to 67.7) | 22 (7.1 to 40) | -15.3 (-36 to 7.2) | -13.6 (-38.2 to 17.2) | -13.8 (-37.6 to 22.4) |
|  |  |  | All ages | 30.7 (9.9 to 53.1) | 18.2 (5.4 to 32.4) | 12.5 (3.8 to 22.9) | 134.2 (44.3 to 235.6) | 72.2 (22.8 to 132.5) | 62.1 (19.2 to 110.3) | 337.3 (226.7 to 464.5) | 296.3 (175.2 to 468.7) | 397 (255 to 612.8) |
|  |  | Deaths | Age-standardized | 2.2 (0.6 to 4) | 2.6 (0.7 to 4.9) | 1.3 (0.3 to 2.7) | 1.8 (0.5 to 3.3) | 2.3 (0.6 to 4.6) | 1.1 (0.3 to 2.3) | -18.2 (-42.2 to 9.6) | -12.5 (-44.1 to 24.2) | -19.7 (-48 to 33.4) |
|  |  |  | All ages | 1.2 (0.3 to 2.1) | 0.8 (0.2 to 1.5) | 0.3 (0.1 to 0.7) | 4.6 (1.5 to 8.7) | 3.3 (0.9 to 6.5) | 1.4 (0.5 to 2.6) | 301.1 (179.2 to 459.7) | 301.8 (162.2 to 484.5) | 299.6 (150.6 to 604.7) |
|  | Lead exposure | DALYs (Disability-Adjusted Life Years) | Age-standardized | 2.6 (-0.4 to 7) | 2.7 (-0.4 to 7.4) | 2.4 (-0.3 to 6.4) | 2.3 (-0.3 to 6) | 2.4 (-0.3 to 6.5) | 2.1 (-0.3 to 5.6) | -12.7 (-31.8 to 7.6) | -12 (-37.5 to 17.1) | -12.5 (-32.4 to 17) |
|  |  |  | All ages | 2.4 (-0.3 to 6.4) | 1.2 (-0.2 to 3.3) | 1.2 (-0.2 to 3.1) | 9.6 (-1.2 to 25.3) | 4.5 (-0.6 to 12.2) | 5.1 (-0.6 to 14.2) | 300.6 (214.1 to 405.6) | 271.9 (156.2 to 397.8) | 329.4 (223.8 to 481.6) |
|  |  | Deaths | Age-standardized | 0.2 (0 to 0.4) | 0.2 (0 to 0.5) | 0.1 (0 to 0.4) | 0.1 (0 to 0.4) | 0.2 (0 to 0.5) | 0.1 (0 to 0.3) | -10.7 (-35.6 to 16.9) | -6.2 (-38.2 to 29.5) | -12.7 (-36.8 to 39.3) |
|  |  |  | All ages | 0.1 (0 to 0.2) | 0.1 (0 to 0.1) | 0 (0 to 0.1) | 0.3 (-0.1 to 0.9) | 0.2 (0 to 0.6) | 0.1 (0 to 0.4) | 304.4 (186.1 to 447.3) | 308.1 (156.1 to 476.4) | 298.3 (169.1 to 550) |
|  | Smoking | DALYs (Disability-Adjusted Life Years) | Age-standardized | 3.6 (1.9 to 6) | 2 (0.9 to 3.6) | 5.3 (2.7 to 9) | 2.8 (1.5 to 4.6) | 1.4 (0.6 to 2.7) | 4.2 (2.3 to 6.7) | -21.4 (-41.5 to 1.9) | -33.6 (-61.1 to 11.3) | -21.5 (-42.4 to 4.2) |
|  |  |  | All ages | 5.1 (2.9 to 8.1) | 1.2 (0.6 to 2) | 3.9 (2.1 to 6.1) | 22 (12.1 to 35.5) | 3.6 (1.8 to 6.4) | 18.4 (10.1 to 28.6) | 332.5 (223.3 to 453.2) | 193.4 (83 to 360.9) | 376.3 (243 to 541.1) |
|  |  | Deaths | Age-standardized | 0.1 (0.1 to 0.3) | 0.1 (0 to 0.2) | 0.2 (0.1 to 0.4) | 0.1 (0 to 0.2) | 0.1 (0 to 0.2) | 0.2 (0.1 to 0.3) | -26.1 (-50.8 to 12.8) | -28.8 (-66.7 to 40.6) | -29 (-54.9 to 13.4) |
|  |  |  | All ages | 0.1 (0.1 to 0.2) | 0 (0 to 0.1) | 0.1 (0 to 0.1) | 0.4 (0.2 to 0.7) | 0.1 (0 to 0.3) | 0.3 (0.2 to 0.5) | 234.3 (119 to 400.1) | 199.4 (60.4 to 433.4) | 252.5 (114.1 to 509.9) |
| Djibouti | All risk factors | DALYs (Disability-Adjusted Life Years) | Age-standardized | 24.9 (11.3 to 41.6) | 23.2 (8.3 to 42.5) | 26.8 (13.5 to 42.4) | 30.4 (12.9 to 51.1) | 28.5 (10 to 51.3) | 32.3 (15.2 to 53.1) | 22.1 (-6.5 to 58.7) | 23.2 (-9.8 to 67.9) | 20.3 (-10.9 to 63.9) |
|  |  |  | All ages | 23.8 (11.4 to 38.7) | 10.7 (3.8 to 19.2) | 13.1 (7.1 to 20.4) | 136 (60.7 to 224.5) | 58.2 (20.9 to 103.9) | 77.8 (38.3 to 124.8) | 471.5 (342.6 to 637) | 445.7 (305.7 to 651.3) | 492.4 (343 to 713.8) |
|  |  | Deaths | Age-standardized | 1.1 (0.4 to 1.9) | 1 (0.3 to 2) | 1.2 (0.5 to 2) | 1.3 (0.5 to 2.4) | 1.3 (0.4 to 2.5) | 1.4 (0.6 to 2.5) | 23.3 (-14.9 to 75.7) | 23.8 (-18.6 to 88.5) | 21.6 (-20.8 to 88.2) |
|  |  |  | All ages | 0.7 (0.3 to 1.1) | 0.3 (0.1 to 0.7) | 0.3 (0.2 to 0.6) | 3.8 (1.5 to 6.8) | 1.8 (0.6 to 3.5) | 2 (0.9 to 3.5) | 467.7 (288.1 to 715.2) | 437.9 (249.8 to 697.1) | 496.5 (295 to 859.7) |
|  | Alcohol use | DALYs (Disability-Adjusted Life Years) | Age-standardized | 0.5 (0.2 to 1) | 0.2 (0 to 0.3) | 0.9 (0.3 to 1.8) | 0.1 (0 to 0.2) | 0 (0 to 0.1) | 0.1 (0 to 0.2) | -86.1 (-94.5 to -66.7) | -82.1 (-93.8 to -52) | -87.8 (-95.4 to -70.5) |
|  |  |  | All ages | 0.7 (0.2 to 1.3) | 0.1 (0 to 0.2) | 0.6 (0.2 to 1.1) | 0.5 (0.1 to 1.1) | 0.1 (0 to 0.2) | 0.4 (0.1 to 0.9) | -28.3 (-70.3 to 58) | -15.2 (-68 to 131.1) | -30.5 (-70.9 to 52.4) |
|  |  | Deaths | Age-standardized | 0 (0 to 0) | 0 (0 to 0) | 0 (0 to 0.1) | 0 (0 to 0) | 0 (0 to 0) | 0 (0 to 0) | -87.9 (-95.6 to -68.2) | -83.9 (-95.5 to -54.1) | -89.6 (-96.7 to -69.9) |
|  |  |  | All ages | 0 (0 to 0) | 0 (0 to 0) | 0 (0 to 0) | 0 (0 to 0) | 0 (0 to 0) | 0 (0 to 0) | -37.7 (-76 to 60.9) | -26.2 (-76.9 to 109) | -39.7 (-78.2 to 58.2) |
|  | Diet high in sodium | DALYs (Disability-Adjusted Life Years) | Age-standardized | 3.4 (0.2 to 10.3) | 3.1 (0.1 to 9.3) | 3.8 (0.2 to 11.3) | 2.6 (0.1 to 8.3) | 2.6 (0.1 to 8.7) | 2.6 (0 to 9.2) | -24.3 (-78.1 to -0.5) | -15.6 (-64 to 21.9) | -31.9 (-90.9 to -4.3) |
|  |  |  | All ages | 3 (0.2 to 8.9) | 1.4 (0.1 to 4.3) | 1.6 (0.1 to 4.6) | 10.5 (0.2 to 35.2) | 5.3 (0.2 to 18) | 5.2 (0 to 18.4) | 252.5 (-3.8 to 365.7) | 281.5 (68.7 to 461) | 227.1 (-61.6 to 394.5) |
|  |  | Deaths | Age-standardized | 0.2 (0 to 0.5) | 0.1 (0 to 0.4) | 0.2 (0 to 0.6) | 0.1 (0 to 0.4) | 0.1 (0 to 0.4) | 0.1 (0 to 0.5) | -23.6 (-80.7 to 7.3) | -18.2 (-74 to 28.5) | -29.7 (-90.9 to 13.5) |
|  |  |  | All ages | 0.1 (0 to 0.3) | 0 (0 to 0.1) | 0 (0 to 0.1) | 0.3 (0 to 1.1) | 0.2 (0 to 0.5) | 0.2 (0 to 0.6) | 252.9 (-4.7 to 414.8) | 265.3 (31.9 to 463.9) | 241.5 (-54.4 to 450.5) |
|  | High body-mass index | DALYs (Disability-Adjusted Life Years) | Age-standardized | 0.1 (0 to 0.4) | 0.3 (0 to 0.8) | 0 (-0.1 to 0.1) | 0.8 (0.2 to 1.7) | 1.1 (0.2 to 2.5) | 0.5 (0.1 to 1.5) | 614.5 (-7471.3 to 6093.9) | 303.3 (-823.6 to 3050.7) | -1217.2 (-7402.7 to 4502.1) |
|  |  |  | All ages | 0.2 (0 to 0.6) | 0.2 (0 to 0.5) | 0 (0 to 0.1) | 4.6 (1.1 to 10) | 3 (0.8 to 6.5) | 1.7 (0.3 to 4.2) | 2558.4 (-25736.3 to 19093.7) | 1496 (662.1 to 9115.8) | -13496.2 (-51334.1 to 80125.5) |
|  |  | Deaths | Age-standardized | 0 (0 to 0) | 0 (0 to 0) | 0 (0 to 0) | 0 (0 to 0.1) | 0 (0 to 0.1) | 0 (0 to 0.1) | 1179.9 (-10920 to 11109.4) | 403.3 (-4485.4 to 10586) | -759.1 (-3342.9 to 1500.3) |
|  |  |  | All ages | 0 (0 to 0) | 0 (0 to 0) | 0 (0 to 0) | 0.1 (0 to 0.2) | 0.1 (0 to 0.2) | 0 (0 to 0.1) | 3190 (-30326.3 to 34659) | 1606 (-6309.9 to 16257.9) | -6630.4 (-51790 to 35922.1) |
|  | High systolic blood pressure | DALYs (Disability-Adjusted Life Years) | Age-standardized | 21.1 (6.9 to 38.3) | 22.1 (7.4 to 41.6) | 19.8 (6 to 35.7) | 27 (9.1 to 47.6) | 27.4 (9 to 50.5) | 26.5 (8.2 to 46.9) | 28 (-3.5 to 67.9) | 23.9 (-10.6 to 74.2) | 34 (-3.9 to 99.3) |
|  |  |  | All ages | 19 (6.3 to 34.5) | 10 (3.2 to 18.8) | 9 (2.8 to 16.5) | 115.6 (38.9 to 205) | 55.1 (18 to 100.9) | 60.5 (19.2 to 108.7) | 507.2 (366 to 711.8) | 448.9 (296.4 to 671.5) | 572.2 (381.5 to 927.8) |
|  |  | Deaths | Age-standardized | 1 (0.3 to 1.8) | 1 (0.3 to 2) | 0.9 (0.3 to 1.7) | 1.2 (0.4 to 2.3) | 1.2 (0.4 to 2.5) | 1.2 (0.4 to 2.4) | 28.5 (-12.7 to 87.5) | 24.6 (-18.8 to 91.3) | 34.8 (-14.5 to 121.7) |
|  |  |  | All ages | 0.6 (0.2 to 1) | 0.3 (0.1 to 0.6) | 0.2 (0.1 to 0.5) | 3.4 (1.1 to 6.3) | 1.7 (0.5 to 3.4) | 1.7 (0.5 to 3.2) | 498.3 (311.5 to 787.8) | 441.9 (247.5 to 716.5) | 569.6 (326.3 to 1061.9) |
|  | Lead exposure | DALYs (Disability-Adjusted Life Years) | Age-standardized | 3 (-0.4 to 7.9) | 2 (-0.3 to 5.1) | 4.4 (-0.6 to 11.6) | 2.8 (-0.4 to 7) | 1.9 (-0.3 to 4.8) | 3.8 (-0.6 to 9.5) | -8 (-26.5 to 17.3) | -2.5 (-26.2 to 25.1) | -13.7 (-34 to 15.5) |
|  |  |  | All ages | 2.7 (-0.4 to 6.9) | 0.9 (-0.1 to 2.3) | 1.8 (-0.3 to 4.7) | 11 (-1.5 to 28.2) | 3.6 (-0.5 to 9.3) | 7.3 (-1.1 to 19.1) | 309.2 (223.7 to 421.8) | 318.4 (217.8 to 429.8) | 304.9 (210.2 to 432.7) |
|  |  | Deaths | Age-standardized | 0.1 (0 to 0.4) | 0.1 (0 to 0.2) | 0.2 (0 to 0.6) | 0.1 (0 to 0.3) | 0.1 (0 to 0.2) | 0.2 (0 to 0.5) | -3.3 (-29.9 to 34) | 0.4 (-32.1 to 42.2) | -9.3 (-36.9 to 38.8) |
|  |  |  | All ages | 0.1 (0 to 0.2) | 0 (0 to 0.1) | 0.1 (0 to 0.2) | 0.4 (-0.1 to 0.9) | 0.1 (0 to 0.3) | 0.2 (0 to 0.6) | 330.2 (206.3 to 522.1) | 331.3 (184.1 to 510.4) | 329.6 (196.5 to 571.2) |
|  | Smoking | DALYs (Disability-Adjusted Life Years) | Age-standardized | 4.5 (2.6 to 6.9) | 1.1 (0.5 to 1.9) | 8.3 (4.7 to 13.1) | 4.4 (2.4 to 6.9) | 1 (0.5 to 1.8) | 7.8 (4.2 to 12.3) | -2.1 (-25.2 to 28.7) | -14.3 (-45.6 to 33.7) | -6.6 (-31.5 to 25.5) |
|  |  |  | All ages | 5.5 (3.2 to 8.5) | 0.6 (0.3 to 1.1) | 4.8 (2.8 to 7.6) | 25.4 (13.6 to 39.9) | 2.5 (1.2 to 4.3) | 23 (12.5 to 36.4) | 364.2 (258.9 to 512.6) | 284.6 (152.6 to 468) | 374.8 (257.9 to 532.1) |
|  |  | Deaths | Age-standardized | 0.2 (0.1 to 0.3) | 0 (0 to 0.1) | 0.3 (0.2 to 0.6) | 0.2 (0.1 to 0.3) | 0 (0 to 0.1) | 0.3 (0.1 to 0.5) | -4.4 (-36.4 to 46.4) | -16.4 (-61.4 to 56.5) | -9.7 (-40.7 to 41.7) |
|  |  |  | All ages | 0.1 (0.1 to 0.2) | 0 (0 to 0) | 0.1 (0.1 to 0.2) | 0.6 (0.3 to 0.9) | 0.1 (0 to 0.1) | 0.5 (0.2 to 0.8) | 346 (193.1 to 576.4) | 261.8 (95.9 to 534.2) | 357.7 (193.2 to 615.4) |
| Egypt | All risk factors | DALYs (Disability-Adjusted Life Years) | Age-standardized | 25.2 (12.7 to 38.8) | 30.2 (14.3 to 51.2) | 21.8 (11.5 to 33.7) | 33.3 (18.7 to 47.7) | 44.3 (23.5 to 64.3) | 29.8 (17.1 to 42.7) | 32 (4.5 to 70.7) | 46.8 (2.8 to 112.2) | 36.5 (5.8 to 87.9) |
|  |  |  | All ages | 4344.6 (2245.9 to 6526.9) | 2194.6 (1046.8 to 3459.1) | 2149.9 (1177.3 to 3328.3) | 14051.2 (8082.1 to 20234.9) | 6529.9 (3690.9 to 9615.1) | 7521.3 (4377.4 to 10867.3) | 223.4 (165.4 to 310.9) | 197.5 (120 to 306.1) | 249.8 (172.8 to 358.2) |
|  |  | Deaths | Age-standardized | 1.3 (0.7 to 2.2) | 1.9 (0.8 to 3.5) | 0.9 (0.4 to 1.6) | 1.5 (0.9 to 2.2) | 2.7 (1.4 to 4.1) | 1.1 (0.7 to 1.7) | 15.5 (-17.4 to 67.4) | 46.3 (-11 to 142.2) | 22.7 (-17.7 to 106) |
|  |  |  | All ages | 153.1 (78.1 to 247.6) | 90.8 (42.9 to 154.9) | 62.2 (31 to 101.2) | 426.9 (255.5 to 606.9) | 221.8 (121.2 to 328.1) | 205 (123.9 to 296.8) | 178.8 (103.5 to 295.9) | 144.2 (55.9 to 269.2) | 229.4 (126.9 to 444.5) |
|  | Alcohol use | DALYs (Disability-Adjusted Life Years) | Age-standardized | 0 (0 to 0.1) | 0 (0 to 0) | 0.1 (0 to 0.1) | 0.1 (0 to 0.1) | 0 (0 to 0) | 0.1 (0.1 to 0.2) | 80.2 (18.9 to 203.2) | 24.7 (-43.8 to 203.4) | 73.6 (17.3 to 182.5) |
|  |  |  | All ages | 10.2 (5.6 to 16.2) | 0.9 (0.3 to 1.7) | 9.3 (5 to 14.8) | 41.5 (25.3 to 62.3) | 2.3 (1.2 to 3.7) | 39.2 (23.8 to 59) | 306.9 (175.9 to 553.7) | 154.7 (34.7 to 486.8) | 321.9 (185.2 to 577.8) |
|  |  | Deaths | Age-standardized | 0 (0 to 0) | 0 (0 to 0) | 0 (0 to 0) | 0 (0 to 0) | 0 (0 to 0) | 0 (0 to 0) | 75.6 (-5.2 to 265.4) | 28.6 (-54.3 to 271.7) | 63 (-9.4 to 244.1) |
|  |  |  | All ages | 0.2 (0.1 to 0.4) | 0 (0 to 0.1) | 0.2 (0.1 to 0.3) | 0.8 (0.5 to 1.3) | 0.1 (0 to 0.1) | 0.8 (0.4 to 1.2) | 286.2 (119.9 to 670.2) | 123 (-5 to 480.7) | 310.1 (134.3 to 716.8) |
|  | Diet high in sodium | DALYs (Disability-Adjusted Life Years) | Age-standardized | 0.4 (0 to 2.4) | 0.3 (0 to 2.3) | 0.5 (0 to 2.7) | 0.4 (0 to 2.6) | 0.3 (0 to 2.3) | 0.5 (0 to 2.9) | 3.2 (-94.4 to 557.2) | 3.2 (-1751 to 2378) | 2.5 (-94.6 to 536.1) |
|  |  |  | All ages | 79.3 (0 to 443) | 24.9 (0 to 180.3) | 54.4 (0 to 279) | 194.3 (0 to 1123.7) | 53.3 (0 to 396.8) | 141 (0 to 743.7) | 145 (-82.1 to 1268.2) | 114.4 (-2573.4 to 5064.2) | 159 (-82.4 to 1273.3) |
|  |  | Deaths | Age-standardized | 0 (0 to 0.1) | 0 (0 to 0.1) | 0 (0 to 0.1) | 0 (0 to 0.1) | 0 (0 to 0.1) | 0 (0 to 0.1) | -6 (-97 to 514.5) | 4.2 (-1851.3 to 3631.8) | -7.6 (-97.1 to 535.5) |
|  |  |  | All ages | 2.3 (0 to 14.3) | 0.9 (0 to 7) | 1.4 (0 to 7.6) | 5.1 (0 to 32.5) | 1.7 (0 to 12.5) | 3.5 (0 to 20.3) | 118.6 (-90.1 to 1196.6) | 84.3 (-1643.3 to 6294.9) | 139.9 (-90.2 to 1236) |
|  | High body-mass index | DALYs (Disability-Adjusted Life Years) | Age-standardized | 8.1 (3.2 to 14.3) | 12.2 (4.7 to 22.5) | 4.9 (1.8 to 9.6) | 16.1 (7.1 to 26.8) | 23.7 (10.8 to 39.1) | 12.8 (5.4 to 22) | 98.8 (42.7 to 172.4) | 93.8 (30.5 to 181.5) | 161.4 (65.8 to 360.7) |
|  |  |  | All ages | 1437.6 (589.1 to 2550.5) | 971.9 (399.5 to 1721.3) | 465.6 (169.6 to 882.5) | 6842.5 (3074.3 to 11425.1) | 3681.8 (1736.4 to 5952.8) | 3160.7 (1359.8 to 5546) | 376 (262.1 to 516.6) | 278.8 (178.9 to 406.8) | 578.8 (372.1 to 916) |
|  |  | Deaths | Age-standardized | 0.4 (0.2 to 0.8) | 0.7 (0.3 to 1.4) | 0.2 (0.1 to 0.5) | 0.7 (0.3 to 1.2) | 1.4 (0.6 to 2.4) | 0.5 (0.2 to 0.9) | 74.1 (10.3 to 174.2) | 100 (18.6 to 242.5) | 129.2 (19.6 to 498.3) |
|  |  |  | All ages | 50.3 (20.3 to 90.2) | 36.4 (14.7 to 68.1) | 13.9 (4.8 to 27.4) | 208.7 (93 to 351.1) | 119.2 (54.8 to 197.1) | 89.5 (38.4 to 155.2) | 314.7 (171 to 508) | 227.5 (102.3 to 405.9) | 542.5 (265.6 to 1129) |
|  | High systolic blood pressure | DALYs (Disability-Adjusted Life Years) | Age-standardized | 18.4 (5.8 to 31.8) | 23.4 (7.3 to 44.4) | 14.8 (4.6 to 27) | 22.6 (7.5 to 37.6) | 32.1 (11 to 52.9) | 19.1 (6 to 32.1) | 22.6 (-5 to 62.3) | 37 (-9.7 to 107.5) | 28.7 (-4.3 to 81.3) |
|  |  |  | All ages | 3025.7 (923.8 to 5189) | 1645 (506.4 to 2852.5) | 1380.7 (408.6 to 2489.4) | 9329.5 (3050.3 to 15467.8) | 4633 (1500.2 to 7574.1) | 4696.5 (1503 to 8051.1) | 208.3 (144 to 293.9) | 181.6 (101.6 to 301.7) | 240.2 (160.8 to 364.8) |
|  |  | Deaths | Age-standardized | 1 (0.3 to 1.9) | 1.5 (0.5 to 3) | 0.7 (0.2 to 1.3) | 1 (0.4 to 1.7) | 2 (0.7 to 3.4) | 0.7 (0.2 to 1.2) | 5.1 (-29.1 to 58) | 35.1 (-24.9 to 140.6) | 12.7 (-27.9 to 97.6) |
|  |  |  | All ages | 113.5 (35.7 to 208.7) | 70.7 (22.3 to 134.8) | 42.9 (12.5 to 81.7) | 292.6 (100.8 to 485.3) | 160.7 (56.3 to 272.3) | 131.9 (44.9 to 220.8) | 157.7 (80.4 to 268.1) | 127.4 (41.5 to 265.3) | 207.7 (103.3 to 423.9) |
|  | Lead exposure | DALYs (Disability-Adjusted Life Years) | Age-standardized | 4 (-0.6 to 10) | 4.1 (-0.7 to 10.8) | 3.9 (-0.5 to 9.9) | 4.3 (-0.6 to 10.4) | 5 (-0.7 to 12.6) | 4.2 (-0.5 to 10.3) | 7.3 (-12.7 to 32.6) | 21.9 (-14.1 to 63.4) | 7.5 (-12.6 to 40.6) |
|  |  |  | All ages | 672.3 (-93.9 to 1745.5) | 308.7 (-47.6 to 812.8) | 363.6 (-48.3 to 921.2) | 1596.2 (-201.7 to 3984.8) | 650.8 (-87.6 to 1624.6) | 945.4 (-115.9 to 2359.4) | 137.4 (96.3 to 191.7) | 110.8 (55.1 to 169.8) | 160 (112.1 to 234.7) |
|  |  | Deaths | Age-standardized | 0.2 (0 to 0.5) | 0.3 (0 to 0.7) | 0.2 (0 to 0.4) | 0.2 (0 to 0.5) | 0.3 (-0.1 to 0.8) | 0.2 (0 to 0.4) | 1.6 (-24.9 to 42.3) | 31.9 (-17.1 to 93.6) | 2 (-27.8 to 59.6) |
|  |  |  | All ages | 23.3 (-3.8 to 58.8) | 12.2 (-2.1 to 31.4) | 11.1 (-1.6 to 29.1) | 53.8 (-8 to 131.7) | 23.9 (-3.7 to 60) | 29.9 (-4.3 to 71.5) | 130.4 (70.1 to 222.7) | 95.1 (25.2 to 181.5) | 169.3 (90.3 to 321.4) |
|  | Smoking | DALYs (Disability-Adjusted Life Years) | Age-standardized | 3.3 (1.9 to 5) | 0.4 (0.2 to 0.7) | 5.7 (3.3 to 8.8) | 3.8 (2.2 to 6) | 0.4 (0.2 to 0.7) | 6.3 (3.5 to 9.8) | 16.5 (-8.8 to 52.6) | 4.2 (-49.1 to 100.3) | 9.2 (-14.6 to 42.2) |
|  |  |  | All ages | 705.7 (396.5 to 1070.9) | 27.4 (12.9 to 46.5) | 678.3 (381.3 to 1037.2) | 1919.6 (1101.8 to 2903.9) | 54.5 (28.8 to 89.1) | 1865.1 (1074.3 to 2831.1) | 172 (120 to 238.7) | 99.3 (27.4 to 217.7) | 175 (120.6 to 246.4) |
|  |  | Deaths | Age-standardized | 0.1 (0.1 to 0.2) | 0 (0 to 0) | 0.2 (0.1 to 0.3) | 0.1 (0.1 to 0.2) | 0 (0 to 0) | 0.2 (0.1 to 0.3) | 9.6 (-27.2 to 77.1) | 7.7 (-59.4 to 153.1) | -2.4 (-36 to 60.9) |
|  |  |  | All ages | 16.7 (9.1 to 26.2) | 1 (0.4 to 2.1) | 15.6 (8.5 to 24.7) | 41.6 (23.5 to 61.5) | 1.8 (0.9 to 3.2) | 39.8 (22.3 to 59) | 149.1 (77.4 to 286.4) | 72.6 (-15.9 to 236.4) | 154.2 (78.5 to 302.9) |
| Iran (Islamic Republic of) | All risk factors | DALYs (Disability-Adjusted Life Years) | Age-standardized | 22.6 (10.1 to 35.6) | 25.9 (11.5 to 41.7) | 18.9 (8.1 to 29.8) | 27.4 (14.7 to 40.8) | 32.2 (16.8 to 49.5) | 23 (12.1 to 35.3) | 21 (-2.1 to 61.4) | 24.1 (-6.6 to 66.3) | 21.9 (2.9 to 69.3) |
|  |  |  | All ages | 4046.2 (1834.1 to 6377.4) | 2210.8 (993.9 to 3499.2) | 1835.4 (827.8 to 2919.4) | 18252.5 (9774.3 to 27579.4) | 10305.9 (5381.3 to 15540.2) | 7946.6 (4210.4 to 12110.5) | 351.1 (274.3 to 481.3) | 366.2 (259.2 to 510.8) | 333 (268.3 to 470.6) |
|  |  | Deaths | Age-standardized | 1 (0.4 to 1.7) | 1.3 (0.6 to 2.2) | 0.7 (0.2 to 1.1) | 1.2 (0.6 to 1.8) | 1.7 (0.8 to 2.6) | 0.7 (0.4 to 1.1) | 17 (-13.8 to 71.4) | 27.6 (-12.7 to 85.1) | 13.1 (-12.7 to 97.3) |
|  |  |  | All ages | 129 (56.3 to 209.6) | 86.1 (38.5 to 144.7) | 42.9 (16.5 to 70.5) | 704.2 (368 to 1079) | 468.9 (228.4 to 722) | 235.3 (118.8 to 358.2) | 445.9 (303.9 to 686) | 444.8 (277.8 to 675.6) | 448.1 (316.8 to 818.7) |
|  | Alcohol use | DALYs (Disability-Adjusted Life Years) | Age-standardized | 0 (0 to 0) | 0 (0 to 0) | 0 (0 to 0) | 0.2 (0.1 to 0.3) | 0 (0 to 0.1) | 0.3 (0.2 to 0.5) | NA | NA | NA |
|  |  |  | All ages | 0 (0 to 0) | 0 (0 to 0) | 0 (0 to 0) | 141 (87.4 to 211.6) | 11.5 (5.7 to 20) | 129.5 (80.8 to 195.2) | NA | NA | NA |
|  |  | Deaths | Age-standardized | 0 (0 to 0) | 0 (0 to 0) | 0 (0 to 0) | 0 (0 to 0) | 0 (0 to 0) | 0 (0 to 0) | NA | NA | NA |
|  |  |  | All ages | 0 (0 to 0) | 0 (0 to 0) | 0 (0 to 0) | 3.2 (1.8 to 4.9) | 0.4 (0.2 to 0.8) | 2.8 (1.5 to 4.2) | NA | NA | NA |
|  | Diet high in sodium | DALYs (Disability-Adjusted Life Years) | Age-standardized | 0.4 (0 to 2.3) | 0.3 (0 to 2) | 0.5 (0 to 2.6) | 0.4 (0 to 2.4) | 0.3 (0 to 2.1) | 0.5 (0 to 2.7) | 0.7 (-86.9 to 356) | 0.2 (-99.5 to 2615.1) | 1 (-86.2 to 374.9) |
|  |  |  | All ages | 78 (0 to 443.5) | 25.8 (0 to 190) | 52.2 (0 to 263.7) | 264.2 (0 to 1594.9) | 91.1 (0 to 690.3) | 173.1 (0 to 940.5) | 238.6 (-55 to 1036.4) | 252.9 (-99.8 to 4560.2) | 231.5 (-57.4 to 1037.7) |
|  |  | Deaths | Age-standardized | 0 (0 to 0.1) | 0 (0 to 0.1) | 0 (0 to 0.1) | 0 (0 to 0.1) | 0 (0 to 0.1) | 0 (0 to 0.1) | -1.6 (-88.4 to 381.9) | 3.6 (-100 to 3113) | -6.9 (-88.7 to 425.1) |
|  |  |  | All ages | 2 (0 to 12.3) | 0.9 (0 to 6.9) | 1.1 (0 to 5.8) | 8.1 (0 to 56.5) | 3.6 (0 to 29.6) | 4.5 (0 to 27.8) | 308.9 (-60.8 to 1189.9) | 311.7 (-99.3 to 7053.5) | 306.8 (-61.7 to 1249.8) |
|  | High body-mass index | DALYs (Disability-Adjusted Life Years) | Age-standardized | 3.5 (1.4 to 6) | 5.4 (2.1 to 9.3) | 1.5 (0.6 to 2.9) | 10.5 (4.4 to 18.1) | 13.9 (5.9 to 23.9) | 7.4 (2.9 to 12.8) | 200.7 (80.4 to 354.7) | 157.8 (37.1 to 299.2) | 375.2 (198.8 to 904.3) |
|  |  |  | All ages | 706.5 (276.7 to 1173.3) | 523.9 (210.9 to 881.9) | 182.7 (69.5 to 329.9) | 7061.4 (2941.6 to 12080.8) | 4520.5 (1937.9 to 7735.9) | 2540.9 (1001.6 to 4465.5) | 899.4 (537 to 1280.2) | 762.9 (410.3 to 1114.4) | 1291 (800.1 to 2445.4) |
|  |  | Deaths | Age-standardized | 0.1 (0.1 to 0.3) | 0.2 (0.1 to 0.4) | 0 (0 to 0.1) | 0.5 (0.2 to 0.8) | 0.7 (0.3 to 1.2) | 0.3 (0.1 to 0.4) | 219.6 (66.3 to 494.4) | 198 (39.3 to 468.7) | 476.7 (198.5 to 1714) |
|  |  |  | All ages | 20.4 (8.1 to 35.2) | 17 (6.7 to 31.1) | 3.4 (1.1 to 6.5) | 273.6 (115.9 to 484.6) | 195.3 (81.9 to 340.9) | 78.3 (30.9 to 134.5) | 1241.6 (654.1 to 2101.8) | 1048.8 (479.9 to 1853.4) | 2208.2 (1226.6 to 5379.2) |
|  | High systolic blood pressure | DALYs (Disability-Adjusted Life Years) | Age-standardized | 19.6 (6.5 to 32.9) | 22.8 (7.7 to 38.5) | 15.9 (5 to 27.4) | 20.5 (6.8 to 34.5) | 24.5 (8.2 to 41.7) | 16.8 (5.6 to 29.4) | 4.7 (-12.1 to 23.1) | 7.7 (-14.5 to 29.8) | 5.2 (-8.9 to 28.8) |
|  |  |  | All ages | 3370.5 (1113.1 to 5682) | 1896.4 (641.9 to 3199.7) | 1474.1 (458.5 to 2552.3) | 13514.7 (4484.7 to 22734.4) | 7813.4 (2582.7 to 13294.2) | 5701.2 (1888.2 to 9843.1) | 301 (241.1 to 367.9) | 312 (229.8 to 391) | 286.8 (237.6 to 372.6) |
|  |  | Deaths | Age-standardized | 0.9 (0.3 to 1.6) | 1.2 (0.4 to 2.1) | 0.6 (0.2 to 1.1) | 0.9 (0.3 to 1.5) | 1.3 (0.5 to 2.2) | 0.6 (0.2 to 1) | -0.1 (-24.1 to 31.3) | 9.6 (-21.5 to 44.1) | -4.7 (-24.6 to 45.7) |
|  |  |  | All ages | 113.1 (38.1 to 193.5) | 76.3 (26.6 to 132.5) | 36.8 (10.9 to 64.8) | 537.5 (189.4 to 916.7) | 362.9 (126.4 to 617.3) | 174.6 (59.6 to 306.2) | 375.3 (260.9 to 520.2) | 375.5 (240.1 to 524.6) | 375.1 (268.3 to 616.1) |
|  | Lead exposure | DALYs (Disability-Adjusted Life Years) | Age-standardized | 3.8 (-0.5 to 9.8) | 4 (-0.6 to 10.4) | 3.6 (-0.5 to 8.9) | 4 (-0.6 to 10.1) | 4.3 (-0.6 to 10.8) | 3.8 (-0.5 to 9.4) | 5.2 (-11.5 to 26.2) | 7.1 (-16.6 to 32.9) | 6.1 (-8.6 to 32.9) |
|  |  |  | All ages | 674.1 (-90.6 to 1695.5) | 340.4 (-47.8 to 873.6) | 333.7 (-44.5 to 834.4) | 2604.4 (-350.4 to 6540) | 1330.8 (-180.3 to 3297.7) | 1273.6 (-170.1 to 3156.2) | 286.4 (226.7 to 362.4) | 291 (209.4 to 380) | 281.7 (229 to 374) |
|  |  | Deaths | Age-standardized | 0.2 (0 to 0.4) | 0.2 (0 to 0.5) | 0.1 (0 to 0.3) | 0.2 (0 to 0.5) | 0.3 (0 to 0.6) | 0.1 (0 to 0.3) | 8.8 (-16.3 to 43.7) | 18.8 (-15.7 to 58.9) | 3.8 (-17 to 63.7) |
|  |  |  | All ages | 22.1 (-3.4 to 55.4) | 13.6 (-2.2 to 34.9) | 8.4 (-1.3 to 21.5) | 111.9 (-16.7 to 273.9) | 68.2 (-10 to 166.1) | 43.7 (-7 to 107.2) | 406.6 (290.4 to 572.4) | 400 (254 to 561.7) | 417.4 (305.5 to 708.9) |
|  | Smoking | DALYs (Disability-Adjusted Life Years) | Age-standardized | 1.8 (1 to 2.8) | 0.6 (0.3 to 1) | 3 (1.7 to 4.6) | 1.9 (1.1 to 3) | 0.6 (0.3 to 1) | 3.2 (1.8 to 5.1) | 8.3 (-10.9 to 30.9) | 3.9 (-30.7 to 52.8) | 9.5 (-10.1 to 36.3) |
|  |  |  | All ages | 444 (253.9 to 692.2) | 72.6 (39.8 to 113.1) | 371.4 (210.7 to 580.6) | 1487.3 (822.5 to 2315.4) | 241.9 (126.5 to 399.2) | 1245.3 (688.9 to 1930.4) | 234.9 (178 to 298.5) | 233.3 (131.3 to 373.7) | 235.3 (176 to 312.8) |
|  |  | Deaths | Age-standardized | 0 (0 to 0.1) | 0 (0 to 0) | 0.1 (0 to 0.1) | 0 (0 to 0.1) | 0 (0 to 0) | 0.1 (0 to 0.1) | 7.1 (-23.7 to 63.5) | 6 (-44 to 97.8) | 1.5 (-29.4 to 64.6) |
|  |  |  | All ages | 7.9 (4.3 to 11.7) | 1.8 (0.9 to 2.8) | 6.1 (3.2 to 9.5) | 31.7 (17 to 49.7) | 6.8 (3.4 to 12.1) | 24.9 (13 to 39.6) | 301 (186.6 to 497.3) | 285 (121.4 to 564.9) | 305.5 (174.2 to 542.3) |
| Iraq | All risk factors | DALYs (Disability-Adjusted Life Years) | Age-standardized | 32.5 (16.9 to 47.6) | 31.3 (15.8 to 48.6) | 33.6 (18.5 to 49.8) | 42.1 (21.8 to 64.3) | 41.8 (21.1 to 64.8) | 41.9 (22.2 to 62.8) | 29.7 (2.2 to 76.9) | 33.3 (-1.9 to 88.6) | 24.6 (-1.3 to 83) |
|  |  |  | All ages | 2372.8 (1249 to 3469.7) | 1197.8 (607.1 to 1853.3) | 1175 (661.3 to 1751.4) | 7521.9 (3942.9 to 11353.2) | 3879.6 (1971.8 to 6056.8) | 3642.3 (1944.1 to 5343) | 217 (148.2 to 329.2) | 223.9 (139.3 to 361.6) | 210 (141.6 to 347.4) |
|  |  | Deaths | Age-standardized | 1.3 (0.7 to 2.1) | 1.4 (0.6 to 2.3) | 1.2 (0.6 to 2) | 2.1 (1 to 3.4) | 2.3 (1.1 to 3.7) | 1.8 (0.9 to 2.9) | 59.5 (11.7 to 152.3) | 64.9 (6.1 to 167.9) | 47.8 (0.2 to 176.7) |
|  |  |  | All ages | 92.3 (46.7 to 145) | 52.7 (24.5 to 89.6) | 39.6 (19.3 to 62.6) | 287.5 (147 to 450.1) | 174.2 (85.5 to 276.3) | 113.4 (58.1 to 179.4) | 211.6 (115 to 396.2) | 230.3 (112.4 to 439.5) | 186.6 (94.9 to 437.1) |
|  | Alcohol use | DALYs (Disability-Adjusted Life Years) | Age-standardized | 0.1 (0.1 to 0.2) | 0.1 (0.1 to 0.2) | 0.2 (0.1 to 0.2) | 0.1 (0.1 to 0.1) | 0.1 (0.1 to 0.1) | 0.1 (0.1 to 0.2) | -20.7 (-40.6 to 14.9) | -22.1 (-50.7 to 23.5) | -19.5 (-41.9 to 19.7) |
|  |  |  | All ages | 10 (6.3 to 14) | 4.3 (2.6 to 6.4) | 5.7 (3.5 to 8.1) | 21.6 (14.6 to 29.8) | 8.9 (5.7 to 12.8) | 12.7 (8.4 to 17.8) | 116.2 (61 to 199.5) | 106.3 (32.4 to 230.9) | 123.8 (64.9 to 228) |
|  |  | Deaths | Age-standardized | 0 (0 to 0) | 0 (0 to 0) | 0 (0 to 0) | 0 (0 to 0) | 0 (0 to 0) | 0 (0 to 0) | -6.1 (-40.1 to 65.1) | -5.3 (-50.1 to 84.8) | -7.2 (-42.3 to 83.6) |
|  |  |  | All ages | 0.3 (0.2 to 0.5) | 0.2 (0.1 to 0.3) | 0.2 (0.1 to 0.2) | 0.6 (0.4 to 1) | 0.3 (0.2 to 0.5) | 0.3 (0.2 to 0.5) | 102.2 (29.5 to 243.8) | 103.8 (11.6 to 289.4) | 100.6 (27 to 292.2) |
|  | Diet high in sodium | DALYs (Disability-Adjusted Life Years) | Age-standardized | 0.4 (0 to 2.5) | 0.3 (0 to 1.9) | 0.6 (0 to 3.2) | 0.5 (0 to 2.9) | 0.3 (0 to 2.3) | 0.7 (0 to 3.8) | 11 (-91.1 to 659.7) | 16.2 (-890.4 to 3420.4) | 10.4 (-91.1 to 657.4) |
|  |  |  | All ages | 32.4 (0 to 189.1) | 10.3 (0 to 73.2) | 22 (0 to 114.1) | 96.5 (0 to 554.6) | 31.4 (0 to 225.9) | 65 (0 to 358.1) | 198 (-69.1 to 1976.9) | 204.2 (-2173.9 to 5752.7) | 195.1 (-69.5 to 1986.2) |
|  |  | Deaths | Age-standardized | 0 (0 to 0.1) | 0 (0 to 0.1) | 0 (0 to 0.1) | 0 (0 to 0.1) | 0 (0 to 0.1) | 0 (0 to 0.2) | 31.4 (-93.7 to 654.6) | 40 (-952.5 to 2702.6) | 30 (-93.8 to 687.7) |
|  |  |  | All ages | 1.1 (0 to 6.4) | 0.4 (0 to 3.1) | 0.7 (0 to 3.7) | 3 (0 to 19.3) | 1.2 (0 to 9.5) | 1.8 (0 to 10.3) | 187.8 (-77.3 to 2073.1) | 207.5 (-1969.5 to 7353.9) | 176.2 (-77.5 to 2076.2) |
|  | High body-mass index | DALYs (Disability-Adjusted Life Years) | Age-standardized | 9.3 (3.4 to 18.5) | 10.2 (3.9 to 19.9) | 8.2 (2.7 to 17.6) | 14.5 (6.1 to 26.3) | 15.3 (6.5 to 28.8) | 13.3 (5.6 to 24.1) | 56.4 (15 to 130.1) | 49.5 (9.1 to 122.6) | 62.6 (15.6 to 174.2) |
|  |  |  | All ages | 683.8 (251.6 to 1356.1) | 394.5 (150.8 to 767.4) | 289.3 (94.5 to 609.4) | 2602.4 (1105.5 to 4655.4) | 1439.7 (614.9 to 2689.1) | 1162.8 (495.6 to 2061.3) | 280.6 (182.8 to 454.5) | 264.9 (164.9 to 439.5) | 301.9 (185.5 to 558.7) |
|  |  | Deaths | Age-standardized | 0.4 (0.1 to 0.8) | 0.4 (0.2 to 0.9) | 0.3 (0.1 to 0.7) | 0.7 (0.3 to 1.4) | 0.8 (0.3 to 1.5) | 0.6 (0.2 to 1.1) | 95.7 (24.3 to 249) | 89.4 (19.5 to 238.1) | 97.3 (17.9 to 354.5) |
|  |  |  | All ages | 26.2 (9.2 to 55.2) | 16.7 (5.9 to 35.9) | 9.5 (2.8 to 21.1) | 99.2 (41.1 to 182.6) | 63.2 (25.5 to 117) | 36 (15.5 to 66.8) | 278.1 (143 to 562) | 278.2 (140.5 to 575.4) | 277.8 (130.7 to 731.4) |
|  | High systolic blood pressure | DALYs (Disability-Adjusted Life Years) | Age-standardized | 24.7 (9.1 to 41.3) | 25.3 (9 to 43.7) | 24.1 (8.3 to 41.3) | 32.6 (11 to 54.9) | 33.6 (11.1 to 57.3) | 30.9 (10.1 to 53) | 31.7 (1 to 80.1) | 32.8 (-5.8 to 95.8) | 28.3 (-1.3 to 90.5) |
|  |  |  | All ages | 1788.4 (660.2 to 2977.1) | 962.9 (342.9 to 1661.9) | 825.5 (276.9 to 1419.4) | 5704.5 (1896.6 to 9756.1) | 3099.3 (999.8 to 5298) | 2605.2 (848 to 4372) | 219 (143.4 to 337.5) | 221.9 (127.3 to 371.5) | 215.6 (139.8 to 358.9) |
|  |  | Deaths | Age-standardized | 1 (0.4 to 1.8) | 1.1 (0.4 to 2) | 0.9 (0.3 to 1.6) | 1.7 (0.6 to 2.9) | 1.9 (0.7 to 3.2) | 1.4 (0.4 to 2.5) | 60.5 (7.6 to 155.8) | 63 (0.3 to 178.1) | 51 (-1.2 to 193.3) |
|  |  |  | All ages | 71.8 (25 to 122.9) | 43 (14.8 to 77.1) | 28.7 (9.4 to 50.8) | 224.3 (78.4 to 389.3) | 140.6 (50.5 to 238.3) | 83.7 (28.2 to 150.2) | 212.4 (110.7 to 407) | 226.6 (102.9 to 453.4) | 191.2 (93.2 to 462) |
|  | Lead exposure | DALYs (Disability-Adjusted Life Years) | Age-standardized | 2.5 (-0.3 to 6.3) | 2.1 (-0.3 to 5.5) | 2.9 (-0.4 to 7.6) | 3 (-0.4 to 7.5) | 2.8 (-0.4 to 7.2) | 3.3 (-0.4 to 8.4) | 23.1 (-3.7 to 69.9) | 33.1 (-3 to 91.3) | 15 (-11.9 to 71.2) |
|  |  |  | All ages | 178.9 (-23.9 to 463.2) | 80.1 (-10.8 to 209) | 98.8 (-13 to 260.3) | 502.4 (-65.5 to 1260.1) | 244.9 (-33.5 to 635.3) | 257.4 (-32.4 to 649.7) | 180.9 (119.4 to 285.1) | 205.8 (120.8 to 344) | 160.6 (100.7 to 285) |
|  |  | Deaths | Age-standardized | 0.1 (0 to 0.3) | 0.1 (0 to 0.2) | 0.1 (0 to 0.3) | 0.2 (0 to 0.4) | 0.2 (0 to 0.4) | 0.2 (0 to 0.4) | 60.4 (9.6 to 161.7) | 74.4 (12.9 to 183.9) | 44.2 (-4.8 to 178.3) |
|  |  |  | All ages | 7.1 (-1 to 18.5) | 3.6 (-0.5 to 9.2) | 3.5 (-0.5 to 9.4) | 21.1 (-3 to 54.6) | 12 (-1.7 to 31.1) | 9 (-1.2 to 24.2) | 196.1 (105.5 to 382.5) | 233.7 (117 to 440.8) | 157.4 (69.7 to 382.4) |
|  | Smoking | DALYs (Disability-Adjusted Life Years) | Age-standardized | 5 (2.9 to 7.6) | 1.6 (0.9 to 2.6) | 8.7 (4.9 to 13.2) | 4.5 (2.5 to 6.9) | 1.4 (0.8 to 2.4) | 8 (4.6 to 12.5) | -10.5 (-31.1 to 27.7) | -11.8 (-43.9 to 40.8) | -7.9 (-29.2 to 37) |
|  |  |  | All ages | 388.8 (222.4 to 588.7) | 64.4 (34.7 to 103.2) | 324.4 (181.9 to 484.4) | 968.7 (547.8 to 1464.2) | 149.4 (80 to 243.8) | 819.3 (472.6 to 1240.1) | 149.2 (88 to 255.4) | 132 (45 to 262.8) | 152.6 (88.8 to 267.3) |
|  |  | Deaths | Age-standardized | 0.2 (0.1 to 0.3) | 0.1 (0 to 0.1) | 0.3 (0.1 to 0.5) | 0.2 (0.1 to 0.3) | 0.1 (0 to 0.1) | 0.3 (0.2 to 0.5) | 3.2 (-30.9 to 84.9) | 12.4 (-42.2 to 129.5) | 6.6 (-30.4 to 105.9) |
|  |  |  | All ages | 11.8 (6.6 to 18.4) | 2.3 (1.1 to 4.2) | 9.5 (4.9 to 15.2) | 27 (14.9 to 43.9) | 5.5 (2.8 to 9.8) | 21.5 (11.7 to 34.6) | 129 (52.1 to 315.1) | 141.9 (30.7 to 383.9) | 125.9 (48.6 to 328.4) |
| Jordan | All risk factors | DALYs (Disability-Adjusted Life Years) | Age-standardized | 27.3 (14.9 to 40.1) | 31.1 (15.5 to 47.2) | 23.7 (12.7 to 35.4) | 28.3 (16.1 to 41.3) | 32.2 (17.9 to 48) | 25.5 (14.3 to 37.3) | 3.4 (-16.8 to 27.4) | 3.4 (-23.7 to 36.6) | 7.8 (-15.7 to 35) |
|  |  |  | All ages | 268.7 (150.8 to 393.8) | 146 (75.7 to 218) | 122.8 (67.8 to 182.4) | 1561.5 (913.4 to 2290.1) | 766.4 (430.7 to 1140.3) | 795.1 (461.9 to 1179) | 481.1 (372.8 to 612.5) | 425.1 (294.7 to 589.8) | 547.7 (402.3 to 708.3) |
|  |  | Deaths | Age-standardized | 1.2 (0.6 to 1.8) | 1.6 (0.8 to 2.5) | 0.9 (0.5 to 1.3) | 1.2 (0.7 to 1.8) | 1.7 (0.9 to 2.6) | 0.8 (0.5 to 1.2) | -2.7 (-29.5 to 30.5) | 8.3 (-27.7 to 55.4) | -6.7 (-36 to 39.6) |
|  |  |  | All ages | 9.2 (4.9 to 13.9) | 5.8 (2.9 to 9.1) | 3.4 (1.8 to 5.2) | 45.3 (26.6 to 67.6) | 27.8 (15.5 to 42.4) | 17.5 (10.5 to 25.9) | 393.9 (257.9 to 560.9) | 378.8 (216.1 to 592.6) | 419.9 (254.1 to 667) |
|  | Alcohol use | DALYs (Disability-Adjusted Life Years) | Age-standardized | 0.1 (0.1 to 0.1) | 0 (0 to 0) | 0.2 (0.1 to 0.3) | 0.1 (0.1 to 0.1) | 0 (0 to 0) | 0.2 (0.1 to 0.3) | 4.5 (-24.2 to 48.2) | -15.2 (-51.2 to 48.4) | 1 (-27.3 to 42.6) |
|  |  |  | All ages | 1.1 (0.6 to 1.6) | 0.1 (0 to 0.1) | 1 (0.6 to 1.5) | 6.5 (4 to 9.4) | 0.3 (0.1 to 0.4) | 6.2 (3.8 to 9.1) | 498.8 (344.4 to 726.5) | 339.9 (159.4 to 635.5) | 508.6 (345.9 to 742.1) |
|  |  | Deaths | Age-standardized | 0 (0 to 0) | 0 (0 to 0) | 0 (0 to 0) | 0 (0 to 0) | 0 (0 to 0) | 0 (0 to 0) | -9.4 (-43.8 to 54.9) | -13.7 (-62.2 to 85.4) | -15.6 (-48.9 to 45.9) |
|  |  |  | All ages | 0 (0 to 0) | 0 (0 to 0) | 0 (0 to 0) | 0.1 (0.1 to 0.2) | 0 (0 to 0) | 0.1 (0.1 to 0.2) | 367.6 (198.6 to 667) | 281.2 (88.9 to 628.9) | 374.7 (197.7 to 695.8) |
|  | Diet high in sodium | DALYs (Disability-Adjusted Life Years) | Age-standardized | 0.4 (0 to 2.3) | 0.3 (0 to 1.9) | 0.5 (0 to 2.7) | 0.3 (0 to 2.1) | 0.2 (0 to 1.7) | 0.5 (0 to 2.5) | -7.9 (-90.1 to 538.7) | -12.8 (-853673.7 to 1397.7) | -7.4 (-91 to 513.4) |
|  |  |  | All ages | 3.9 (0 to 23.3) | 1.3 (0 to 9.4) | 2.6 (0 to 14.6) | 20.7 (0 to 124.4) | 6 (0 to 43.5) | 14.7 (0 to 84.6) | 428.8 (-23.7 to 3075.8) | 359.7 (-10712926.8 to 7356.6) | 463.2 (-23.7 to 3141.9) |
|  |  | Deaths | Age-standardized | 0 (0 to 0.1) | 0 (0 to 0.1) | 0 (0 to 0.1) | 0 (0 to 0.1) | 0 (0 to 0.1) | 0 (0 to 0.1) | -17 (-93.3 to 563.5) | -12.1 (-171120.5 to 1488.6) | -20.5 (-93.8 to 573.7) |
|  |  |  | All ages | 0.1 (0 to 0.7) | 0 (0 to 0.4) | 0.1 (0 to 0.4) | 0.5 (0 to 3.1) | 0.2 (0 to 1.5) | 0.3 (0 to 1.8) | 333.5 (-43.8 to 2504.8) | 307.5 (-1368351 to 6321) | 352 (-44.4 to 2522.2) |
|  | High body-mass index | DALYs (Disability-Adjusted Life Years) | Age-standardized | 8.2 (3.3 to 14.4) | 11.2 (4.6 to 19.7) | 5.3 (1.9 to 9.6) | 13.6 (6.1 to 23.2) | 17.9 (8.1 to 29.6) | 10.2 (4.3 to 18.1) | 66.3 (29.8 to 108) | 60.2 (21.9 to 112.3) | 94.6 (33.2 to 189.3) |
|  |  |  | All ages | 85.6 (35 to 150.7) | 56.9 (23.4 to 98) | 28.7 (10.6 to 53.7) | 765.6 (344.7 to 1307.1) | 443.1 (203.2 to 741.4) | 322.5 (138.9 to 560.7) | 794.3 (624.4 to 1014.3) | 678.3 (505.7 to 904) | 1024.5 (720 to 1452) |
|  |  | Deaths | Age-standardized | 0.3 (0.1 to 0.6) | 0.5 (0.2 to 0.9) | 0.2 (0.1 to 0.4) | 0.6 (0.2 to 1) | 0.9 (0.4 to 1.6) | 0.3 (0.1 to 0.6) | 66 (15.8 to 137.8) | 80 (15.1 to 173.8) | 75.1 (0.3 to 231.2) |
|  |  |  | All ages | 2.7 (1.1 to 4.9) | 2 (0.8 to 3.5) | 0.7 (0.3 to 1.5) | 22.1 (9.7 to 38.8) | 15.1 (6.5 to 26.8) | 7 (3 to 12.8) | 707.8 (467.6 to 1022) | 655.3 (401.9 to 990.2) | 850.7 (462.8 to 1575.9) |
|  | High systolic blood pressure | DALYs (Disability-Adjusted Life Years) | Age-standardized | 20.3 (7.1 to 34.1) | 24.4 (8.2 to 41.5) | 16.4 (5.6 to 28.2) | 18.8 (6 to 32.5) | 21.9 (7.2 to 37.9) | 16.6 (5 to 28.8) | -7.6 (-27.3 to 13.2) | -10.5 (-36.5 to 21.4) | 1.2 (-23.7 to 32.7) |
|  |  |  | All ages | 191.3 (64.6 to 320) | 111.1 (37.4 to 189.9) | 80.2 (26.4 to 138.9) | 997.8 (318.9 to 1748.8) | 507.2 (167.4 to 891.3) | 490.6 (154.5 to 866.5) | 421.6 (313.2 to 542.2) | 356.4 (227.3 to 528.5) | 512 (357.5 to 691.4) |
|  |  | Deaths | Age-standardized | 0.9 (0.3 to 1.6) | 1.3 (0.4 to 2.2) | 0.6 (0.2 to 1.1) | 0.8 (0.3 to 1.4) | 1.2 (0.4 to 2.1) | 0.5 (0.2 to 0.9) | -13.7 (-39.8 to 14.9) | -6.8 (-40.8 to 38) | -12.9 (-42.6 to 31.8) |
|  |  |  | All ages | 7 (2.4 to 11.8) | 4.6 (1.6 to 8.1) | 2.4 (0.8 to 4.2) | 30.7 (10.8 to 53.9) | 19.2 (6.3 to 34.1) | 11.5 (3.8 to 20) | 340.8 (206.2 to 484.9) | 316.3 (162.6 to 504.4) | 388.5 (222.6 to 630.8) |
|  | Lead exposure | DALYs (Disability-Adjusted Life Years) | Age-standardized | 2 (-0.3 to 5.3) | 1.8 (-0.2 to 4.7) | 2.3 (-0.3 to 5.7) | 1.9 (-0.3 to 4.9) | 1.8 (-0.2 to 4.6) | 2.1 (-0.3 to 5.5) | -4.8 (-23.1 to 14.3) | -2.1 (-30.5 to 27.2) | -5.8 (-23.5 to 16.5) |
|  |  |  | All ages | 19.6 (-2.7 to 51.3) | 8.4 (-1.1 to 21.5) | 11.2 (-1.5 to 28.6) | 96.6 (-12.8 to 252.3) | 38.4 (-5.2 to 98) | 58.2 (-7.6 to 150.2) | 393.1 (300.9 to 483.7) | 359.5 (231.1 to 485.3) | 418 (323.8 to 529.4) |
|  |  | Deaths | Age-standardized | 0.1 (0 to 0.2) | 0.1 (0 to 0.2) | 0.1 (0 to 0.2) | 0.1 (0 to 0.2) | 0.1 (0 to 0.3) | 0.1 (0 to 0.2) | -2.7 (-29.6 to 27.8) | 10.1 (-30.5 to 53.7) | -9.2 (-35.9 to 30.3) |
|  |  |  | All ages | 0.7 (-0.1 to 1.8) | 0.3 (0 to 0.8) | 0.3 (0 to 0.9) | 3.1 (-0.5 to 7.7) | 1.5 (-0.2 to 4) | 1.6 (-0.2 to 3.9) | 369.1 (231.2 to 510.5) | 358.4 (187.8 to 555) | 380.2 (233.8 to 600.6) |
|  | Smoking | DALYs (Disability-Adjusted Life Years) | Age-standardized | 4.2 (2.5 to 6.5) | 1.9 (1 to 3.1) | 6.5 (3.7 to 9.9) | 3.8 (2.2 to 5.8) | 1.6 (0.9 to 2.7) | 5.7 (3.3 to 8.8) | -9.8 (-27.4 to 11.4) | -11.9 (-43.8 to 35.2) | -12.1 (-30.5 to 8.6) |
|  |  |  | All ages | 48.8 (29.1 to 75.5) | 9.9 (5.4 to 15.9) | 38.9 (22.7 to 60.3) | 256.6 (145.4 to 389.4) | 43.8 (23.9 to 70.2) | 212.9 (118.9 to 324.7) | 426 (319.1 to 550.9) | 342.2 (202.8 to 532.8) | 447.3 (331 to 574.1) |
|  |  | Deaths | Age-standardized | 0.1 (0.1 to 0.2) | 0.1 (0 to 0.2) | 0.2 (0.1 to 0.3) | 0.1 (0.1 to 0.2) | 0.1 (0 to 0.1) | 0.1 (0.1 to 0.2) | -21.6 (-46.1 to 14.6) | -8.3 (-56.2 to 69.7) | -28.5 (-52.4 to 7.7) |
|  |  |  | All ages | 1.2 (0.7 to 1.8) | 0.3 (0.2 to 0.6) | 0.9 (0.5 to 1.3) | 4.9 (2.8 to 7.5) | 1.4 (0.7 to 2.3) | 3.5 (2 to 5.5) | 306.6 (184.7 to 481.5) | 301.1 (113.7 to 577) | 308.8 (172 to 511) |
| Kuwait | All risk factors | DALYs (Disability-Adjusted Life Years) | Age-standardized | 24.8 (12.5 to 37.5) | 26.4 (12.5 to 40.8) | 23.6 (11.9 to 36.1) | 30.4 (16.9 to 44.2) | 28.1 (15.2 to 41.2) | 32 (18.1 to 47.4) | 22.2 (2.6 to 54.8) | 6.3 (-12.9 to 38.2) | 35.5 (11.7 to 82.4) |
|  |  |  | All ages | 108.8 (55.3 to 163.3) | 48.2 (23.5 to 74.1) | 60.7 (32.8 to 93.5) | 694.9 (389.3 to 1017.3) | 263 (144.9 to 386.6) | 431.9 (248.2 to 638.5) | 538.5 (447.3 to 686.5) | 446.2 (349.5 to 619.4) | 611.8 (489.8 to 798.2) |
|  |  | Deaths | Age-standardized | 1 (0.5 to 1.6) | 1.2 (0.5 to 1.9) | 0.8 (0.4 to 1.3) | 1.4 (0.8 to 2) | 1.3 (0.7 to 1.9) | 1.4 (0.8 to 2) | 35.3 (6.6 to 85) | 12.6 (-15.3 to 58.2) | 63.3 (22.3 to 131.2) |
|  |  |  | All ages | 3.3 (1.7 to 5.1) | 2 (0.9 to 3.2) | 1.3 (0.7 to 2) | 27.2 (15.2 to 39.6) | 11.5 (6.1 to 16.8) | 15.7 (8.7 to 23.1) | 714.9 (547.8 to 979.4) | 474.4 (329.1 to 711.9) | 1077.8 (798.4 to 1460.7) |
|  | Alcohol use | DALYs (Disability-Adjusted Life Years) | Age-standardized | 0 (0 to 0) | 0 (0 to 0) | 0 (0 to 0) | 0 (0 to 0) | 0 (0 to 0) | 0 (0 to 0.1) | NA | NA | NA |
|  |  |  | All ages | 0 (0 to 0) | 0 (0 to 0) | 0 (0 to 0) | 0.6 (0 to 1.5) | 0 (0 to 0) | 0.5 (0 to 1.4) | NA | NA | NA |
|  |  | Deaths | Age-standardized | 0 (0 to 0) | 0 (0 to 0) | 0 (0 to 0) | 0 (0 to 0) | 0 (0 to 0) | 0 (0 to 0) | NA | NA | NA |
|  |  |  | All ages | 0 (0 to 0) | 0 (0 to 0) | 0 (0 to 0) | 0 (0 to 0) | 0 (0 to 0) | 0 (0 to 0) | NA | NA | NA |
|  | Diet high in sodium | DALYs (Disability-Adjusted Life Years) | Age-standardized | 0.8 (0 to 3.7) | 0.5 (0 to 2.9) | 1.1 (0 to 4.7) | 0.9 (0 to 4.3) | 0.5 (0 to 2.8) | 1.2 (0 to 5.2) | 12.2 (-73 to 187.6) | -12.1 (-97.6 to 682.7) | 14.7 (-69.5 to 207.7) |
|  |  |  | All ages | 3.8 (0 to 16.6) | 1 (0 to 5.4) | 2.9 (0 to 11.7) | 21.1 (0 to 96.5) | 4.3 (0 to 26) | 16.8 (0 to 70.1) | 454.5 (48.5 to 1072.2) | 349.1 (-74.5 to 3562) | 489.7 (47.7 to 1075.8) |
|  |  | Deaths | Age-standardized | 0 (0 to 0.1) | 0 (0 to 0.1) | 0 (0 to 0.2) | 0 (0 to 0.2) | 0 (0 to 0.1) | 0 (0 to 0.2) | 28.8 (-78.9 to 308.3) | -12.4 (-98.9 to 1213.7) | 31.4 (-79.9 to 314) |
|  |  |  | All ages | 0.1 (0 to 0.5) | 0 (0 to 0.2) | 0.1 (0 to 0.3) | 0.7 (0 to 3.4) | 0.2 (0 to 1) | 0.5 (0 to 2.4) | 622 (20.9 to 1303.3) | 336.6 (-86.3 to 2956.7) | 790.3 (20.5 to 1398.4) |
|  | High body-mass index | DALYs (Disability-Adjusted Life Years) | Age-standardized | 6 (2.3 to 11.4) | 8 (3.2 to 14.9) | 4.6 (1.7 to 9.1) | 15.4 (7 to 25.8) | 15.4 (7.1 to 25.1) | 15.5 (6.6 to 27.1) | 158.9 (94 to 250.1) | 93.2 (47.6 to 173.7) | 233.4 (119.6 to 406.4) |
|  |  |  | All ages | 30.2 (12.3 to 55.8) | 15.9 (6.3 to 29.5) | 14.3 (5.6 to 26.9) | 368.3 (172.2 to 625.3) | 153 (71.8 to 251.2) | 215.3 (96.3 to 378.9) | 1120 (853.2 to 1482.1) | 865.4 (649.6 to 1221.9) | 1401.4 (1004.5 to 1922.7) |
|  |  | Deaths | Age-standardized | 0.2 (0.1 to 0.4) | 0.3 (0.1 to 0.6) | 0.2 (0.1 to 0.3) | 0.7 (0.3 to 1.1) | 0.7 (0.3 to 1.1) | 0.7 (0.3 to 1.2) | 208.2 (108.4 to 375.4) | 127.5 (53 to 268.7) | 328 (138.5 to 709.6) |
|  |  |  | All ages | 0.8 (0.3 to 1.5) | 0.5 (0.2 to 1) | 0.3 (0.1 to 0.5) | 13.3 (5.9 to 23) | 5.8 (2.5 to 10.1) | 7.5 (3.1 to 13.4) | 1602.6 (1083.7 to 2379.8) | 1028.2 (661.5 to 1681.4) | 2712.5 (1660 to 4392) |
|  | High systolic blood pressure | DALYs (Disability-Adjusted Life Years) | Age-standardized | 20.1 (6.7 to 33.7) | 22 (7.5 to 36.4) | 18.5 (5.9 to 31.9) | 20.8 (6.7 to 34.6) | 19.8 (6.3 to 33) | 21.4 (7 to 36.2) | 3.4 (-13.6 to 21.4) | -9.7 (-28.3 to 16.7) | 15.8 (-4.4 to 44.5) |
|  |  |  | All ages | 81.7 (26.3 to 140.1) | 38.9 (13 to 65.3) | 42.8 (13 to 75.7) | 451.6 (144.4 to 755.9) | 177.6 (56.1 to 292.9) | 274 (88.9 to 474.7) | 453 (369.4 to 540.7) | 356.6 (260 to 480) | 540.7 (428.3 to 687.3) |
|  |  | Deaths | Age-standardized | 0.9 (0.3 to 1.4) | 1 (0.3 to 1.7) | 0.7 (0.2 to 1.2) | 1 (0.3 to 1.6) | 1 (0.3 to 1.6) | 1 (0.3 to 1.6) | 14.3 (-11.2 to 43.6) | -3 (-30.1 to 30.7) | 39.5 (4.7 to 86.3) |
|  |  |  | All ages | 2.8 (1 to 4.6) | 1.7 (0.6 to 2.9) | 1 (0.4 to 1.7) | 19.3 (6.6 to 32) | 8.5 (2.8 to 13.9) | 10.8 (3.6 to 18.6) | 602.4 (453.1 to 776.6) | 395.5 (252.7 to 575.9) | 945.8 (697.5 to 1247.4) |
|  | Lead exposure | DALYs (Disability-Adjusted Life Years) | Age-standardized | 1.6 (-0.2 to 4) | 1.4 (-0.2 to 3.6) | 1.7 (-0.2 to 4.4) | 1.6 (-0.2 to 4.2) | 1.2 (-0.2 to 3.1) | 1.9 (-0.3 to 4.9) | 3.6 (-9.4 to 19) | -14.7 (-33 to 5.8) | 11.5 (-6.3 to 32) |
|  |  |  | All ages | 6.9 (-0.9 to 17.9) | 2.7 (-0.4 to 6.8) | 4.3 (-0.5 to 11.1) | 34.3 (-4.6 to 86.9) | 10.9 (-1.5 to 27.6) | 23.4 (-3.1 to 61) | 397 (338.4 to 465.3) | 311.8 (226.4 to 406.2) | 450.1 (372.5 to 548.6) |
|  |  | Deaths | Age-standardized | 0.1 (0 to 0.2) | 0.1 (0 to 0.2) | 0.1 (0 to 0.2) | 0.1 (0 to 0.2) | 0.1 (0 to 0.2) | 0.1 (0 to 0.2) | 25.1 (1.9 to 54.1) | -4.2 (-30.4 to 24.6) | 42.3 (10.1 to 78.6) |
|  |  |  | All ages | 0.2 (0 to 0.5) | 0.1 (0 to 0.3) | 0.1 (0 to 0.2) | 1.5 (-0.2 to 3.9) | 0.5 (-0.1 to 1.4) | 1 (-0.1 to 2.5) | 641.3 (507.7 to 798.3) | 386.9 (255.8 to 531) | 929.2 (695.9 to 1183.8) |
|  | Smoking | DALYs (Disability-Adjusted Life Years) | Age-standardized | 2.8 (1.6 to 4.3) | 0.9 (0.5 to 1.5) | 4.3 (2.4 to 6.5) | 2.7 (1.5 to 4.1) | 0.6 (0.3 to 1) | 4.2 (2.4 to 6.5) | -5.3 (-21.6 to 14.8) | -37.6 (-63.1 to 8.5) | -1.6 (-18.1 to 21.2) |
|  |  |  | All ages | 17.3 (9.8 to 26.9) | 1.9 (1 to 3.1) | 15.4 (8.5 to 24) | 77.6 (43.6 to 120.5) | 6.6 (3.3 to 10.7) | 71 (40.5 to 110.1) | 348.4 (277 to 444.7) | 245.3 (117.9 to 458) | 361.1 (285.3 to 470.7) |
|  |  | Deaths | Age-standardized | 0.1 (0 to 0.1) | 0 (0 to 0.1) | 0.1 (0.1 to 0.2) | 0.1 (0 to 0.1) | 0 (0 to 0) | 0.1 (0.1 to 0.2) | 17.1 (-16.8 to 65.6) | -34.4 (-72.1 to 48.2) | 14.8 (-17.4 to 65.1) |
|  |  |  | All ages | 0.3 (0.2 to 0.4) | 0.1 (0 to 0.1) | 0.2 (0.1 to 0.3) | 1.9 (1 to 2.9) | 0.2 (0.1 to 0.4) | 1.7 (0.9 to 2.6) | 507.8 (343.7 to 720.5) | 233.5 (53.3 to 596.6) | 582.2 (404 to 840.6) |
| Lebanon | All risk factors | DALYs (Disability-Adjusted Life Years) | Age-standardized | 33.7 (16 to 54.3) | 35.1 (15.6 to 60.7) | 31.8 (14.1 to 55.9) | 28.7 (15.5 to 41.2) | 27.5 (15 to 40.8) | 30 (16.2 to 44) | -14.8 (-40.9 to 33.6) | -21.5 (-52.9 to 27.8) | -5.6 (-39.2 to 56.6) |
|  |  |  | All ages | 602.7 (291.7 to 951) | 332.6 (154.3 to 579.2) | 270.1 (127 to 467.3) | 1868.8 (1005.5 to 2704.4) | 982 (533.7 to 1459.1) | 886.9 (475.9 to 1298.7) | 210.1 (114.6 to 372.8) | 195.2 (79.4 to 380.1) | 228.4 (115.5 to 452.9) |
|  |  | Deaths | Age-standardized | 1.7 (0.7 to 2.9) | 1.9 (0.7 to 3.6) | 1.4 (0.5 to 2.9) | 1.3 (0.7 to 1.9) | 1.4 (0.7 to 2.2) | 1.2 (0.7 to 1.9) | -22.5 (-51.6 to 54.7) | -26.2 (-60.4 to 51.4) | -15.6 (-55.8 to 108.6) |
|  |  |  | All ages | 25.5 (10.7 to 44.2) | 15.9 (6.5 to 30.4) | 9.6 (3.3 to 19.2) | 91.1 (48.5 to 134.1) | 53.5 (28.5 to 83.5) | 37.6 (20.2 to 59.1) | 256.5 (118.1 to 599.2) | 235.4 (82.4 to 578.3) | 291.6 (106.5 to 860.6) |
|  | Alcohol use | DALYs (Disability-Adjusted Life Years) | Age-standardized | 1 (0.5 to 1.6) | 0.1 (0.1 to 0.3) | 2.1 (1.1 to 3.4) | 0.5 (0.3 to 0.8) | 0.1 (0 to 0.1) | 1.1 (0.6 to 1.6) | -50.8 (-70.1 to -14.7) | -59 (-81.2 to -15.8) | -49.6 (-70 to -9.8) |
|  |  |  | All ages | 20.7 (11.1 to 32.7) | 1.4 (0.6 to 2.7) | 19.3 (10.2 to 30.6) | 32 (19.2 to 47.6) | 1.8 (0.7 to 3.4) | 30.1 (18.2 to 44.9) | 54.4 (-6 to 164.4) | 32.9 (-40.1 to 172.5) | 56 (-5.5 to 171.9) |
|  |  | Deaths | Age-standardized | 0 (0 to 0.1) | 0 (0 to 0) | 0.1 (0 to 0.2) | 0 (0 to 0) | 0 (0 to 0) | 0 (0 to 0.1) | -56.5 (-79.1 to 6.2) | -62.9 (-87.5 to 3.6) | -57.3 (-80.2 to 10.2) |
|  |  |  | All ages | 0.6 (0.3 to 1.2) | 0.1 (0 to 0.1) | 0.6 (0.2 to 1.1) | 1.1 (0.6 to 1.8) | 0.1 (0 to 0.2) | 1.1 (0.6 to 1.7) | 77.6 (-14.5 to 326) | 51.1 (-51.4 to 310) | 79.9 (-17 to 345.6) |
|  | Diet high in sodium | DALYs (Disability-Adjusted Life Years) | Age-standardized | 0.5 (0 to 3) | 0.3 (0 to 2.5) | 0.7 (0 to 3.7) | 0.4 (0 to 2.2) | 0.2 (0 to 1.7) | 0.5 (0 to 2.9) | -26.9 (-95.2 to 393.3) | -34.5 (-581.8 to 3097.4) | -22.2 (-95.3 to 415.6) |
|  |  |  | All ages | 9.2 (0 to 54.8) | 3.3 (0 to 24.4) | 5.9 (0 to 31.4) | 22.7 (0 to 143.7) | 7.6 (0 to 60.1) | 15.1 (0 to 83.8) | 146.5 (-87.4 to 1223.5) | 131 (-3446.7 to 10423.9) | 155.1 (-87.9 to 1266.8) |
|  |  | Deaths | Age-standardized | 0 (0 to 0.1) | 0 (0 to 0.1) | 0 (0 to 0.2) | 0 (0 to 0.1) | 0 (0 to 0.1) | 0 (0 to 0.1) | -35.8 (-98.1 to 293.7) | -40.9 (-860.6 to 2509.7) | -33.2 (-98.1 to 331.8) |
|  |  |  | All ages | 0.3 (0 to 2.2) | 0.1 (0 to 1.1) | 0.2 (0 to 1.1) | 0.9 (0 to 6.4) | 0.4 (0 to 3) | 0.6 (0 to 3.6) | 171.4 (-94.9 to 991.5) | 150.1 (-1524.8 to 9546.8) | 186.7 (-94.9 to 1037.9) |
|  | High body-mass index | DALYs (Disability-Adjusted Life Years) | Age-standardized | 8.1 (3 to 15.6) | 10.5 (3.8 to 21.5) | 5.1 (1.8 to 10.4) | 11.4 (4.6 to 20.8) | 12.6 (5.2 to 22.6) | 10 (3.9 to 18.9) | 41.8 (-7.4 to 122.4) | 19.5 (-29.5 to 93.4) | 97.3 (5.6 to 263.2) |
|  |  |  | All ages | 148.7 (55.5 to 290.2) | 102.7 (38.6 to 210.3) | 46 (16.2 to 96) | 743.5 (300.9 to 1357.1) | 448.2 (185.2 to 809.8) | 295.3 (115.7 to 560.4) | 399.9 (230.9 to 678.2) | 336.3 (161.7 to 595.8) | 541.9 (273.9 to 1060.7) |
|  |  | Deaths | Age-standardized | 0.4 (0.1 to 0.8) | 0.5 (0.2 to 1.2) | 0.2 (0.1 to 0.5) | 0.5 (0.2 to 1) | 0.6 (0.3 to 1.2) | 0.4 (0.2 to 0.8) | 36.6 (-25.1 to 170.8) | 20.4 (-37.5 to 133.4) | 95.5 (-29.2 to 487.4) |
|  |  |  | All ages | 6 (2.1 to 12.5) | 4.6 (1.6 to 10.4) | 1.5 (0.4 to 3.4) | 36.7 (14.9 to 67.7) | 24.3 (9.6 to 44.6) | 12.4 (4.8 to 25) | 510.3 (240.9 to 1099.6) | 434.8 (175.1 to 936.6) | 745.6 (233.7 to 2344.9) |
|  | High systolic blood pressure | DALYs (Disability-Adjusted Life Years) | Age-standardized | 25.1 (7.6 to 45.9) | 26.5 (8.4 to 52.1) | 23.4 (6.3 to 47.3) | 19.6 (6.3 to 33.4) | 18.6 (6.2 to 31.9) | 20.7 (6.6 to 34.8) | -22.2 (-48.3 to 23.1) | -30 (-60.6 to 16) | -11.4 (-45.5 to 53.3) |
|  |  |  | All ages | 437.9 (133.5 to 785.2) | 246.9 (79.6 to 482.1) | 191 (50.9 to 373.3) | 1281.1 (412.1 to 2186.5) | 664.9 (221.7 to 1148.6) | 616.2 (192.4 to 1024) | 192.5 (94.3 to 352.7) | 169.3 (54.1 to 346.4) | 222.6 (99.4 to 471.6) |
|  |  | Deaths | Age-standardized | 1.3 (0.4 to 2.5) | 1.5 (0.4 to 3.1) | 1.1 (0.2 to 2.5) | 0.9 (0.3 to 1.6) | 1 (0.3 to 1.7) | 0.9 (0.3 to 1.5) | -30.8 (-58.8 to 35.2) | -35.4 (-67.9 to 29.3) | -22.3 (-60.7 to 94.9) |
|  |  |  | All ages | 19.6 (6 to 37.3) | 12.4 (3.6 to 26.1) | 7.2 (1.6 to 16.1) | 63.7 (20.8 to 111.5) | 36.7 (12.8 to 67.8) | 27 (8.5 to 48.3) | 224.7 (92.7 to 522.9) | 196.6 (48.3 to 482.6) | 272.7 (90.9 to 840.2) |
|  | Lead exposure | DALYs (Disability-Adjusted Life Years) | Age-standardized | 2 (-0.3 to 5.5) | 2 (-0.3 to 5.6) | 2.1 (-0.3 to 6.1) | 1.6 (-0.2 to 4.2) | 1.5 (-0.2 to 3.8) | 1.7 (-0.2 to 4.7) | -22 (-47.1 to 20.2) | -26.3 (-56.6 to 17.6) | -17.1 (-46.4 to 39.6) |
|  |  |  | All ages | 35.3 (-5.4 to 94.6) | 18.6 (-2.6 to 51.9) | 16.7 (-2.4 to 48.7) | 106.4 (-13.5 to 276.7) | 54.4 (-7.2 to 140.9) | 52 (-6.3 to 142.2) | 201.8 (106 to 359.3) | 192.2 (72.8 to 363.9) | 212.5 (104 to 435.3) |
|  |  | Deaths | Age-standardized | 0.1 (0 to 0.3) | 0.1 (0 to 0.3) | 0.1 (0 to 0.3) | 0.1 (0 to 0.2) | 0.1 (0 to 0.2) | 0.1 (0 to 0.2) | -22.2 (-53.2 to 50.1) | -24.6 (-60.5 to 39.8) | -18.7 (-56.5 to 94.7) |
|  |  |  | All ages | 1.6 (-0.2 to 4.4) | 0.9 (-0.1 to 2.7) | 0.6 (-0.1 to 2.2) | 6 (-0.8 to 15.4) | 3.4 (-0.5 to 8.8) | 2.6 (-0.3 to 7) | 275 (125.8 to 621.4) | 257.1 (87.5 to 567.8) | 301.3 (115.7 to 900.3) |
|  | Smoking | DALYs (Disability-Adjusted Life Years) | Age-standardized | 5.4 (2.9 to 8.9) | 4.3 (2.3 to 7.5) | 6.6 (3.2 to 11.6) | 4.6 (2.6 to 7) | 3.4 (1.8 to 5.4) | 6 (3.4 to 8.9) | -14.1 (-40.9 to 27.4) | -19.2 (-51.6 to 35.7) | -9.4 (-41 to 43.6) |
|  |  |  | All ages | 109.3 (58.7 to 175.5) | 44.7 (23.8 to 76.7) | 64.5 (32.2 to 113) | 288.5 (160.6 to 446) | 117.7 (62.9 to 188.1) | 170.8 (98.2 to 256.8) | 164 (81.2 to 294.1) | 163.1 (57.1 to 352.6) | 164.6 (73.9 to 327.6) |
|  |  | Deaths | Age-standardized | 0.2 (0.1 to 0.4) | 0.2 (0.1 to 0.4) | 0.2 (0.1 to 0.5) | 0.2 (0.1 to 0.3) | 0.1 (0.1 to 0.2) | 0.2 (0.1 to 0.3) | -22.6 (-56.8 to 57.6) | -24.7 (-63.1 to 65.9) | -20.8 (-59.2 to 88.4) |
|  |  |  | All ages | 3.5 (1.7 to 6.2) | 1.7 (0.8 to 3.2) | 1.8 (0.7 to 3.5) | 10.8 (5.8 to 17.3) | 5.2 (2.4 to 9.3) | 5.6 (3.1 to 9.5) | 210.5 (70.9 to 532.8) | 211 (55 to 589.1) | 210 (60.6 to 660.7) |
| Libya | All risk factors | DALYs (Disability-Adjusted Life Years) | Age-standardized | 23.4 (10.7 to 36.7) | 25.9 (11.4 to 42.8) | 20.9 (10 to 33.8) | 31.7 (16.1 to 50.5) | 36.3 (16.6 to 61) | 26.8 (14.2 to 40.6) | 35.5 (3.4 to 92.8) | 39.9 (-3.4 to 110.5) | 28.4 (-0.3 to 83.2) |
|  |  |  | All ages | 381 (181.2 to 596.4) | 204.2 (91 to 338.3) | 176.8 (86.2 to 282.3) | 1357.9 (698.6 to 2130.1) | 770.3 (356.3 to 1289.3) | 587.7 (314.7 to 892.9) | 256.4 (174.2 to 403.3) | 277.3 (160.1 to 467.8) | 232.3 (163.3 to 366.4) |
|  |  | Deaths | Age-standardized | 0.9 (0.4 to 1.5) | 1.2 (0.5 to 2) | 0.7 (0.3 to 1.2) | 1.3 (0.6 to 2.3) | 1.8 (0.7 to 3.2) | 0.9 (0.4 to 1.5) | 43.7 (-9.8 to 136.4) | 52.2 (-15.4 to 155.9) | 25 (-26.3 to 137.3) |
|  |  |  | All ages | 14.2 (6.5 to 23.4) | 8.9 (3.5 to 15.3) | 5.3 (2.4 to 9.5) | 48.9 (21.7 to 85.2) | 33.4 (13.2 to 60.5) | 15.5 (7.6 to 26.7) | 244.6 (113.2 to 471.8) | 277.6 (104.7 to 533.9) | 190 (69 to 447) |
|  | Alcohol use | DALYs (Disability-Adjusted Life Years) | Age-standardized | 0 (0 to 0) | 0 (0 to 0) | 0 (0 to 0) | 0.1 (0.1 to 0.2) | 0 (0 to 0) | 0.2 (0.1 to 0.3) | 18409.4 (7046.8 to 115608) | 6859.7 (2269.9 to 40906.7) | 20705.7 (7892 to 138433.7) |
|  |  |  | All ages | 0 (0 to 0) | 0 (0 to 0) | 0 (0 to 0) | 5 (2.7 to 7.7) | 0.1 (0.1 to 0.2) | 4.8 (2.6 to 7.6) | 45972.8 (18843.3 to 251305.9) | 19150.5 (6560.9 to 98139.4) | 47716.2 (19490.4 to 265107) |
|  |  | Deaths | Age-standardized | 0 (0 to 0) | 0 (0 to 0) | 0 (0 to 0) | 0 (0 to 0) | 0 (0 to 0) | 0 (0 to 0) | 25316.2 (8129.8 to 275342) | 8057.6 (2051.9 to 83781.5) | 29502 (9199.3 to 388591.8) |
|  |  |  | All ages | 0 (0 to 0) | 0 (0 to 0) | 0 (0 to 0) | 0.1 (0 to 0.2) | 0 (0 to 0) | 0.1 (0 to 0.2) | 59253.9 (19437.9 to 530441.4) | 20607.5 (5486.5 to 190701.8) | 65071.7 (21412.3 to 636391.4) |
|  | Diet high in sodium | DALYs (Disability-Adjusted Life Years) | Age-standardized | 0.3 (0 to 2) | 0.2 (0 to 1.6) | 0.4 (0 to 2.4) | 0.4 (0 to 2.3) | 0.3 (0 to 1.8) | 0.5 (0 to 2.7) | 9.5 (-88.2 to 916.6) | 15.1 (-6309.9 to 22842.1) | 8.5 (-87.9 to 1053.2) |
|  |  |  | All ages | 5.6 (0 to 33.2) | 1.8 (0 to 12.9) | 3.8 (0 to 20.3) | 16.2 (0 to 99.8) | 5.5 (0 to 39.6) | 10.7 (0 to 58.6) | 190.6 (-55.7 to 2152.6) | 212.9 (-87561.4 to 43527.8) | 180.3 (-56.1 to 2152.6) |
|  |  | Deaths | Age-standardized | 0 (0 to 0.1) | 0 (0 to 0.1) | 0 (0 to 0.1) | 0 (0 to 0.1) | 0 (0 to 0.1) | 0 (0 to 0.1) | 15.1 (-93.8 to 1049.9) | 26.5 (-24692 to 15725.8) | 8.1 (-93.8 to 1150.9) |
|  |  |  | All ages | 0.2 (0 to 1.2) | 0.1 (0 to 0.5) | 0.1 (0 to 0.6) | 0.5 (0 to 3) | 0.2 (0 to 1.7) | 0.3 (0 to 1.5) | 181.1 (-76.2 to 2680.6) | 219.6 (-60197 to 61123.7) | 156.3 (-76.6 to 2718.4) |
|  | High body-mass index | DALYs (Disability-Adjusted Life Years) | Age-standardized | 4.3 (1.6 to 7.8) | 6.2 (2.2 to 11.6) | 2.6 (0.9 to 4.9) | 13.7 (5.3 to 25.1) | 17.3 (6.8 to 31.4) | 10 (4 to 18.6) | 216.9 (116.2 to 377.8) | 179.9 (86.9 to 334.5) | 284.5 (125 to 573.7) |
|  |  |  | All ages | 73.8 (27.1 to 132.6) | 50.9 (18.6 to 95) | 22.9 (8.3 to 42.6) | 600.1 (234.7 to 1088.3) | 381.4 (153.8 to 684.7) | 218.7 (87.7 to 410.5) | 713.3 (458.2 to 1093.2) | 649.1 (408.3 to 1039.4) | 856.2 (485 to 1496.2) |
|  |  | Deaths | Age-standardized | 0.2 (0.1 to 0.3) | 0.2 (0.1 to 0.5) | 0.1 (0 to 0.2) | 0.6 (0.2 to 1.1) | 0.8 (0.3 to 1.6) | 0.3 (0.1 to 0.7) | 266.9 (91.7 to 568.1) | 242.2 (70.5 to 547.9) | 310.5 (70.4 to 1062.2) |
|  |  |  | All ages | 2.4 (0.8 to 4.7) | 1.8 (0.6 to 3.8) | 0.6 (0.2 to 1.4) | 21.1 (7.7 to 40.4) | 15.3 (5.4 to 30.1) | 5.8 (2 to 11.6) | 766.5 (359.8 to 1480.7) | 744.1 (324 to 1475) | 832 (288.6 to 2326.1) |
|  | High systolic blood pressure | DALYs (Disability-Adjusted Life Years) | Age-standardized | 19.8 (6.6 to 33.1) | 22.9 (7.7 to 39.1) | 16.8 (5.2 to 30.2) | 23.5 (7.5 to 41.7) | 27.8 (8.7 to 50.8) | 19 (6.4 to 33.7) | 18.7 (-8.4 to 56.2) | 21 (-17.9 to 69.9) | 13.5 (-11.7 to 51.2) |
|  |  |  | All ages | 317.7 (106.6 to 531.3) | 179.2 (61.7 to 307) | 138.4 (43.2 to 248.7) | 988.4 (320.8 to 1748.2) | 580.2 (180.1 to 1051.8) | 408.2 (133.3 to 720) | 211.2 (138.6 to 305.3) | 223.7 (122.5 to 353.9) | 194.9 (131.2 to 288.2) |
|  |  | Deaths | Age-standardized | 0.8 (0.3 to 1.4) | 1.1 (0.3 to 1.9) | 0.6 (0.2 to 1.1) | 1 (0.3 to 1.9) | 1.4 (0.4 to 2.8) | 0.6 (0.2 to 1.3) | 24.9 (-22.4 to 92) | 31.4 (-26.2 to 108.8) | 8.6 (-36 to 89.6) |
|  |  |  | All ages | 12.4 (4.2 to 21.5) | 8 (2.7 to 14.1) | 4.4 (1.4 to 8.4) | 37.1 (11.5 to 69.8) | 25.9 (7.3 to 50.9) | 11.2 (3.8 to 22.4) | 198.8 (83.1 to 358.6) | 224.8 (78.3 to 420.3) | 152.1 (47.6 to 342.4) |
|  | Lead exposure | DALYs (Disability-Adjusted Life Years) | Age-standardized | 2.2 (-0.3 to 5.4) | 2 (-0.3 to 5) | 2.4 (-0.3 to 6.5) | 2.2 (-0.3 to 5.7) | 2.2 (-0.3 to 5.7) | 2.3 (-0.3 to 5.8) | 3.5 (-20 to 38.4) | 11 (-27.8 to 57.7) | -2.8 (-22.8 to 31.5) |
|  |  |  | All ages | 35.4 (-4.8 to 89.6) | 15.6 (-2.2 to 39.4) | 19.7 (-2.6 to 54.1) | 89.6 (-12.7 to 229.1) | 44.1 (-6 to 113) | 45.5 (-6.7 to 115.5) | 153.6 (96.8 to 238.4) | 182.7 (84.4 to 300.5) | 130.5 (83.6 to 210.2) |
|  |  | Deaths | Age-standardized | 0.1 (0 to 0.2) | 0.1 (0 to 0.2) | 0.1 (0 to 0.2) | 0.1 (0 to 0.3) | 0.1 (0 to 0.3) | 0.1 (0 to 0.2) | 19.1 (-25.4 to 86) | 31.8 (-33.1 to 109.4) | 3.9 (-35 to 83.5) |
|  |  |  | All ages | 1.3 (-0.2 to 3.3) | 0.7 (-0.1 to 1.8) | 0.6 (-0.1 to 1.7) | 3.6 (-0.6 to 10) | 2.2 (-0.3 to 6.1) | 1.5 (-0.2 to 4) | 175 (69.5 to 335.8) | 215.8 (60.7 to 410.4) | 131.3 (41.7 to 314) |
|  | Smoking | DALYs (Disability-Adjusted Life Years) | Age-standardized | 2.3 (1.3 to 3.6) | 0.2 (0.1 to 0.4) | 4.2 (2.3 to 6.6) | 2.4 (1.3 to 3.7) | 0.2 (0.1 to 0.4) | 4.6 (2.4 to 7.1) | 4.4 (-16.3 to 38.2) | 7.8 (-37.1 to 81.9) | 8.8 (-13.9 to 45.2) |
|  |  |  | All ages | 41.4 (23 to 64.4) | 1.7 (0.9 to 2.9) | 39.7 (21.6 to 62) | 122.4 (68.9 to 192.6) | 5.3 (2.6 to 9.4) | 117.1 (65.5 to 185.3) | 195.4 (138.3 to 286.9) | 212.8 (82.3 to 431.7) | 194.7 (137.5 to 288.2) |
|  |  | Deaths | Age-standardized | 0.1 (0 to 0.1) | 0 (0 to 0) | 0.1 (0.1 to 0.2) | 0.1 (0 to 0.1) | 0 (0 to 0) | 0.1 (0.1 to 0.2) | 3.7 (-37.5 to 88.7) | 16.7 (-49.7 to 140.1) | 6.1 (-38.1 to 94.9) |
|  |  |  | All ages | 0.9 (0.5 to 1.6) | 0.1 (0 to 0.1) | 0.9 (0.5 to 1.5) | 2.4 (1.2 to 4.4) | 0.2 (0.1 to 0.4) | 2.2 (1.1 to 4.1) | 158.5 (55.6 to 375.9) | 202.8 (30.4 to 500.8) | 155.5 (50.9 to 374.6) |
| Morocco | All risk factors | DALYs (Disability-Adjusted Life Years) | Age-standardized | 26.3 (10.8 to 42.6) | 29 (12.1 to 47.3) | 23.7 (8.9 to 39.2) | 33.6 (16.1 to 51.7) | 38.1 (18.3 to 61.3) | 28.6 (13.4 to 45.1) | 27.7 (4.1 to 62) | 31.7 (0.2 to 71) | 20.5 (-5.6 to 63) |
|  |  |  | All ages | 3150 (1320.8 to 5057.8) | 1729.2 (728.8 to 2813.2) | 1420.8 (563.7 to 2325.3) | 9526 (4646.4 to 14654.3) | 5565.5 (2628.2 to 8875.7) | 3960.5 (1897.5 to 6237.3) | 202.4 (148.3 to 279.9) | 221.9 (148.1 to 322.1) | 178.7 (124.7 to 265.3) |
|  |  | Deaths | Age-standardized | 1.2 (0.4 to 2) | 1.4 (0.5 to 2.4) | 0.9 (0.3 to 1.8) | 1.7 (0.7 to 2.7) | 2.1 (0.9 to 3.4) | 1.2 (0.5 to 2.1) | 45.2 (6.2 to 109.1) | 51.4 (2.2 to 111.6) | 31.4 (-14.5 to 151.5) |
|  |  |  | All ages | 117.4 (44.4 to 200.5) | 71.1 (25.5 to 123.1) | 46.2 (13.9 to 83.6) | 395.6 (170.9 to 634.4) | 256.5 (110.5 to 421.5) | 139.1 (60 to 233.4) | 237.1 (149.5 to 372.6) | 260.6 (148 to 399.2) | 200.9 (105.1 to 437.4) |
|  | Alcohol use | DALYs (Disability-Adjusted Life Years) | Age-standardized | 0 (0 to 0.1) | 0 (0 to 0) | 0.1 (0.1 to 0.1) | 0 (0 to 0.1) | 0 (0 to 0) | 0.1 (0 to 0.1) | -6.7 (-31.4 to 25) | -61.2 (-86.6 to -20.5) | -5.6 (-30.8 to 27.1) |
|  |  |  | All ages | 6.7 (4 to 10) | 0.1 (0 to 0.2) | 6.6 (3.9 to 9.8) | 15.2 (8.8 to 22.6) | 0.1 (0 to 0.2) | 15.1 (8.8 to 22.5) | 125.7 (66.5 to 199.7) | -4.9 (-63.8 to 91.3) | 127.9 (67.4 to 204.1) |
|  |  | Deaths | Age-standardized | 0 (0 to 0) | 0 (0 to 0) | 0 (0 to 0) | 0 (0 to 0) | 0 (0 to 0) | 0 (0 to 0) | -0.8 (-35 to 71.5) | -56 (-89.1 to 7.6) | 2.3 (-33.6 to 79.5) |
|  |  |  | All ages | 0.1 (0 to 0.2) | 0 (0 to 0) | 0.1 (0 to 0.2) | 0.3 (0.1 to 0.4) | 0 (0 to 0) | 0.3 (0.1 to 0.4) | 141.2 (55.6 to 301.3) | 5.7 (-70.8 to 134.2) | 146.1 (57.5 to 311.8) |
|  | Diet high in sodium | DALYs (Disability-Adjusted Life Years) | Age-standardized | 0.4 (0 to 2.2) | 0.2 (0 to 1.6) | 0.5 (0 to 3) | 0.4 (0 to 2.6) | 0.3 (0 to 2) | 0.6 (0 to 3.2) | 15.4 (-95.1 to 699.5) | 18.8 (-961.9 to 2656.7) | 16.1 (-95 to 763) |
|  |  |  | All ages | 45.9 (0 to 266.9) | 13.9 (0 to 99.5) | 32.1 (0 to 181.8) | 126.7 (0 to 755.6) | 40.2 (0 to 298.6) | 86.4 (0 to 453.8) | 175.8 (-88.3 to 1474.2) | 190.1 (-2468.4 to 3910) | 169.6 (-88.3 to 1563.3) |
|  |  | Deaths | Age-standardized | 0 (0 to 0.1) | 0 (0 to 0.1) | 0 (0 to 0.1) | 0 (0 to 0.1) | 0 (0 to 0.1) | 0 (0 to 0.1) | 31.3 (-95.5 to 1167.9) | 36.6 (-1450.2 to 3470.8) | 29.8 (-95.6 to 1184.7) |
|  |  |  | All ages | 1.4 (0 to 9.6) | 0.5 (0 to 3.7) | 0.9 (0 to 5.9) | 4.3 (0 to 29.3) | 1.6 (0 to 13.2) | 2.7 (0 to 15.4) | 207.3 (-88.1 to 2301.1) | 225.9 (-2308.5 to 5204.7) | 197.5 (-88.1 to 2374.6) |
|  | High body-mass index | DALYs (Disability-Adjusted Life Years) | Age-standardized | 2.6 (1 to 4.7) | 4.3 (1.5 to 8.4) | 0.8 (0.2 to 2.1) | 7.9 (3.2 to 14.6) | 11 (4.5 to 20.9) | 4.7 (1.8 to 8.2) | 210.8 (121.5 to 337.8) | 157.8 (74.7 to 284.8) | 455.5 (154.7 to 1376.6) |
|  |  |  | All ages | 322.6 (124 to 609.9) | 269.5 (99.7 to 534.6) | 53.1 (14.2 to 127.5) | 2372 (966.4 to 4354.9) | 1690.3 (686.4 to 3209.5) | 681.8 (255.3 to 1199) | 635.3 (441.4 to 891) | 527.1 (333.4 to 807.6) | 1184.3 (508.3 to 3249.7) |
|  |  | Deaths | Age-standardized | 0.1 (0 to 0.2) | 0.2 (0.1 to 0.4) | 0 (0 to 0.1) | 0.4 (0.1 to 0.7) | 0.5 (0.2 to 1) | 0.2 (0.1 to 0.4) | 252.2 (104.6 to 502.2) | 199.7 (62.8 to 426.1) | 529.5 (120.5 to 2402.6) |
|  |  |  | All ages | 11 (3.8 to 22.8) | 9.5 (3.3 to 20) | 1.5 (0.3 to 4.5) | 89 (35.4 to 167.8) | 67.4 (26.7 to 133.6) | 21.6 (7.7 to 40.4) | 711.8 (410.3 to 1212.5) | 612.4 (315.4 to 1097.8) | 1335.4 (441.6 to 4666.4) |
|  | High systolic blood pressure | DALYs (Disability-Adjusted Life Years) | Age-standardized | 23.9 (8.4 to 40.4) | 27 (9.7 to 45.4) | 20.9 (6.2 to 36.5) | 28.9 (9.9 to 47.8) | 33 (11.4 to 56.6) | 24.4 (8.2 to 41.7) | 20.9 (-2.5 to 49.6) | 22.3 (-7.4 to 53.1) | 16.8 (-10.9 to 58.3) |
|  |  |  | All ages | 2834.6 (991.2 to 4777.4) | 1603.7 (578.8 to 2709) | 1231 (368.6 to 2148.1) | 8081.2 (2820.2 to 13479.7) | 4773.3 (1625.3 to 8188.5) | 3307.9 (1139.1 to 5687.5) | 185.1 (131.2 to 251.5) | 197.7 (124.1 to 273) | 168.7 (110.1 to 258.8) |
|  |  | Deaths | Age-standardized | 1.1 (0.3 to 2) | 1.3 (0.4 to 2.3) | 0.9 (0.2 to 1.7) | 1.5 (0.5 to 2.5) | 1.8 (0.6 to 3.2) | 1.1 (0.4 to 1.9) | 38 (-0.4 to 96.5) | 42.1 (-4.5 to 98) | 27.4 (-18.6 to 146.5) |
|  |  |  | All ages | 108.8 (35.2 to 193.7) | 66.8 (22.1 to 118.6) | 41.9 (9.5 to 78.2) | 347.5 (118.8 to 584.7) | 225.6 (77.1 to 396.7) | 121.9 (39.7 to 214.4) | 219.5 (132.2 to 339.8) | 237.5 (127.7 to 358.8) | 190.8 (93.6 to 423.4) |
|  | Lead exposure | DALYs (Disability-Adjusted Life Years) | Age-standardized | 2 (-0.3 to 5.2) | 1.6 (-0.3 to 4.2) | 2.3 (-0.3 to 6.1) | 2.4 (-0.3 to 6) | 2.1 (-0.3 to 5.4) | 2.7 (-0.4 to 7.1) | 22.1 (-3.2 to 58.2) | 29.4 (-10.6 to 82.9) | 17.6 (-7.5 to 58.2) |
|  |  |  | All ages | 231.7 (-32.7 to 610.5) | 97.8 (-15 to 243.7) | 133.9 (-18.6 to 356) | 649.1 (-92.1 to 1617.2) | 296.8 (-45.2 to 764.4) | 352.3 (-47 to 905.6) | 180.1 (125 to 255) | 203.5 (111.2 to 327.6) | 163.1 (112.8 to 242.9) |
|  |  | Deaths | Age-standardized | 0.1 (0 to 0.2) | 0.1 (0 to 0.2) | 0.1 (0 to 0.3) | 0.1 (0 to 0.3) | 0.1 (0 to 0.3) | 0.1 (0 to 0.4) | 46.1 (2.3 to 118.5) | 59.5 (-5.9 to 139.9) | 33.9 (-13.8 to 150.3) |
|  |  |  | All ages | 8.7 (-1.3 to 23.7) | 4 (-0.7 to 10.7) | 4.7 (-0.7 to 13.7) | 29 (-4.6 to 75.7) | 14.8 (-2.2 to 38.1) | 14.2 (-2.2 to 37.3) | 232 (135.9 to 395.2) | 267.2 (117.7 to 443.7) | 202 (102.4 to 432.6) |
|  | Smoking | DALYs (Disability-Adjusted Life Years) | Age-standardized | 1.8 (1 to 2.9) | 0.2 (0.1 to 0.3) | 3.5 (1.8 to 5.5) | 1.5 (0.8 to 2.3) | 0.2 (0.1 to 0.3) | 2.8 (1.6 to 4.3) | -20 (-35.9 to 0.9) | -17.2 (-46.8 to 29) | -19.4 (-36.1 to 2.3) |
|  |  |  | All ages | 250 (130.7 to 397.1) | 12.8 (6 to 22.3) | 237.3 (124.4 to 377.9) | 502.9 (281.2 to 773.7) | 25.6 (13.5 to 43.2) | 477.3 (264.4 to 736.2) | 101.1 (62.8 to 151) | 100.6 (30 to 209.2) | 101.2 (62 to 152.7) |
|  |  | Deaths | Age-standardized | 0 (0 to 0.1) | 0 (0 to 0) | 0.1 (0 to 0.2) | 0 (0 to 0.1) | 0 (0 to 0) | 0.1 (0 to 0.1) | -22.8 (-46.6 to 31.4) | -2.7 (-51.2 to 89.4) | -23.2 (-48.5 to 36.4) |
|  |  |  | All ages | 5.6 (2.4 to 9.4) | 0.4 (0.2 to 0.7) | 5.2 (2.2 to 8.9) | 10.9 (5.9 to 16.9) | 0.9 (0.4 to 1.6) | 10.1 (5.3 to 15.6) | 96.3 (38 to 222.6) | 132.9 (23.8 to 322.8) | 93.7 (32.5 to 227.9) |
| Oman | All risk factors | DALYs (Disability-Adjusted Life Years) | Age-standardized | 20.8 (8.8 to 34.3) | 21.4 (9.2 to 37.9) | 20.9 (8.5 to 36) | 37.8 (20.8 to 56.8) | 38.5 (20.3 to 59.8) | 37.6 (20.5 to 56.5) | 81.6 (27 to 175.6) | 79.6 (9.6 to 187.5) | 80.5 (20.5 to 210.4) |
|  |  |  | All ages | 112.2 (49.5 to 182.6) | 57.1 (24.9 to 100) | 55.1 (25 to 93.1) | 536.2 (294.2 to 801.9) | 256.9 (139.8 to 389.2) | 279.4 (150.4 to 443.4) | 378 (225.1 to 621.4) | 350.1 (180.5 to 607.8) | 406.9 (222.3 to 746.8) |
|  |  | Deaths | Age-standardized | 1 (0.4 to 1.8) | 1 (0.4 to 1.9) | 1 (0.3 to 1.8) | 1.9 (1 to 2.9) | 2 (1 to 3.2) | 1.9 (0.9 to 3) | 91.1 (23.2 to 240.9) | 89.5 (4.2 to 235.5) | 89 (8.2 to 327) |
|  |  |  | All ages | 4.2 (1.8 to 7.4) | 2.6 (1 to 4.7) | 1.7 (0.6 to 3.1) | 18.8 (10.5 to 28.4) | 10.8 (5.6 to 17) | 7.9 (4.4 to 12.1) | 342.8 (173.5 to 649.7) | 324.7 (137.2 to 655.5) | 370.2 (160.7 to 875) |
|  | Alcohol use | DALYs (Disability-Adjusted Life Years) | Age-standardized | 0.1 (0 to 0.1) | 0 (0 to 0) | 0.1 (0.1 to 0.2) | 0.1 (0.1 to 0.2) | 0 (0 to 0) | 0.2 (0.1 to 0.3) | 61.4 (-6 to 209.4) | 7.3 (-50.2 to 147.6) | 64.1 (-6.6 to 216.9) |
|  |  |  | All ages | 0.5 (0.2 to 0.9) | 0 (0 to 0.1) | 0.5 (0.2 to 0.8) | 2.6 (1.3 to 4.3) | 0.1 (0 to 0.2) | 2.5 (1.3 to 4.2) | 402.7 (197.2 to 844.7) | 188.6 (44.5 to 543.1) | 414.9 (201.1 to 870.1) |
|  |  | Deaths | Age-standardized | 0 (0 to 0) | 0 (0 to 0) | 0 (0 to 0) | 0 (0 to 0) | 0 (0 to 0) | 0 (0 to 0) | 65 (-19.5 to 272.2) | 12.4 (-59.6 to 183.3) | 66.3 (-21.2 to 299.7) |
|  |  |  | All ages | 0 (0 to 0) | 0 (0 to 0) | 0 (0 to 0) | 0.1 (0 to 0.1) | 0 (0 to 0) | 0 (0 to 0.1) | 329.7 (98.8 to 888.2) | 165.5 (7.2 to 552.2) | 345.2 (105.1 to 970.5) |
|  | Diet high in sodium | DALYs (Disability-Adjusted Life Years) | Age-standardized | 0.4 (0 to 2.8) | 0.3 (0 to 2.3) | 0.6 (0 to 3.4) | 0.5 (0 to 3.2) | 0.3 (0 to 2.4) | 0.7 (0 to 4.2) | 15.5 (-93 to 641.9) | 2.6 (-189.6 to 7860.7) | 19.9 (-93.6 to 710.2) |
|  |  |  | All ages | 2.5 (0 to 15.3) | 0.8 (0 to 6.2) | 1.7 (0 to 8.8) | 8 (0 to 46.9) | 2.2 (0 to 16.7) | 5.8 (0 to 30.6) | 218.4 (-66.6 to 1855.9) | 165.5 (-422.8 to 22745.8) | 244.5 (-66.7 to 1856.4) |
|  |  | Deaths | Age-standardized | 0 (0 to 0.1) | 0 (0 to 0.1) | 0 (0 to 0.2) | 0 (0 to 0.2) | 0 (0 to 0.1) | 0 (0 to 0.2) | 18.7 (-95 to 1009.6) | 5.7 (-114.7 to 10106.9) | 22.3 (-95.6 to 1047.9) |
|  |  |  | All ages | 0.1 (0 to 0.5) | 0 (0 to 0.3) | 0.1 (0 to 0.3) | 0.2 (0 to 1.6) | 0.1 (0 to 0.6) | 0.2 (0 to 0.9) | 186.4 (-70.7 to 1814.5) | 147.8 (-188 to 20610.1) | 212.8 (-70.7 to 1846.9) |
|  | High body-mass index | DALYs (Disability-Adjusted Life Years) | Age-standardized | 4.1 (1.6 to 7.2) | 5.7 (2.2 to 9.9) | 2.6 (0.8 to 5.3) | 15.1 (6.4 to 26.2) | 16.3 (7.1 to 29.1) | 14.2 (5.9 to 24.8) | 268.3 (96.2 to 521.6) | 188.4 (46.2 to 369.9) | 440.8 (175.2 to 1171.3) |
|  |  |  | All ages | 24.2 (9.7 to 41.5) | 15.9 (6.3 to 27.8) | 8.3 (3 to 15) | 230.6 (95.6 to 398.5) | 116.3 (50.4 to 199.5) | 114.4 (47.7 to 200) | 854.9 (417.2 to 1350) | 632.1 (298.9 to 1049.5) | 1282.7 (590.2 to 2479.5) |
|  |  | Deaths | Age-standardized | 0.2 (0.1 to 0.3) | 0.2 (0.1 to 0.4) | 0.1 (0 to 0.3) | 0.7 (0.3 to 1.3) | 0.8 (0.3 to 1.4) | 0.7 (0.3 to 1.4) | 304.2 (93.5 to 793.7) | 226.9 (40.4 to 570.5) | 533.3 (168.1 to 2238.9) |
|  |  |  | All ages | 0.8 (0.3 to 1.5) | 0.6 (0.2 to 1.1) | 0.2 (0.1 to 0.4) | 7.5 (3.2 to 13.4) | 4.4 (1.9 to 7.9) | 3 (1.2 to 5.1) | 811.6 (357.5 to 1600.7) | 637.2 (232.5 to 1336.4) | 1298.9 (515.4 to 3427.8) |
|  | High systolic blood pressure | DALYs (Disability-Adjusted Life Years) | Age-standardized | 16.9 (5.4 to 30.2) | 17.6 (5.7 to 34.7) | 16.6 (4.7 to 31.2) | 29.1 (10.2 to 48.8) | 30.2 (10.3 to 51) | 28.4 (9.8 to 48) | 72.5 (17.5 to 161.4) | 71 (2.6 to 183.9) | 70.5 (13.7 to 204.4) |
|  |  |  | All ages | 87.1 (27.3 to 157.1) | 46.3 (15 to 90.7) | 40.8 (11.7 to 78.8) | 395.5 (132.3 to 661.5) | 196.5 (66.3 to 330.8) | 199 (63.8 to 345.7) | 354.1 (201.2 to 575.7) | 324.2 (156.9 to 571.5) | 388.1 (208.7 to 732.1) |
|  |  | Deaths | Age-standardized | 0.8 (0.3 to 1.6) | 0.9 (0.3 to 1.8) | 0.8 (0.2 to 1.6) | 1.5 (0.5 to 2.6) | 1.6 (0.5 to 2.8) | 1.4 (0.5 to 2.5) | 79.2 (12.3 to 202.6) | 79 (-3.2 to 229.2) | 74.9 (-1.1 to 309.3) |
|  |  |  | All ages | 3.5 (1.1 to 6.7) | 2.1 (0.7 to 4.3) | 1.3 (0.3 to 2.7) | 14.5 (4.9 to 24.1) | 8.6 (2.9 to 14.8) | 5.9 (2 to 10.2) | 316.8 (150.2 to 589.8) | 299.2 (113.2 to 614.7) | 345.1 (148.2 to 828) |
|  | Lead exposure | DALYs (Disability-Adjusted Life Years) | Age-standardized | 2.6 (-0.4 to 6.6) | 2.5 (-0.4 to 6.6) | 2.9 (-0.4 to 8) | 2.6 (-0.4 to 6.6) | 2.3 (-0.3 to 5.9) | 3.1 (-0.4 to 8) | 0.5 (-27.9 to 38.9) | -7.9 (-41.8 to 31) | 7.8 (-23.6 to 72.7) |
|  |  |  | All ages | 13.3 (-1.9 to 34.7) | 6.5 (-1 to 17.4) | 6.9 (-1 to 19) | 31.6 (-4.6 to 80.3) | 13.9 (-2.1 to 36.1) | 17.7 (-2.4 to 46.3) | 137 (71.6 to 226.5) | 115.1 (34.6 to 204.5) | 157.8 (71.5 to 297.4) |
|  |  | Deaths | Age-standardized | 0.1 (0 to 0.3) | 0.1 (0 to 0.3) | 0.1 (0 to 0.4) | 0.1 (0 to 0.4) | 0.1 (0 to 0.3) | 0.2 (0 to 0.5) | 12.3 (-27.2 to 78) | 3.7 (-42.2 to 66.6) | 19.6 (-31.4 to 145.7) |
|  |  |  | All ages | 0.5 (-0.1 to 1.4) | 0.3 (0 to 0.8) | 0.2 (0 to 0.7) | 1.3 (-0.2 to 3.3) | 0.7 (-0.1 to 1.7) | 0.6 (-0.1 to 1.6) | 142.9 (56.9 to 276.2) | 121 (21.8 to 252) | 171.9 (67.8 to 409.5) |
|  | Smoking | DALYs (Disability-Adjusted Life Years) | Age-standardized | 2.1 (1.2 to 3.4) | 0.8 (0.4 to 1.3) | 3.5 (1.9 to 5.6) | 1.9 (1 to 2.9) | 0.6 (0.3 to 0.9) | 3.2 (1.7 to 5) | -12.2 (-42 to 28.7) | -30.1 (-58.6 to 14.5) | -9.5 (-39.8 to 36.7) |
|  |  |  | All ages | 14.7 (8.3 to 23) | 2.3 (1.2 to 3.8) | 12.4 (6.9 to 19.9) | 39.3 (21.1 to 61.8) | 4.4 (2.3 to 7) | 34.9 (18.5 to 56.2) | 167.8 (75.9 to 290.2) | 89.7 (15.1 to 195.2) | 182.3 (76.3 to 324.5) |
|  |  | Deaths | Age-standardized | 0.1 (0 to 0.1) | 0 (0 to 0.1) | 0.1 (0.1 to 0.2) | 0.1 (0 to 0.1) | 0 (0 to 0) | 0.1 (0.1 to 0.2) | -10 (-45.1 to 61.3) | -28.4 (-63.7 to 40.8) | -7.2 (-46.2 to 87.4) |
|  |  |  | All ages | 0.4 (0.2 to 0.6) | 0.1 (0 to 0.2) | 0.3 (0.1 to 0.5) | 0.9 (0.5 to 1.4) | 0.2 (0.1 to 0.3) | 0.7 (0.4 to 1.2) | 127.6 (25.2 to 304.4) | 69.6 (-11.2 to 213.1) | 145 (29.3 to 376.1) |
| Pakistan | All risk factors | DALYs (Disability-Adjusted Life Years) | Age-standardized | 27.4 (12 to 44.9) | 27.8 (10.5 to 48.6) | 27.3 (12.6 to 44) | 36.2 (16.8 to 57.4) | 35.6 (15.2 to 60) | 36.8 (16.4 to 58.9) | 32 (8 to 56.5) | 28.2 (-2.2 to 72.5) | 34.7 (8.1 to 67.8) |
|  |  |  | All ages | 12972 (5847.6 to 20981.5) | 5499.6 (2159 to 9536.5) | 7472.4 (3486.8 to 12024.4) | 33490.9 (15695.1 to 52488.1) | 15370.1 (6786.8 to 25046.5) | 18120.8 (8302.2 to 28825.3) | 158.2 (117.7 to 202.4) | 179.5 (118.3 to 265.7) | 142.5 (97.2 to 197.1) |
|  |  | Deaths | Age-standardized | 0.9 (0.3 to 1.7) | 1.1 (0.4 to 2.2) | 0.8 (0.3 to 1.4) | 1.5 (0.6 to 2.6) | 1.6 (0.7 to 3) | 1.4 (0.5 to 2.5) | 64.8 (17.4 to 131.4) | 50.4 (-2.3 to 131.8) | 75.6 (15.6 to 175.6) |
|  |  |  | All ages | 352.2 (129.1 to 636.6) | 174.4 (63.1 to 359) | 177.8 (63.6 to 312) | 1077.1 (458.7 to 1839.5) | 554.6 (223.6 to 1010.8) | 522.5 (218.3 to 877.5) | 205.8 (122.9 to 325.6) | 218.1 (108.9 to 379.7) | 193.8 (100.7 to 358.3) |
|  | Alcohol use | DALYs (Disability-Adjusted Life Years) | Age-standardized | 0.1 (0 to 0.2) | 0 (0 to 0) | 0.2 (0 to 0.4) | 0.3 (0.1 to 0.5) | 0.1 (0 to 0.1) | 0.5 (0.2 to 0.8) | 125.4 (41.5 to 616) | 149.3 (41.2 to 703.8) | 137.7 (44.8 to 672) |
|  |  |  | All ages | 66.2 (13.1 to 126.1) | 5.3 (1.2 to 10.5) | 60.8 (11.2 to 118.5) | 316.5 (133.9 to 533.9) | 30.5 (13.8 to 53.9) | 286 (117.2 to 485.3) | 378.4 (212.4 to 1561.8) | 471.5 (232.4 to 1783.1) | 370.3 (202 to 1570.5) |
|  |  | Deaths | Age-standardized | 0 (0 to 0) | 0 (0 to 0) | 0 (0 to 0) | 0 (0 to 0) | 0 (0 to 0) | 0 (0 to 0) | 192.5 (53.4 to 720.9) | 198.5 (43 to 799.5) | 216.4 (58.3 to 850.1) |
|  |  |  | All ages | 1.2 (0.2 to 2.5) | 0.1 (0 to 0.3) | 1.1 (0.2 to 2.2) | 6.8 (2.5 to 12.5) | 0.8 (0.3 to 1.6) | 6.1 (2.1 to 11.3) | 471.1 (221.4 to 1716.7) | 544 (242.4 to 2023.7) | 462.9 (205.1 to 1769.4) |
|  | Diet high in sodium | DALYs (Disability-Adjusted Life Years) | Age-standardized | 1.5 (0 to 6) | 1.3 (0 to 5.5) | 1.6 (0 to 6.3) | 2.6 (0 to 9.3) | 2.5 (0 to 9) | 2.8 (0 to 9.4) | 82.1 (38.4 to 1256.1) | 91.5 (35.5 to 2128.9) | 78.5 (24.7 to 1620.2) |
|  |  |  | All ages | 654.3 (2.1 to 2726.8) | 246 (0.3 to 1080) | 408.3 (1.1 to 1681.5) | 2305.1 (32.8 to 8086.9) | 1013.3 (7.7 to 3700.6) | 1291.8 (18.5 to 4432.5) | 252.3 (170.1 to 2823.7) | 311.9 (197.6 to 4809.1) | 216.3 (127.2 to 2842.2) |
|  |  | Deaths | Age-standardized | 0.1 (0 to 0.2) | 0.1 (0 to 0.2) | 0.1 (0 to 0.2) | 0.1 (0 to 0.5) | 0.1 (0 to 0.5) | 0.1 (0 to 0.4) | 126.1 (51 to 1625.9) | 126.3 (39.9 to 3273.3) | 126.1 (31.3 to 2142.7) |
|  |  |  | All ages | 19.2 (0.1 to 75.8) | 8.2 (0 to 37.6) | 11 (0 to 46.3) | 81.2 (1.1 to 297.4) | 39.2 (0.3 to 149.5) | 42 (0.5 to 153.6) | 322.4 (196.2 to 3366.3) | 379 (208.5 to 6149.5) | 280.4 (130.1 to 4222.6) |
|  | High body-mass index | DALYs (Disability-Adjusted Life Years) | Age-standardized | 0.3 (0.1 to 0.8) | 0.6 (0.1 to 1.4) | 0.2 (0 to 0.5) | 3.9 (1.5 to 6.9) | 4.9 (2 to 8.9) | 3 (1 to 5.7) | 1059.9 (487.6 to 2981.7) | 767.3 (326.7 to 2048.8) | 1818.9 (-3337.6 to 24824.5) |
|  |  |  | All ages | 189.9 (37.6 to 446.3) | 140.8 (35.5 to 332.2) | 49 (1.1 to 138.2) | 4136.6 (1556 to 6994) | 2485.8 (978.9 to 4389.2) | 1650.7 (564.2 to 3136.7) | 2078.8 (1024.6 to 5085.8) | 1665.2 (812.3 to 3963) | 3267 (1001.4 to 26243.3) |
|  |  | Deaths | Age-standardized | 0 (0 to 0) | 0 (0 to 0) | 0 (0 to 0) | 0.1 (0 to 0.3) | 0.2 (0.1 to 0.4) | 0.1 (0 to 0.2) | 1716.2 (543.7 to 8700.9) | 1211.4 (375.2 to 5448.3) | 3309 (-46150.7 to 55691.2) |
|  |  |  | All ages | 3.5 (0.5 to 10.3) | 2.8 (0.4 to 8) | 0.8 (-0.2 to 2.9) | 107.5 (41.8 to 204.6) | 69.2 (25.7 to 139.2) | 38.3 (11.7 to 80.2) | 2931.6 (1114.7 to 10398.1) | 2396.4 (904.2 to 8249.8) | 4851.7 (-38311.8 to 56059.1) |
|  | High systolic blood pressure | DALYs (Disability-Adjusted Life Years) | Age-standardized | 23.1 (7.8 to 40.1) | 26.7 (9.1 to 47.6) | 20.5 (6.3 to 36.5) | 31.3 (11 to 54) | 32.5 (12 to 57.5) | 30.2 (9.8 to 53.3) | 35.6 (11.7 to 65.1) | 22 (-8.9 to 58.7) | 47.3 (19.3 to 88.3) |
|  |  |  | All ages | 10749.9 (3581.1 to 18644.3) | 5236.7 (1807.8 to 9246.4) | 5513.2 (1701.9 to 9772.2) | 28327.4 (9933.1 to 48125.6) | 13834.6 (5092.7 to 24091.2) | 14492.7 (4643.3 to 25355.8) | 163.5 (121.5 to 214.2) | 164.2 (104.3 to 239.3) | 162.9 (113.1 to 232.3) |
|  |  | Deaths | Age-standardized | 0.8 (0.2 to 1.6) | 1.1 (0.4 to 2.2) | 0.6 (0.2 to 1.3) | 1.4 (0.5 to 2.5) | 1.5 (0.5 to 2.9) | 1.2 (0.4 to 2.3) | 67 (17.5 to 133) | 43.8 (-7.4 to 118) | 89.4 (25.3 to 203.7) |
|  |  |  | All ages | 307.9 (93.4 to 590.9) | 169.2 (56.3 to 352.3) | 138.6 (32.1 to 272.7) | 949.6 (327.7 to 1720) | 512.1 (188.3 to 959.4) | 437.5 (131.8 to 793.5) | 208.4 (120.7 to 321.8) | 202.6 (98.6 to 352.6) | 215.6 (114.9 to 397.7) |
|  | Lead exposure | DALYs (Disability-Adjusted Life Years) | Age-standardized | 3.6 (-0.5 to 9.2) | 2.8 (-0.4 to 7.5) | 4.2 (-0.6 to 10.7) | 4.9 (-0.7 to 12) | 4.4 (-0.6 to 11.4) | 5.3 (-0.8 to 12.9) | 36 (15.9 to 67) | 56.2 (22.2 to 111.5) | 27.8 (5.3 to 60.8) |
|  |  |  | All ages | 1664.1 (-239.5 to 4311.1) | 557.3 (-79.1 to 1494.9) | 1106.8 (-163.8 to 2853.3) | 4153.9 (-569 to 10201.7) | 1756.7 (-238.7 to 4616.4) | 2397.2 (-345.3 to 5847.8) | 149.6 (114.2 to 204.7) | 215.2 (148.1 to 308.5) | 116.6 (81.9 to 166.6) |
|  |  | Deaths | Age-standardized | 0.1 (0 to 0.3) | 0.1 (0 to 0.3) | 0.1 (0 to 0.4) | 0.2 (0 to 0.6) | 0.2 (0 to 0.6) | 0.2 (0 to 0.6) | 86.7 (36.6 to 167.3) | 104.7 (36.7 to 218.4) | 76.6 (20.4 to 172.4) |
|  |  |  | All ages | 45.3 (-7.2 to 124.1) | 17.3 (-3 to 47.8) | 28 (-4 to 81.3) | 151 (-23.3 to 380.5) | 71.2 (-10.8 to 186.7) | 79.7 (-13.5 to 208.2) | 233.2 (145.6 to 375.4) | 311.9 (179.6 to 526.2) | 184.7 (100 to 347.1) |
|  | Smoking | DALYs (Disability-Adjusted Life Years) | Age-standardized | 5.5 (3 to 8.7) | 1.2 (0.6 to 2.1) | 8.8 (4.7 to 13.9) | 4.2 (2.3 to 6.5) | 1 (0.5 to 1.7) | 7.1 (4 to 10.9) | -24.1 (-38.7 to -4) | -19.1 (-46.8 to 19.9) | -19.2 (-35.6 to 4.1) |
|  |  |  | All ages | 2832.4 (1519 to 4441.5) | 291.9 (149.8 to 500.1) | 2540.5 (1358.1 to 4036.6) | 4471.5 (2465.3 to 6675.8) | 511.5 (256.1 to 851.8) | 3960 (2190.3 to 5974.4) | 57.9 (30.4 to 94.4) | 75.2 (18 to 157.6) | 55.9 (26.1 to 94.1) |
|  |  | Deaths | Age-standardized | 0.1 (0.1 to 0.2) | 0 (0 to 0.1) | 0.2 (0.1 to 0.4) | 0.1 (0.1 to 0.2) | 0 (0 to 0.1) | 0.2 (0.1 to 0.4) | -4.8 (-36.8 to 55) | 16.1 (-41 to 122.2) | 1.4 (-34.8 to 68.1) |
|  |  |  | All ages | 56.9 (26 to 93.8) | 5.8 (2.7 to 11.4) | 51.1 (21.9 to 85.7) | 105.5 (54.3 to 166.7) | 13.5 (6.2 to 25.9) | 92 (46.3 to 148) | 85.4 (29.6 to 190.3) | 131.3 (28.8 to 318.9) | 80.2 (19.4 to 194.4) |
| Palestine | All risk factors | DALYs (Disability-Adjusted Life Years) | Age-standardized | 30.7 (15.3 to 46.8) | 30.9 (15.4 to 48.8) | 30.6 (15.4 to 48.1) | 34.8 (19.2 to 50.8) | 34.1 (18.4 to 49.8) | 37.3 (20.8 to 57.4) | 13.3 (-12.5 to 51.9) | 10.4 (-20.3 to 59.7) | 21.8 (-11.7 to 67.9) |
|  |  |  | All ages | 210.9 (107.3 to 320.2) | 120.5 (59.9 to 190.8) | 90.4 (47.3 to 138.3) | 612.4 (346.6 to 884.9) | 336 (186.9 to 495.8) | 276.5 (153.9 to 404.3) | 190.4 (127 to 284) | 178.7 (101.3 to 295.4) | 205.9 (135.5 to 319.3) |
|  |  | Deaths | Age-standardized | 1.7 (0.8 to 2.8) | 1.8 (0.8 to 2.9) | 1.6 (0.7 to 2.8) | 2 (1 to 3) | 2 (1 to 3) | 2.1 (1.1 to 3.8) | 15.3 (-17.3 to 71.8) | 12.8 (-25.8 to 81.3) | 29.5 (-17.8 to 109.6) |
|  |  |  | All ages | 9.8 (4.6 to 15.6) | 6.2 (2.9 to 10.2) | 3.6 (1.6 to 5.8) | 26 (14.6 to 38.4) | 16.6 (8.8 to 24.4) | 9.4 (5.1 to 14.5) | 165.3 (90.4 to 292.7) | 169.2 (80.2 to 332.7) | 158.6 (72.5 to 317.6) |
|  | Alcohol use | DALYs (Disability-Adjusted Life Years) | Age-standardized | 0.3 (0.2 to 0.5) | 0.1 (0 to 0.1) | 0.7 (0.4 to 1) | 0.3 (0.2 to 0.4) | 0.1 (0 to 0.1) | 0.6 (0.4 to 0.9) | -13.6 (-39.7 to 28.3) | -20 (-60.7 to 91.7) | -11.8 (-40.3 to 30.6) |
|  |  |  | All ages | 2.5 (1.5 to 3.7) | 0.3 (0.1 to 0.5) | 2.3 (1.4 to 3.3) | 6.5 (4.3 to 9.2) | 0.6 (0.3 to 0.9) | 5.9 (3.8 to 8.4) | 155.9 (80.9 to 276.8) | 107.9 (1.3 to 379.5) | 161.9 (83.8 to 281.7) |
|  |  | Deaths | Age-standardized | 0 (0 to 0) | 0 (0 to 0) | 0 (0 to 0.1) | 0 (0 to 0) | 0 (0 to 0) | 0 (0 to 0) | -21.1 (-51.6 to 37.6) | -17.8 (-66 to 130.7) | -11.3 (-52.5 to 56.3) |
|  |  |  | All ages | 0.1 (0 to 0.1) | 0 (0 to 0) | 0.1 (0 to 0.1) | 0.2 (0.1 to 0.3) | 0 (0 to 0) | 0.1 (0.1 to 0.2) | 99.6 (23.7 to 238.7) | 98.7 (-10 to 441.5) | 99.7 (22.4 to 243.6) |
|  | Diet high in sodium | DALYs (Disability-Adjusted Life Years) | Age-standardized | 0.4 (0 to 2.8) | 0.3 (0 to 2.2) | 0.6 (0 to 3.7) | 0.4 (0 to 2.9) | 0.3 (0 to 2.3) | 0.7 (0 to 3.8) | -1 (-92.8 to 698) | 1.3 (-161965 to 63195) | 0.9 (-93.3 to 664.6) |
|  |  |  | All ages | 3.2 (0 to 19.7) | 1.2 (0 to 9) | 2 (0 to 11) | 8.4 (0 to 52.8) | 3 (0 to 22.9) | 5.4 (0 to 29.4) | 161.8 (-65.7 to 2243.3) | 155.7 (-425972.4 to 781273.6) | 165.3 (-66 to 2290.8) |
|  |  | Deaths | Age-standardized | 0 (0 to 0.1) | 0 (0 to 0.1) | 0 (0 to 0.2) | 0 (0 to 0.2) | 0 (0 to 0.1) | 0 (0 to 0.2) | -1.2 (-95.8 to 1000.9) | 5.8 (-1678.3 to 59781.6) | 3.9 (-96.1 to 971.9) |
|  |  |  | All ages | 0.1 (0 to 0.8) | 0.1 (0 to 0.4) | 0.1 (0 to 0.4) | 0.3 (0 to 2.1) | 0.1 (0 to 1.1) | 0.2 (0 to 1) | 133.8 (-81.9 to 2424.9) | 154.2 (-10792.2 to 323154.5) | 119.8 (-82.6 to 2458.3) |
|  | High body-mass index | DALYs (Disability-Adjusted Life Years) | Age-standardized | 8.5 (3.2 to 16.5) | 10.6 (3.9 to 20.2) | 5.7 (2 to 11) | 16.3 (6.8 to 27.7) | 18.6 (8 to 31.2) | 13.7 (5.2 to 24.3) | 92.5 (37.6 to 171.7) | 75.8 (21.4 to 156) | 140.6 (46.6 to 325.6) |
|  |  |  | All ages | 59.5 (22.3 to 113.9) | 42.8 (16.2 to 81.1) | 16.8 (6 to 32.3) | 289.2 (121.3 to 495.7) | 187.2 (80.1 to 313.6) | 102 (40.4 to 183.3) | 385.8 (254.3 to 555.9) | 337.9 (210.2 to 511.8) | 507.7 (302.5 to 863.7) |
|  |  | Deaths | Age-standardized | 0.5 (0.2 to 0.9) | 0.6 (0.2 to 1.1) | 0.3 (0.1 to 0.7) | 0.9 (0.4 to 1.6) | 1.1 (0.4 to 1.8) | 0.8 (0.3 to 1.6) | 103.8 (31.5 to 223.3) | 88.6 (16.3 to 226.2) | 158.9 (34.2 to 498.3) |
|  |  |  | All ages | 2.7 (1 to 5.3) | 2 (0.8 to 4) | 0.7 (0.2 to 1.3) | 12.3 (5.3 to 20.6) | 8.9 (3.7 to 14.9) | 3.4 (1.3 to 6) | 361.5 (199 to 626) | 343.4 (177.7 to 646.2) | 416.7 (195.2 to 1026.1) |
|  | High systolic blood pressure | DALYs (Disability-Adjusted Life Years) | Age-standardized | 23.3 (7.7 to 39.4) | 24.5 (8.2 to 42.9) | 21.7 (6.5 to 39.6) | 23.4 (7.5 to 40.8) | 23.2 (7.7 to 40.9) | 25 (8.1 to 45.8) | 0.5 (-25.4 to 37.4) | -5.4 (-35.7 to 42.1) | 15 (-19.6 to 67.3) |
|  |  |  | All ages | 157.7 (52.3 to 270.5) | 94.9 (31.2 to 165.2) | 62.8 (19.5 to 112.9) | 400.7 (129.6 to 688.5) | 225.7 (75 to 398.6) | 175 (56.1 to 306.7) | 154.1 (91.6 to 238.6) | 137.9 (62.9 to 247.6) | 178.5 (102.2 to 289.3) |
|  |  | Deaths | Age-standardized | 1.3 (0.4 to 2.4) | 1.4 (0.5 to 2.6) | 1.2 (0.3 to 2.3) | 1.4 (0.4 to 2.4) | 1.4 (0.5 to 2.4) | 1.5 (0.5 to 3) | 1.6 (-32.4 to 53.7) | -3.8 (-41.4 to 64.3) | 22.2 (-25.6 to 112.8) |
|  |  |  | All ages | 7.6 (2.4 to 13.3) | 5 (1.6 to 9) | 2.6 (0.8 to 4.9) | 17.8 (5.8 to 30.8) | 11.5 (3.8 to 20.2) | 6.3 (2 to 11.9) | 134.1 (58.8 to 251.2) | 130.4 (43.5 to 286.2) | 141.3 (50.8 to 296.5) |
|  | Lead exposure | DALYs (Disability-Adjusted Life Years) | Age-standardized | 4.1 (-0.6 to 10.6) | 3.5 (-0.6 to 9.1) | 4.9 (-0.6 to 12.8) | 3.9 (-0.6 to 10.4) | 3.6 (-0.5 to 9.3) | 4.8 (-0.7 to 13.1) | -2.9 (-22.4 to 24) | 2.1 (-24.7 to 37.2) | -3.4 (-26.3 to 28.3) |
|  |  |  | All ages | 26.9 (-3.8 to 69.3) | 13.4 (-2.1 to 35.4) | 13.5 (-1.7 to 34.3) | 61.2 (-8.6 to 158.4) | 32.5 (-4.7 to 84.8) | 28.7 (-4 to 73.6) | 127.6 (82.4 to 184.1) | 142.1 (79.9 to 224.5) | 113 (66.8 to 181.9) |
|  |  | Deaths | Age-standardized | 0.2 (0 to 0.6) | 0.2 (0 to 0.6) | 0.3 (0 to 0.8) | 0.3 (0 to 0.7) | 0.2 (0 to 0.6) | 0.3 (0 to 0.9) | 5.9 (-20.4 to 47.5) | 12.4 (-22.8 to 68.4) | 6.3 (-26.5 to 56.7) |
|  |  |  | All ages | 1.3 (-0.2 to 3.5) | 0.7 (-0.1 to 1.9) | 0.6 (-0.1 to 1.6) | 3.1 (-0.5 to 7.9) | 1.9 (-0.3 to 4.8) | 1.2 (-0.2 to 3.2) | 129.8 (70.4 to 221.3) | 157.5 (76.3 to 283.1) | 96.5 (39.3 to 200.9) |
|  | Smoking | DALYs (Disability-Adjusted Life Years) | Age-standardized | 3.9 (2.1 to 5.9) | 1.3 (0.6 to 2.3) | 7.5 (4.1 to 11.4) | 3.5 (2 to 5.3) | 0.9 (0.5 to 1.7) | 7.2 (4 to 11.6) | -10.7 (-30.1 to 18.7) | -28.2 (-60.6 to 36.7) | -4.6 (-29 to 29.6) |
|  |  |  | All ages | 29.4 (16.8 to 43.9) | 5.1 (2.5 to 8.7) | 24.3 (14.1 to 36.5) | 77 (44.4 to 115.3) | 9.7 (5 to 16.8) | 67.3 (38.7 to 99.9) | 161.7 (108.5 to 242.9) | 91.3 (11.3 to 229.6) | 176.4 (117.6 to 261) |
|  |  | Deaths | Age-standardized | 0.2 (0.1 to 0.3) | 0.1 (0 to 0.1) | 0.3 (0.2 to 0.6) | 0.1 (0.1 to 0.2) | 0.1 (0 to 0.1) | 0.3 (0.2 to 0.6) | -18.4 (-45.2 to 27.2) | -30.3 (-69.3 to 69.5) | -2.7 (-38.6 to 52.2) |
|  |  |  | All ages | 1.1 (0.6 to 1.7) | 0.3 (0.1 to 0.5) | 0.8 (0.4 to 1.3) | 2.2 (1.2 to 3.4) | 0.4 (0.2 to 0.9) | 1.8 (0.9 to 2.7) | 104 (41.1 to 215.2) | 69.4 (-20.1 to 276.5) | 114.9 (45.2 to 236.7) |
| Qatar | All risk factors | DALYs (Disability-Adjusted Life Years) | Age-standardized | 43.5 (17.4 to 70.6) | 52.4 (18 to 89.8) | 34.4 (14 to 60.6) | 35.2 (17.1 to 56.6) | 39.9 (14.7 to 68) | 29.1 (14.9 to 47.3) | -19 (-41.2 to 10.4) | -23.8 (-46.3 to 6.6) | -15.6 (-42.8 to 36.5) |
|  |  |  | All ages | 24.1 (11.7 to 37.7) | 10.2 (3.8 to 17.4) | 13.9 (7 to 21.8) | 188.4 (105.1 to 300.5) | 64.7 (30.7 to 106.1) | 123.7 (66.1 to 207.6) | 681.6 (461.1 to 1054.3) | 533.2 (348.4 to 827.3) | 791 (484.7 to 1279.9) |
|  |  | Deaths | Age-standardized | 2.7 (0.8 to 4.6) | 3.4 (1 to 6) | 1.8 (0.5 to 3.7) | 1.8 (0.5 to 3.1) | 2.3 (0.5 to 4.3) | 1.1 (0.5 to 2.4) | -33.4 (-56.5 to -3.5) | -33 (-63.3 to 0) | -38.3 (-63 to 8.1) |
|  |  |  | All ages | 0.9 (0.3 to 1.5) | 0.5 (0.2 to 0.9) | 0.4 (0.1 to 0.7) | 4.5 (2.1 to 8) | 2.6 (0.7 to 5) | 1.9 (0.9 to 3.2) | 415.6 (222.2 to 731.7) | 410 (215.7 to 655.5) | 423.9 (148.5 to 1137.2) |
|  | Alcohol use | DALYs (Disability-Adjusted Life Years) | Age-standardized | 0.2 (0.1 to 0.3) | 0 (0 to 0.1) | 0.3 (0.2 to 0.5) | 0.2 (0.1 to 0.2) | 0 (0 to 0) | 0.2 (0.1 to 0.4) | -28.2 (-56.6 to 21.8) | -49.1 (-76.7 to 7.5) | -27.9 (-56.8 to 24) |
|  |  |  | All ages | 0.2 (0.1 to 0.4) | 0 (0 to 0) | 0.2 (0.1 to 0.4) | 1.9 (1 to 3.2) | 0 (0 to 0.1) | 1.9 (0.9 to 3.1) | 700.8 (389.7 to 1143.8) | 354.9 (128.9 to 856.8) | 711.5 (394 to 1167.9) |
|  |  | Deaths | Age-standardized | 0 (0 to 0) | 0 (0 to 0) | 0 (0 to 0) | 0 (0 to 0) | 0 (0 to 0) | 0 (0 to 0) | -51.5 (-76.2 to 6.3) | -56.4 (-83.9 to 4.4) | -50.7 (-75.9 to 5.8) |
|  |  |  | All ages | 0 (0 to 0) | 0 (0 to 0) | 0 (0 to 0) | 0 (0 to 0) | 0 (0 to 0) | 0 (0 to 0) | 372.8 (87.7 to 1094.2) | 242.2 (49.8 to 619.8) | 382.4 (87.4 to 1138.2) |
|  | Diet high in sodium | DALYs (Disability-Adjusted Life Years) | Age-standardized | 0.6 (0 to 4.1) | 0.4 (0 to 3.6) | 0.7 (0 to 4.7) | 0.4 (0 to 2.6) | 0.3 (0 to 2.2) | 0.5 (0 to 3.1) | -31.3 (-93.4 to 564.7) | -35.8 (-422.5 to 26039.2) | -29.9 (-93.7 to 563.1) |
|  |  |  | All ages | 0.4 (0 to 2.4) | 0.1 (0 to 0.7) | 0.3 (0 to 1.7) | 2.8 (0 to 16.1) | 0.5 (0 to 3.8) | 2.3 (0 to 12.2) | 588.7 (-15.1 to 4540.2) | 451.7 (-61559.6 to 42280.8) | 629.3 (-15.2 to 4568.4) |
|  |  | Deaths | Age-standardized | 0 (0 to 0.2) | 0 (0 to 0.2) | 0 (0 to 0.2) | 0 (0 to 0.1) | 0 (0 to 0.1) | 0 (0 to 0.1) | -47.4 (-98.2 to 563) | -45.2 (-16163.6 to 4246.8) | -49.1 (-98.5 to 558.6) |
|  |  |  | All ages | 0 (0 to 0.1) | 0 (0 to 0) | 0 (0 to 0) | 0.1 (0 to 0.3) | 0 (0 to 0.1) | 0 (0 to 0.2) | 332.1 (-59.6 to 4277.7) | 332.9 (-109030.5 to 35712.1) | 331.7 (-59.7 to 4276.3) |
|  | High body-mass index | DALYs (Disability-Adjusted Life Years) | Age-standardized | 11.9 (3.8 to 25.1) | 16.5 (5.2 to 34.6) | 7.9 (2.6 to 17.1) | 17.4 (6.7 to 32.2) | 21.7 (7.6 to 41.2) | 12.9 (5.4 to 24) | 45.9 (-0.4 to 113) | 31.4 (-12.4 to 90.9) | 63.1 (-0.8 to 186) |
|  |  |  | All ages | 7.4 (2.6 to 14.3) | 3.6 (1.2 to 7) | 3.9 (1.4 to 7.5) | 98.9 (43.7 to 170.7) | 38.6 (15.1 to 69) | 60.3 (25.9 to 109.4) | 1234 (825.1 to 1866) | 986 (661.2 to 1413.8) | 1462.5 (882.7 to 2505.6) |
|  |  | Deaths | Age-standardized | 0.7 (0.2 to 1.6) | 1 (0.3 to 2.3) | 0.4 (0.1 to 0.9) | 0.9 (0.2 to 1.8) | 1.2 (0.3 to 2.5) | 0.5 (0.2 to 1.2) | 23.4 (-24 to 100.2) | 18.4 (-34.6 to 98.2) | 24.5 (-35.2 to 171.4) |
|  |  |  | All ages | 0.2 (0.1 to 0.6) | 0.2 (0 to 0.3) | 0.1 (0 to 0.2) | 2.3 (0.9 to 4.4) | 1.4 (0.4 to 2.9) | 0.8 (0.3 to 1.8) | 828.1 (465.9 to 1451.6) | 790 (446.5 to 1288.9) | 900.5 (341.9 to 2357.7) |
|  | High systolic blood pressure | DALYs (Disability-Adjusted Life Years) | Age-standardized | 35.8 (11.3 to 63.1) | 43.9 (12.7 to 79.7) | 27.9 (8.4 to 53.9) | 25.3 (8.1 to 46.8) | 28.7 (7.5 to 55) | 20.9 (7.3 to 37.3) | -29.4 (-48 to -6.9) | -34.7 (-55.4 to -10.8) | -25.1 (-49.7 to 15.8) |
|  |  |  | All ages | 18.2 (6.4 to 31.8) | 8.3 (2.5 to 15.1) | 9.9 (3.1 to 18.1) | 121.7 (39.3 to 221.2) | 43.4 (13.1 to 82.8) | 78.2 (23.9 to 152) | 567.7 (374.8 to 865.6) | 421.5 (255 to 631.5) | 690.9 (415.2 to 1185.7) |
|  |  | Deaths | Age-standardized | 2.2 (0.6 to 4.1) | 2.9 (0.8 to 5.4) | 1.5 (0.4 to 3.3) | 1.3 (0.3 to 2.5) | 1.7 (0.3 to 3.4) | 0.8 (0.2 to 1.9) | -42.1 (-62.6 to -17.3) | -42.1 (-69.9 to -12.1) | -45.5 (-66.8 to -10.3) |
|  |  |  | All ages | 0.7 (0.2 to 1.3) | 0.4 (0.1 to 0.8) | 0.3 (0.1 to 0.6) | 3.2 (1 to 6.2) | 1.9 (0.4 to 3.9) | 1.3 (0.4 to 2.6) | 345.9 (178.5 to 582.1) | 336.5 (159.4 to 546.9) | 360.5 (122.6 to 959.7) |
|  | Lead exposure | DALYs (Disability-Adjusted Life Years) | Age-standardized | 2.1 (-0.3 to 5.6) | 2.3 (-0.3 to 6.5) | 1.9 (-0.3 to 5.1) | 1.4 (-0.2 to 3.9) | 1.5 (-0.2 to 4.3) | 1.3 (-0.2 to 3.5) | -30.9 (-46.6 to -11.3) | -34 (-53.8 to -11.4) | -28.9 (-49.9 to 4.8) |
|  |  |  | All ages | 1.1 (-0.1 to 3) | 0.4 (-0.1 to 1.3) | 0.7 (-0.1 to 1.9) | 6.4 (-0.7 to 17.3) | 2.3 (-0.3 to 6.2) | 4.1 (-0.5 to 11) | 463.4 (321.9 to 670.7) | 413.8 (267.7 to 590.1) | 494.8 (302.9 to 795.7) |
|  |  | Deaths | Age-standardized | 0.1 (0 to 0.4) | 0.2 (0 to 0.4) | 0.1 (0 to 0.3) | 0.1 (0 to 0.2) | 0.1 (0 to 0.3) | 0.1 (0 to 0.2) | -40.3 (-60.6 to -18.6) | -39.8 (-67.4 to -13.4) | -43.9 (-63.3 to -11.2) |
|  |  |  | All ages | 0 (0 to 0.1) | 0 (0 to 0.1) | 0 (0 to 0.1) | 0.2 (0 to 0.5) | 0.1 (0 to 0.3) | 0.1 (0 to 0.2) | 331.3 (173.9 to 538.4) | 348.3 (165.7 to 539.6) | 311 (119.4 to 773.9) |
|  | Smoking | DALYs (Disability-Adjusted Life Years) | Age-standardized | 2.4 (1.3 to 3.9) | 0.6 (0.2 to 1.1) | 3.5 (1.9 to 6) | 1.7 (0.9 to 2.9) | 0.3 (0.1 to 0.6) | 2.6 (1.4 to 4.3) | -27.7 (-51.5 to 10) | -47.3 (-72.5 to -4) | -26.3 (-51.2 to 13.3) |
|  |  |  | All ages | 2.9 (1.6 to 4.8) | 0.2 (0.1 to 0.3) | 2.7 (1.5 to 4.5) | 22.7 (10.9 to 40.5) | 0.8 (0.4 to 1.4) | 21.9 (10.5 to 39.5) | 685.9 (426.6 to 1059.6) | 406.8 (202.4 to 734) | 701.6 (432.4 to 1095.6) |
|  |  | Deaths | Age-standardized | 0.1 (0 to 0.2) | 0 (0 to 0.1) | 0.1 (0.1 to 0.3) | 0 (0 to 0.1) | 0 (0 to 0) | 0.1 (0 to 0.1) | -50 (-73.6 to -6.5) | -55.1 (-82.4 to 8.4) | -48.7 (-73 to -2.9) |
|  |  |  | All ages | 0 (0 to 0.1) | 0 (0 to 0) | 0 (0 to 0.1) | 0.2 (0.1 to 0.5) | 0 (0 to 0) | 0.2 (0.1 to 0.4) | 375.5 (104.9 to 984.9) | 250.5 (65.6 to 596.1) | 392.1 (99.1 to 1073.2) |
| Saudi Arabia | All risk factors | DALYs (Disability-Adjusted Life Years) | Age-standardized | 22.1 (10 to 34.4) | 24 (10.9 to 38.7) | 20.4 (9.2 to 33) | 30.6 (16.9 to 45.3) | 34.4 (18.4 to 51) | 27.4 (14.7 to 40.9) | 38.3 (4.2 to 94.1) | 43.1 (-2.1 to 120.6) | 34.2 (3.8 to 92.8) |
|  |  |  | All ages | 996.5 (454.1 to 1546.9) | 479.5 (217.6 to 763) | 517 (242.1 to 830.4) | 3858.3 (2223.9 to 5789.3) | 1650.9 (904.7 to 2449.5) | 2207.4 (1234.4 to 3289.6) | 287.2 (194.4 to 456.8) | 244.3 (128.9 to 447.8) | 327 (229.3 to 518.8) |
|  |  | Deaths | Age-standardized | 1 (0.4 to 1.7) | 1.2 (0.5 to 2) | 0.8 (0.3 to 1.4) | 1.4 (0.8 to 2.1) | 1.8 (1 to 2.9) | 1 (0.5 to 1.6) | 40.2 (-4.7 to 116) | 56.5 (-8 to 170.4) | 25.1 (-19.1 to 110.9) |
|  |  |  | All ages | 34.5 (15.3 to 57.6) | 20.4 (8.9 to 34.6) | 14.1 (5.7 to 24.1) | 103.5 (58.4 to 154.9) | 58.6 (31 to 90.2) | 44.9 (25.6 to 71.9) | 199.4 (100.1 to 367.8) | 186.7 (68.6 to 402.8) | 217.8 (101.8 to 446.2) |
|  | Alcohol use | DALYs (Disability-Adjusted Life Years) | Age-standardized | 0.1 (0 to 0.1) | 0 (0 to 0) | 0.1 (0 to 0.2) | 0 (0 to 0.1) | 0 (0 to 0) | 0.1 (0 to 0.2) | -40 (-75.4 to -5.5) | -45.4 (-82.3 to 1.5) | -43.8 (-77 to -10.4) |
|  |  |  | All ages | 4 (1 to 7.2) | 0.1 (0 to 0.2) | 3.9 (1 to 7) | 8.9 (1.1 to 18.2) | 0.2 (0 to 0.4) | 8.8 (1.1 to 17.9) | 121.6 (-0.1 to 228.7) | 73.2 (-32 to 210.8) | 122.7 (-0.1 to 230.4) |
|  |  | Deaths | Age-standardized | 0 (0 to 0) | 0 (0 to 0) | 0 (0 to 0) | 0 (0 to 0) | 0 (0 to 0) | 0 (0 to 0) | -45.5 (-80.9 to 7.7) | -44.4 (-85 to 19.2) | -51.1 (-83.1 to -0.9) |
|  |  |  | All ages | 0.1 (0 to 0.2) | 0 (0 to 0) | 0.1 (0 to 0.2) | 0.1 (0 to 0.3) | 0 (0 to 0) | 0.1 (0 to 0.3) | 59.5 (-35 to 193.9) | 30.5 (-53.1 to 165.7) | 60.7 (-35.1 to 197.9) |
|  | Diet high in sodium | DALYs (Disability-Adjusted Life Years) | Age-standardized | 0.4 (0 to 2.2) | 0.2 (0 to 1.9) | 0.5 (0 to 2.5) | 0.4 (0 to 2.4) | 0.3 (0 to 2) | 0.5 (0 to 2.7) | 11.5 (-90.5 to 845.3) | 12 (-603.3 to 64122.5) | 6.5 (-90.5 to 834.4) |
|  |  |  | All ages | 17.5 (0 to 103.3) | 5 (0 to 39) | 12.5 (0 to 66.1) | 58 (0 to 333.9) | 14.2 (0 to 104.5) | 43.8 (0 to 222.4) | 230.9 (-59.1 to 2579.4) | 183.1 (-675.5 to 264706.9) | 250.2 (-59.1 to 2581.2) |
|  |  | Deaths | Age-standardized | 0 (0 to 0.1) | 0 (0 to 0.1) | 0 (0 to 0.1) | 0 (0 to 0.1) | 0 (0 to 0.1) | 0 (0 to 0.1) | 8.4 (-93.1 to 900.6) | 18.5 (-438.5 to 49119.6) | -1.8 (-93.7 to 910.4) |
|  |  |  | All ages | 0.5 (0 to 3.4) | 0.2 (0 to 1.6) | 0.3 (0 to 1.8) | 1.3 (0 to 7.9) | 0.4 (0 to 3.4) | 0.8 (0 to 4.6) | 154.1 (-59 to 2831.3) | 134.9 (-615.2 to 261391.8) | 166.1 (-61.9 to 2819) |
|  | High body-mass index | DALYs (Disability-Adjusted Life Years) | Age-standardized | 5.8 (2.3 to 10.1) | 7.3 (2.9 to 13) | 4.6 (1.8 to 8.2) | 15.6 (6.8 to 26.6) | 20.3 (9 to 35) | 12.1 (4.9 to 20.7) | 168.1 (94.9 to 253.2) | 177.2 (84.1 to 279.3) | 161.7 (71.2 to 270.1) |
|  |  |  | All ages | 278.2 (110.8 to 484.2) | 155.3 (61.3 to 273.8) | 122.9 (47.2 to 218) | 2120.2 (971 to 3574.4) | 1082.6 (514.9 to 1827.4) | 1037.7 (449.5 to 1796.8) | 662.1 (463.8 to 903) | 596.9 (368.9 to 860.3) | 744.5 (481.9 to 1046.3) |
|  |  | Deaths | Age-standardized | 0.2 (0.1 to 0.4) | 0.3 (0.1 to 0.6) | 0.2 (0.1 to 0.3) | 0.7 (0.3 to 1.2) | 1 (0.4 to 1.9) | 0.4 (0.2 to 0.8) | 186.5 (82.3 to 330.8) | 221.4 (87.6 to 416.1) | 147.2 (38.6 to 344.5) |
|  |  |  | All ages | 9 (3.7 to 16.3) | 5.8 (2.4 to 11) | 3.2 (1.1 to 6.2) | 55.5 (24.4 to 94.5) | 35.3 (14.8 to 62) | 20.2 (9 to 35.7) | 519.1 (296.1 to 827.4) | 507.9 (250.4 to 850) | 539.6 (272.9 to 981.3) |
|  | High systolic blood pressure | DALYs (Disability-Adjusted Life Years) | Age-standardized | 18.4 (6.4 to 31.7) | 20.1 (6.7 to 35.7) | 16.9 (5.7 to 29.8) | 21.4 (7.1 to 36.6) | 23 (7.2 to 40.7) | 19.9 (6.2 to 34.7) | 16.3 (-9.6 to 50.2) | 14.5 (-23.8 to 69.2) | 17.7 (-9.8 to 65.3) |
|  |  |  | All ages | 806.1 (283.7 to 1412.7) | 394.9 (130.5 to 694.7) | 411.2 (137.3 to 719.5) | 2455.3 (771.1 to 4285.3) | 1009.1 (312.5 to 1784.7) | 1446.1 (468.6 to 2547) | 204.6 (133.8 to 290.8) | 155.5 (69.5 to 264) | 251.7 (164 to 374.6) |
|  |  | Deaths | Age-standardized | 0.8 (0.3 to 1.5) | 1 (0.3 to 1.9) | 0.7 (0.2 to 1.3) | 1 (0.3 to 1.7) | 1.3 (0.4 to 2.3) | 0.7 (0.3 to 1.4) | 18.1 (-19.8 to 68.6) | 27.3 (-26.8 to 104.6) | 10.4 (-30.9 to 85.4) |
|  |  |  | All ages | 29.1 (10.3 to 51.1) | 17.3 (6 to 31.6) | 11.8 (3.6 to 21.3) | 70.2 (23.1 to 117) | 38.5 (12.3 to 69.9) | 31.7 (10.8 to 57.1) | 141.3 (62.3 to 244.9) | 122.3 (30.5 to 253.3) | 169.1 (69.8 to 356.4) |
|  | Lead exposure | DALYs (Disability-Adjusted Life Years) | Age-standardized | 2.3 (-0.3 to 6) | 2 (-0.3 to 5.5) | 2.6 (-0.4 to 6.8) | 2.4 (-0.3 to 6.4) | 2.3 (-0.3 to 6) | 2.5 (-0.3 to 6.6) | 5.3 (-16.9 to 33.6) | 13.6 (-22.1 to 53.8) | -1.9 (-22.8 to 29.1) |
|  |  |  | All ages | 103.1 (-14 to 270.9) | 39.8 (-5.4 to 107.8) | 63.3 (-8.6 to 165.4) | 262.7 (-35.6 to 681.3) | 96.7 (-13.4 to 260.7) | 166 (-22.4 to 440.1) | 154.8 (98.7 to 226.6) | 142.7 (67 to 223.9) | 162.4 (108.6 to 245.2) |
|  |  | Deaths | Age-standardized | 0.1 (0 to 0.3) | 0.1 (0 to 0.3) | 0.1 (0 to 0.3) | 0.1 (0 to 0.3) | 0.1 (0 to 0.3) | 0.1 (0 to 0.3) | 13.2 (-18.6 to 61.7) | 28.6 (-24 to 90.4) | -0.6 (-33.9 to 58.5) |
|  |  |  | All ages | 3.5 (-0.5 to 9.4) | 1.7 (-0.3 to 4.8) | 1.8 (-0.3 to 4.8) | 7.8 (-1.2 to 19.7) | 3.9 (-0.6 to 10.2) | 4 (-0.6 to 10.3) | 121.9 (56.3 to 214.2) | 121.7 (31.8 to 227.2) | 122.1 (44.3 to 257.6) |
|  | Smoking | DALYs (Disability-Adjusted Life Years) | Age-standardized | 1.1 (0.6 to 1.6) | 0.3 (0.1 to 0.5) | 1.6 (0.9 to 2.6) | 1.5 (0.8 to 2.3) | 0.4 (0.2 to 0.6) | 2.3 (1.3 to 3.4) | 42.4 (12.1 to 82.3) | 28.6 (-19.3 to 99.9) | 37.9 (7.1 to 82.3) |
|  |  |  | All ages | 66.5 (35.9 to 106) | 6.8 (3.5 to 11.3) | 59.8 (32.1 to 96) | 370.8 (200.8 to 567.8) | 32.6 (17 to 54.1) | 338.2 (183 to 526.8) | 457.3 (327 to 641.3) | 381.2 (204.8 to 645.5) | 466 (328.1 to 657.5) |
|  |  | Deaths | Age-standardized | 0 (0 to 0) | 0 (0 to 0) | 0 (0 to 0.1) | 0 (0 to 0.1) | 0 (0 to 0) | 0 (0 to 0.1) | 27.2 (-17.9 to 95.6) | 21.5 (-37.2 to 118.9) | 18.4 (-29 to 92) |
|  |  |  | All ages | 1.2 (0.7 to 2) | 0.2 (0.1 to 0.3) | 1 (0.6 to 1.7) | 5.2 (2.7 to 8) | 0.7 (0.4 to 1.1) | 4.5 (2.3 to 7) | 324.5 (164.4 to 562.3) | 278.1 (97.3 to 557.5) | 332.8 (157.1 to 601.3) |
| Somalia | All risk factors | DALYs (Disability-Adjusted Life Years) | Age-standardized | 17.8 (6.3 to 32.2) | 17.6 (6 to 34.1) | 17.9 (6.2 to 31.1) | 20.1 (7.3 to 36.9) | 19.9 (7 to 38.4) | 20 (6.7 to 35.2) | 12.9 (-11.2 to 50.3) | 13 (-16.4 to 64.1) | 11.8 (-16.6 to 44.3) |
|  |  |  | All ages | 308.2 (116.3 to 539.1) | 154.7 (52.3 to 299.9) | 153.5 (61 to 263.4) | 875.7 (320.3 to 1566.2) | 497.4 (176.9 to 936.2) | 378.3 (143.5 to 655) | 184.2 (118.1 to 277.1) | 221.5 (131.4 to 366.6) | 146.5 (76.8 to 229.5) |
|  |  | Deaths | Age-standardized | 0.7 (0.2 to 1.4) | 0.7 (0.2 to 1.6) | 0.7 (0.1 to 1.3) | 0.7 (0.2 to 1.5) | 0.7 (0.2 to 1.7) | 0.7 (0.1 to 1.4) | 2.1 (-28.5 to 57.9) | 1.4 (-35.9 to 72.8) | 0.2 (-33.3 to 46.3) |
|  |  |  | All ages | 8.1 (2.2 to 15.6) | 4.3 (1.2 to 9.7) | 3.8 (0.8 to 7.1) | 19.3 (5.4 to 41.6) | 11.6 (3.1 to 28) | 7.8 (1.5 to 15) | 139 (63.9 to 257) | 168.4 (69.4 to 331.4) | 105.5 (27.5 to 202) |
|  | Alcohol use | DALYs (Disability-Adjusted Life Years) | Age-standardized | 0 (0 to 0) | 0 (0 to 0) | 0 (0 to 0) | 0 (0 to 0) | 0 (0 to 0) | 0 (0 to 0) | NA | NA | NA |
|  |  |  | All ages | 0 (0 to 0) | 0 (0 to 0) | 0 (0 to 0) | 0 (0 to 0) | 0 (0 to 0) | 0 (0 to 0) | NA | NA | NA |
|  |  | Deaths | Age-standardized | 0 (0 to 0) | 0 (0 to 0) | 0 (0 to 0) | 0 (0 to 0) | 0 (0 to 0) | 0 (0 to 0) | NA | NA | NA |
|  |  |  | All ages | 0 (0 to 0) | 0 (0 to 0) | 0 (0 to 0) | 0 (0 to 0) | 0 (0 to 0) | 0 (0 to 0) | NA | NA | NA |
|  | Diet high in sodium | DALYs (Disability-Adjusted Life Years) | Age-standardized | 2.9 (0.1 to 9) | 2.7 (0.1 to 8.7) | 3.1 (0.1 to 9.2) | 2 (0 to 7) | 2.1 (0 to 7.1) | 1.8 (0 to 6.9) | -29.8 (-77.6 to -10) | -21.6 (-69.6 to 15.1) | -39.5 (-92.5 to -14.4) |
|  |  |  | All ages | 45.2 (2.2 to 137.4) | 23.1 (1 to 74.7) | 22.2 (1 to 66.2) | 81.5 (1.9 to 289.8) | 53.1 (1.3 to 175.8) | 28.4 (0.2 to 109.1) | 80.1 (-40.9 to 130.7) | 130.2 (-10.4 to 252.8) | 27.9 (-86.5 to 89.8) |
|  |  | Deaths | Age-standardized | 0.1 (0 to 0.4) | 0.1 (0 to 0.4) | 0.1 (0 to 0.4) | 0.1 (0 to 0.3) | 0.1 (0 to 0.3) | 0.1 (0 to 0.3) | -37.2 (-82 to -11.8) | -31.3 (-78.9 to 2.7) | -44.9 (-93 to -16.1) |
|  |  |  | All ages | 1.3 (0.1 to 4.3) | 0.7 (0 to 2.3) | 0.6 (0 to 2.1) | 1.9 (0 to 7.8) | 1.3 (0 to 5.1) | 0.7 (0 to 2.9) | 50.2 (-55.6 to 115.2) | 90.2 (-35.4 to 214.3) | 8 (-87.2 to 68.1) |
|  | High body-mass index | DALYs (Disability-Adjusted Life Years) | Age-standardized | 0.4 (0.1 to 0.9) | 0.7 (0.2 to 1.5) | 0 (-0.1 to 0.2) | 1.1 (0.3 to 2.3) | 1.6 (0.5 to 3.3) | 0.5 (0.1 to 1.5) | 202.4 (75.9 to 612.6) | 128.5 (31.1 to 405.7) | 2357.7 (-12399.5 to 8422.7) |
|  |  |  | All ages | 11.5 (3.2 to 24.3) | 11.1 (3.2 to 23.4) | 0.4 (-0.5 to 2) | 73.8 (24.7 to 145.2) | 61.4 (20.8 to 124.4) | 12.4 (2.3 to 30.6) | 540.7 (307.5 to 1139.2) | 453.3 (249.5 to 992.8) | 2800.6 (-20655.2 to 27981.2) |
|  |  | Deaths | Age-standardized | 0 (0 to 0) | 0 (0 to 0) | 0 (0 to 0) | 0 (0 to 0.1) | 0 (0 to 0.1) | 0 (0 to 0.1) | 259.8 (-555.6 to 1531.2) | 142.4 (-8.7 to 766.4) | 239443.5 (-10923.8 to 3714.5) |
|  |  |  | All ages | 0.2 (0 to 0.5) | 0.2 (0 to 0.4) | 0 (0 to 0) | 1.1 (0.3 to 2.8) | 0.9 (0.2 to 2.4) | 0.2 (0 to 0.7) | 555.6 (225.4 to 1546.8) | 439.8 (152.8 to 1197.5) | 3821.4 (-12091.8 to 16449.6) |
|  | High systolic blood pressure | DALYs (Disability-Adjusted Life Years) | Age-standardized | 15.7 (4.5 to 30.3) | 16.7 (5 to 33.5) | 14.5 (3.2 to 27.9) | 18.3 (5.6 to 34.6) | 18.8 (6 to 37.3) | 17.3 (4.9 to 31.5) | 16.4 (-8.9 to 57.8) | 12.2 (-18.4 to 64.6) | 19.1 (-12.6 to 66.8) |
|  |  |  | All ages | 258.6 (73.9 to 490.3) | 142.6 (41.8 to 285) | 116 (27.9 to 222.3) | 761.3 (221.6 to 1436.5) | 454.3 (135.5 to 884.7) | 307 (80.3 to 582.9) | 194.4 (123.7 to 299.1) | 218.6 (124.8 to 382) | 164.6 (87 to 279.3) |
|  |  | Deaths | Age-standardized | 0.6 (0.1 to 1.3) | 0.7 (0.2 to 1.6) | 0.6 (0.1 to 1.2) | 0.7 (0.2 to 1.5) | 0.7 (0.2 to 1.7) | 0.6 (0.1 to 1.2) | 5.1 (-26.9 to 60.4) | 1.2 (-36.4 to 75.6) | 5.8 (-30.3 to 64.5) |
|  |  |  | All ages | 7.1 (1.6 to 14.7) | 4.1 (1 to 9.5) | 3 (0.5 to 6.1) | 17.5 (3.9 to 39.1) | 10.9 (2.7 to 27) | 6.6 (1 to 13.4) | 145.5 (71 to 275.3) | 166.8 (66.3 to 339.2) | 116.7 (33.6 to 231) |
|  | Lead exposure | DALYs (Disability-Adjusted Life Years) | Age-standardized | 3.9 (-0.7 to 10.9) | 2.9 (-0.5 to 8) | 5.2 (-0.8 to 13.9) | 3.7 (-0.6 to 9.9) | 3.1 (-0.5 to 8.6) | 4.8 (-0.7 to 12) | -4.2 (-20.7 to 17.8) | 9.3 (-15 to 41.3) | -7.3 (-27.7 to 14.3) |
|  |  |  | All ages | 62.2 (-10.1 to 175.3) | 23.9 (-3.9 to 64.7) | 38.3 (-5.8 to 102.7) | 152.1 (-24.1 to 397) | 73.6 (-11.8 to 199) | 78.5 (-12.1 to 200.6) | 144.6 (101.7 to 199.6) | 208 (145.2 to 288.8) | 105.1 (59.1 to 158.1) |
|  |  | Deaths | Age-standardized | 0.2 (0 to 0.5) | 0.1 (0 to 0.4) | 0.2 (0 to 0.6) | 0.1 (0 to 0.4) | 0.1 (0 to 0.3) | 0.2 (0 to 0.5) | -15.4 (-37.3 to 15.8) | -2.4 (-33.9 to 42.3) | -19.2 (-43.9 to 12.3) |
|  |  |  | All ages | 1.8 (-0.3 to 4.8) | 0.7 (-0.1 to 2.1) | 1.1 (-0.2 to 3) | 3.5 (-0.6 to 9.5) | 1.8 (-0.3 to 5.1) | 1.8 (-0.3 to 5) | 100.7 (42.5 to 183.4) | 156.9 (69.3 to 286.9) | 64.3 (9.9 to 133.7) |
|  | Smoking | DALYs (Disability-Adjusted Life Years) | Age-standardized | 2.4 (1.2 to 3.9) | 0.7 (0.3 to 1.3) | 4.3 (2.1 to 7.3) | 1.7 (0.8 to 2.9) | 0.5 (0.2 to 1) | 3.3 (1.6 to 5.9) | -29.7 (-48 to -5.9) | -26.8 (-54.5 to 19.8) | -21.4 (-41.9 to 8.8) |
|  |  |  | All ages | 54.2 (26.9 to 90.6) | 7.8 (3.6 to 14.9) | 46.4 (22.5 to 80.5) | 102.5 (48.3 to 184.3) | 15.9 (6.9 to 31.7) | 86.6 (40.3 to 157) | 89 (40.6 to 153.8) | 102.7 (23.5 to 216.5) | 86.7 (37.7 to 154.8) |
|  |  | Deaths | Age-standardized | 0.1 (0 to 0.1) | 0 (0 to 0) | 0.1 (0 to 0.2) | 0 (0 to 0.1) | 0 (0 to 0) | 0.1 (0 to 0.2) | -41.1 (-61.9 to -10.3) | -34.9 (-66.6 to 33.5) | -32.4 (-56.7 to 2.2) |
|  |  |  | All ages | 1.1 (0.4 to 1.9) | 0.2 (0.1 to 0.4) | 0.9 (0.3 to 1.7) | 1.7 (0.5 to 3.6) | 0.3 (0.1 to 0.7) | 1.4 (0.4 to 3.1) | 58.7 (-1 to 143) | 73.9 (-4.6 to 212.8) | 56.1 (-3.9 to 151.3) |
| Sudan | All risk factors | DALYs (Disability-Adjusted Life Years) | Age-standardized | 24.4 (11.1 to 40.9) | 27.3 (12.2 to 47.6) | 21.7 (9.1 to 37.8) | 31.1 (15.7 to 48) | 35.5 (17 to 55.1) | 27.6 (14 to 43.9) | 27.4 (-0.8 to 70.4) | 29.7 (-5.2 to 79.6) | 27.4 (-2.7 to 78.3) |
|  |  |  | All ages | 1794 (808.9 to 2953.3) | 973 (432.4 to 1658.3) | 820.9 (357 to 1384.7) | 4753.6 (2448.6 to 7311.5) | 2434.5 (1180.3 to 3763.2) | 2319.1 (1202.3 to 3638.4) | 165 (104.5 to 251) | 150.2 (81.4 to 249.9) | 182.5 (115 to 292.6) |
|  |  | Deaths | Age-standardized | 1.1 (0.4 to 1.9) | 1.3 (0.5 to 2.6) | 0.8 (0.2 to 1.7) | 1.4 (0.6 to 2.2) | 1.7 (0.8 to 2.7) | 1.1 (0.5 to 1.8) | 29.1 (-10.2 to 103.9) | 35.4 (-14.2 to 109.7) | 26.9 (-21.4 to 141.7) |
|  |  |  | All ages | 58.8 (22.3 to 108.7) | 36.3 (14.2 to 72.3) | 22.5 (6.8 to 44.3) | 168 (81.6 to 269.4) | 98.2 (44.8 to 156.8) | 69.8 (31.2 to 118) | 185.9 (97.3 to 356) | 170.9 (72.9 to 315.6) | 210 (91.8 to 499.6) |
|  | Alcohol use | DALYs (Disability-Adjusted Life Years) | Age-standardized | 0.6 (0.3 to 0.9) | 0.1 (0 to 0.1) | 1.1 (0.6 to 1.7) | 0 (0 to 0) | 0 (0 to 0) | 0 (0 to 0) | -100 (-100 to -99.9) | -100 (-100 to -99.8) | -100 (-100 to -99.9) |
|  |  |  | All ages | 50.5 (28.8 to 75.2) | 2.4 (1.4 to 4) | 48.1 (27.2 to 72.4) | 0 (0 to 0.1) | 0 (0 to 0) | 0 (0 to 0.1) | -100 (-100 to -99.8) | -99.9 (-100 to -99.5) | -100 (-100 to -99.8) |
|  |  | Deaths | Age-standardized | 0 (0 to 0) | 0 (0 to 0) | 0 (0 to 0.1) | 0 (0 to 0) | 0 (0 to 0) | 0 (0 to 0) | -100 (-100 to -100) | -100 (-100 to -99.8) | -100 (-100 to -100) |
|  |  |  | All ages | 1.2 (0.5 to 2) | 0.1 (0 to 0.1) | 1.1 (0.5 to 1.9) | 0 (0 to 0) | 0 (0 to 0) | 0 (0 to 0) | -100 (-100 to -99.9) | -100 (-100 to -99.5) | -100 (-100 to -99.9) |
|  | Diet high in sodium | DALYs (Disability-Adjusted Life Years) | Age-standardized | 0.4 (0 to 2.1) | 0.2 (0 to 1.7) | 0.5 (0 to 2.6) | 0.4 (0 to 2.4) | 0.2 (0 to 1.8) | 0.5 (0 to 2.8) | 10.4 (-94.4 to 1038.5) | 5.8 (-3401.4 to 19135.2) | 8.3 (-95.8 to 943.2) |
|  |  |  | All ages | 29.4 (0 to 163.5) | 9.1 (0 to 65.5) | 20.3 (0 to 100.9) | 65.2 (0 to 385.7) | 18.1 (0 to 130.9) | 47.1 (0 to 245) | 121.7 (-84 to 2066.7) | 99.3 (-873.7 to 634155.6) | 131.7 (-82 to 2044.8) |
|  |  | Deaths | Age-standardized | 0 (0 to 0.1) | 0 (0 to 0.1) | 0 (0 to 0.1) | 0 (0 to 0.1) | 0 (0 to 0.1) | 0 (0 to 0.1) | 9.8 (-96.7 to 1583.8) | 8.1 (-7889.3 to 36259.2) | 6 (-96.8 to 1470.9) |
|  |  |  | All ages | 0.8 (0 to 4.8) | 0.3 (0 to 2.3) | 0.5 (0 to 2.9) | 1.8 (0 to 12) | 0.6 (0 to 4.6) | 1.2 (0 to 7.2) | 126.5 (-93.1 to 2608.5) | 103.8 (-4851.8 to 38714.1) | 140.2 (-93.2 to 2665.6) |
|  | High body-mass index | DALYs (Disability-Adjusted Life Years) | Age-standardized | 3.7 (1.4 to 6.8) | 5.9 (2.2 to 10.7) | 1.6 (0.5 to 3.3) | 9.3 (3.6 to 15.9) | 11.8 (4.6 to 20.9) | 7.3 (2.7 to 12.8) | 151 (71.3 to 270.9) | 100 (15.7 to 208.7) | 344.3 (159.2 to 1005.5) |
|  |  |  | All ages | 293.1 (111.8 to 523.7) | 225.3 (85 to 411.2) | 67.8 (21.9 to 134.7) | 1498.9 (594 to 2601) | 869.6 (340.5 to 1536.7) | 629.4 (238.4 to 1087.3) | 411.4 (248.9 to 613.9) | 286 (131.6 to 472.6) | 828.4 (481.5 to 1954.1) |
|  |  | Deaths | Age-standardized | 0.2 (0.1 to 0.3) | 0.2 (0.1 to 0.5) | 0.1 (0 to 0.1) | 0.4 (0.1 to 0.7) | 0.5 (0.2 to 0.9) | 0.3 (0.1 to 0.5) | 154.2 (42.5 to 351.4) | 111.2 (-4.2 to 291.5) | 393.6 (118.4 to 1817.7) |
|  |  |  | All ages | 9.2 (3.3 to 18.4) | 7.6 (2.7 to 15.9) | 1.6 (0.4 to 3.7) | 49 (18.1 to 85.9) | 31.2 (11.5 to 56.6) | 17.9 (6.7 to 33.2) | 432.7 (214.7 to 765.8) | 310.8 (110.7 to 636.7) | 1003.8 (453.1 to 3137.4) |
|  | High systolic blood pressure | DALYs (Disability-Adjusted Life Years) | Age-standardized | 21.1 (6.8 to 37.8) | 24.3 (8.2 to 44.4) | 18.1 (5.2 to 34.5) | 25.8 (8.7 to 43.7) | 29.9 (10.1 to 49) | 22.4 (7.1 to 38.7) | 22.2 (-5.2 to 62.7) | 23 (-11.4 to 69.8) | 24.1 (-6.1 to 76) |
|  |  |  | All ages | 1522.2 (479 to 2685.4) | 856.3 (288.7 to 1543.6) | 665.9 (197.6 to 1258.5) | 3864.6 (1326.2 to 6536.8) | 2016.2 (677.5 to 3374) | 1848.4 (600.2 to 3206.8) | 153.9 (95.5 to 237.2) | 135.5 (69.7 to 228.1) | 177.6 (112.9 to 290.3) |
|  |  | Deaths | Age-standardized | 0.9 (0.3 to 1.8) | 1.1 (0.3 to 2.4) | 0.7 (0.2 to 1.5) | 1.1 (0.4 to 1.9) | 1.5 (0.5 to 2.5) | 0.9 (0.3 to 1.6) | 23.3 (-13.3 to 89.2) | 29.1 (-18.2 to 98.9) | 21.7 (-23.7 to 143.7) |
|  |  |  | All ages | 51.4 (14.6 to 100.1) | 32.4 (9.7 to 66.3) | 19 (4.3 to 40) | 141 (49.5 to 239.6) | 83.6 (27.4 to 142.2) | 57.4 (18.7 to 105.9) | 174.1 (89.7 to 329.1) | 158 (63.3 to 301.5) | 201.6 (91.4 to 493.6) |
|  | Lead exposure | DALYs (Disability-Adjusted Life Years) | Age-standardized | 3.2 (-0.5 to 8.3) | 2.8 (-0.4 to 7.3) | 3.5 (-0.5 to 9.3) | 3.5 (-0.5 to 8.8) | 3.1 (-0.5 to 8) | 3.9 (-0.5 to 9.9) | 12.5 (-11.8 to 49.4) | 10.5 (-24.3 to 60.2) | 11.8 (-14.2 to 53.3) |
|  |  |  | All ages | 228.3 (-35.6 to 596.2) | 101 (-14.4 to 254.5) | 127.3 (-19.1 to 345.4) | 520.1 (-74.6 to 1305.6) | 210.1 (-32.6 to 533.1) | 310 (-42 to 794.6) | 127.8 (78.8 to 200.5) | 108 (45.5 to 190) | 143.5 (88.1 to 227.7) |
|  |  | Deaths | Age-standardized | 0.1 (0 to 0.4) | 0.1 (0 to 0.4) | 0.1 (0 to 0.4) | 0.2 (0 to 0.4) | 0.2 (0 to 0.4) | 0.2 (0 to 0.4) | 19.2 (-17.2 to 95) | 21.4 (-29.3 to 105.4) | 15.7 (-23.7 to 116.1) |
|  |  |  | All ages | 7.4 (-1.3 to 19.6) | 3.7 (-0.5 to 10.2) | 3.7 (-0.6 to 11.4) | 19.2 (-3 to 50.7) | 8.9 (-1.4 to 22.8) | 10.3 (-1.6 to 28.7) | 161 (80.2 to 327.2) | 139.3 (42.7 to 288.4) | 183.1 (84.6 to 437) |
|  | Smoking | DALYs (Disability-Adjusted Life Years) | Age-standardized | 1.8 (1 to 2.9) | 0.5 (0.2 to 0.8) | 3.1 (1.6 to 5.1) | 1.7 (0.9 to 2.7) | 0.4 (0.2 to 0.7) | 2.8 (1.5 to 4.4) | -4.8 (-26.6 to 32.3) | -20.3 (-52.4 to 31.5) | -7.3 (-30 to 32.1) |
|  |  |  | All ages | 150.7 (83.9 to 240.4) | 17.8 (8.9 to 31.4) | 132.8 (70.3 to 218) | 301.9 (162.6 to 478.5) | 28.8 (15.2 to 49.4) | 273.1 (142 to 433.1) | 100.4 (52.9 to 172.1) | 61.5 (1.7 to 159.5) | 105.6 (54.4 to 183) |
|  |  | Deaths | Age-standardized | 0.1 (0 to 0.1) | 0 (0 to 0) | 0.1 (0 to 0.2) | 0.1 (0 to 0.1) | 0 (0 to 0) | 0.1 (0 to 0.1) | -2.1 (-36.7 to 79.4) | -13.4 (-59.3 to 84.1) | -7.2 (-43 to 86.4) |
|  |  |  | All ages | 3.4 (1.5 to 6.2) | 0.6 (0.3 to 1.1) | 2.9 (1.1 to 5.3) | 7.2 (3.8 to 11.8) | 1 (0.4 to 1.9) | 6.2 (3.1 to 10.2) | 108.2 (32.3 to 272.9) | 73.6 (-8.5 to 230.7) | 115 (30.1 to 321.2) |
| Syrian Arab Republic | All risk factors | DALYs (Disability-Adjusted Life Years) | Age-standardized | 30 (15.1 to 47.3) | 35.3 (16.8 to 59.3) | 25.5 (12.8 to 40.2) | 37 (20.8 to 55.9) | 48.8 (24.1 to 78.8) | 31.7 (18 to 47.4) | 23.4 (-8 to 72) | 38.1 (-4.6 to 101.5) | 24.5 (-2.9 to 79.9) |
|  |  |  | All ages | 1235.3 (628 to 1915.3) | 659.6 (320.3 to 1075.2) | 575.7 (290.8 to 889.4) | 3614.1 (2086.1 to 5298.1) | 1901 (967.7 to 2923.6) | 1713.1 (1004.3 to 2515) | 192.6 (123 to 309) | 188.2 (103.2 to 325.2) | 197.6 (132.3 to 318.4) |
|  |  | Deaths | Age-standardized | 1.5 (0.7 to 2.5) | 2.1 (0.9 to 3.6) | 1.1 (0.5 to 1.8) | 1.9 (0.9 to 3.1) | 3.2 (1.4 to 5.4) | 1.4 (0.7 to 2.1) | 24.4 (-20.7 to 101.2) | 53.4 (-7.2 to 149.8) | 27.9 (-17.9 to 143.7) |
|  |  |  | All ages | 55.2 (24.9 to 88.9) | 34.2 (15.4 to 59.1) | 21 (9.1 to 34.7) | 137.4 (69.9 to 220.3) | 81.9 (35.8 to 137.4) | 55.5 (30.1 to 85.8) | 149 (62.9 to 293.9) | 139.6 (48.7 to 299.3) | 164.4 (76.1 to 381.8) |
|  | Alcohol use | DALYs (Disability-Adjusted Life Years) | Age-standardized | 0.4 (0.2 to 0.5) | 0.1 (0 to 0.1) | 0.6 (0.4 to 0.9) | 0.2 (0.1 to 0.3) | 0 (0 to 0.1) | 0.3 (0.2 to 0.6) | -44.6 (-69.3 to -13.3) | -51 (-82.5 to -1) | -45.6 (-68.9 to -15.9) |
|  |  |  | All ages | 17.1 (10 to 24.9) | 1.5 (0.6 to 2.6) | 15.6 (9.1 to 22.7) | 23 (10.5 to 37.5) | 1.6 (0.4 to 3.6) | 21.4 (9.8 to 34.9) | 35.1 (-21.4 to 103.9) | 12.5 (-57.5 to 113.4) | 37.2 (-19.9 to 106) |
|  |  | Deaths | Age-standardized | 0 (0 to 0) | 0 (0 to 0) | 0 (0 to 0) | 0 (0 to 0) | 0 (0 to 0) | 0 (0 to 0) | -41.8 (-73.8 to 22.4) | -45.4 (-83.9 to 30.3) | -48.2 (-76.1 to 12.8) |
|  |  |  | All ages | 0.5 (0.3 to 0.8) | 0.1 (0 to 0.1) | 0.5 (0.2 to 0.7) | 0.6 (0.2 to 1.1) | 0.1 (0 to 0.1) | 0.5 (0.2 to 0.9) | 12.3 (-46 to 119.7) | -4.8 (-67.6 to 101.5) | 14.5 (-45.8 to 131.6) |
|  | Diet high in sodium | DALYs (Disability-Adjusted Life Years) | Age-standardized | 0.4 (0 to 2.5) | 0.3 (0 to 2.4) | 0.5 (0 to 2.8) | 0.5 (0 to 2.9) | 0.3 (0 to 2.7) | 0.6 (0 to 3.2) | 6.9 (-92.4 to 619.9) | 10.8 (-1751.9 to 9967.8) | 5.9 (-91.4 to 696) |
|  |  |  | All ages | 18.3 (0 to 103.2) | 6 (0 to 45.3) | 12.3 (0 to 63.6) | 47.6 (0 to 287.1) | 15.5 (0 to 115.5) | 32.1 (0 to 173.7) | 160.4 (-86.2 to 1236.4) | 158 (-12100.4 to 6617.1) | 161.5 (-86.4 to 1269.5) |
|  |  | Deaths | Age-standardized | 0 (0 to 0.1) | 0 (0 to 0.1) | 0 (0 to 0.1) | 0 (0 to 0.1) | 0 (0 to 0.2) | 0 (0 to 0.1) | 13.5 (-97 to 1032.4) | 23.9 (-1252.5 to 16292.3) | 8.8 (-96.3 to 1061.4) |
|  |  |  | All ages | 0.7 (0 to 4.3) | 0.3 (0 to 2.2) | 0.4 (0 to 2.3) | 1.5 (0 to 10.1) | 0.6 (0 to 4.8) | 0.9 (0 to 5.7) | 132.1 (-93.7 to 2744.4) | 124.3 (-1033.4 to 19988.8) | 137.4 (-93.7 to 2741.1) |
|  | High body-mass index | DALYs (Disability-Adjusted Life Years) | Age-standardized | 6.8 (2.7 to 12.6) | 10.8 (4.3 to 20.8) | 3.4 (1.3 to 6.5) | 17.3 (7.3 to 30.4) | 25.1 (10 to 43.3) | 13.2 (5.4 to 23.9) | 153.8 (70.4 to 261.5) | 132.3 (54.5 to 240.9) | 287.7 (118.2 to 540.1) |
|  |  |  | All ages | 291 (117.8 to 534.1) | 209.9 (84.7 to 393) | 81.1 (30.7 to 150.3) | 1733.8 (740.6 to 3027.1) | 1015.6 (421.7 to 1718) | 718.2 (298.7 to 1296.2) | 495.8 (311.7 to 719.5) | 383.8 (231 to 583.6) | 785.5 (426.7 to 1237.8) |
|  |  | Deaths | Age-standardized | 0.3 (0.1 to 0.7) | 0.6 (0.2 to 1.2) | 0.1 (0 to 0.3) | 0.9 (0.3 to 1.6) | 1.6 (0.6 to 2.9) | 0.6 (0.2 to 1.1) | 156.6 (50.1 to 329.1) | 167.7 (53.3 to 355.5) | 348.1 (83.9 to 996.4) |
|  |  |  | All ages | 12.6 (4.9 to 23.6) | 10 (3.7 to 19.7) | 2.6 (0.8 to 5.3) | 64.5 (25.8 to 116.5) | 41.7 (16.3 to 73.3) | 22.8 (9 to 42.8) | 412.4 (205.5 to 720.4) | 315.8 (138.9 to 587.6) | 790.1 (296.4 to 1804.7) |
|  | High systolic blood pressure | DALYs (Disability-Adjusted Life Years) | Age-standardized | 23.6 (7.7 to 41.5) | 29.1 (9.4 to 52.6) | 19 (5.7 to 34.3) | 25.9 (8.5 to 44.4) | 35.3 (11 to 63.5) | 21.4 (6.9 to 37.3) | 9.6 (-19.6 to 48.8) | 21.2 (-19.1 to 81.7) | 12.7 (-13.3 to 61.6) |
|  |  |  | All ages | 951.9 (316.5 to 1652.3) | 535.6 (174.4 to 961.8) | 416.3 (126.3 to 749.7) | 2472.7 (813.5 to 4237) | 1347.5 (419.8 to 2421.5) | 1125.1 (374.4 to 1963.8) | 159.8 (96.4 to 246.7) | 151.6 (74.1 to 269.1) | 170.3 (109.6 to 275) |
|  |  | Deaths | Age-standardized | 1.3 (0.4 to 2.2) | 1.8 (0.6 to 3.3) | 0.8 (0.2 to 1.5) | 1.4 (0.4 to 2.4) | 2.4 (0.7 to 4.6) | 1 (0.3 to 1.7) | 9.2 (-31.1 to 74.5) | 34.2 (-22.8 to 124.4) | 14 (-27.6 to 109.7) |
|  |  |  | All ages | 44.8 (14.7 to 79.5) | 28.5 (9.2 to 52.7) | 16.3 (4.6 to 30.2) | 98.1 (32.6 to 177.3) | 59.7 (18.3 to 111.4) | 38.3 (12.2 to 68.5) | 119.1 (41 to 236.3) | 109.7 (22.7 to 245.6) | 135.6 (51.5 to 325.6) |
|  | Lead exposure | DALYs (Disability-Adjusted Life Years) | Age-standardized | 3.4 (-0.4 to 8.7) | 3.5 (-0.5 to 9.1) | 3.3 (-0.4 to 8.6) | 3.6 (-0.5 to 9.2) | 4.2 (-0.6 to 10.8) | 3.5 (-0.5 to 8.9) | 8 (-17.9 to 41.4) | 21.9 (-18.6 to 68.7) | 6.7 (-15.4 to 47.4) |
|  |  |  | All ages | 137.9 (-17.7 to 358.4) | 64.7 (-8.7 to 171.1) | 73.2 (-9.6 to 188.3) | 323.8 (-42.6 to 823) | 147.4 (-19.3 to 380.7) | 176.4 (-23.7 to 436.4) | 134.8 (80.1 to 200.5) | 127.8 (57.7 to 214.8) | 140.9 (90.3 to 224.2) |
|  |  | Deaths | Age-standardized | 0.2 (0 to 0.5) | 0.2 (0 to 0.5) | 0.1 (0 to 0.4) | 0.2 (0 to 0.5) | 0.3 (0 to 0.8) | 0.2 (0 to 0.5) | 17 (-23.3 to 79.5) | 43.9 (-13.4 to 121.7) | 15.9 (-25.8 to 108.8) |
|  |  |  | All ages | 6.3 (-0.9 to 16.3) | 3.4 (-0.5 to 9) | 2.9 (-0.4 to 8) | 13.4 (-2 to 35.1) | 6.8 (-1 to 18.1) | 6.5 (-1 to 17.3) | 113.1 (40 to 220.5) | 101.5 (18.5 to 213) | 126.6 (53.3 to 306.1) |
|  | Smoking | DALYs (Disability-Adjusted Life Years) | Age-standardized | 3.9 (2.1 to 6.1) | 1.2 (0.6 to 2.2) | 6.3 (3.4 to 9.8) | 3.3 (1.8 to 5.1) | 1 (0.5 to 1.7) | 5.3 (2.9 to 8.1) | -15.3 (-33.3 to 14) | -20.4 (-53.9 to 36.6) | -15.8 (-33.9 to 13.8) |
|  |  |  | All ages | 184.5 (100.8 to 285.8) | 27.8 (14 to 47.6) | 156.8 (87 to 243.6) | 382.6 (211.1 to 584.5) | 50.8 (26.5 to 84.3) | 331.8 (185.1 to 500.9) | 107.3 (62.4 to 173.8) | 82.9 (15.7 to 195.1) | 111.6 (62.7 to 179.7) |
|  |  | Deaths | Age-standardized | 0.1 (0.1 to 0.2) | 0.1 (0 to 0.1) | 0.2 (0.1 to 0.3) | 0.1 (0.1 to 0.2) | 0.1 (0 to 0.1) | 0.2 (0.1 to 0.3) | -10.9 (-43.4 to 54.3) | -10.8 (-61 to 93.8) | -18.3 (-46.7 to 47.9) |
|  |  |  | All ages | 5.5 (2.7 to 8.7) | 1 (0.5 to 1.9) | 4.4 (2.2 to 7.1) | 9.7 (5.1 to 15.7) | 1.6 (0.7 to 3) | 8.1 (4.3 to 12.8) | 77.4 (20.6 to 193.7) | 54.8 (-17.2 to 188.4) | 82.6 (21.8 to 221.1) |
| Tunisia | All risk factors | DALYs (Disability-Adjusted Life Years) | Age-standardized | 23.6 (10.9 to 36.8) | 24.3 (9.6 to 40) | 22.6 (11.1 to 36.1) | 30.2 (16.2 to 48.7) | 29.3 (13.8 to 49.7) | 31.6 (17.6 to 50.3) | 27.9 (-3.6 to 82.8) | 20.8 (-17.9 to 85.1) | 39.7 (-2.3 to 105.2) |
|  |  |  | All ages | 901.8 (441.3 to 1383.3) | 450.7 (191.6 to 732.2) | 451.1 (225.6 to 704) | 3476.5 (1862.5 to 5473.2) | 1851.7 (877 to 3134.9) | 1624.8 (920.9 to 2520.1) | 285.5 (196.4 to 423.9) | 310.9 (181.5 to 503.8) | 260.2 (172.8 to 401.6) |
|  |  | Deaths | Age-standardized | 1.1 (0.5 to 1.8) | 1.2 (0.4 to 2.2) | 0.9 (0.4 to 1.7) | 1.6 (0.8 to 2.7) | 1.6 (0.7 to 3) | 1.6 (0.8 to 2.8) | 44.3 (-8.4 to 140) | 31.8 (-23 to 128.2) | 74.8 (-6.3 to 239.8) |
|  |  |  | All ages | 31.1 (12.8 to 49.7) | 17.8 (6.5 to 30.3) | 13.3 (5.7 to 23.5) | 164.4 (82.2 to 271.2) | 97.3 (39.9 to 177.7) | 67.2 (35.2 to 112.6) | 428.3 (244.2 to 736.8) | 445.3 (213.6 to 817.9) | 405.5 (198.1 to 786.5) |
|  | Alcohol use | DALYs (Disability-Adjusted Life Years) | Age-standardized | 0.3 (0.2 to 0.4) | 0 (0 to 0) | 0.5 (0.3 to 0.8) | 0.4 (0.3 to 0.6) | 0 (0 to 0) | 0.9 (0.5 to 1.2) | 46.2 (7.2 to 99.9) | 19.1 (-41.9 to 158.9) | 58.9 (13 to 124.3) |
|  |  |  | All ages | 12.5 (7.4 to 17.6) | 0.1 (0 to 0.2) | 12.4 (7.3 to 17.5) | 49.4 (31.5 to 70) | 0.3 (0.1 to 0.6) | 49 (31.2 to 69.4) | 296 (198 to 426.1) | 286.2 (87.3 to 675.4) | 296.1 (198.1 to 427.9) |
|  |  | Deaths | Age-standardized | 0 (0 to 0) | 0 (0 to 0) | 0 (0 to 0) | 0 (0 to 0) | 0 (0 to 0) | 0 (0 to 0.1) | 78.8 (-0.2 to 228.6) | 22.2 (-55.1 to 232.3) | 99.5 (5.9 to 284.5) |
|  |  |  | All ages | 0.3 (0.1 to 0.4) | 0 (0 to 0) | 0.3 (0.1 to 0.4) | 1.5 (0.8 to 2.5) | 0 (0 to 0) | 1.5 (0.8 to 2.5) | 434.5 (213.2 to 794) | 382.1 (89.6 to 1079) | 435.1 (211.6 to 795) |
|  | Diet high in sodium | DALYs (Disability-Adjusted Life Years) | Age-standardized | 0.4 (0 to 2.3) | 0.2 (0 to 1.8) | 0.5 (0 to 2.9) | 0.4 (0 to 2.4) | 0.3 (0 to 2) | 0.6 (0 to 3.3) | 7.8 (-88.5 to 961.5) | 4.3 (-152198.1 to 7227) | 16.5 (-87.9 to 1172.1) |
|  |  |  | All ages | 15.7 (0 to 91.1) | 4.9 (0 to 34.4) | 10.8 (0 to 58) | 48.3 (0 to 293.5) | 16.5 (0 to 124.8) | 31.8 (0 to 171.4) | 208.4 (-76.6 to 2263.8) | 239.1 (-1061119.6 to 12947.8) | 194.5 (-77.7 to 2586.4) |
|  |  | Deaths | Age-standardized | 0 (0 to 0.1) | 0 (0 to 0.1) | 0 (0 to 0.1) | 0 (0 to 0.1) | 0 (0 to 0.1) | 0 (0 to 0.2) | 20.3 (-93.3 to 1995.9) | 7.9 (-113755.7 to 6558.4) | 40.4 (-93 to 3140.3) |
|  |  |  | All ages | 0.5 (0 to 2.9) | 0.2 (0 to 1.4) | 0.3 (0 to 1.7) | 1.8 (0 to 13.1) | 0.7 (0 to 6) | 1.1 (0 to 6.7) | 292.5 (-84.1 to 3398.4) | 313 (-417168.4 to 13290.3) | 280 (-85.2 to 4931.9) |
|  | High body-mass index | DALYs (Disability-Adjusted Life Years) | Age-standardized | 2.6 (1 to 5.1) | 4 (1.6 to 7.9) | 1.3 (0.4 to 2.6) | 10.3 (4.1 to 18.5) | 11.5 (4.8 to 21.9) | 9.1 (3.6 to 16.7) | 292.9 (138.3 to 479.1) | 186.7 (74.5 to 331.1) | 625.8 (215.7 to 1433.5) |
|  |  |  | All ages | 117.3 (44 to 213.9) | 88.9 (34.7 to 169.5) | 28.5 (8.9 to 56.6) | 1195.1 (478.9 to 2093) | 740.3 (307.8 to 1399) | 454.9 (179.1 to 806.7) | 918.7 (603.6 to 1334.4) | 733 (459.8 to 1104.3) | 1498.3 (686.6 to 2869.9) |
|  |  | Deaths | Age-standardized | 0.1 (0 to 0.2) | 0.2 (0.1 to 0.4) | 0 (0 to 0.1) | 0.5 (0.2 to 1.1) | 0.6 (0.2 to 1.2) | 0.5 (0.2 to 1) | 447 (165.7 to 944.7) | 293.5 (78.7 to 664.4) | 1094.4 (261.5 to 3668.3) |
|  |  |  | All ages | 3.4 (1.3 to 6.9) | 2.7 (1 to 5.8) | 0.7 (0.2 to 1.6) | 55.5 (21.3 to 105.3) | 35.5 (13.1 to 74.9) | 20 (7.6 to 39.2) | 1544.7 (837.8 to 2578.1) | 1224.2 (590.7 to 2194.8) | 2787.4 (906.5 to 7081.9) |
|  | High systolic blood pressure | DALYs (Disability-Adjusted Life Years) | Age-standardized | 19.2 (6.5 to 32.5) | 21.8 (7.3 to 37.3) | 16.2 (4.7 to 29.6) | 21.8 (7.1 to 39.1) | 22.9 (7.1 to 43.2) | 20.7 (6.5 to 38.6) | 13.5 (-17.2 to 57.8) | 4.9 (-30.3 to 57.7) | 27.9 (-11.4 to 94) |
|  |  |  | All ages | 712.7 (239.6 to 1215.5) | 397.5 (136.4 to 685.5) | 315.2 (91.5 to 565.3) | 2494 (811.3 to 4470.4) | 1438 (442.8 to 2695.2) | 1055.9 (333.3 to 1941.8) | 249.9 (163.3 to 373.5) | 261.8 (142.2 to 429.3) | 235 (144.2 to 382.8) |
|  |  | Deaths | Age-standardized | 1 (0.3 to 1.7) | 1.1 (0.3 to 2.1) | 0.7 (0.2 to 1.4) | 1.2 (0.3 to 2.2) | 1.3 (0.3 to 2.6) | 1.1 (0.3 to 2.2) | 25.5 (-22 to 98.7) | 14.2 (-36.7 to 89.5) | 56.4 (-18.8 to 218.1) |
|  |  |  | All ages | 26 (8 to 44.6) | 16.2 (4.8 to 28.8) | 9.8 (2.5 to 19.8) | 122.4 (35.7 to 228.1) | 77.5 (20.7 to 153) | 44.8 (12.9 to 90.9) | 371.1 (205.7 to 612.6) | 378.6 (162.4 to 672.1) | 358.6 (162.9 to 725.8) |
|  | Lead exposure | DALYs (Disability-Adjusted Life Years) | Age-standardized | 2.5 (-0.3 to 6.6) | 2.3 (-0.3 to 6.1) | 2.7 (-0.4 to 7.4) | 2.9 (-0.4 to 7.2) | 2.6 (-0.3 to 6.8) | 3.4 (-0.4 to 8.6) | 14.6 (-12.7 to 47.9) | 10.1 (-24.7 to 47.8) | 25.3 (-9.8 to 82.4) |
|  |  |  | All ages | 93 (-11.9 to 233.7) | 41.5 (-5.8 to 108) | 51.5 (-6.4 to 137) | 323.4 (-43.2 to 806.7) | 160.1 (-21.3 to 418.9) | 163.3 (-20.7 to 415.7) | 247.8 (171.8 to 347.8) | 285.8 (168.8 to 415.9) | 217.2 (142.8 to 320.7) |
|  |  | Deaths | Age-standardized | 0.1 (0 to 0.3) | 0.1 (0 to 0.3) | 0.1 (0 to 0.4) | 0.2 (0 to 0.4) | 0.2 (0 to 0.4) | 0.2 (0 to 0.5) | 35.8 (-13.5 to 98.8) | 23.7 (-25.9 to 80.6) | 65.8 (-6.2 to 219.3) |
|  |  |  | All ages | 3.3 (-0.5 to 8.9) | 1.7 (-0.3 to 4.5) | 1.6 (-0.2 to 4.8) | 17 (-2.4 to 43) | 9.4 (-1.3 to 25) | 7.7 (-1.1 to 20.2) | 412.5 (230 to 636.3) | 440 (216.1 to 681.2) | 382.5 (194.4 to 697.3) |
|  | Smoking | DALYs (Disability-Adjusted Life Years) | Age-standardized | 3.9 (2.2 to 6) | 0.7 (0.3 to 1.2) | 7.3 (4.1 to 11.2) | 3.9 (2.1 to 5.9) | 0.6 (0.3 to 1.1) | 8 (4.3 to 12.3) | -1.4 (-25 to 33.4) | -18.7 (-54.2 to 32.8) | 8.7 (-19.7 to 53.4) |
|  |  |  | All ages | 167.9 (94.7 to 250.1) | 14 (7.1 to 23.6) | 153.9 (87.5 to 229.1) | 470.1 (259.5 to 713.9) | 36.8 (18.2 to 69.2) | 433.2 (238.2 to 653.1) | 179.9 (117.5 to 263.6) | 163.2 (56.3 to 311) | 181.5 (118.1 to 268.1) |
|  |  | Deaths | Age-standardized | 0.1 (0.1 to 0.2) | 0 (0 to 0.1) | 0.3 (0.1 to 0.5) | 0.2 (0.1 to 0.3) | 0 (0 to 0.1) | 0.4 (0.2 to 0.6) | 16.1 (-32.2 to 98.8) | -15.7 (-66.5 to 69.2) | 32.5 (-28.1 to 144.6) |
|  |  |  | All ages | 4.5 (2.5 to 7.4) | 0.5 (0.2 to 0.9) | 4.1 (2.2 to 6.8) | 17.1 (8.5 to 27.9) | 1.6 (0.6 to 3.5) | 15.5 (7.8 to 24.9) | 277.7 (131.8 to 507.5) | 239.2 (44.8 to 551.1) | 282.2 (129.8 to 527.7) |
| United Arab Emirates | All risk factors | DALYs (Disability-Adjusted Life Years) | Age-standardized | 32.1 (15 to 50.1) | 34.1 (14.5 to 57) | 30.5 (14.3 to 47.6) | 36.7 (20 to 53.6) | 99.5 (50 to 154.1) | 24.2 (13 to 36.3) | 14.3 (-12.7 to 61.9) | 191.7 (90.4 to 349) | -20.7 (-38 to 20.8) |
|  |  |  | All ages | 97.3 (49.5 to 146.4) | 38.7 (17.1 to 64.1) | 58.6 (30.7 to 94.5) | 796.7 (449.6 to 1225.1) | 243.5 (133.6 to 358.3) | 553.2 (300.7 to 879.9) | 718.6 (493 to 1093.8) | 528.9 (320 to 856.8) | 843.9 (587 to 1274.4) |
|  |  | Deaths | Age-standardized | 1.4 (0.6 to 2.4) | 1.6 (0.6 to 2.9) | 1.3 (0.5 to 2.1) | 1.6 (0.8 to 2.5) | 6.6 (3.2 to 10.5) | 0.7 (0.4 to 1.2) | 13.8 (-27.1 to 81.8) | 322.6 (151.8 to 602.8) | -42.4 (-67 to 16.6) |
|  |  |  | All ages | 2.7 (1.2 to 4.6) | 1.5 (0.6 to 2.7) | 1.3 (0.5 to 2.2) | 13.4 (7.4 to 20.2) | 8 (4 to 12.1) | 5.4 (3.1 to 8.2) | 389.2 (228 to 699.3) | 446.3 (222.8 to 824.1) | 324 (184 to 668) |
|  | Alcohol use | DALYs (Disability-Adjusted Life Years) | Age-standardized | 0.9 (0.5 to 1.5) | 0.2 (0 to 0.3) | 1.5 (0.8 to 2.4) | 0.7 (0.4 to 1) | 0.2 (0 to 0.4) | 0.8 (0.4 to 1.2) | -26.5 (-56.2 to 19.8) | 19.4 (-63.1 to 183.8) | -45.6 (-67.7 to -13.3) |
|  |  |  | All ages | 4.3 (2.3 to 6.8) | 0.2 (0.1 to 0.4) | 4 (2.3 to 6.5) | 30.3 (16 to 47.6) | 0.7 (0.3 to 1.3) | 29.6 (15.6 to 46.8) | 610.9 (356 to 1010.4) | 234.5 (45.1 to 655.2) | 630.9 (366.1 to 1031.2) |
|  |  | Deaths | Age-standardized | 0 (0 to 0.1) | 0 (0 to 0) | 0.1 (0 to 0.1) | 0 (0 to 0) | 0 (0 to 0) | 0 (0 to 0) | -41 (-70.9 to 18.7) | 81.3 (-48.5 to 370.7) | -62.5 (-80.7 to -21.7) |
|  |  |  | All ages | 0.1 (0 to 0.1) | 0 (0 to 0) | 0.1 (0 to 0.1) | 0.3 (0.1 to 0.4) | 0 (0 to 0) | 0.2 (0.1 to 0.4) | 246.1 (89.9 to 563.2) | 189.6 (0.3 to 662.8) | 252 (94.7 to 591.6) |
|  | Diet high in sodium | DALYs (Disability-Adjusted Life Years) | Age-standardized | 0.5 (0 to 3) | 0.3 (0 to 2.4) | 0.6 (0 to 3.4) | 0.5 (0 to 3.2) | 0.8 (0 to 6.2) | 0.5 (0 to 2.6) | 6.4 (-93.2 to 698.2) | 131.2 (-2164.8 to 194428.3) | -23.8 (-95.6 to 524.7) |
|  |  |  | All ages | 1.6 (0 to 9.5) | 0.4 (0 to 3) | 1.2 (0 to 6.4) | 13.4 (0 to 72.1) | 2.3 (0 to 17.7) | 11.1 (0 to 57.3) | 726.1 (-40.7 to 4085.6) | 461.7 (-8812.5 to 2959149) | 813.7 (-40.8 to 4080.6) |
|  |  | Deaths | Age-standardized | 0 (0 to 0.1) | 0 (0 to 0.1) | 0 (0 to 0.1) | 0 (0 to 0.1) | 0 (0 to 0.4) | 0 (0 to 0.1) | -0.2 (-97.5 to 633.3) | 233 (-2662.1 to 261928.6) | -44.2 (-98.4 to 529.7) |
|  |  |  | All ages | 0 (0 to 0.2) | 0 (0 to 0.1) | 0 (0 to 0.1) | 0.2 (0 to 1.2) | 0.1 (0 to 0.6) | 0.1 (0 to 0.6) | 375.4 (-69.4 to 2379.7) | 430.5 (-2882.7 to 2203302) | 343.6 (-69.4 to 2379.8) |
|  | High body-mass index | DALYs (Disability-Adjusted Life Years) | Age-standardized | 5.9 (2.2 to 10.8) | 7.6 (3 to 13.9) | 4.6 (1.6 to 8.4) | 17.9 (8.2 to 30.7) | 50.6 (22.2 to 87.4) | 11.2 (4.7 to 19.4) | 202.2 (99.5 to 311.6) | 561.3 (309.1 to 894) | 142.3 (37.5 to 287.6) |
|  |  |  | All ages | 20.7 (8.2 to 38.2) | 9.6 (3.8 to 17.4) | 11.1 (4.4 to 21.2) | 437 (199.7 to 738.9) | 143.9 (69.1 to 230) | 293.2 (126.8 to 523.2) | 2011.2 (1391.3 to 2798.1) | 1397.9 (841.7 to 2102.9) | 2542.3 (1672.6 to 3691.2) |
|  |  | Deaths | Age-standardized | 0.2 (0.1 to 0.4) | 0.3 (0.1 to 0.6) | 0.2 (0.1 to 0.4) | 0.8 (0.3 to 1.4) | 3.3 (1.3 to 5.7) | 0.3 (0.1 to 0.7) | 230 (73.1 to 451.8) | 980.9 (483.5 to 1795.1) | 86.6 (-28 to 327.5) |
|  |  |  | All ages | 0.5 (0.2 to 1) | 0.3 (0.1 to 0.6) | 0.2 (0.1 to 0.4) | 7 (3.1 to 11.7) | 4.3 (2 to 7.3) | 2.7 (1.1 to 4.8) | 1265.7 (769.7 to 1943.9) | 1289.5 (675.3 to 2119.1) | 1229 (662.5 to 2307.2) |
|  | High systolic blood pressure | DALYs (Disability-Adjusted Life Years) | Age-standardized | 27.4 (9.3 to 45.4) | 29.9 (9.7 to 53.8) | 25.4 (8.5 to 42.8) | 25.7 (9.2 to 43.2) | 72.8 (23.8 to 124.5) | 16.3 (5.3 to 29) | -6.3 (-29.8 to 19.5) | 143.2 (60.9 to 246.7) | -35.9 (-51.4 to -14.7) |
|  |  |  | All ages | 77 (25.4 to 127) | 33.3 (10.8 to 59.7) | 43.7 (14.1 to 77.8) | 477.1 (152.3 to 840.3) | 162 (54.2 to 274.7) | 315.1 (97.3 to 600.2) | 519.5 (354.1 to 701.4) | 386.4 (208.4 to 574.4) | 621 (404.9 to 868) |
|  |  | Deaths | Age-standardized | 1.2 (0.4 to 2.2) | 1.4 (0.4 to 2.7) | 1.1 (0.3 to 1.9) | 1.2 (0.4 to 2) | 4.9 (1.6 to 8.5) | 0.5 (0.2 to 1) | -6.6 (-39.8 to 38.6) | 247.7 (106 to 432.1) | -53.4 (-73.5 to -12.5) |
|  |  |  | All ages | 2.3 (0.8 to 4.2) | 1.3 (0.4 to 2.5) | 1 (0.3 to 1.9) | 9 (3.1 to 15.4) | 5.6 (1.8 to 9.7) | 3.4 (1.1 to 6.2) | 287.8 (157.6 to 447.6) | 337.7 (153.6 to 576.4) | 226 (116.9 to 436.7) |
|  | Lead exposure | DALYs (Disability-Adjusted Life Years) | Age-standardized | 1.8 (-0.3 to 4.7) | 1.8 (-0.3 to 4.8) | 1.8 (-0.2 to 4.6) | 1.7 (-0.2 to 4.4) | 4.3 (-0.6 to 11.3) | 1.3 (-0.2 to 3.3) | -1.8 (-27 to 22.6) | 143.5 (61.7 to 238.4) | -29.2 (-44.9 to -4) |
|  |  |  | All ages | 5.2 (-0.7 to 13.6) | 2 (-0.3 to 5.4) | 3.3 (-0.4 to 8.7) | 26.8 (-3.3 to 70.4) | 8.1 (-1.1 to 21.2) | 18.7 (-2.3 to 50.8) | 411.8 (289.9 to 547.9) | 310.4 (164.3 to 455.7) | 473.2 (315.3 to 651.5) |
|  |  | Deaths | Age-standardized | 0.1 (0 to 0.2) | 0.1 (0 to 0.2) | 0.1 (0 to 0.2) | 0.1 (0 to 0.2) | 0.3 (0 to 0.8) | 0 (0 to 0.1) | 0.8 (-38.9 to 49.6) | 267.9 (120.4 to 463.4) | -43.1 (-69.8 to 12.6) |
|  |  |  | All ages | 0.1 (0 to 0.4) | 0.1 (0 to 0.2) | 0.1 (0 to 0.2) | 0.5 (-0.1 to 1.4) | 0.3 (0 to 0.8) | 0.2 (0 to 0.6) | 258.3 (130.5 to 385.3) | 301.1 (126.8 to 515.3) | 215.4 (111.4 to 416.9) |
|  | Smoking | DALYs (Disability-Adjusted Life Years) | Age-standardized | 2.3 (1.3 to 3.6) | 0.8 (0.4 to 1.4) | 3.3 (1.8 to 5.3) | 2.1 (1.2 to 3.4) | 1.2 (0.6 to 2.2) | 2.4 (1.3 to 3.8) | -5.1 (-26.6 to 23) | 53.8 (-10.2 to 173.6) | -27.1 (-44.3 to -5.2) |
|  |  |  | All ages | 12.3 (6.7 to 20.5) | 1.1 (0.6 to 1.9) | 11.2 (6 to 18.7) | 100.9 (48.6 to 168.7) | 6.3 (3.3 to 10.2) | 94.6 (44.5 to 157.7) | 720.6 (484.8 to 1008) | 465.6 (228.2 to 810.6) | 746 (485.8 to 1076.5) |
|  |  | Deaths | Age-standardized | 0.1 (0 to 0.1) | 0 (0 to 0.1) | 0.1 (0.1 to 0.2) | 0.1 (0 to 0.1) | 0.1 (0 to 0.1) | 0.1 (0 to 0.1) | -15 (-45.8 to 43.8) | 155.3 (24.1 to 436) | -45.2 (-65.4 to 0.6) |
|  |  |  | All ages | 0.2 (0.1 to 0.3) | 0 (0 to 0.1) | 0.2 (0.1 to 0.3) | 0.8 (0.5 to 1.4) | 0.1 (0.1 to 0.2) | 0.7 (0.4 to 1.2) | 335.2 (190.1 to 592.5) | 307 (117.6 to 603.1) | 340.6 (184.6 to 634.6) |
| Yemen | All risk factors | DALYs (Disability-Adjusted Life Years) | Age-standardized | 21 (8.6 to 35) | 21.8 (8.5 to 39.9) | 19.8 (7.9 to 35.5) | 26.5 (12.9 to 41.9) | 27.7 (12 to 44.6) | 25.2 (12.2 to 41.9) | 26.5 (-4.6 to 83.8) | 26.7 (-10.2 to 93.6) | 27.6 (-5.2 to 97.9) |
|  |  |  | All ages | 734.6 (325.9 to 1255.8) | 428.9 (169.3 to 786.1) | 305.7 (132.2 to 518.7) | 2768.9 (1383.2 to 4296.8) | 1478 (667.9 to 2339.4) | 1290.9 (657.6 to 2079.3) | 276.9 (183.8 to 423.2) | 244.6 (141.9 to 418.6) | 322.3 (219.9 to 504.6) |
|  |  | Deaths | Age-standardized | 1 (0.3 to 1.8) | 1 (0.3 to 2.2) | 0.8 (0.2 to 1.6) | 1.3 (0.6 to 2.2) | 1.4 (0.6 to 2.5) | 1.1 (0.5 to 2) | 33.2 (-12.5 to 126) | 37 (-11.3 to 147) | 34.1 (-14.3 to 193) |
|  |  |  | All ages | 24.2 (8.6 to 45.8) | 16.5 (5.6 to 35.1) | 7.7 (2.1 to 15.1) | 101.8 (48.5 to 171.3) | 60.8 (24.5 to 106.8) | 41 (17.3 to 74.9) | 320 (177.6 to 590.4) | 268.7 (139.8 to 548.9) | 429.2 (242.5 to 983.6) |
|  | Alcohol use | DALYs (Disability-Adjusted Life Years) | Age-standardized | 0.3 (0.1 to 0.4) | 0.1 (0 to 0.1) | 0.5 (0.3 to 0.8) | 0.1 (0 to 0.1) | 0 (0 to 0) | 0.2 (0.1 to 0.3) | -68.8 (-79.6 to -52.2) | -80.1 (-90.8 to -61.4) | -70.7 (-81.1 to -54.7) |
|  |  |  | All ages | 12.2 (6.7 to 18.5) | 1.3 (0.7 to 2.2) | 10.9 (5.9 to 17.1) | 11.3 (6.2 to 18) | 0.7 (0.3 to 1.4) | 10.6 (5.9 to 17) | -6.9 (-37.5 to 38.5) | -43.5 (-72.8 to 4.8) | -2.6 (-34.9 to 43.4) |
|  |  | Deaths | Age-standardized | 0 (0 to 0) | 0 (0 to 0) | 0 (0 to 0) | 0 (0 to 0) | 0 (0 to 0) | 0 (0 to 0) | -67.4 (-82.3 to -36.3) | -79.2 (-92.2 to -53.6) | -72.7 (-85.4 to -40) |
|  |  |  | All ages | 0.3 (0.1 to 0.5) | 0 (0 to 0.1) | 0.2 (0.1 to 0.4) | 0.3 (0.1 to 0.5) | 0 (0 to 0) | 0.2 (0.1 to 0.4) | -3.7 (-44.8 to 80) | -42.6 (-75.6 to 22.2) | 3.6 (-40 to 108.3) |
|  | Diet high in sodium | DALYs (Disability-Adjusted Life Years) | Age-standardized | 0.3 (0 to 2.1) | 0.2 (0 to 1.9) | 0.5 (0 to 2.6) | 0.4 (0 to 2.4) | 0.3 (0 to 2) | 0.5 (0 to 2.9) | 13.7 (-92.3 to 959.3) | 6.2 (-269183 to 2173.8) | 8.7 (-91.5 to 919.2) |
|  |  |  | All ages | 13.5 (0 to 75) | 5.1 (0 to 37.5) | 8.4 (0 to 40.1) | 43.7 (0 to 262.8) | 14.8 (0 to 112.6) | 29 (0 to 154) | 223.5 (-62.2 to 2959.6) | 186.9 (-2480784.9 to 6727) | 246.1 (-62.2 to 2959.6) |
|  |  | Deaths | Age-standardized | 0 (0 to 0.1) | 0 (0 to 0.1) | 0 (0 to 0.1) | 0 (0 to 0.1) | 0 (0 to 0.1) | 0 (0 to 0.1) | 24.4 (-94.8 to 1476.8) | 14.7 (-81249 to 3356.7) | 14.9 (-95.2 to 1510.7) |
|  |  |  | All ages | 0.4 (0 to 2.3) | 0.2 (0 to 1.3) | 0.2 (0 to 1.1) | 1.4 (0 to 8.8) | 0.5 (0 to 4.3) | 0.8 (0 to 4.8) | 262.9 (-80 to 3701.8) | 202.7 (-832989.2 to 6863.9) | 317 (-80 to 3705.3) |
|  | High body-mass index | DALYs (Disability-Adjusted Life Years) | Age-standardized | 1.4 (0.4 to 3.1) | 2.2 (0.7 to 4.8) | 0.4 (0.1 to 1) | 6.2 (2.5 to 11.3) | 6.4 (2.6 to 11.8) | 6 (2.2 to 11.9) | 331.9 (155.8 to 751.2) | 193.1 (75 to 491) | 1330.8 (636.4 to 4666) |
|  |  |  | All ages | 58.7 (19.5 to 122.9) | 48.9 (16.3 to 106.1) | 9.9 (2 to 21.9) | 678 (268.8 to 1171.7) | 361.2 (139.1 to 650.7) | 316.8 (114.2 to 589.7) | 1054.6 (644.4 to 2030.2) | 639.3 (365.2 to 1279.3) | 3110.7 (1705.6 to 8854.2) |
|  |  | Deaths | Age-standardized | 0.1 (0 to 0.1) | 0.1 (0 to 0.2) | 0 (0 to 0) | 0.3 (0.1 to 0.6) | 0.3 (0.1 to 0.6) | 0.3 (0.1 to 0.6) | 389.7 (130.5 to 1331.5) | 266.5 (60.8 to 955.3) | 2383.3 (650.4 to 17645.4) |
|  |  |  | All ages | 1.7 (0.5 to 3.8) | 1.5 (0.4 to 3.5) | 0.2 (0 to 0.5) | 23.6 (9.2 to 44.9) | 13.9 (5.1 to 26) | 9.7 (3 to 20.4) | 1271 (616.9 to 2912.4) | 803.6 (377.3 to 1994.5) | 5174.9 (2351 to 20776.6) |
|  | High systolic blood pressure | DALYs (Disability-Adjusted Life Years) | Age-standardized | 18.1 (5.8 to 33.2) | 19.8 (6.5 to 37.8) | 15.8 (4 to 31.7) | 21.2 (7.3 to 36.6) | 23.5 (7.9 to 41.2) | 18.7 (6.1 to 34.9) | 17.2 (-13.6 to 66.6) | 18.6 (-15.9 to 85.3) | 18.4 (-16.5 to 86.4) |
|  |  |  | All ages | 604.3 (193 to 1123.9) | 382.3 (128.2 to 742.6) | 222 (54.1 to 428.3) | 2138.4 (730.9 to 3639) | 1232 (415 to 2124.8) | 906.4 (286.9 to 1699.8) | 253.9 (164.1 to 392.5) | 222.3 (123.1 to 395) | 308.2 (193.7 to 513.8) |
|  |  | Deaths | Age-standardized | 0.9 (0.2 to 1.7) | 1 (0.3 to 2.1) | 0.7 (0.1 to 1.5) | 1.1 (0.4 to 1.9) | 1.2 (0.4 to 2.3) | 0.9 (0.3 to 1.8) | 22.3 (-19.2 to 103) | 27.6 (-18.7 to 134.1) | 23.1 (-25 to 159.2) |
|  |  |  | All ages | 21.2 (6.2 to 42.2) | 15.1 (4.7 to 33) | 6.1 (1.1 to 13.3) | 82.9 (27.9 to 149.9) | 52 (16.8 to 97.8) | 30.9 (9.4 to 63.8) | 290.7 (158.7 to 525.4) | 244.1 (123 to 517.4) | 405.8 (219.6 to 962.1) |
|  | Lead exposure | DALYs (Disability-Adjusted Life Years) | Age-standardized | 3.9 (-0.6 to 9.9) | 3.6 (-0.6 to 9.7) | 4.4 (-0.7 to 12.1) | 4.8 (-0.7 to 12.1) | 4.4 (-0.7 to 11.6) | 5.1 (-0.8 to 13.7) | 22.9 (-7.7 to 63.4) | 22.4 (-14 to 73.1) | 17.2 (-7.3 to 66.7) |
|  |  |  | All ages | 134 (-20.8 to 348.6) | 70.4 (-11.3 to 185.6) | 63.5 (-9.7 to 177.3) | 466.7 (-71.3 to 1180.5) | 224.5 (-34.1 to 582.2) | 242.2 (-37 to 639.5) | 248.4 (166.3 to 354.6) | 218.7 (120.7 to 350) | 281.2 (202.2 to 418.6) |
|  |  | Deaths | Age-standardized | 0.2 (0 to 0.5) | 0.2 (0 to 0.5) | 0.2 (0 to 0.6) | 0.2 (0 to 0.6) | 0.2 (0 to 0.6) | 0.2 (0 to 0.7) | 37.5 (-10.8 to 111) | 39.5 (-11 to 122.5) | 27.7 (-14.1 to 157.6) |
|  |  |  | All ages | 4.4 (-0.7 to 12) | 2.7 (-0.4 to 7.5) | 1.7 (-0.2 to 5.2) | 18.5 (-3 to 49.2) | 9.9 (-1.5 to 26.4) | 8.5 (-1.4 to 23.8) | 317.4 (177.5 to 544.8) | 264.1 (133.9 to 469.6) | 402.9 (247.6 to 877.8) |
|  | Smoking | DALYs (Disability-Adjusted Life Years) | Age-standardized | 2.7 (1.5 to 4.2) | 1.3 (0.6 to 2.2) | 4.5 (2.4 to 7.4) | 3 (1.6 to 4.9) | 1.4 (0.7 to 2.4) | 4.7 (2.4 to 7.8) | 13.3 (-13.9 to 53.4) | 11.6 (-30.3 to 75.7) | 4.3 (-21.9 to 50.2) |
|  |  |  | All ages | 122.5 (68.1 to 192.7) | 30 (14.7 to 52.8) | 92.5 (49.1 to 150.3) | 392.4 (213 to 639.8) | 94.8 (45.8 to 158.5) | 297.6 (160.3 to 487.1) | 220.4 (143.9 to 325.1) | 215.8 (108.2 to 374.3) | 221.9 (147.2 to 341.2) |
|  |  | Deaths | Age-standardized | 0.1 (0 to 0.1) | 0 (0 to 0.1) | 0.1 (0 to 0.3) | 0.1 (0 to 0.2) | 0.1 (0 to 0.1) | 0.2 (0.1 to 0.3) | 28.7 (-19.3 to 131.6) | 22.6 (-39.5 to 143.7) | 8.4 (-31.1 to 124.3) |
|  |  |  | All ages | 2.7 (1.2 to 4.6) | 0.9 (0.4 to 1.7) | 1.8 (0.7 to 3.5) | 9.9 (4.7 to 17.1) | 2.9 (1.1 to 5) | 7 (3.2 to 12.9) | 264.5 (137.8 to 529.5) | 231 (84.4 to 492.1) | 280.2 (143.5 to 633.3) |
